# Mechanism of the palladium-catalyzed diazenylation of aryl electrophiles: carbonate-facilitated transmetalation and ligand-dependent selectivity

**DOI:** 10.1039/d6sc04320h

**Published:** 2026-06-22

**Authors:** Torben Rogge, Wolfgang Obermayer, Martin Oestreich

**Affiliations:** a Institut für Chemie, Technische Universität Berlin Strasse des 17. Juni 115 10623 Berlin Germany martin.oestreich@tu-berlin.de torben.rogge@chem.tu-berlin.de

## Abstract

A quantum-chemical investigation of the palladium-catalyzed cross-coupling of silylated diazenes as diazenyl anion equivalents and aryl electrophiles is reported. The selectivity, that is azobenzene formation by diazenylation *versus* biaryl formation by denitrogenative coupling, is largely controlled by the diphosphine ligand employed, with dppf suppressing any loss of dinitrogen. This experimental observation is further corroborated by a screening of representative ligands. The key role of the base additive is also clarified by density functional theory calculations. These reveal an unusual two-step transmetalation process characterized by a barrierless desilylation event facilitated by a silaphilic carbonate base. From this, a full mechanistic picture of a rare C(sp^2^)–N(sp^2^) cross-coupling reaction emerges.

## Abstract 2

A quantum-chemical investigation of the palladium-catalyzed cross-coupling of silylated diazenes as diazenyl anion equivalents reveals an unusual two-step transmetalation process and a ligand-dependent switch in chemoselectivity.

## Graphical abstract

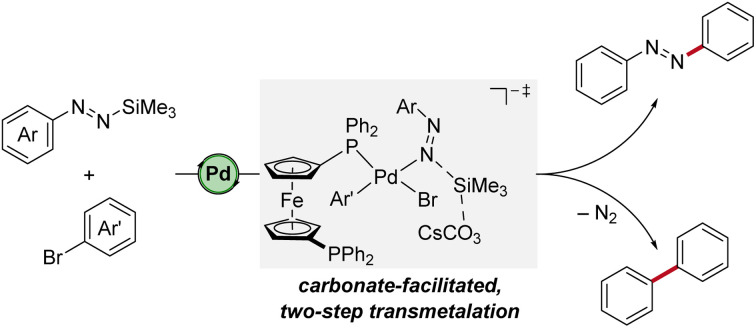

## Introduction

Azobenzenes are an important class of organic compounds that have found widespread application as organic dyes, molecular photoswitches, photoresponsive materials, and pharmaceutical agents.^1–6^ While the synthesis of symmetric azobenzenes can be considered established, the synthesis of their non-symmetric and heteroaryl-containing derivatives is still being investigated. Over the years, a number of methods for the synthesis of non-symmetric azoarenes have been developed.^7^ Among others,^8–13^ recent examples include the addition of organozinc reagents to diazonium salts,^14,15^ Buchwald–Hartwig cross-coupling reactions of aryl-substituted hydrazines and aryl electrophiles followed by oxidation,^16,17^ and the cross-coupling of aryl diazonium salts with arylboronic acids.^18^

**Scheme 1:**
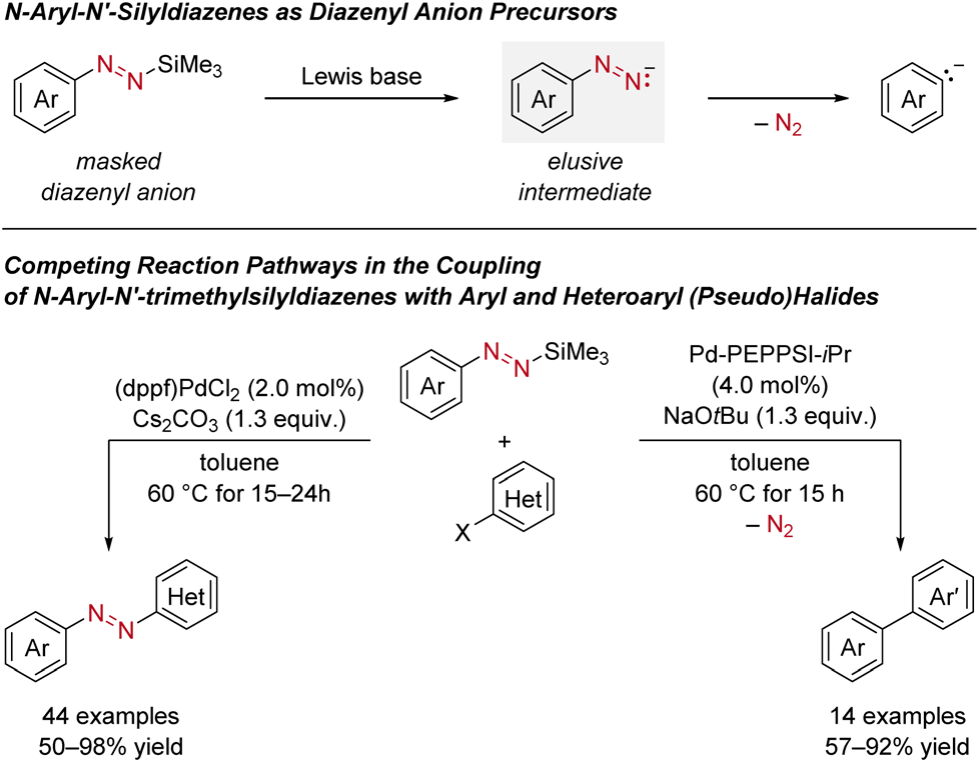
*N*-Aryl *N*′-silylated diazenes as masked precursors to elusive diazenyl anions and their engagement in cross-coupling reactions.

Another potential pathway to directly access non-symmetric azoarenes is the coupling of diazenyl anions and arene electrophiles. However, in nearly all cases “free” diazenyl anions rapidly decompose into the corresponding carbon nucleophiles under liberation of dinitrogen before capture by an electrophile.^19^*N*-Aryl-*N*′-silyldiazenes are easy-to-handle diazenyl anion precursors which release those elusive intermediates after desilylation by a silaphilic Lewis base (Scheme 1, top).^19,20^ These compounds were first used by Bottaro in 1978, who used them in conjunction with sodium methoxide as the Lewis base for the arylation of aldehydes and ketones under dinitrogen extrusion.^21^ In 2020, this strategy was rediscovered by our laboratory and put into broader context, *i.e.* the 1,2-addition of functionalized aryl pronucleophiles to carbonyl compounds catalyzed by alkali metal silanolates.^22^ Applications to other electrophiles again with loss of dinitrogen followed.^23–25^ In 2022, we accomplished an intriguing cross-coupling of *N*-aryl *N*′-silylated diazenes and aryl electrophiles with no loss of dinitrogen to afford azobenzenes (Scheme 1, bottom left).^26^ At the time, this was an unprecedented example of a reaction formally involving a diazenyl anion where the azo unit is retained in the product molecules. The dppf as the ligand in precatalyst (dppf)PdCl_2_ and the use of cesium carbonate were found to be crucial. Using Pd-PEPPSI-*i*Pr as a precatalyst and sodium *tert*-butoxide as the Lewis base, dinitrogen extrusion was observed, and the corresponding biaryls were formed (Scheme 1, bottom right).^27^

That palladium-catalyzed diazenylation proved to be robust and remarkably general for the synthesis of non-symmetric azobenzenes,^26^ and we recently applied this novel diazenylation strategy to the kinetic resolution of biaryl triflates using chiral dppf congeners^28^ and to the coupling of azo groups to small peptides containing a triflated tyrosine residue.^29^ Despite this progress, a detailed understanding of the reaction mechanism, in particular the role of the diphosphine ligand and the base additive, are missing. We now report on an in-depth mechanistic investigation of the palladium-catalyzed diazenylation of aryl halides by computation with experimental support.

## Results and discussion

Starting from the previously noted considerable influence of the employed phosphine ligand on the reaction outcome,^26^ we initiated our studies by systematically testing a variety of representative bidentate phosphines in the presence of cesium carbonate base^30^ in toluene (Table 1). While almost exclusive formation of the azobenzene 3aa was observed with (dppf)PdCl_2_ as precatalyst, the use of the corresponding Xantphos and DPEphos complexes resulted in a markedly diminished catalytic efficacy, which can likely be attributed to a decreased ability of these ligands to undergo a change in denticity (entries 1–3). In addition, dtbpf as a sterically demanding variant of dppf, led to a mixture of products 3aa and 4aa (entry 4). In turn, dppb with a smaller bite angle^31,32^ resulted in a complete switch in selectivity, furnishing biaryl 4aa as the sole product (entry 5). At reduced temperatures of 40 °C or room temperature, formation of product 3aa was also observed, albeit in markedly diminished yields of 16% and 2%, respectively (entries 6–7).

**Table 1:** Screening of diphosphine ligands^a^

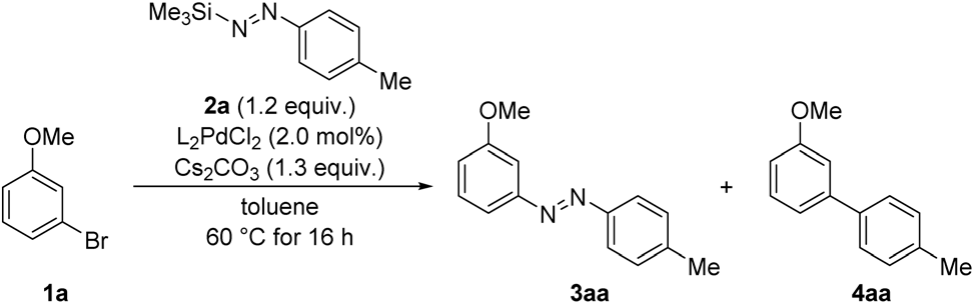				
Entry	L_2_PdCl_2_	Bite angle (°)	Yield of 3aa (%)	Yield of 4aa (%)
1	(dppf)PdCl_2_	99	99	1
2^b^	(Xantphos)PdCl_2_	108	1	1
3	(DPEphos)PdCl_2_	104	8	16
4^b^^,^^c^	(dtbpf)PdCl_2_	104	16	35
5^b^^,^^d^	(dppb)PdCl_2_	94	0	56
6^e^	(dppf)PdCl_2_	99	16	0
7^f^	(dppf)PdCl_2_	99	2	0

a Reaction conditions: 1a (0.10 mmol), 2a (1.2 equiv.), L_2_PdCl_2_ (2.0 mol%), Cs_2_CO_3_ (1.3 equiv.), toluene (0.2 mL) at 60 °C for 16 h. Yields were determined by GLC analysis with tetracosane as an internal standard.

b 48 h reaction time.

c Data from ref. 26.

d Data from ref. 33.

e Performed at 40 °C for 24 h.

f Performed at r.t. for 24 h. dppf, 1,1′-bis(diphenylphosphino)ferrocene; dtbpf, 1,1′-bis(di-*tert*-butylphosphino)-ferrocene; dppb, 1,4-bis(diphenylphosphino)butane.

**Fig. 1:**
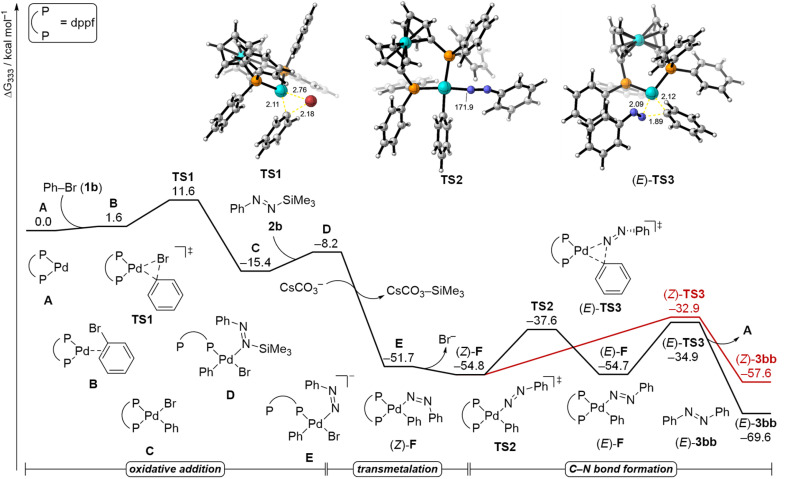
Calculated Gibbs free energy diagram (in kcal mol^−1^) for the formation of azobenzene 3bb. Distances are given in Å and angles in °.

**Fig. 2:**
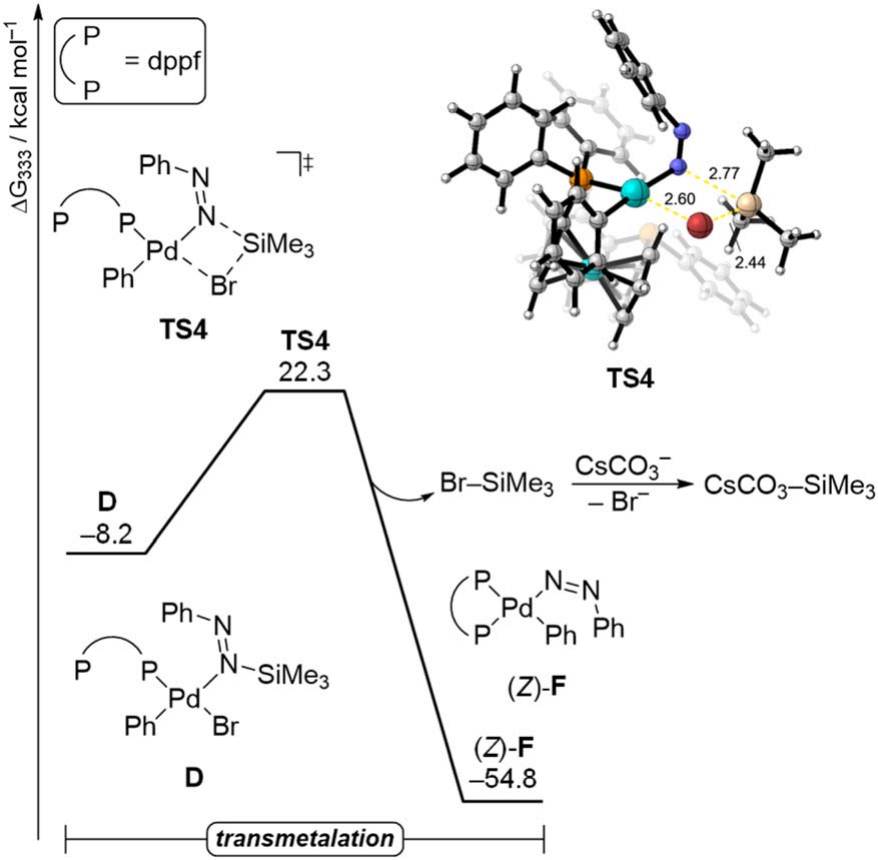
Calculated Gibbs free energy diagram (in kcal mol^−1^) relative to A for transmetalation without carbonate assistance. Distances are given in Å.

To delineate the reaction mechanism, we performed density functional theory (DFT) studies at the ωB97X-D/def2-TZVP+ SMD(toluene)//TPSS-D3(BJ)/def2-SVP level of theory for the model reaction of *N*-phenyl-*N*′-trimethylsilyldiazene (2b) and bromobenzene (1b).^34–40^ Starting from palladium(0) complex (dppf)Pd^0^ (A), the transformation is initiated by a slightly endergonic coordination of bromobenzene (1b) to palladium, generating intermediate B (Fig. 1). Thereafter, oxidative addition readily takes place *via* transition state TS1 with a Gibbs free energy of activation of only 11.6 kcal mol^−1^, yielding tetracoordinated palladium(ii) intermediate C, which was found to be 15.4 kcal mol^−1^ more stable than complex A. Next, coordination of silyldiazene 2b through the silicon-bonded nitrogen atom and concurrent decoordination of one phosphine donor of dppf occurs to form intermediate D. By this change in denticity, the unfavorable formation of a pentacoordinated palladium(ii) species is avoided, highlighting the crucial hemilabile nature of dppf. Upon coordination of 2b to palladium, an elongation of the N–Si bond by 0.05 Å was observed, indicating a decrease in bond strength, thus allowing for an easier desilylation (Fig. S5 in the SI). In contrast, alternative modes of coordination such as coordination *via* the other, phenyl-substituted nitrogen atom of diazene 2b were found to be energetically unfavorable. Then, transmetalation, that is simultaneous cleavage of the N–Si bond and formation of a new Si–Br bond, presumably proceeds. Of note, a typical four-membered cyclic transition state TS4 was found to be energetically prohibitive with an overall energy barrier of >35 kcal mol^−1^ (Fig. 2). However, when mono-anionic cesium carbonate (CsCO_3_^−^) was included in the calculations, a barrierless cleavage of the N–Si bond and very favorable formation of CsCO_3_SiMe_3_ through an outer-sphere process was observed (see also Scheme S1 and Fig. S6 in the SI). In contrast, desilylation *via* an inner-sphere pathway, *i.e.* C–N bond cleavage facilitated through a carbonate ligand coordinated to palladium,^41^ was found to be in principle feasible, but required a considerable Gibbs free energy of activation of 27.1 kcal mol^−1^ (Fig. S8 in the SI). Overall, these results are in good agreement with the experimentally observed key role of silaphilic bases for catalytic efficacy.^42^ Subsequently, decoordination of bromide from intermediate E results in the formation of (*Z*)-configured intermediate (*Z*)-F, which can readily undergo isomerization *via* transition state TS2 to form isoenergetic intermediate (*E*)-F. Finally, azobenzene (*E*)-3bb is formed through turnover-limiting reductive elimination from intermediate (*E*)-F*via* transition state (*E*)-TS3, concurrently regenerating complex A. In contrast, formation of the thermodynamically less stable (*Z*)-configured azobenzene (*Z*)-3bb by means of reductive elimination from complex (*Z*)-F was found to be disfavored by 1.9 kcal mol^−1^ (Fig. 1, shown in red).

**Fig. 3:**
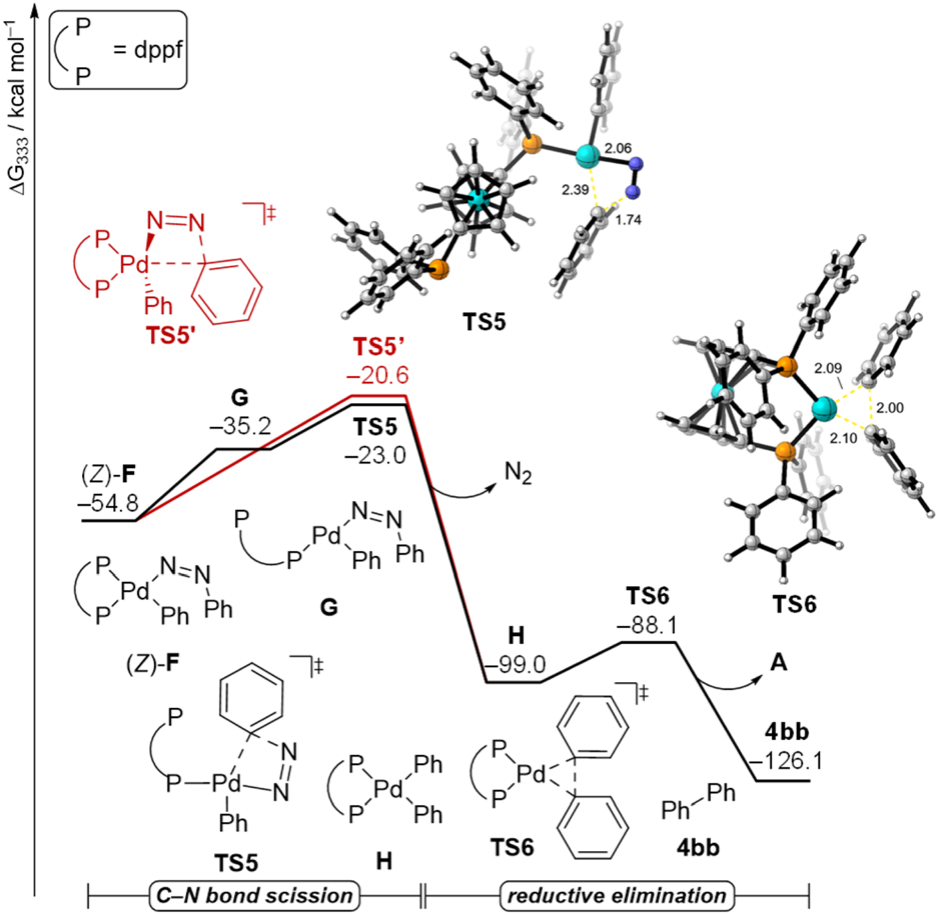
Calculated Gibbs free energy diagram (in kcal mol^−1^) relative to A for the formation of biphenyl (4bb) *via* denitrogenation. Distances are given in Å.

The mechanism of the competing denitrogenative formation of biphenyl (4bb) was elucidated next (Fig. 3). Within this pathway, intermediate (*Z*)-F undergoes C(sp^2^)–N bond cleavage *via* transition state TS5′. Alternatively, a prior change in dppf denticity from bidentate to monodentate coordination generates intermediate G, which allows for a slightly easier C(sp^2^)–N bond scission through TS5, being preferred over TS5′ by 2.4 kcal mol^−1^. Following the highly exergonic formation of dinitrogen, C–C bond forming reductive elimination readily takes place *via* transition state TS6, thus generating biphenyl (4bb). In comparison, the overall energy barrier for the denitrogenative pathway is considerably higher than for azobenzene (3bb) formation, with 31.8 *versus* 19.8 kcal mol^−1^, and cannot be readily overcome at temperatures of only 60 °C. As such, the computational results are in good agreement with the experimentally observed strongly preferred formation of azobenzene when dppf is employed as ligand.

**Fig. 4:**
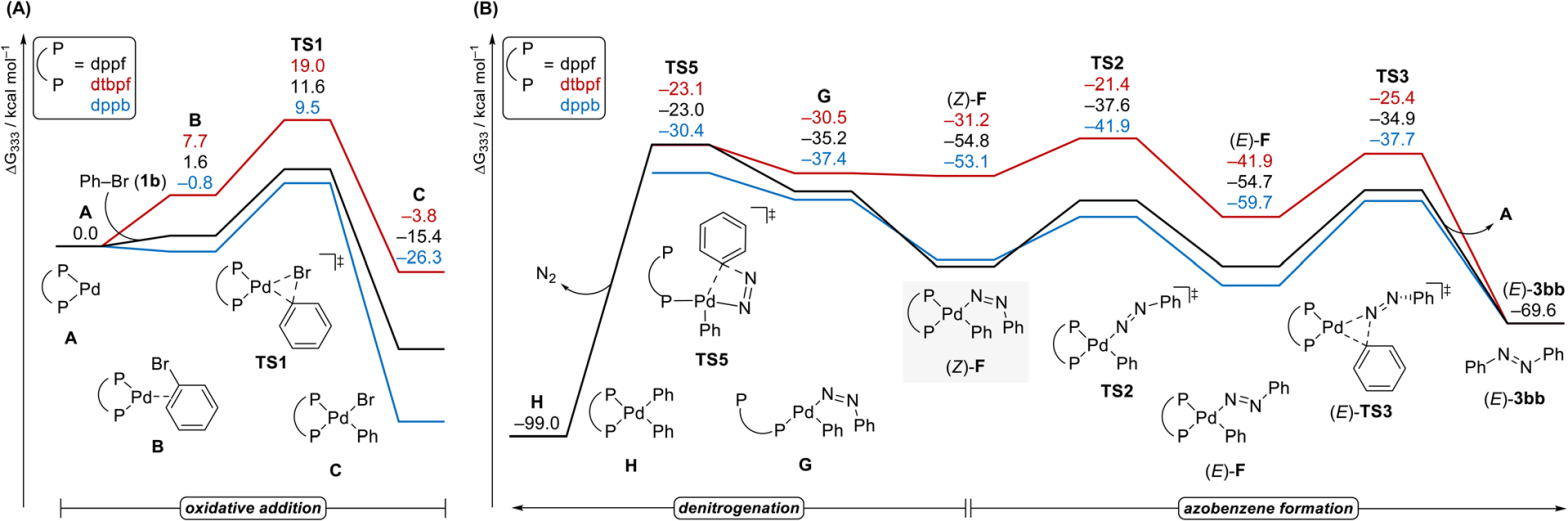
Calculated Gibbs free energy diagram (in kcal mol^−1^) relative to A for (A) oxidative addition of 1b, and (B) formation of azobenzene 3bb*versus* denitrogenation with dppf (black color), dtbpf (red color) and dppb (blue color) as ligands.

With the mechanism for formation of azobenzene 3 and biaryl 4 identified, we were interested in rationalizing the experimentally observed selectivity switch depending on the diphosphine ligand (*cf.*Table 1).^26,27,33^ To this end, the key elementary steps, *i.e.* oxidative addition, reductive elimination with no loss of dinitrogen, and denitrogenation, were investigated with both dtbpf and dppb as ligands *in lieu* of dppf (Fig. 4, S9 and S10 in the SI). Oxidative addition of 1b can readily take place with all three catalysts, although the larger steric bulk of dtbpf results in an increase in Gibbs free energy of activation by 7.4 kcal mol^−1^ compared to dppf. A distortion/interaction-activation strain analysis^43,44^ revealed that the higher Gibbs free energy of activation is mostly the result of an increased unfavorable distortion of 1b in the transition state caused by the ligand's bulky *tert*-butyl substituents (Fig. S11 in the SI).

For the selectivity-determining steps, a more complex picture emerges with dtbpf as ligand. Formation of azobenzene *via* reductive elimination as well as competing denitrogenation were found to be energetically feasible at temperatures of 60 °C, with a difference in Gibbs free energy of activation of only 2.3 kcal mol^−1^ and favoring azobenzene formation. It should be noted that, in contrast to dppf as ligand, the C–N bond cleavage preferentially occurs *via* a more compact transition state, placing both phenyl ligands in *cis*-position. However, a closer investigation revealed that *Z*-to-*E*-isomerization, which is a prerequisite for (*E*)-azobenzene formation, requires an increased Gibbs free energy of activation and is 1.6 kcal mol^−1^ higher in energy than competing denitrogenation *via*TS5. Thus, overall, azobenzene formation is energetically slightly less favorable compared to formation of biphenyl, thereby rationalizing the experimentally obtained mixture of products 3 and 4, with 4 being the main product. In addition, calculations with dppb as ligand revealed a slightly more facile oxidative addition process, which was attributed to an increase in stabilizing interaction energies (Fig. S11 in the SI). On the other hand, reductive elimination proved to be more challenging compared to dppf, with ΔΔ*G* = 2.0 kcal mol^−1^. Due to the high structural flexibility of the butylene backbone of dppb, only a relatively high lying C–N bond scission transition state could be located. Also, a bidentate coordination of dppb did not result in an easier C–N bond cleavage (Fig. S10 in the SI). In addition, dppb is typically not assumed to be a hemilabile ligand and as such might preclude the formation of some of the key intermediates, for example intermediate D, under the experimental reaction conditions. Therefore, a change in mechanism, *e.g.* to a bimetallic pathway, cannot be fully excluded.

**Fig. 5:**
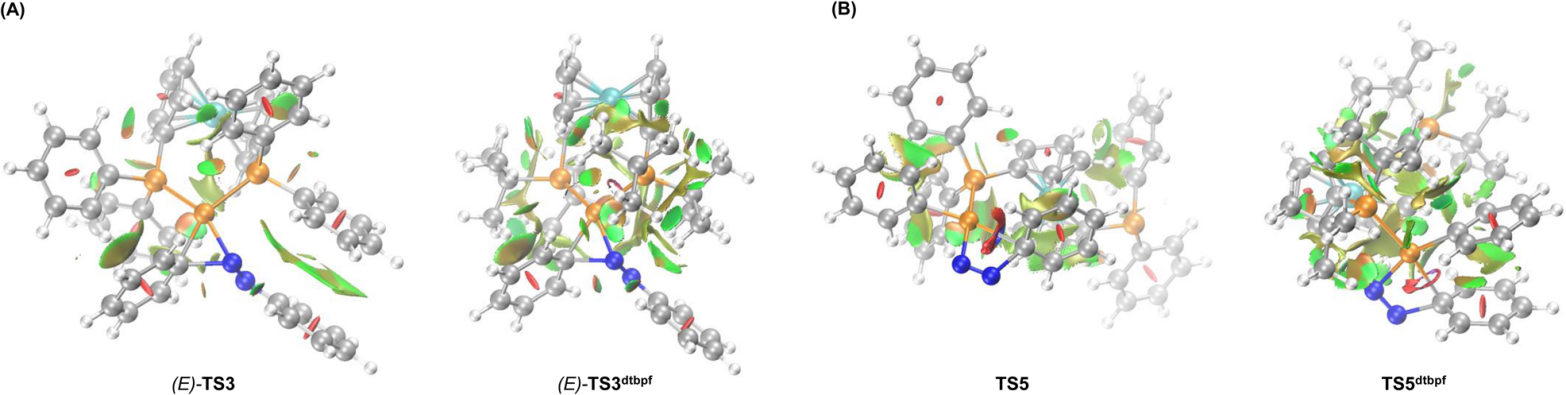
Non-covalent interaction (NCI) analysis for (A) transition state (*E*)-TS3, and (B) transition state TS5 with dppf and dtbpf as ligand. In the plotted surface (isovalue = 0.4), red corresponds to strong repulsive, green/green brown to weak attractive and blue to strong attractive interactions.

To gain further insights into the ligand-dependent switch in selectivity, a qualitative Non-Covalent Interaction (NCI) analysis was performed (Fig. 5 and S12 in the SI). For the azobenzene-forming reductive elimination transition state (*E*)-TS3 with dppf as well as dppb as ligands, the analysis revealed stabilizing π–π interactions between the phenyldiazene motif and one phenyl substituent of the diphosphine. In addition, C–H–π interactions between the phenyl ligand and another phenyl substituent of the phosphine ligand were detected. In contrast, for the corresponding dtbpf ligated complex, only not optimally oriented C–H–π interactions between the phenyldiazene ligand and the phosphine's *tert*-butyl groups were found. For the competing denitrogenation transition state TS5, in case of dppf as ligand, the NCI analysis revealed strong repulsive interactions between the palladium and the diazene's phenyl substituent, which can likely be attributed to the strained four-membered cyclic transition state geometry. On the other hand, when dtbpf is employed as ligand, these repulsive interactions are less pronounced. Furthermore, the different preferred orientation, with the phenyl ligand and the diazene's phenyl motif being in close proximity, allows for some stabilizing π–π interactions, thus rationalizing the reduced energy barrier for denitrogenation.

We were further interested in whether the stereoelectronic properties of the employed silyldiazene influence the selectivity of the reaction. To this end, a variety of substituted silyldiazenes 2 were investigated computationally and experimentally. Although silyldiazenes 2 bearing a methoxy-, fluoro- or trifluoromethyl substituent in *ortho*-position displayed a reduced energy barrier for denitrogenation of up to ΔΔ*G* = −7.0 kcal mol^−1^, formation of azobenzene 3 continued to be strongly preferred. Thus, no significant effect of the steric and electronic properties of 2 was predicted for the (dppf)Pd catalytic system, which was further corroborated by experiment (Table S2 in the SI).

**Scheme 2:**
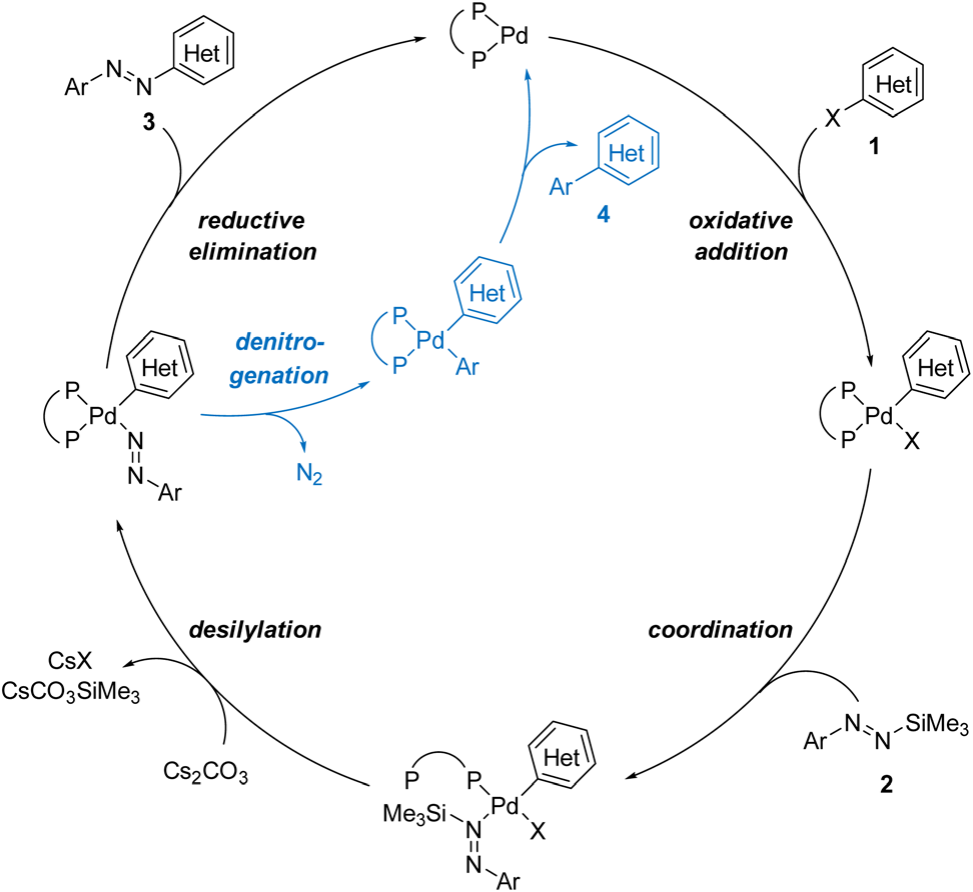
Catalytic cycle.

Based on the computational results, a simplified catalytic cycle is depicted in Scheme 2. After oxidative addition of aryl halide 1, transmetalation occurs stepwise by coordination of the silicon-substituted nitrogen terminus of diazene 2 to the arylpalladium(ii) intermediate followed by carbonate-assisted desilylation. Depending on the employed phosphine ligand, either reductive elimination takes place to form azobenzene 3 or denitrogenation and subsequent reductive elimination generates biaryl product 4.

## Conclusions

The present investigation provides detailed insight into the mechanism of the palladium-catalyzed cross-coupling of silicon-masked diazenyl anions and aryl bromides. The DFT calculations revealed a two-step transmetalation with an unusual barrierless carbonate-facilitated desilylation step, delineating the crucial role of silaphilic carbonate base in this transformation. Furthermore, the key role of bidentate, hemilabile diphosphines for controlling the reactivity as well as selectivity of the reaction was rationalized.

## Author contributions

M. O. conceived the work. T. R. led the project, performed the DFT calculations, and analyzed the data. W. O. conducted the experiments. All authors discussed the results. The manuscript was written through contributions of all authors.

## Conflicts of interest

There are no conflicts to declare.

## Supplementary Material

SC-OLF-D6SC04320H-s001

## Data Availability

The data supporting this article have been included as part of the supplementary information (SI). Supplementary information: Experimental details and procedures, analytical data, computational details, calculated energies, cartesian coordinates, additional analysis and data See DOI: https://doi.org/10.1039/d6sc04320h.
