# Supplementary material for: Mechanism of the palladium-catalyzed diazenylation of aryl electrophiles: carbonate-facilitated transmetalation and ligand-dependent selectivity
Source: Chem Sci. 2026 Jun 22;17(31):15000–5. doi: 10.1039/d6sc04320h (PMC13306188; doi:10.1039/d6sc04320h)
Supplement: Supplementary file 1 [file SC-017-D6SC04320H-s001.pdf]

Supplementary Information (SI) for Chemical Science.
This journal is © The Royal Society of Chemistry 2026

Supplementary Information

Mechanism of the Palladium-Catalyzed Diazenylation of Aryl Electrophiles: Carbonate-Facilitated Transmetalation and Ligand-Dependent Selectivity

Torben Rogge,* Wolfgang Obermayer and Martin Oestreich*

Institut für Chemie, Technische Universität Berlin, Straße des 17. Juni 115, 10623 Berlin (Germany)

*Torben Rogge: torben.rogge@chem.tu-berlin.de
Martin Oestreich: martin.oestreich@tu-berlin.de

Contents

General Information S2

General Procedure for Palladium-Catalyzed Diazenylations (GP 1) S3

Characterization Data for Products S3

Control Experiment for Stability of Silyldiazene in Solution S5

Computational Details S7

Comparison of Level of Theory S7

Additional Computational Results S10

Distortion/Interaction Analysis S16

Non-Covalent Interaction (NCI) Analysis S17

Influence of Substituents on Silyldiazene **2** S18

Summary of Energies S19

Cartesian Coordinates S25

References S119

NMR Spectra S121

General Information

Unless otherwise stated, all reactions were performed in an argon-filled MB-Labstar glovebox (O_2 < 0.5 ppm, H_2O < 0.5 ppm), or under nitrogen atmosphere using conventional Schlenk techniques. Glassware was dried either overnight in a 120 °C oven or under oil pump vacuum using a Steinel heat gun. Solids were added to reaction mixtures in a nitrogen countercurrent. The addition of liquid reagents and solvents was performed by using disposable plastic syringes with disposable cannulas. For reactions performed at temperatures other than room temperature, the temperature of the surrounding aluminum heating block is given as a reference.

Reagents and Solvents

Toluene was heated over sodium, distilled under nitrogen atmosphere, degassed using three freeze-pump-thaw cycles, and stored in an argon-filled glovebox (O_2 < 0.5 ppm, H_2O < 0.5 ppm) over 4Å molecular sieves for 24 h before use. For extractions, filtrations or flash column chromatography, technical grade solvents were distilled prior to use. Reagents were obtained from commercial suppliers and used as received. Cesium carbonate was dried overnight under high vacuum ($1 \cdot 10^{-2}$ mbar) at 80 °C prior to use. All *N*-aryl-*N′*-trimethylsilyldiazenes were synthesized according to literature procedures.[1]

Chromatography

Thin-layer chromatography was performed on Macherey-Nagel Alugram®Xtra SIL G/UV254 aluminum-backed TLC plates pre-coated with silica gel 60 with a layer thickness of 200 µm. Flash column chromatography was performed according to a method reported by Still and coworkers[2] on silica gel Davisil LC60A (grain size: 40–63 µm, pore size: 60 Å, 230–400 mesh ASTM) from Grace GmbH.

Nuclear Magnetic Resonance (NMR) Spectroscopy

^{1}H and ^{13}C NMR spectra were recorded on Bruker AV 400 or AV 500 instruments using $CDCl_3$ as the deuterated solvent. The ^{1}H and ^{13}C chemical shifts are reported in parts per million (ppm) and are referenced to the residual protic solvent signal or to the ^{13}C signal of the deuterated solvent ($CHCl_3$: δ/ppm = 7.26, $^{13}CDCl_3$: δ/ppm = 77.16). The data is reported as follows: Chemical shift, multiplicity (s = singlet, d = doublet, t = triplet, m = multiplet, or combinations thereof), coupling constants (Hz), and integration. The specified multiplicities are

phenomenological and do not necessarily correspond to the theoretically expected multiplicities.

Gas-Chromatography

Gas-Chromatography (GC) analyses were performed on an Agilent Technologies GC 8860 gas chromatograph equipped with an Agilent Technologies J&W HP-5 capillary column (length: 30 m × inner diameter: 0.32 mm, film thickness: 0.25 µm). Measurements were performed using the following protocol: Carrier gas: N_2; injector temperature: 250 °C, detector temperature: 275 °C; flow rate isobaric 11 psi; temperature program: start temperature 40 °C, heating rate 10 °C/min, final temperature: 280 °C for 10 min.

General Procedure for Palladium-Catalyzed Diazenylations (GP 1)

A 1.5-mL screw-capped GLC-vial equipped with a magnetic stirring bar is charged with (dppf)$PdCl_2$ (2.9 mg, 4.0 µmol, 2.0 mol%) and cesium carbonate (84.7 mg, 0.260 mmol, 1.30 equiv.). A solution of bromobenzene (**1b**, 31.4 mg, 0.200 mmol, 1.00 equiv.) and the corresponding diazene **2** (1.20 equiv.) in toluene (0.4 mL) are added, and the reaction mixture is stirred at 60 °C for 3.5 d. A saturated aqueous solution of NH_4Cl (10 mL) and *tert*-butyl methyl ether (10 mL) are added, the phases are separated, and the aqueous phase is extracted with *tert*-butyl methyl ether (3 × 10 mL). The combined organic phases are filtered through a pad of $MgSO_4$, and the solvent is removed under reduced pressure. Purification by flash column chromatography on silica gel affords the non-symmetric azobenzenes **3**.

Characterization Data for Products

(*E*)-1-(4-Fluorophenyl)-2-phenyldiazene (3bc)

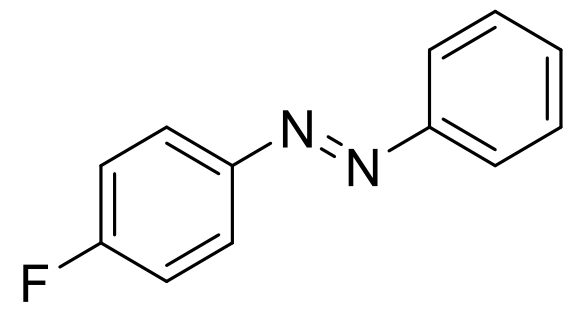

3bc
$C_{12}H_9FN_2$
M = 200.22 g/mol

Prepared according to **GP 1** from bromobenzene (**1b**) and (*E*)-1-(4-fluorophenyl)-2-(trimethylsilyl)diazene (**2c**, 47.1 mg, 0.240 mmol, 1.20 equiv.). Purification by flash column chromatography on silica gel using *n*-pentane : Et_2O = 99.5 : 0.5 as the eluent afforded (*E*)-1-(4-fluorophenyl)-2-phenyldiazene (**3bc**, 28.7 mg, 0.143 mmol, 72%) as a light orange solid.

^{1}H NMR (500 MHz, $CDCl_3$): δ/ppm = 7.99–7.93 (m, 2H), 7.91 (d, *J* = 7.39 Hz, 2H), 7.52 (t, *J* = 7.0 Hz, 2H), 7.48 (t, *J* = 7.0 Hz, 1H), 7.20 (t, *J* = 8.58 Hz, 2H). **^{13}C{^{1}H} NMR** (126 MHz, $CDCl_3$): δ/ppm = 164.5 (d, *J* = 250.6 Hz), 152.6, 149.3 (d, *J* = 3.2 Hz), 131.2, 129.3, 125.0 (d, *J* = 8.8 Hz), 123.0, 116.2 (d, *J* = 22.8 Hz). **^{19}F NMR** (471 MHz, $CDCl_3$): –109.37–(–109.51) (m, 1F). **HRMS** (APCI): calculated for $C_{12}H_{10}FN_2^+$ [$(M+H)^+$]: 201.0823; found: 201.0822.

The NMR data are in accordance with the literature.[3]

(*E*)-1-(4-Methoxyphenyl)-2-phenyldiazene (3bd)

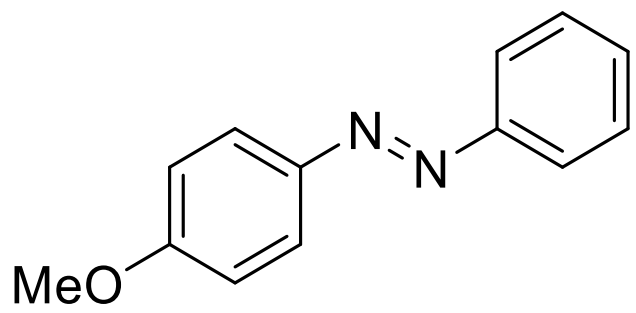

3bd
$C_{13}H_{12}N_2O$
M = 212.25 g/mol

Prepared according to **GP 1** from bromobenzene (**1b**) and (*E*)-1-(4-methoxyphenyl)-2-(trimethylsilyl)diazene (**2d**, 50.0 mg, 0.240 mmol, 1.20 equiv.). Purification by flash column chromatography on silica gel using *n*-pentane : Et_2O = 98 : 2 as the eluent afforded (*E*)-1-(4-Methoxyphenyl)-2-phenyldiazene (**3bd**, 32.8 mg, 0.155 mmol, 78%) as a light orange solid. **^{1}H NMR** (500 MHz, $CDCl_3$): δ/ppm = 7.94 (d, *J* = 9.0 Hz, 2H), 7.89 (d, *J* = 7.7 Hz, 2H), 7.51 (t, *J* = 7.3 Hz, 2H), 7.44 (t, *J* = 7.1 Hz, 1H), 7.02 (d, *J* = 9.0 Hz, 2H), 3.90 (s, 3H). ^{13}C{^{1}H} NMR (101 MHz, $CDCl_3$): δ/ppm = 162.2, 152.9, 147.2, 130.5, 129.2, 124.9, 122.7, 114.4, 55.7. HRMS (APCI): calculated for $C_{13}H_{13}N_2O^+$ [$(M+H)^+$]: 213.1022; found: 213.1021.

The NMR data are in accordance with the literature.[3]

Control Experiment for Stability of Silyldiazene in Solution

To determine whether silyldiazenes react with Cs_2CO_3, in the absence of catalyst and substrate, we performed a control experiment, in which model diazene **2a** was reacted with cesium carbonate in toluene-d_8 at 60 °C for 24 h and analyzed the reaction mixture by ^{1}H NMR. To rule out thermal decomposition of the starting material in the absence of base as an alternative decomposition pathway, we also performed the experiment without Cs_2CO_3. No significant decomposition of the silyldiazene was observed in both cases. The experimental procedure and the corresponding NMR-spectra are given below.

<u>Procedure:</u>

A 1.5-mL screw-capped GLC-vial equipped with a magnetic stirring bar was charged with 1,3,5-trimethoxybenzene (16.8 mg, 0.10 mmol, 1.00 equiv.) and with or without cesium carbonate (35.8 mg, 0.11 mmol, 1.10 equiv.). A solution of *N*-(*p*-tolyl)-*N*′-trimethylsilyldiazene (**2a**, 19.2 mg, 0.10 mmol, 1.00 equiv.) in toluene-d_8 (0.2 mL) was added and the reaction mixture was stirred at 60 °C for 24 h. The reaction mixture was diluted with toluene-d_8 (0.4 mL) and analyzed by ^{1}H NMR spectroscopy.

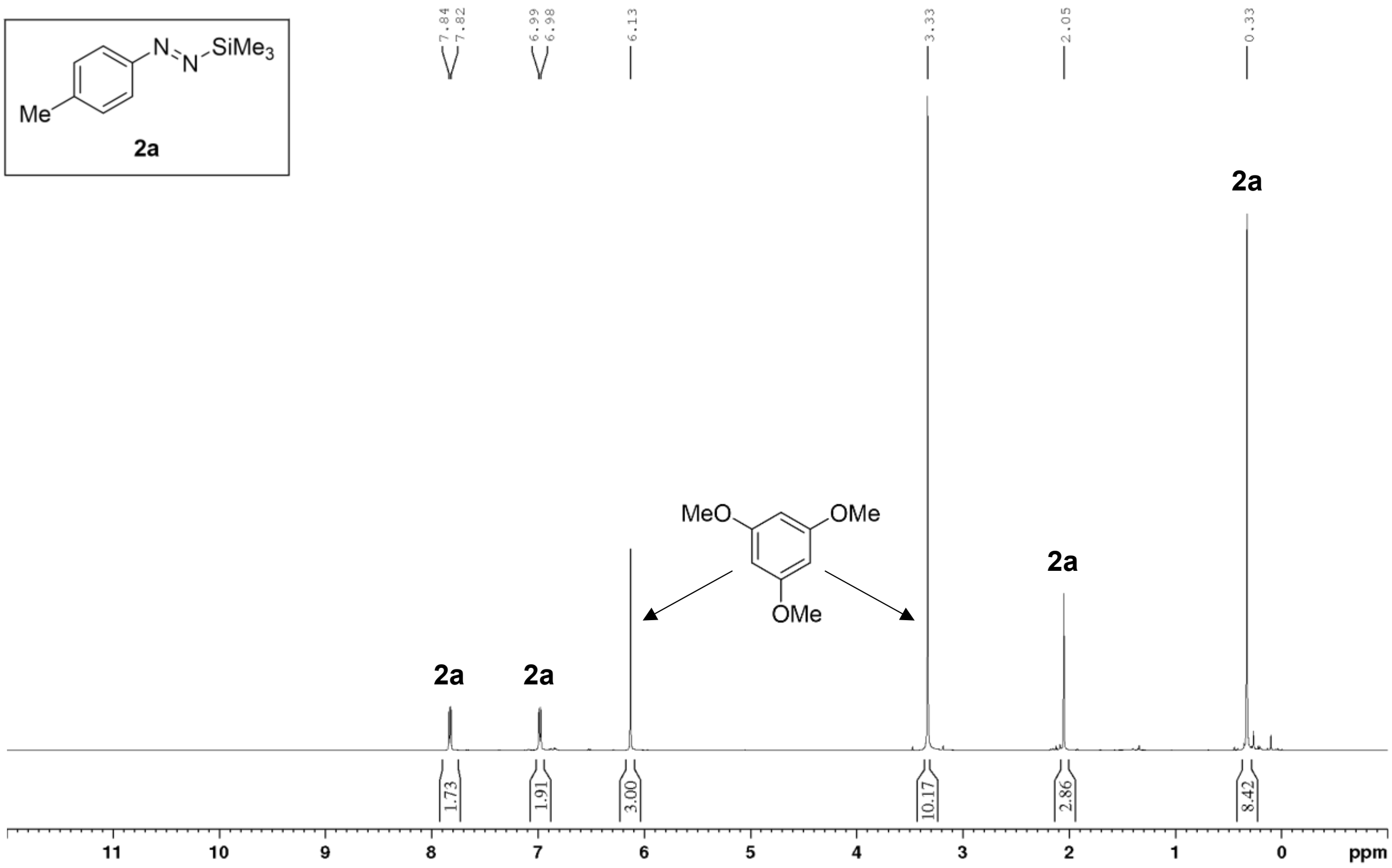

Figure S1. ^{1}H NMR (500 MHz, toluene-d_8) spectrum obtained for the reaction of *N*-(*p*-tolyl)-*N*′-trimethylsilyldiazene with Cs_2CO_3 (1.1 equiv.).

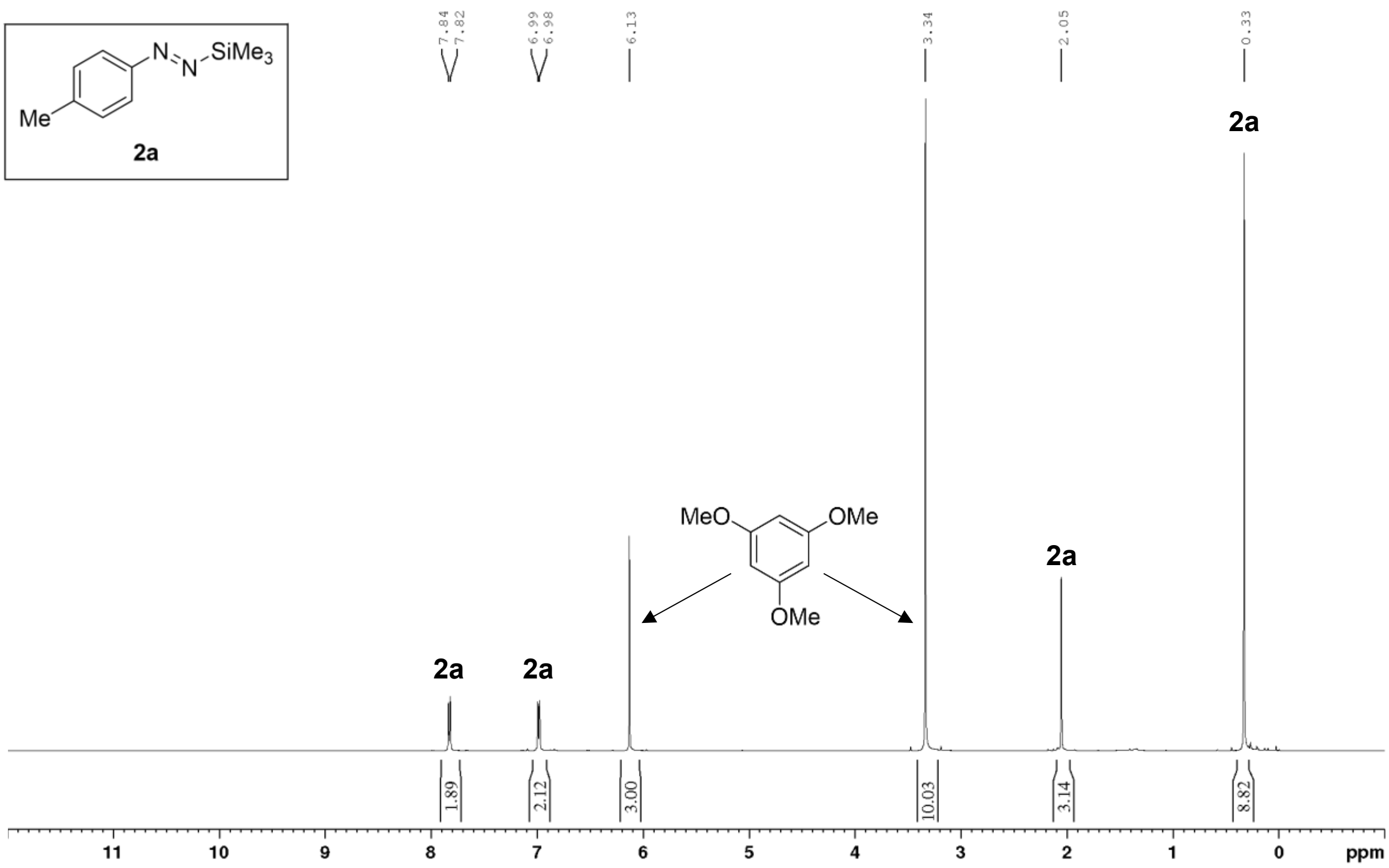

Figure S2. ^{1}H NMR spectrum (500 MHz, toluene-d_8) obtained for the reaction of *N*-(*p*-tolyl)-*N′*-trimethylsilyldiazene without Cs_2CO_3.

Computational Details

All density functional theory (DFT) calculations were performed with the Gaussian 16, Rev. A.03, program package.[4] Geometry optimizations were performed at the TPSS level of theory[5] including Grimme's D3 dispersion correction with Becke-Johnson damping function.[6] Palladium, iron, bromine and cesium were described with the def2-SVP basis set[7] and Stuttgart/Dresden (SDD) effective core potentials,[8] while all other atoms were described with the def2-SVP basis set. Analytical frequency calculations were performed at the same level of theory to characterize all stationary points as intermediates (no imaginary frequencies) or transition states (exactly one imaginary frequency). Intrinsic Reaction Coordinate (IRC) calculations were performed at the same level of theory to confirm the intermediates linked by each transition state. The electronic energy was refined through single point calculations at the ωB97X-D level of theory,[9] employing a def2-TZVP basis set.[7] In all single point calculations, solvent effects were taken into consideration through the use of the SMD implicit solvent model for toluene (ε = 2.3741) as implemented in Gaussian.[10] For silyldiazenes **2**, azobenzenes **3**, biaryls **4**, Br–$SiMe_3$, $CsCO_3^-$ and intermediates of dppb-ligated complexes, conformational searches were performed using Conformer-Rotamer Ensemble Sampling Tool (CREST), version 2.12, followed by subsequent DFT geometry optimizations.[11] GoodVibes[12] (v. 3.0.1) with quasi-harmonic entropy[13] and enthalpy treatment[14] (frequency cut-off value: 100 cm^{-1}) was used to obtain corrected Gibbs free energies and enthalpies at 333 K and 1.0 mol L^{-1}. 3D-visualizations of optimized structures were rendered with CYLview20.[15] Natural Bond Orbital (NBO) analysis was performed with the NBO 6.0 program at the TPPS-D3(BJ)/def2-SVP level of theory.[16] Figures of overlayed structures (Figure S4) and RMSD calculations were performed with PyMOL (version 3.2.0a), using the *align* command.[17] Non-covalent interaction (NCI) analysis was performed with Multiwfn (version 2026.6.2)[18] using densities calculated at the ωB97X-D/def2-TZVP+SMD(toluene) level of theory and visualized with VMD (version 1.9.3).[19]

Comparison of Level of Theory

For silyldiazene **2b**, the geometry calculated at the TPSS-D3(BJ)/def2-SVP level of theory was found to be very similar to the calculated geometry at the TPSS-D3(BJ)/def2-SVP+SMD(toluene) level of theory as well as the XRD structure of a strongly related 4-cyano

substituted silyldiazene (Figure S3). In addition, for the representative complexes **B** and **TS1**, the inclusion of the SMD solvation model in the geometry optimizations did not result in significantly altered geometries with RMSD = 0.08 and 0.24 for **B** and **TS1**, respectively (Figure S4).

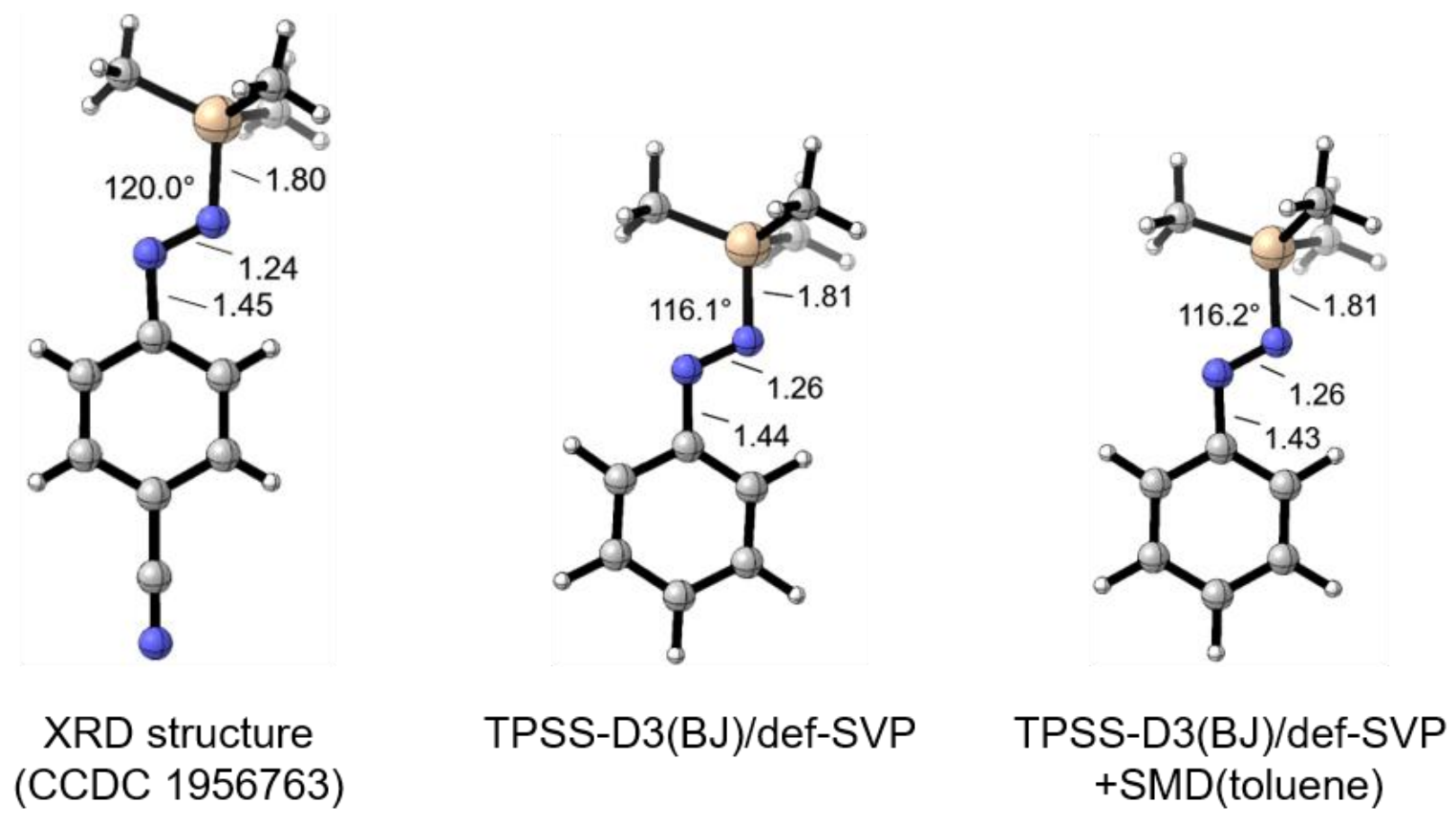

Figure S3. Comparison of the calculated geometries at the TPSS-D3(BJ)/def2-SVP (middle) and TPSS-D3(BJ)/def2-SVP+SMD(toluene) (right) level of theory with the previously reported XRD structure of 4-cyano-substituted silyldiazene (CCDC 1956763, left).[1] Distances are given in Å.

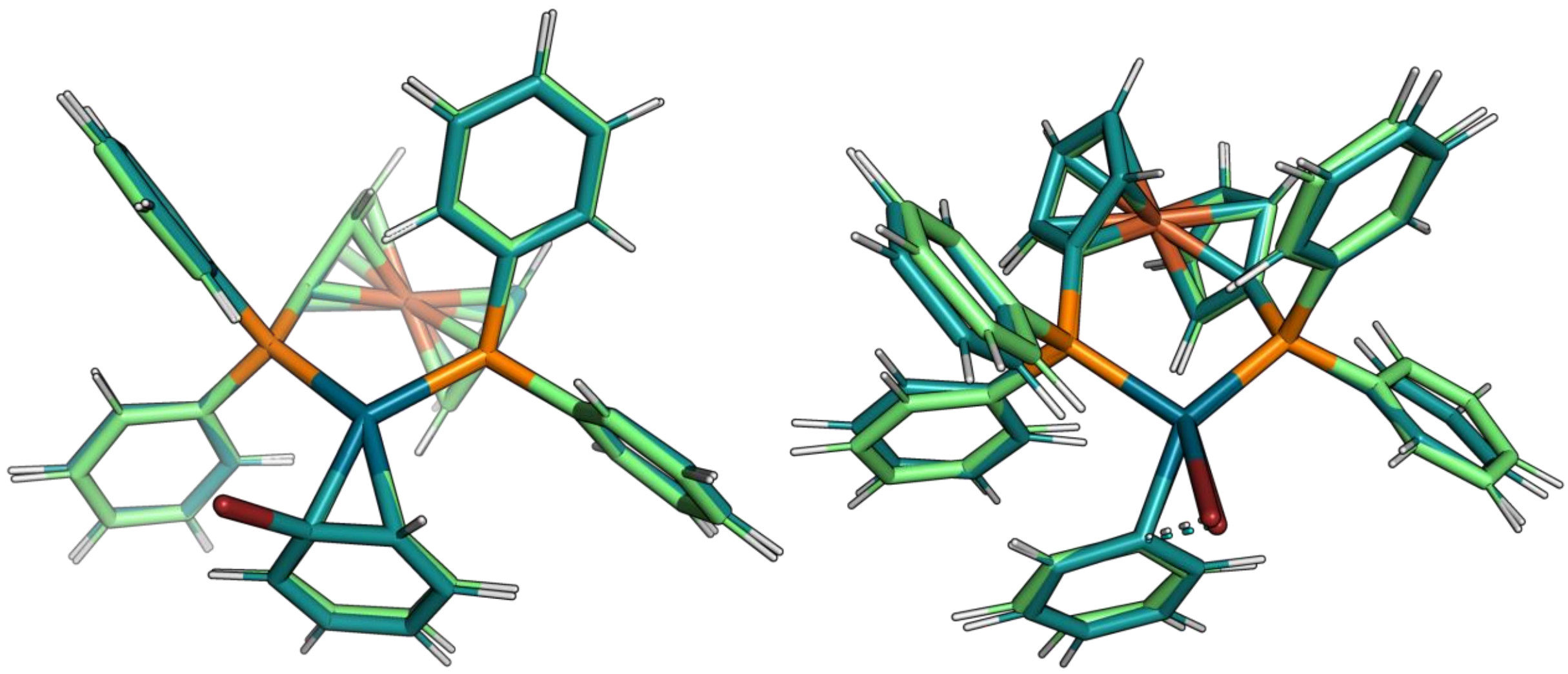

Figure S4. Overlay of calculated geometries at the TPSS-D3(BJ)/def2-SVP (green colored carbon atoms) and TPSS-D3(BJ)/def2-SVP+SMD(toluene) (teal colored carbon atoms) level of theory for intermediate **B** (left) and transition state **TS1** (right).

A comparison of Gibbs free energies for the oxidative addition step at different levels of theory, including single point calculations with the large def2-QZVP basis set, revealed only marginal differences in energies (Table S1). Since calculations with the def2-QZVP basis set are significantly more demanding with respect to CPU time, the def2-TZVP basis set was employed in all single point calculations unless stated otherwise.

Table S1. Calculated Gibbs free energies (in kcal mol^{-1}) relative to A at different levels of theory.

Level of theory	A	B	TS1	C
ωB97X-D/def2-TZVP+SMD(toluene)//TPSS-D3(BJ)/def2-SVP	0.0	1.6	11.6	–15.4
ωB97X-D/def2-TZVP+SMD(toluene)//TPSS-D3(BJ)/def2-SVP+**SMD(toluene)**	0.0	1.8	11.8	--[a]
ωB97X-D/**def2-QZVP**+SMD(toluene)//TPSS-D3(BJ)/def2-SVP	0.0	1.7	12.6	–17.0

[a] Geometry could not be converged.

Additional Computational Results

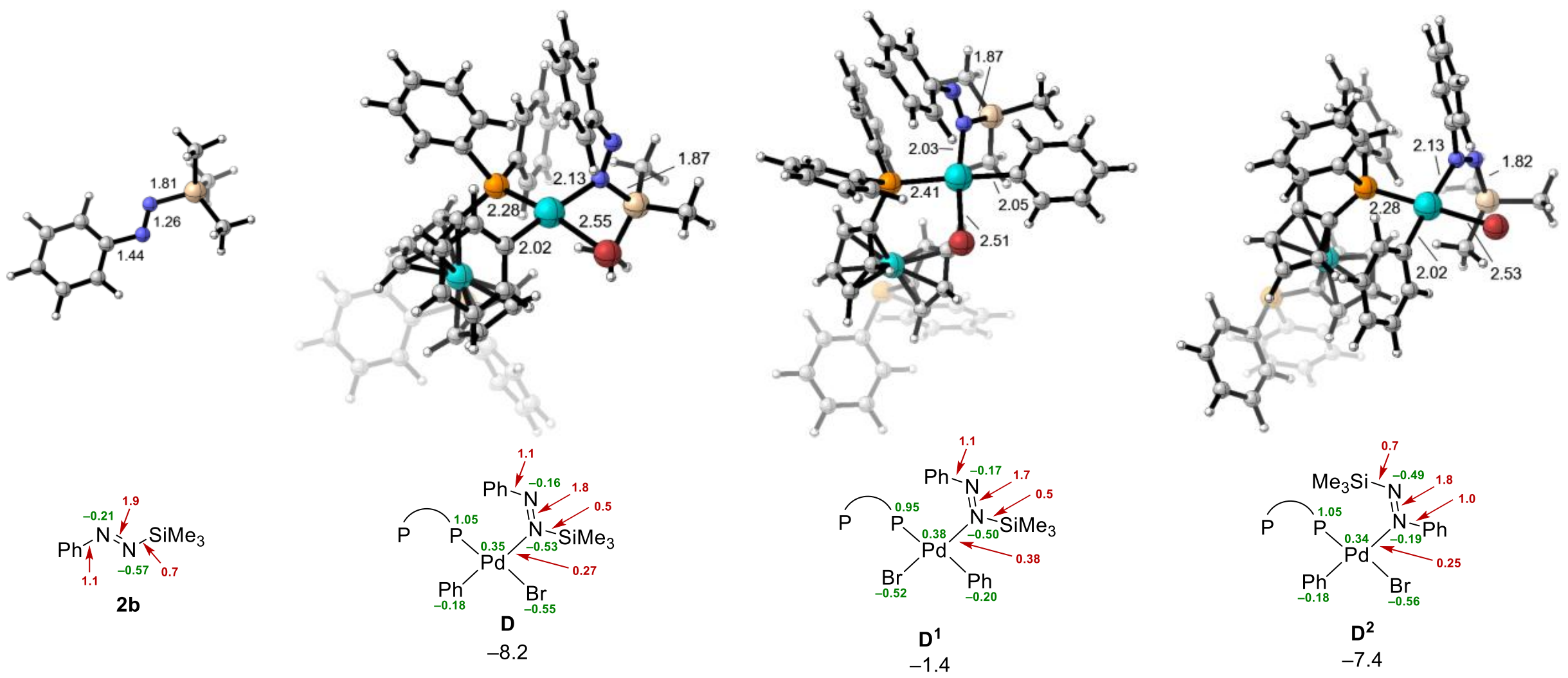

Figure S5. Coordination of **2b**. Distances are given in Å. Values in green color correspond to natural charges, values in red to Wiberg bond indices. Energies are Gibbs free energies (in kcal mol^{-1}) relative to **A**.

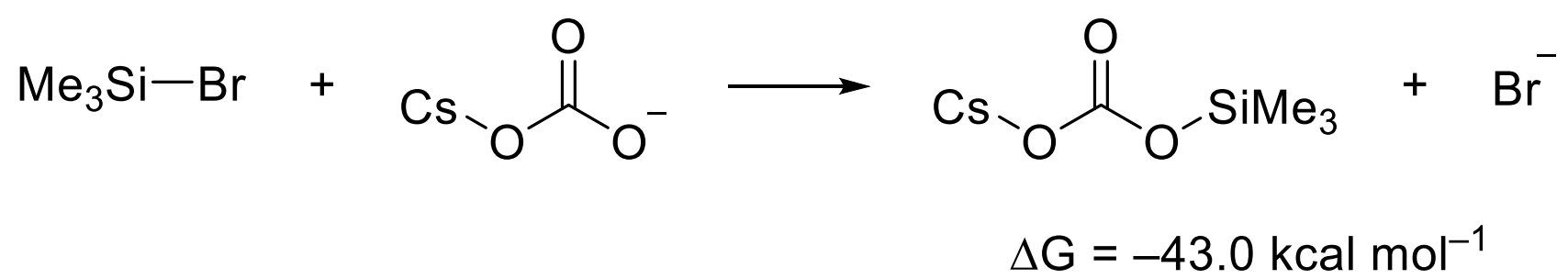

Scheme S1. Calculated Gibbs free energy for the formation of $CsCO_3$–$SiMe_3$.

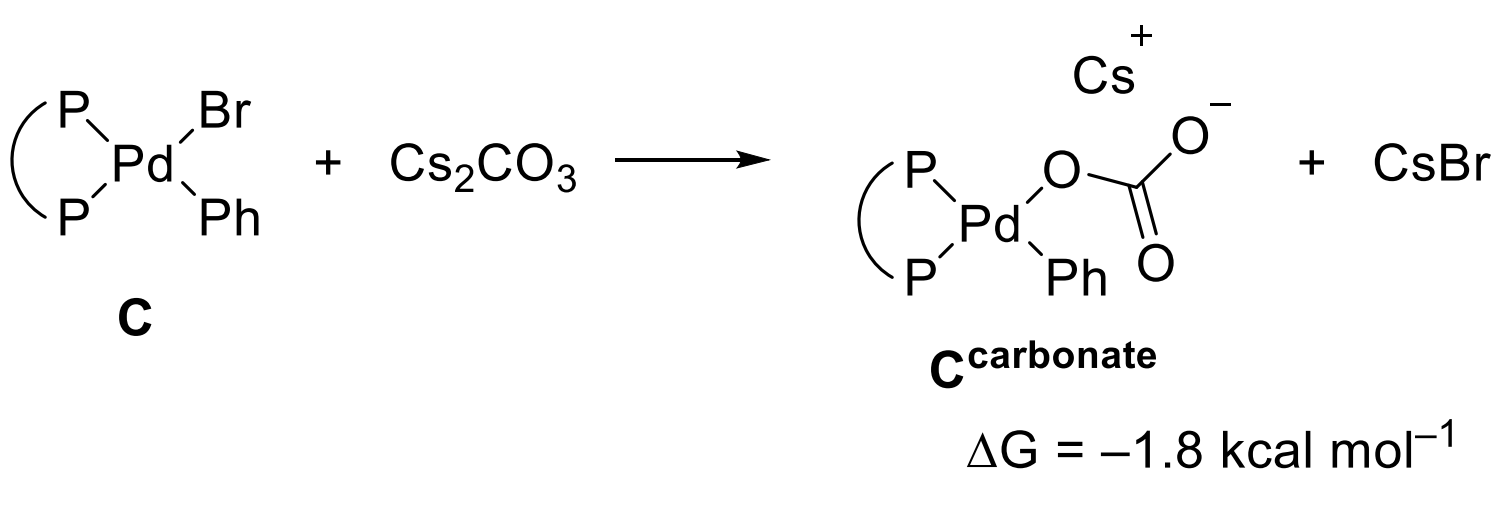

Scheme S2. Calculated Gibbs free energy for ligand exchange on intermediate **C**.

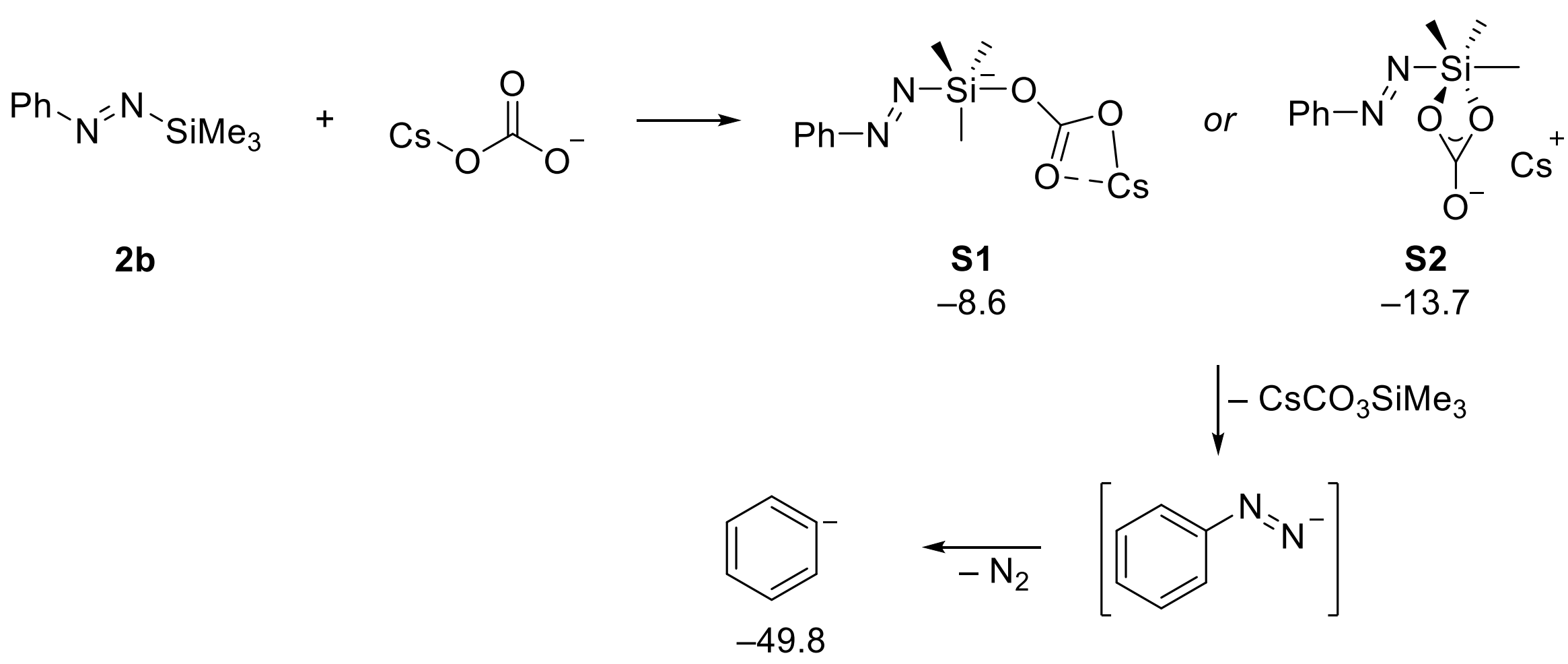

Scheme S3. Calculated Gibbs free energy (in kcal mol^{-1}) for the activation of silyl diazene **2b** in the absence of palladium complex.

While the dissociation of cesium carbonate is calculated to be highly endergonic, these unfavorable thermodynamics can be attributed to the separation of charges (i.e. cesium cations and carbonate anions). Within the framework of the widely employed SMD solvation model, stabilizing effects, such as formation of solvent-separated or solvent-shared ion pairs, can not be described. For the Gibbs free energy profiles, formation of $CsCO_3^-$ is assumed, since formation of highly negatively charged CO_3^{2-} is less likely to occur in toluene as apolar reaction medium.

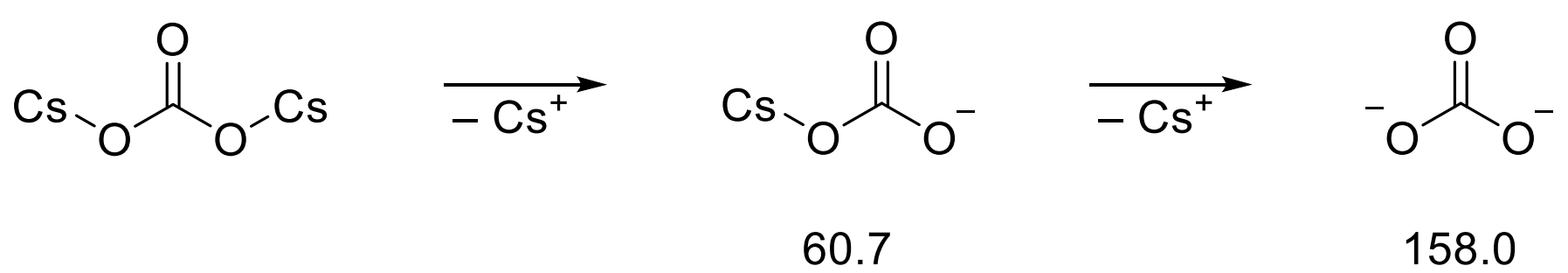

Scheme S4. Calculated Gibbs free energy (in kcal mol^{-1}) for the dissociation of Cs_2CO_3.

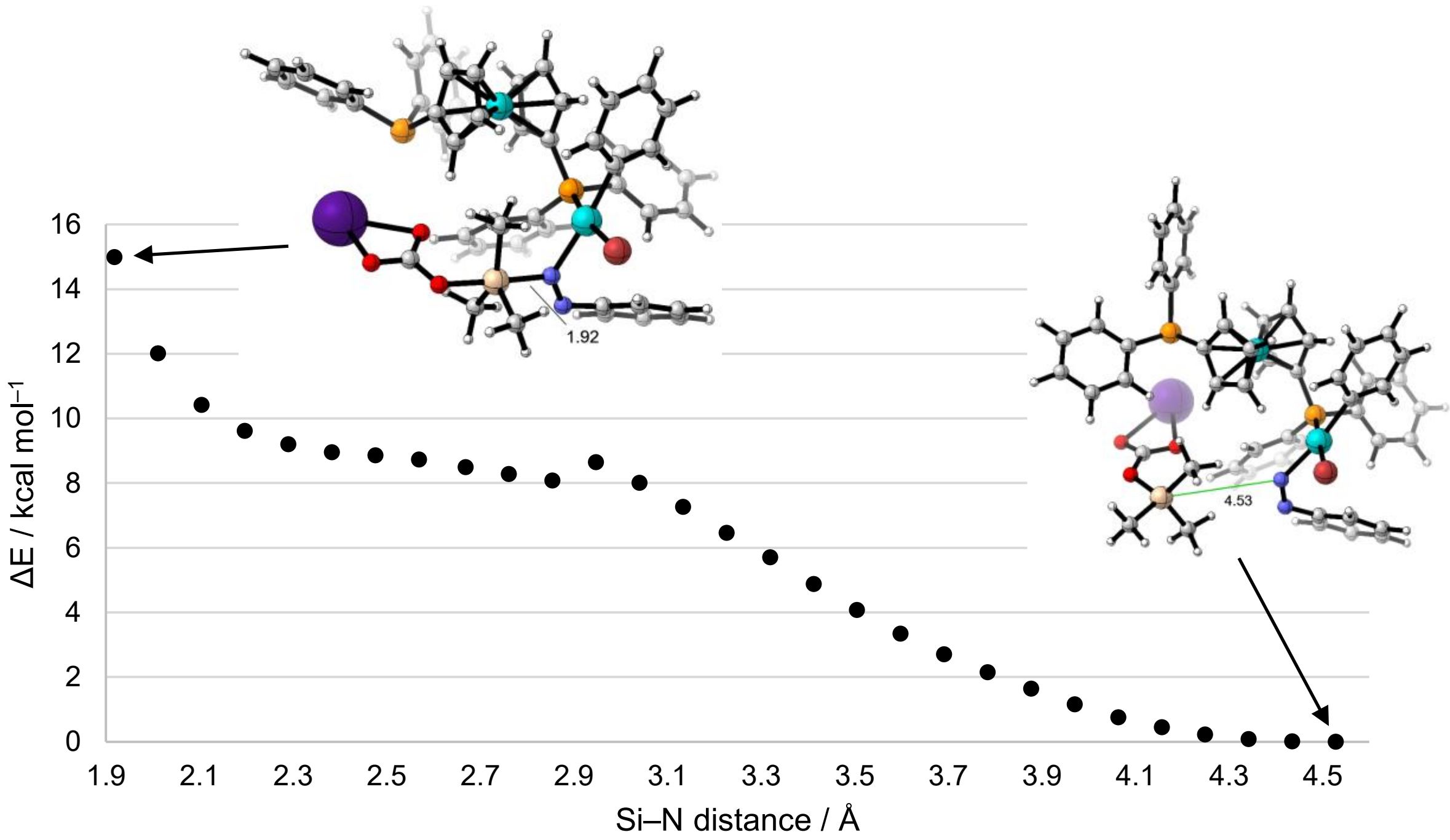

Figure S6. Relaxed potential energy surface scan (in kcal mol^{-1}) for the carbonate-assisted transmetalation. Distances are given in Å.

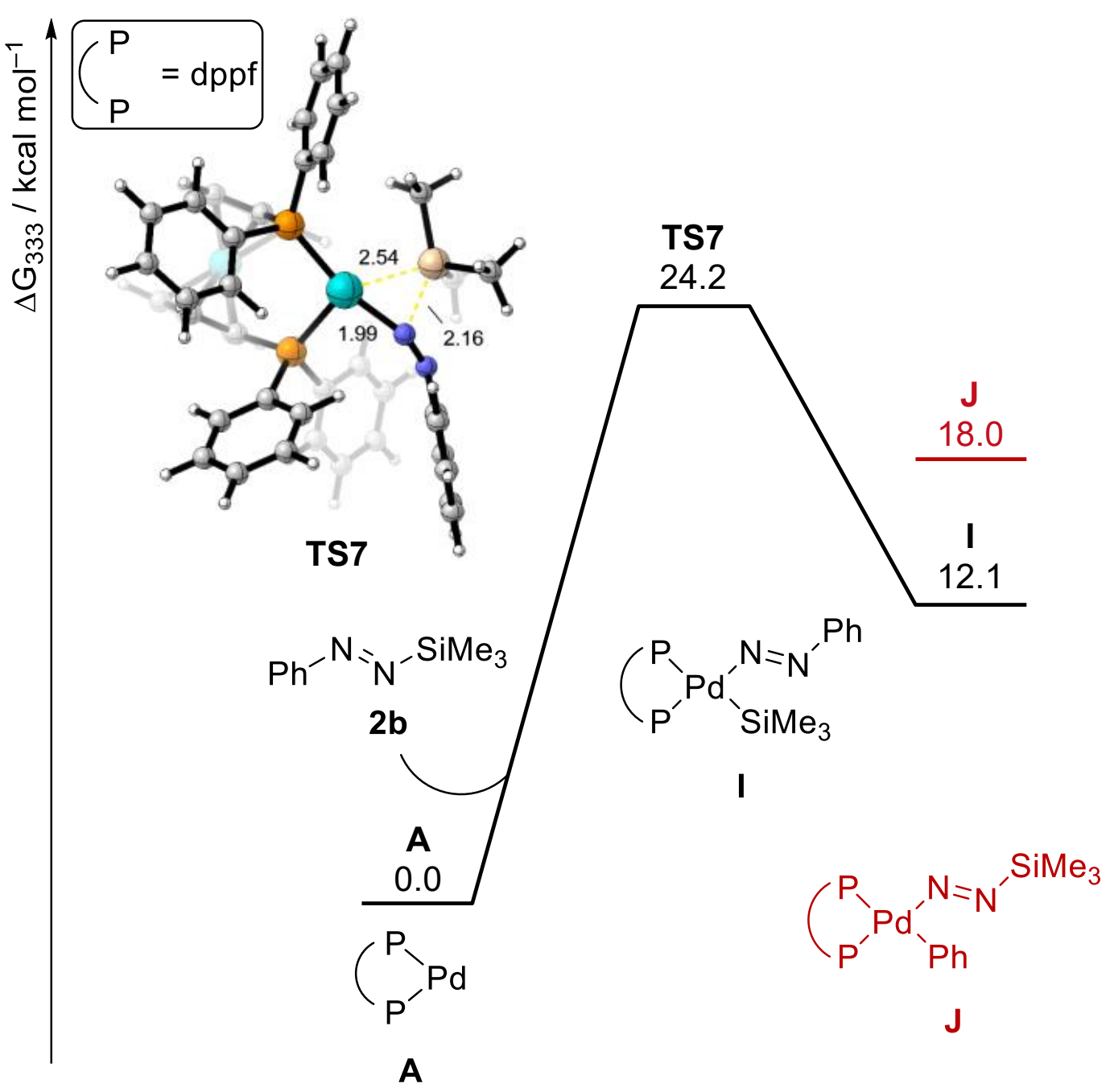

Figure S7. Calculated Gibbs free energy diagram (in kcal mol^{-1}) for the oxidative addition of silyl diazene **2b**. Distances are given in Å.

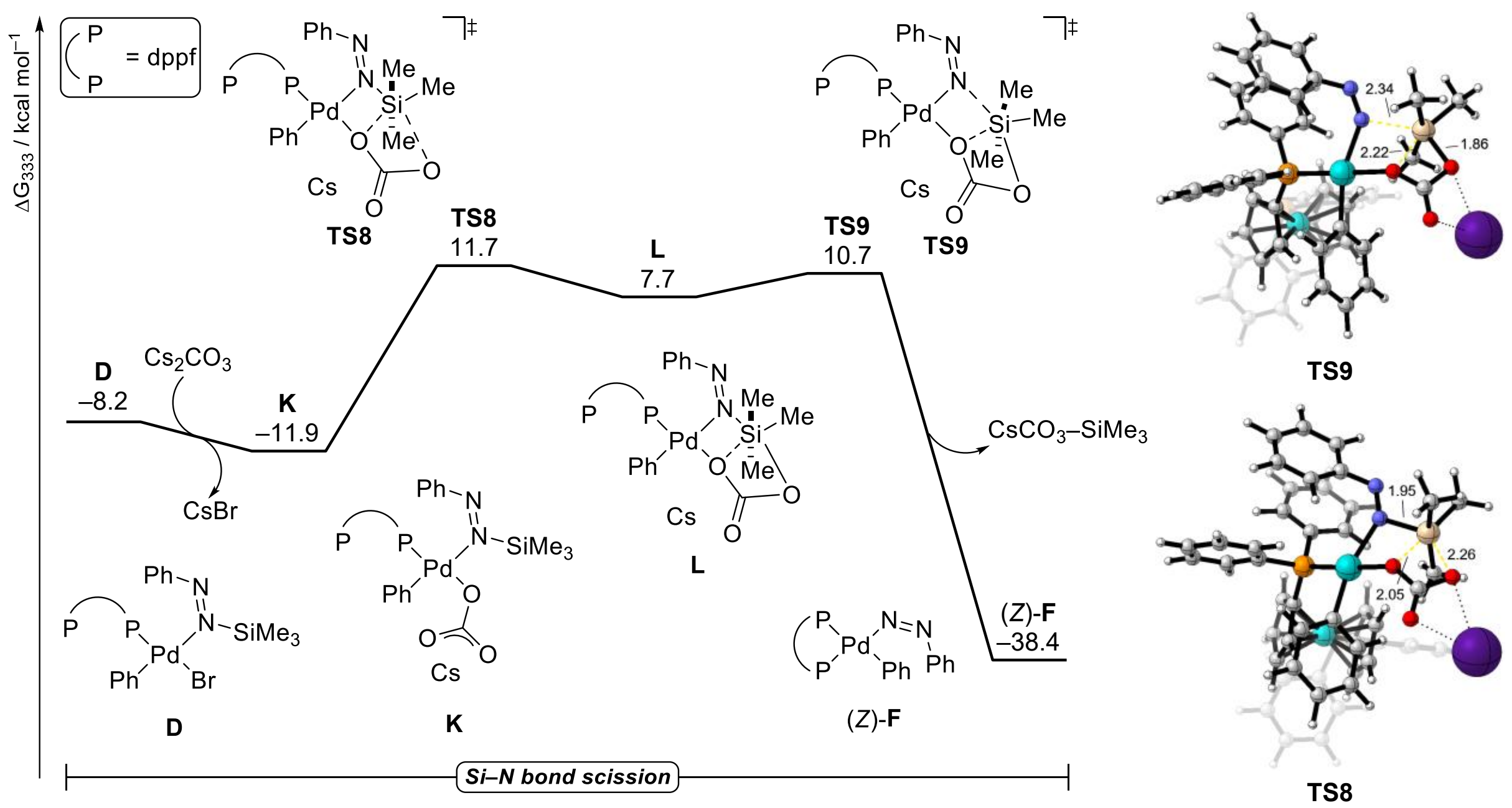

Figure S8. Calculated Gibbs free energy diagram (in kcal mol^{-1}) relative to **A** for the inner-sphere desilylation by carbonate. Distances are given in Å.

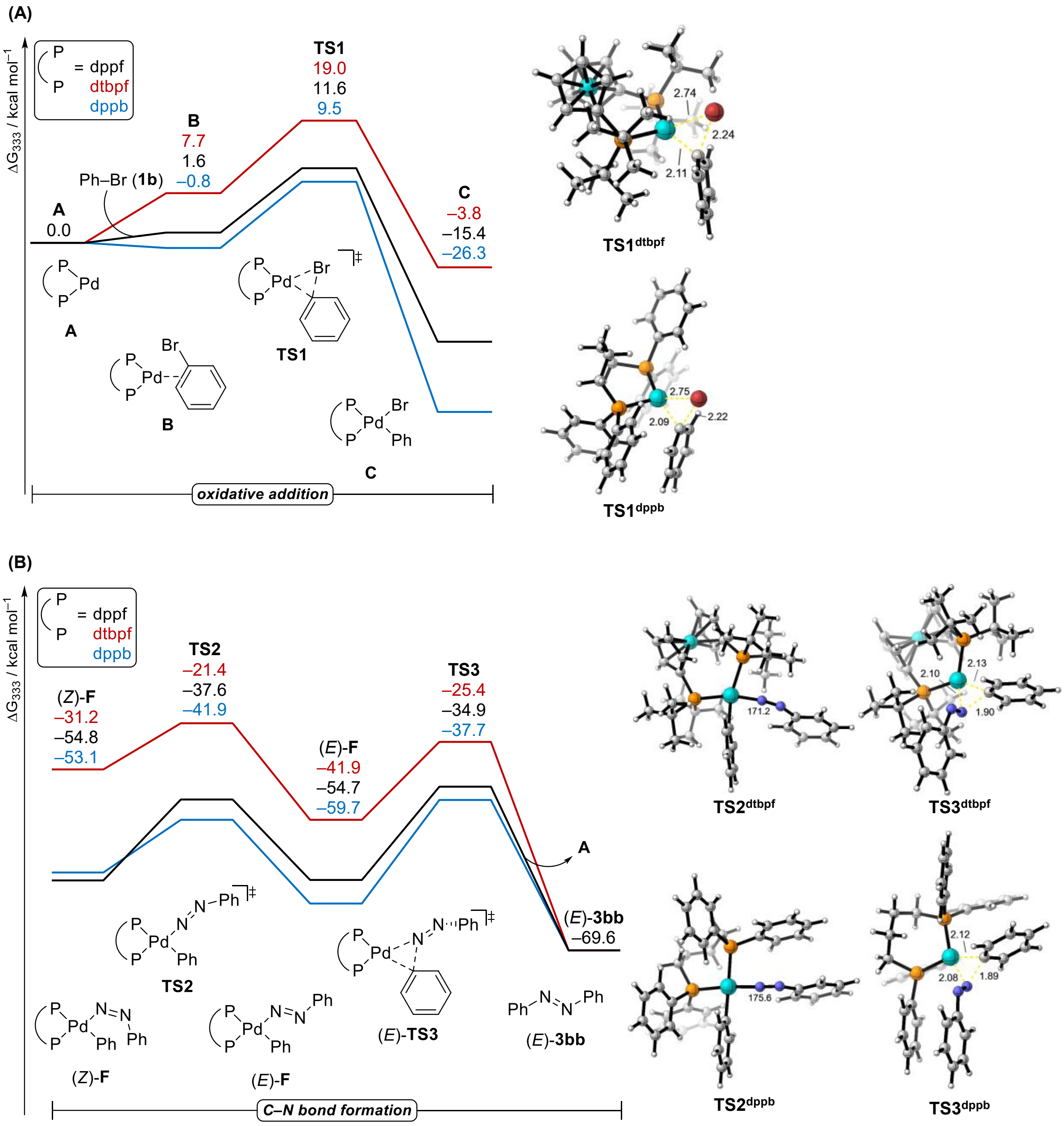

Figure S9. Calculated Gibbs free energy diagram (in kcal mol^{-1}) relative to **A** for dppf (black line), dtbpf (red) and dppb (blue) ligated complexes for (A) oxidative addition of **1b**, and (B) formation of (*E*)**-3bb**. Distances are given in Å and angles in °.

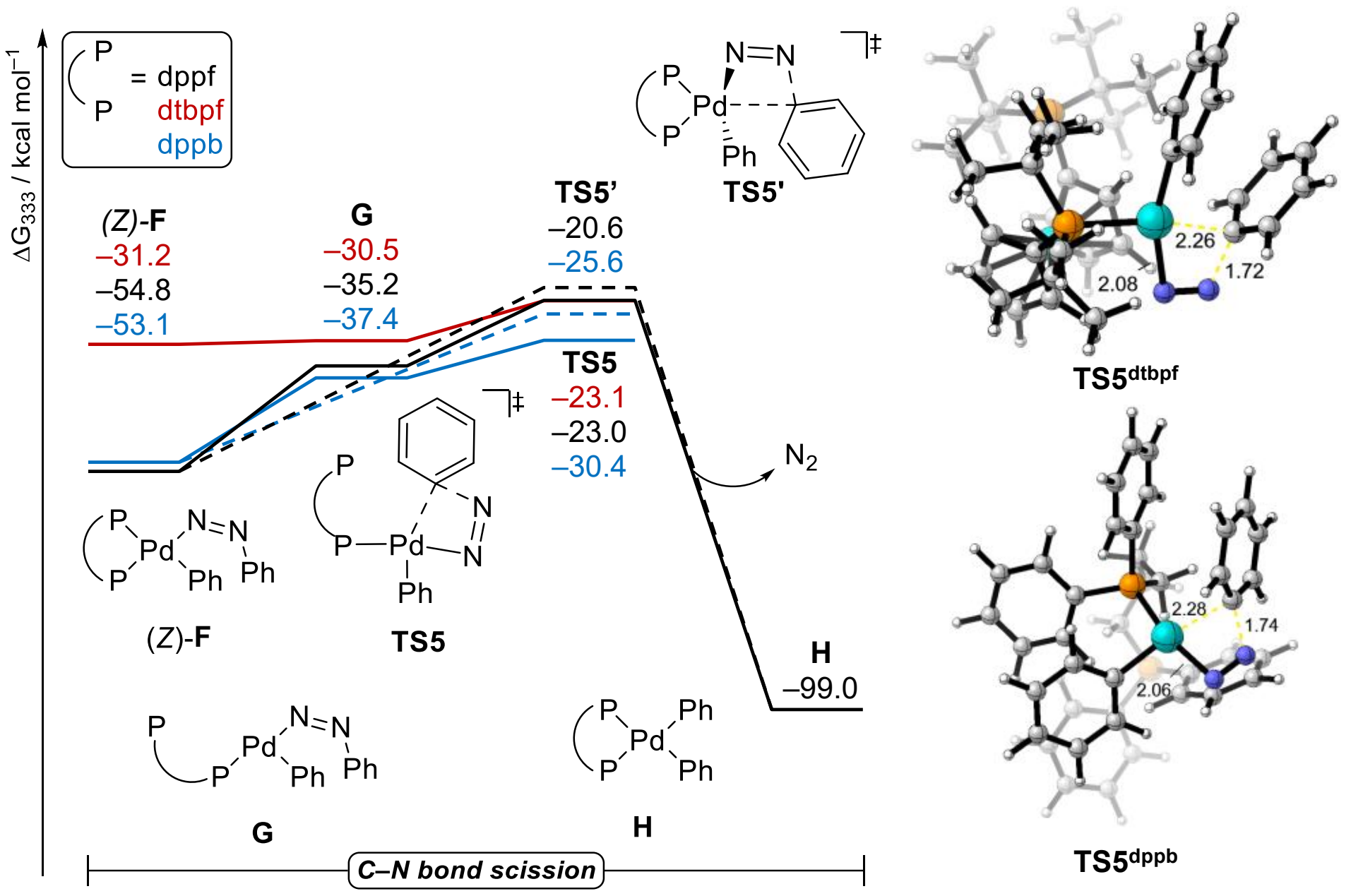

Figure S10. Calculated Gibbs free energy diagram (in kcal mol^{-1}) relative to **A** for dppf (black line), dtbpf (red) and dppb (blue) ligated complexes for denitrogenation. Denitrogenation via **TS5'** shown as dashed lines. Distances are given in Å. For dtbpf as ligand, **TS5'** was not stable due to unfavorable steric interactions.

Distortion/Interaction Analysis

A distortion/interaction analysis for transition states **TS1**, (*E*)-**TS3** and **TS5** was performed by separating the complex into two fragments as shown below. The energies of the distorted and non-distorted fragments were calculated at the ωB97X-D/def2-TZVP+SMD(toluene) level of theory and are calculated with respect to the most stable prior intermediate, i.e. intermediate **A** for **TS1,** intermediate (*E*)-**F** for (*E*)-**TS3** and intermediate (*Z*)-**F** for **TS5**.

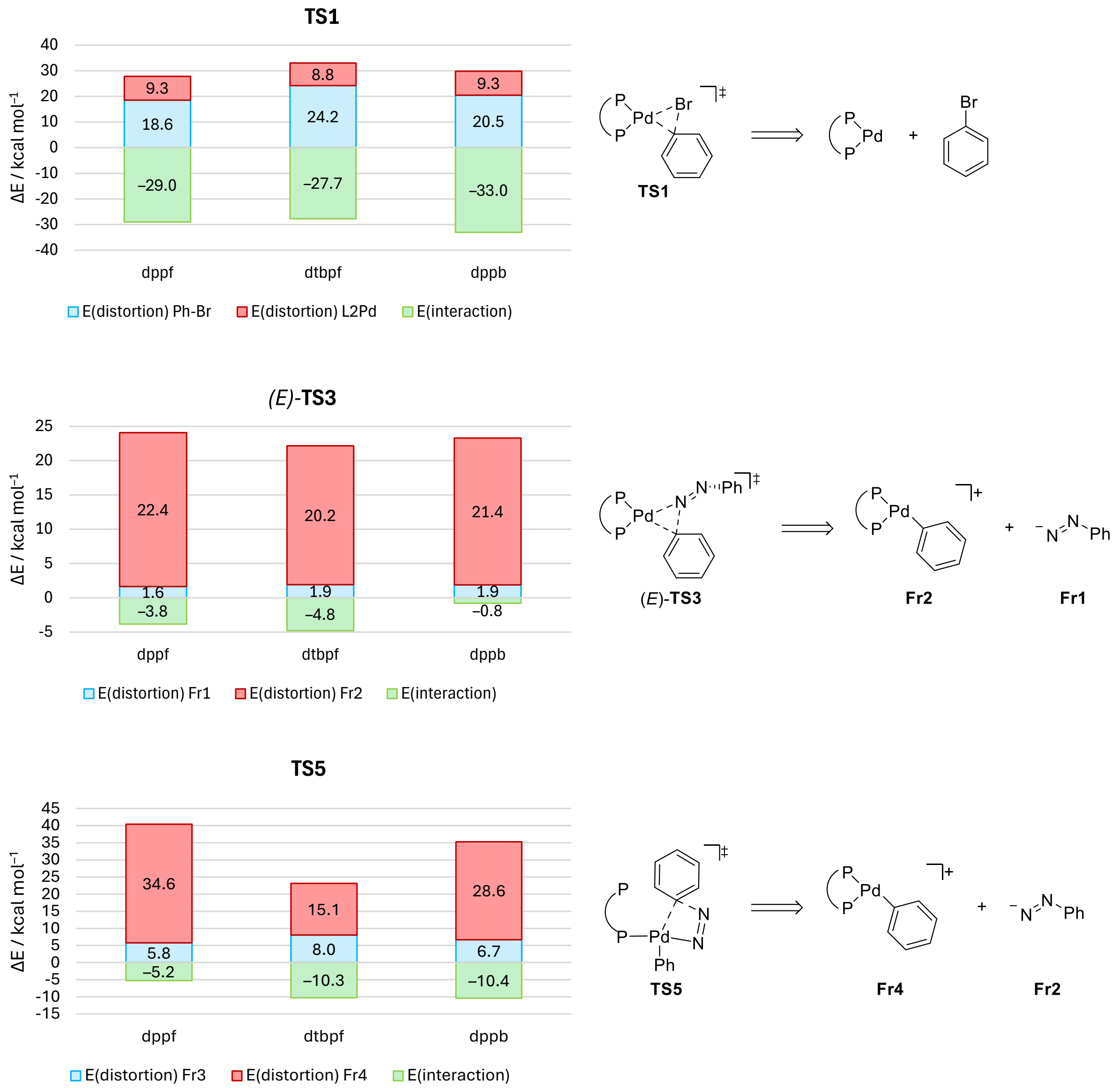

Figure S11. Distortion/interaction analysis for **TS1** (top), (*E*)-**TS3** (middle) and **TS5** (bottom). Energies are electronic energies at the ωB97X-D/def2-TZVP+SMD(toluene) level of theory.

Non-Covalent Interaction (NCI) Analysis

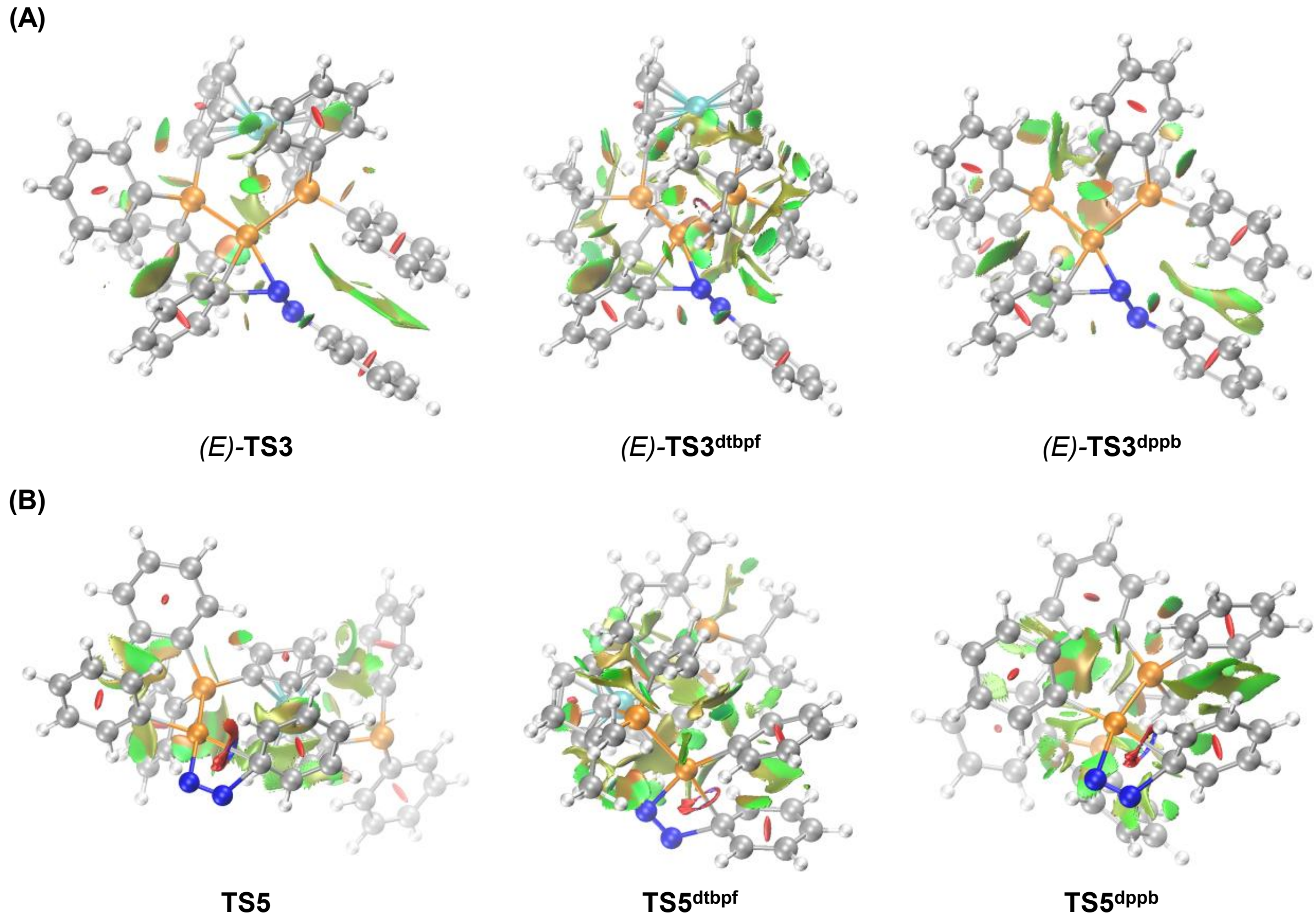

Figure S12. Non-covalent interaction (NCI) analysis of (A) reductive elimination transition state (*E*)-**TS5**, and (B) C–N bond cleavage transition state **TS5**. In the plotted surface (isovalue = 0.4), red corresponds to strong repulsive, green/green-brown to weak attractive and blue to strong attractive interactions.

Influence of Substituents on Silyldiazene 2

Table S2. Transformation of substituted silyldiazenes.

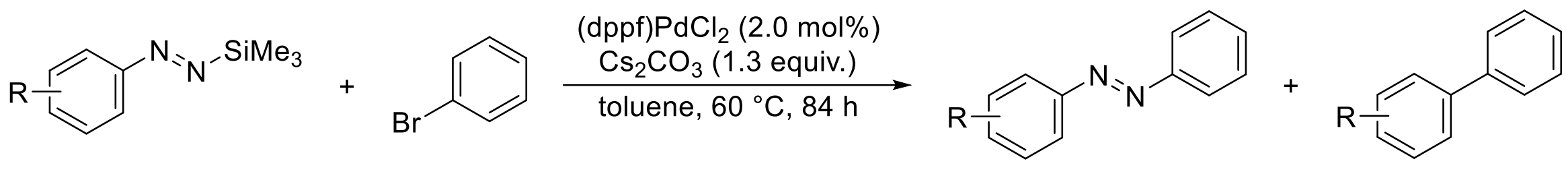

Entry[a]	R	$\Delta G_{(E)\text{-}\mathbf{F}}$	$\Delta G_{\mathbf{G}}$	$\Delta G_{(E)\text{-}\mathbf{TS3}}$	$\Delta G_{\mathbf{TS5}}$	Exp. Results[b]	
						**3** / %	**4** / %
1	H (**2b**)	–54.7	–35.2	–34.9	–23.0	--[c]	--[c]
2	4-F (**2c**)	–54.8	–35.2	–35.2	–22.8	72	n.d.
3	4-OMe (**2d**)	–53.2	–34.5	–33.6	–23.7	78	n.d.
4	4-CF_3 (**2e**)	–54.8	–35.5	–36.4	–23.7	--[c]	--[c]
5	2-F (**2f**)	–54.4	–35.2	–34.9	–26.8	n.d.	n.d.
6	2-OMe (**2g**)	–55.2	–37.4	–36.6	–29.7	--[c,d]	--[c,d]
7	2-CF_3 (**2h**)	–55.4	–35.6	–37.1	–25.6	--[c,d]	--[c,d]

[a] Calculated Gibbs free energies (in kcal mol^{-1}) relative to **A**. [b] Reaction conditions: **1b** (0.10 mmol), **2** (1.2 equiv.), (dppf)$PdCl_2$ (2.0 mol%), Cs_2CO_3 (1.30 equiv.), toluene (0.40 mL) at 60 °C for 3.5 d. Yields were determined by calibrated GLC analysis with tetracosane as internal standard. [c] Experiment not conducted. [d] The synthesis of corresponding diazene **2** is not literature-known. n.d., not detected.

Summary of Energies

Structure	E_SPC (au)	qh-H_SPC (au)	T.qh-S (au)	qh-G(T)_SPC (au)	im. freq. (cm^{-1})
**Figure 1, Figure S3 and S4 and Table S1**					
(dppf)Pd (**A**)	-2246.121667	-2245.575995	0.110250	-2245.686245	--
(dppf)Pd (**A**)[a]	-2246.121564	-2245.575998	0.110541	-2245.686539	--
(dppf)Pd (**A**)[b]	-2246.528621	-2245.982949	0.110250	-2246.093199	--
**B**	-2491.074885	-2490.432508	0.128010	-2490.560518	--
**B**[a]	-2491.074827	-2490.432610	0.127828	-2490.560439	--
**B**[b]	-2491.610697	-2490.968320	0.128010	-2491.096330	--
**TS1**	-2491.058306	-2490.417331	0.127291	-2490.544622	128.38
**TS1**[a]	-2491.058040	-2490.417043	0.127410	-2490.544453	136.91
**TS1**[b]	-2491.592685	-2490.951711	0.127291	-2491.079002	128.38
**C**	-2491.104701	-2490.460636	0.126912	-2490.587548	--
**C**[b]	-2491.643345	-2490.999280	0.126912	-2491.126192	--
**D**	-3241.533399	-3240.664018	0.165351	-3240.829369	--
**E**	-2832.295347	-2831.548722	0.146736	-2831.695458	--
(*Z*)-**F**	-2818.887516	-2818.141007	0.138928	-2818.279935	--
**TS2**	-2818.858718	-2818.113984	0.138505	-2818.252489	389.18
(*E*)-**F**	-2818.886195	-2818.139882	0.139751	-2818.279634	--
(*E*)-**TS3**	-2818.853974	-2818.109491	0.138599	-2818.248091	396.59
(*Z*)-**TS3**	-2818.850940	-2818.106552	0.138477	-2818.245029	363.93
Ph–Br (**1b**)	-244.934719	-244.837407	0.039412	-244.876819	--
Ph–Br (**1b**)[a]	-244.934626	-244.837273	0.039424	-244.876697	--
Ph–Br (**1b**)[b]	-245.063793	-244.966480	0.039412	-245.005893	--
PhN_2–$SiMe_3$ (**2b**)	-750.415185	-750.191238	0.062070	-750.253308	--
$CsCO_3^-$	-284.137090	-284.116486	0.039454	-284.155940	--
$CsCO_3$–$SiMe_3$	-693.439797	-693.298599	0.060638	-693.359237	--
Br^-	-13.405673	-13.403036	0.017506	-13.420542	--
(*E*)-PhN_2–Ph ((*E*)-**3bb**)	-572.764766	-572.563559	0.053588	-572.617147	--
(*Z*)-PhN_2–Ph ((*Z*)-**3bb**)	-572.745639	-572.544932	0.053156	-572.598089	--
**Figure 2**					

**TS4**	-3241.485421	-3240.604732	0.162748	-3240.780902	63.20
$Me_3Si–Br$	-422.630025	-422.508171	0.047113	-422.555284	--
**Figure 3**					
**G**	-2818.849774	-2818.104726	0.143979	-2818.248705	--
**TS5**	-2818.831420	-2818.088211	0.141017	-2818.229228	285.58
**TS5’**	-2818.828549	-2818.085450	0.139985	-2818.225435	272.55
**H**	-2709.412904	-2708.677506	0.135143	-2708.812650	--
**TS6**	-2709.394260	-2708.660839	0.134414	-2708.795253	340.64
N_2	-109.525678	-109.516495	0.021224	-109.537719	--
Ph–Ph (**4bb**)	-463.312073	-463.121464	0.048085	-463.169549	--
**Figure 4 and Figures S9 and S10**					
(dtbpf)Pd (**A**^{dtbpf})	-1950.958542	-1950.283664	0.114536	-1950.398200	--
(dppb)Pd (**A**^{dppb})	-1894.625735	-1894.120520	0.101235	-1894.221755	--
**B**^{dtbpf}	-2195.904625	-2195.131533	0.131210	-2195.262743	--
**B**^{dppb}	-2139.581559	-2138.980176	0.119669	-2139.099845	--
**TS1**^{dtbpf}	-2195.884787	-2195.113321	0.131473	-2195.244794	132.80
**TS1**^{dppb}	-2139.565598	-2138.964988	0.118475	-2139.083463	121.55
**C**^{dtbpf}	-2195.923715	-2195.149428	0.131685	-2195.281113	--
**C**^{dppb}	-2139.626586	-2139.022986	0.117539	-2139.140526	--
(*Z*)-**F**^{dtbpf}	-2523.688527	-2522.811465	0.142778	-2522.954243	--
(*Z*)-**F**^{dppb}	-2467.388160	-2466.682675	0.130013	-2466.812687	--
**TS2**^{dtbpf}	-2523.671300	-2522.795789	0.142873	-2522.938662	445.79
**TS2**^{dppb}	-2467.369891	-2466.665425	0.129388	-2466.794812	348.57
(*E*)-**F**^{dtbpf}	-2523.703851	-2522.827174	0.144038	-2522.971212	--
(*E*)-**F**^{dppb}	-2467.397163	-2466.691537	0.131678	-2466.823214	--
(*E*)-**TS3**^{dtbpf}	-2523.676246	-2522.801760	0.143197	-2522.944957	399.35
(*E*)-**TS3**^{dppb}	-2467.361345	-2466.657865	0.130264	-2466.788130	389.57
**G**^{dtbpf}	-2523.678894	-2522.804618	0.148437	-2522.953055	--
**G**^{dppb}	-2467.358851	-2466.653615	0.133981	-2466.787596	--
**TS5**^{dtbpf}	-2523.668001	-2522.795522	0.145738	-2522.941260	217.94
**TS5**^{dppb}	-2467.348511	-2466.645459	0.131063	-2466.776523	254.41
**TS5’**^{dppb}	-2467.341966	-2466.638548	0.130231	-2466.768779	252.25
**Scheme S2**					
**C**^{carbonate}	-2761.877140	-2761.213612	0.138389	-2761.352002	--

Cs_2CO_3	-304.332047	-304.308564	0.046674	-304.355238	--
CsBr	-33.567325	-33.562648	0.030963	-33.593611	--
**Scheme S3**					
**S1**	-1034.587458	-1034.342797	0.080141	-1034.422938	--
**S2**	-1034.598001	-1034.353673	0.077494	-1034.431167	--
phenyl anion	-231.647156	-231.557034	0.034667	-231.591701	--
**Scheme S4**					
Cs^+	-20.086950	-20.084313	0.018330	-20.102644	--
CO_3^{2-}	-263.886286	-263.867810	0.030404	-263.898214	--
**Figure S5**					
$\mathbf{D^1}$	-3241.521661	-3240.652831	0.165725	-3240.818557	--
$\mathbf{D^2}$	-3241.531848	-3240.663067	0.165117	-3240.828184	--
**Figure S7**					
**TS7**	-2996.526157	-2995.756751	0.144229	-2995.900980	183.55
**I**	-2996.545171	-2995.774451	0.145844	-2995.920295	--
**J**	-2996.530352	-2995.761470	0.149411	-2995.910881	--
**Figure S8**					
**K**	-3512.309355	-3511.421027	0.175926	-3511.596952	--
**TS8**	-3512.275860	-3511.387994	0.171407	-3511.559402	69.42
**L**	-3512.279857	-3511.391939	0.173814	-3511.565754	--
**TS9**	-3512.274995	-3511.388163	0.172854	-3511.561017	92.32
**Figure S11**					
**For transition state TS1**					
Ph–Br (**1b**) from **TS1**	-244.905160	--	--	--	--
(dppf)Pd (**A**) from **TS1**	-2246.106870	--	--	--	--
Ph–Br (**1b**) from $\mathbf{TS1^{dtbpf}}$	-244.896093	--	--	--	--
(dtbpf)Pd ($\mathbf{A^{dtbpf}}$) from $\mathbf{TS1^{dtbpf}}$	-1950.944513	--	--	--	--
Ph–Br (**1b**) from $\mathbf{TS1^{dppb}}$	-244.902036	--	--	--	--
(dppb)Pd ($\mathbf{A^{dppb}}$) from $\mathbf{TS1^{dppb}}$	-1894.610904	--	--	--	--

**For transition state (*E*)-TS3**					
**Fr1** from (*E*)-**F**	-341.135800	--	--	--	--
**Fr1** from (*E*)-**TS3**	-341.133193	--	--	--	--
**Fr2** from (*E*)-**F**	-2477.593722	--	--	--	--
**Fr2** from (*E*)-**TS3**	-2477.557998	--	--	--	--
**Fr1** from (*E*)-$\mathbf{F^{dtbpf}}$	-341.135777	--	--	--	--
**Fr1** from (*E*)-$\mathbf{TS3^{dtbpf}}$	-341.132718	--	--	--	--
**Fr2** from (*E*)-$\mathbf{F^{dtbpf}}$	-2182.418753	--	--	--	--
**Fr2** from (*E*)-$\mathbf{TS3^{dtbpf}}$	-2182.386543	--	--	--	--
**Fr1** from (*E*)-$\mathbf{F^{dppb}}$	-341.135745	--	--	--	--
**Fr1** from (*E*)-$\mathbf{TS3^{dppb}}$	-341.132769	--	--	--	--
**Fr2** from (*E*)-$\mathbf{F^{dppb}}$	-2126.105998	--	--	--	--
**Fr2** from (*E*)-$\mathbf{TS3^{dppb}}$	-2126.071893	--	--	--	--
**For transition state TS5**					
**Fr3** from (*Z*)-**F**	-341.133595	--	--	--	--
**Fr3** from **TS5**	-341.124367	--	--	--	--
**Fr4** from (*Z*)-**F**	-2477.594090	--	--	--	--
**Fr4** from **TS5**	-2477.538909	--	--	--	--
**Fr3** from (*Z*)-$\mathbf{F^{dtbpf}}$	-341.134049	--	--	--	--

**Fr3** from **TS5**^{dtbpf}	-341.121271	--	--	--	--
**Fr4** from (*Z*)-**F**^{dtbpf}	-2182.401718	--	--	--	--
**Fr4** from **TS5**^{dtbpf}	-2182.377634	--	--	--	--
**Fr3** from (*Z*)-**F**^{dppb}	-341.133802	--	--	--	--
**Fr3** from **TS5**^{dppb}	-341.123131	--	--	--	--
**Fr4** from (*Z*)-**F**^{dppb}	-2126.096654	--	--	--	--
**Fr4** from **TS5**^{dppb}	-2126.051097	--	--	--	--
**Table S2**					
(*E*)-**F**^{2c}	-2918.141691	-2917.402799	0.141933	-2917.544732	--
(*E*)-**F**^{2d}	-2933.420157	-2932.639332	0.145923	-2932.785255	--
(*E*)-**F**^{2e}	-3155.991186	-3155.237008	0.148716	-3155.385725	--
(*E*)-**F**^{2f}	-2918.136720	-2917.397628	0.141582	-2917.539210	--
(*E*)-**F**^{2g}	-2933.414613	-2932.633711	0.145288	-2932.779000	--
(*E*)-**F**^{2h}	-3155.989404	-3155.234867	0.147047	-3155.381914	--
(*E*)-**TS3**^{2c}	-2918.109506	-2917.372456	0.140990	-2917.513446	396.82
(*E*)-**TS3**^{2d}	-2933.387777	-2932.608879	0.145097	-2932.753976	397.21
(*E*)-**TS3**^{2e}	-3155.960752	-3155.208405	0.147919	-3155.356323	392.37
(*E*)-**TS3**^{2f}	-2918.104679	-2917.367486	0.140660	-2917.508146	357.13
(*E*)-**TS3**^{2g}	-2933.384518	-2932.605317	0.144033	-2932.749350	340.93
(*E*)-**TS3**^{2h}	-3155.959005	-3155.206421	0.146232	-3155.352652	379.25
**G**^{2c}	-2918.105186	-2917.367568	0.145973	-2917.513541	--
**G**^{2d}	-2933.385370	-2932.605715	0.149766	-2932.755481	--
**G**^{2e}	-3155.953652	-3155.200921	0.153960	-3155.354881	--
**G**^{2f}	-2918.100126	-2917.362292	0.146438	-2917.508731	--
**G**^{2g}	-2933.379570	-2932.599854	0.150822	-2932.750676	--
**G**^{2h}	-3155.951474	-3155.198628	0.151761	-3155.350390	--
**TS5**^{2c}	-2918.086439	-2917.350555	0.143157	-2917.493712	276.12
**TS5**^{2d}	-2933.368875	-2932.590928	0.147403	-2932.738330	259.47
**TS5**^{2e}	-3155.936476	-3155.185429	0.150687	-3155.336116	287.80
**TS5**^{2f}	-2918.088524	-2917.352434	0.142814	-2917.495249	210.68

**TS5[2g]**	-2933.370410	-2932.591931	0.146413	-2932.738344	172.28
**TS5[2h]**	-3155.936352	-3155.185052	0.149302	-3155.334354	264.86
**2c**	-849.670532	-849.453891	0.064251	-849.518142	--
**2d**	-864.951267	-864.692646	0.068630	-864.761277	--
**2e**	-1087.518271	-1087.286575	0.072524	-1087.359098	--
**2f**	-849.665542	-849.448794	0.064535	-849.513329	--
**2g**	-864.942339	-864.683517	0.068260	-864.751777	--
**2h**	-1087.514819	-1087.282829	0.071530	-1087.354358	--
(*E*)-**3bc**	-672.019922	-671.826029	0.055780	-671.881809	--
(*E*)-**3bd**	-687.300888	-687.065029	0.060116	-687.125144	--
(*E*)-**3be**	-909.867720	-909.658763	0.064048	-909.722811	--
(*E*)-**3bf**	-672.015332	-671.821311	0.055947	-671.877258	--
(*E*)-**3bg**	-687.291642	-687.055825	0.060251	-687.116075	--
(*E*)-**3bh**	-909.864342	-909.655218	0.063369	-909.718587	--
**4bc**	-562.567043	-562.383811	0.050395	-562.434207	--
**4bd**	-577.846465	-577.621377	0.054828	-577.676204	--
**4be**	-800.416368	-800.218014	0.058548	-800.276562	--
**4bf**	-562.564853	-562.381416	0.050318	-562.431733	--
**4bg**	-577.843172	-577.617961	0.054761	-577.672722	--
**4bh**	-800.410260	-800.211828	0.057689	-800.269517	--

[a] Calculated at the ωB97X-D/def2-TZVP+SMD(toluene)//TPSS-D3(BJ)/def2-SVP+SMD(toluene) level of theory. [b] Calculated at the ωB97X-D/def2-QZVP+SMD(toluene)//TPSS-D3(BJ)/def2-SVP level of theory.

Cartesian Coordinates

(dppf)Pd (**A**)

C	3.86425700	-4.19070800	0.11906300
C	4.60509300	-3.04811200	0.46034200
C	4.07306100	-1.77048000	0.23724800
C	2.79042800	-1.62645900	-0.32733100
C	2.06152400	-2.77637900	-0.68523900
C	2.59246500	-4.05365800	-0.45720000
P	2.00834300	0.02573000	-0.60564000
C	3.49275500	1.06222300	-0.98540500
C	4.07805100	1.94614000	-0.05861700
C	5.19468300	2.71424100	-0.42316700
C	5.73962300	2.60299100	-1.70993100
C	5.15882600	1.72460800	-2.63957700
C	4.03704100	0.96762100	-2.28288600
C	1.63338300	0.54693500	1.11015100
C	0.81474300	1.69468200	1.43086600
C	0.72368100	1.79677500	2.85787800
C	1.47023500	0.71102400	3.42996400
C	2.03101200	-0.06183400	2.35727100
Fe	0.00004000	0.00075300	2.20502500
C	-0.72357600	-1.79491000	2.85887500
C	-0.81463800	-1.69360300	1.43180900
C	-1.63330900	-0.54605300	1.11046700
C	-2.03093800	0.06340000	2.35725200
C	-1.47014100	-0.70885500	3.43036800
P	-2.00834800	-0.02585800	-0.60561600
C	-3.49241300	-1.06300200	-0.98490500
C	-4.07782300	-1.94618400	-0.05748400
C	-5.19419600	-2.71483000	-0.42167000
C	-5.73876300	-2.60485900	-1.70870400
C	-5.15785200	-1.72721700	-2.63897600
C	-4.03631800	-0.96968800	-2.28263500
Pd	-0.00002400	-0.00017400	-1.63619200
C	-2.79086000	1.62627500	-0.32817400
C	-2.06196000	2.77616100	-0.68620800
C	-2.59313900	4.05345600	-0.45883100
C	-3.86518000	4.19055900	0.11687000
C	-4.60601400	3.04799400	0.45824600
C	-4.07373500	1.77034300	0.23583500
H	0.15135600	2.54486200	3.40938700
H	0.33354200	2.34657400	0.70172600
H	2.62752300	-0.96947400	2.45560400
H	1.56217600	0.48698300	4.49462600
H	-2.62746700	0.97108100	2.45509400
H	-0.33340400	-2.34589300	0.70304900
H	-1.56207700	-0.48423100	4.49490700
H	-0.15123200	-2.54268300	3.41079100
H	-1.07779000	2.64600300	-1.15566000
H	-4.65661500	0.87961100	0.49482900
H	-2.01791800	4.94208600	-0.74051700
H	-5.60469600	3.15291400	0.89631800
H	-4.28496700	5.18744600	0.28979800
H	-3.65906800	-2.03086400	0.95042600
H	-3.57020500	-0.29753500	-3.01323500
H	-5.64131500	-3.40068400	0.30646000
H	-5.57575200	-1.64132600	-3.64798200
H	-6.61049100	-3.20534000	-1.99013400
H	3.65901100	2.03182500	0.94908900
H	3.57103800	0.29487800	-3.01301500
H	5.64171100	3.40067200	0.30447500
H	5.57702000	1.63770800	-3.64837500
H	6.61155500	3.20304400	-1.99164300
H	4.65593800	-0.87971700	0.49615500
H	1.07754400	-2.64626500	-1.15511600
H	5.60358100	-3.15300000	0.89886100
H	2.01725300	-4.94232000	-0.73880300
H	4.28385400	-5.18758500	0.29251300

(dppf)Pd (**A**) with SMD(toluene)

C	-3.90248600	4.18652000	0.08758600
C	-4.63917700	3.04127700	0.42949200
C	-4.09560900	1.76552500	0.22300800
C	-2.80578400	1.62595800	-0.32690900
C	-2.08011700	2.77866600	-0.68380100
C	-2.62252600	4.05405800	-0.47161800
P	-2.01007100	-0.02260200	-0.59037400
C	-3.48062700	-1.07449200	-0.98727000
C	-4.05479600	-1.98266400	-0.07689800

C	-5.15472500	-2.76662200	-0.45898700
C	-5.69481100	-2.64717600	-1.74721500
C	-5.12572100	-1.74412800	-2.66066800
C	-4.02011300	-0.97147600	-2.28675000
C	-1.63928700	-0.53898100	1.12766200
C	-0.82723200	-1.69106900	1.45068200
C	-0.73673500	-1.79035100	2.87800700
C	-1.47567200	-0.69827200	3.44754600
C	-2.03180900	0.07591900	2.37339500
Fe	0.00000000	0.00001000	2.22019400
C	0.73673500	1.79037600	2.87799200
C	0.82723200	1.69108200	1.45066700
C	1.63928700	0.53899100	1.12765700
C	2.03180900	-0.07589900	2.37339500
C	1.47567100	0.69830100	3.44754000
P	2.01007000	0.02260000	-0.59037500
C	3.48063000	1.07448400	-0.98727800
C	4.05480400	1.98265600	-0.07691000
C	5.15473800	2.76660500	-0.45900200
C	5.69482300	2.64715000	-1.74723100
C	5.12572600	1.74410300	-2.66068000
C	4.02011400	0.97145900	-2.28675800
Pd	-0.00000100	-0.00000400	-1.61825000
C	2.80578000	-1.62595900	-0.32690000
C	2.08011700	-2.77866800	-0.68379300
C	2.62252400	-4.05406000	-0.47160100
C	3.90248000	-4.18651900	0.08761300
C	4.63916900	-3.04127500	0.42952100
C	4.09560200	-1.76552400	0.22302700
H	-0.16987100	-2.54157800	3.43150100
H	-0.35426600	-2.35074600	0.72261100
H	-2.62322900	0.98702500	2.47243600
H	-1.56582700	-0.47086300	4.51193800
H	2.62322800	-0.98700400	2.47244500
H	0.35426900	2.35075400	0.72259100
H	1.56582700	0.47090200	4.51193400
H	0.16987100	2.54160800	3.43147900
H	1.08833300	-2.65455800	-1.13838100
H	4.67581200	-0.87428400	0.48590300
H	2.04931300	-4.94449900	-0.75202700
H	5.64321300	-3.14226500	0.85630400
H	4.33114800	-5.18180400	0.24815400
H	3.64216300	2.07571000	0.93277700
H	3.56475700	0.27922500	-3.00556500
H	5.59253900	3.47152600	0.25662100
H	5.54017000	1.64961200	-3.67046600
H	6.55388100	3.25934300	-2.04250000
H	-3.64215400	-2.07571100	0.93278900
H	-3.56476100	-0.27924100	-3.00556100
H	-5.59252200	-3.47154200	0.25664000
H	-5.54016600	-1.64964400	-3.67045400
H	-6.55386600	-3.25937500	-2.04248200
H	-4.67582100	0.87428600	0.48588400
H	-1.08833100	2.65455400	-1.13838200
H	-5.64322500	3.14226800	0.85626800
H	-2.04931300	4.94449600	-0.75204400
H	-4.33115700	5.18180500	0.24811800

(dtbpf)Pd (**A**^{dtbpf})

P	2.08187900	-0.66044400	0.03312500
C	1.72044300	1.10946100	-0.35943300
C	1.03010400	1.45872300	-1.58308900
C	0.93497100	2.88489600	-1.68110000
C	1.54155500	3.44170700	-0.50741500
C	2.02166400	2.35729300	0.30358200
Fe	0.00000900	2.20530200	-0.00014900
C	-0.93496700	2.88504500	1.68073300
C	-1.03006600	1.45885900	1.58286700
C	-1.72039200	1.10945700	0.35924200
C	-2.02164500	2.35721400	-0.30389800
C	-1.54156400	3.44172300	0.50699000
P	-2.08190000	-0.66049800	-0.03302400
Pd	0.00000700	-1.56281800	0.00017200
H	0.45215400	3.44208900	-2.48585900
H	0.62155600	0.74175800	-2.29053200
H	2.51700000	2.47421600	1.26703800
H	1.60088400	4.50175600	-0.25243800
H	-2.51698000	2.47402500	-1.26736800
H	-0.62149800	0.74197200	2.29037700
H	-1.60091300	4.50174400	0.25190300
H	-0.45216300	3.44233500	2.48543200
C	3.39987400	-1.04278400	-1.32668500

C	4.18753000	-2.30881000	-0.94272500
C	2.59344400	-1.36476400	-2.60486100
C	4.35987600	0.12578300	-1.61312300
H	4.79033000	-2.63628000	-1.81073600
H	4.88272300	-2.13122700	-0.10598300
H	3.50937400	-3.13664500	-0.66997800
H	2.08488300	-0.47294000	-3.00307400
H	3.28336300	-1.73333900	-3.38794600
H	1.82914200	-2.13722700	-2.40656700
H	5.03713400	-0.15083600	-2.44434100
H	3.80890500	1.03252600	-1.91330600
H	4.98540400	0.37643300	-0.74217400
C	2.92217000	-0.66089500	1.75521800
C	4.27074200	0.07388900	1.84143300
C	1.90166700	-0.03576400	2.72977000
C	3.08809800	-2.13434100	2.19255600
H	5.05178200	-0.43955600	1.25685800
H	4.20615900	1.11426600	1.48339500
H	4.61055700	0.09860200	2.89471500
H	0.94982200	-0.59460300	2.67677900
H	2.29518700	-0.10688400	3.76139500
H	1.69046200	1.02114300	2.50963600
H	2.13316500	-2.67772200	2.07688400
H	3.86064400	-2.66685100	1.61901700
H	3.38044900	-2.16252800	3.25939900
C	-2.92209400	-0.66129200	-1.75518200
C	-4.27072900	0.07333800	-1.84165000
C	-1.90158100	-0.03622100	-2.72976200
C	-3.08785400	-2.13482300	-2.19229300
H	-5.05172500	-0.43995600	-1.25688600
H	-4.20623100	1.11385400	-1.48400400
H	-4.61054700	0.09762800	-2.89494200
H	-0.94970400	-0.59499400	-2.67664500
H	-2.29503700	-0.10751500	-3.76139900
H	-1.69046100	1.02073100	-2.50976700
H	-3.38010800	-2.16320400	-3.25915800
H	-2.13288300	-2.67810000	-2.07644600
H	-3.86040600	-2.66730900	-1.61873700
C	-3.39998900	-1.04241700	1.32680700
C	-2.59366100	-1.36442600	2.60504100
C	-4.35971500	0.12639200	1.61309500
C	-4.18789400	-2.30832300	0.94297300
H	-1.82946500	-2.13701700	2.40683500
H	-2.08499000	-0.47264500	3.00320500
H	-3.28366600	-1.73282800	3.38813100
H	-4.98501500	0.37723600	0.74203700
H	-5.03719500	-0.15007400	2.44418800
H	-3.80853800	1.03296400	1.91340700
H	-4.79076800	-2.63557900	1.81101500
H	-4.88304100	-2.13070900	0.10620200
H	-3.50988900	-3.13632200	0.67034600

(dppb)Pd (**A**^{dppb})

C	-1.65449300	-0.66878200	1.90498700
C	1.53319100	-0.14979300	1.85999800
C	-0.89150900	0.44727400	2.64647500
C	0.43752900	0.93231000	2.03868400
H	1.16912900	-1.14123700	2.18365000
H	-1.08977100	-1.61799000	1.93750700
H	-0.71092000	0.08890300	3.67927400
H	-2.61883200	-0.84305600	2.41766300
H	2.43498800	0.07781200	2.45689500
H	-1.55893500	1.32387700	2.73640800
H	0.83144400	1.73865600	2.68204100
H	0.22268700	1.40453900	1.06123300
P	-1.99786200	-0.34659600	0.07987400
P	2.00296100	-0.31128900	0.04959400
Pd	-0.00379000	-0.61966200	-0.94731200
C	-3.50743100	-1.37354000	-0.19622800
C	-4.47285200	-0.96242500	-1.14069800
C	-3.66322700	-2.62948200	0.42663200
C	-5.57335800	-1.77569400	-1.43601100
H	-4.36226000	0.00612300	-1.64101100
C	-4.76404200	-3.44396800	0.12539400
H	-2.91955400	-2.98170700	1.14897600
C	-5.72466100	-3.02002300	-0.80364800
H	-6.31783600	-1.43622700	-2.16461800
H	-4.87080300	-4.41491300	0.62159000
H	-6.58522700	-3.65601100	-1.03651300
C	-2.70496700	1.35852600	0.15134700
C	-3.88503900	1.65253200	0.86477600
C	-2.00099100	2.40440700	-0.47302500

C	-4.34741700	2.97210500	0.95067500
H	-4.44613600	0.84359300	1.34619200
C	-2.46196200	3.72594800	-0.38397900
H	-1.08426500	2.16059400	-1.02376900
C	-3.63557200	4.01079900	0.32826500
H	-5.26607900	3.19295700	1.50516900
H	-1.90479700	4.53247100	-0.87269300
H	-3.99935400	5.04166600	0.39788800
C	3.47730100	-1.41928000	0.10199900
C	4.42444600	-1.34612900	-0.94299000
C	3.64018600	-2.40850600	1.09311700
C	5.51091000	-2.22670300	-0.98495900
H	4.30821200	-0.58639000	-1.72439600
C	4.72676100	-3.29454500	1.04554800
H	2.91718300	-2.49391700	1.91044500
C	5.66641600	-3.20627600	0.00955000
H	6.24048000	-2.15012600	-1.79865100
H	4.83936300	-4.05530000	1.82588200
H	6.51540600	-3.89716600	-0.02527600
C	2.79311100	1.33536800	-0.24156900
C	3.95613100	1.74370500	0.44204800
C	2.17879400	2.22356000	-1.14355600
C	4.48871400	3.02141300	0.22801700
H	4.45004500	1.05423600	1.13636100
C	2.70955300	3.50468100	-1.35508200
H	1.27975700	1.88670400	-1.67601700
C	3.86475900	3.90456700	-0.66858400
H	5.39443500	3.33113400	0.76092100
H	2.22378800	4.18826200	-2.05984900
H	4.28404100	4.90294400	-0.83404200

intermediate **B**

C	4.89359000	0.85352600	-2.82865500
C	5.21657900	0.41497900	-1.53552900
C	4.23200100	-0.14471300	-0.70918400
C	2.90552300	-0.26385800	-1.16750900
C	2.57995800	0.21075100	-2.45759100
C	3.57175500	0.74578400	-3.28820500
P	1.53981700	-0.78413900	-0.06018900
C	2.38401400	-1.61503800	1.34708700
C	2.97775400	-2.88726300	1.22150200
C	3.58774100	-3.48913500	2.32934100
C	3.61026700	-2.82698600	3.56800800
C	3.02210200	-1.56056400	3.69732000
C	2.40709100	-0.95625900	2.59100500
C	0.82179100	-2.22318500	-0.93545700
C	-0.15240000	-3.10099900	-0.32618200
C	-0.54410600	-4.07991400	-1.29743100
C	0.17041700	-3.81132700	-2.51251100
C	1.01501100	-2.67222400	-2.29311300
Fe	-0.94524100	-2.18899700	-1.95496000
C	-1.70426800	-1.16926100	-3.56173300
C	-1.41544400	-0.27932300	-2.47555000
C	-2.18111700	-0.70818600	-1.32477900
C	-2.93912000	-1.87327700	-1.71747800
C	-2.64226200	-2.15194100	-3.09418100
P	-2.01701200	0.13333800	0.27928100
C	-3.37537000	1.37095000	0.23436100
C	-4.01631000	1.74047700	-0.96334700
C	-4.92914200	2.80637900	-0.97570900
C	-5.21436200	3.50785600	0.20372600
C	-4.58209200	3.13895400	1.40370000
C	-3.66370300	2.08397500	1.41792600
Pd	0.12553000	0.99118700	0.40482600
C	1.28974900	2.79687300	0.30296200
C	1.92696000	2.99670300	-0.96221500
C	1.17501300	3.44901800	-2.03576200
C	-0.19866900	3.79278100	-1.87876700
C	-0.81093700	3.68261800	-0.64021800
C	-0.09674000	3.16997000	0.49284900
C	-2.60946500	-1.08757000	1.51997600
C	-1.67458100	-1.57876200	2.45174500
C	-2.06101300	-2.52632500	3.41027500
C	-3.38814000	-2.97913900	3.45029200
C	-4.33068400	-2.47521300	2.53918100
C	-3.94625200	-1.52851100	1.57994400
Br	2.47770800	2.80902000	1.90480000
H	-1.28483000	-4.86695200	-1.14565100
H	-0.53065200	-3.02129300	0.69289600
H	1.68263000	-2.21705800	-3.02445800
H	0.07095800	-4.35903900	-3.45152000
H	-3.58795000	-2.45931500	-1.06564500

H	-0.73428200	0.57568600	-2.47653800
H	-3.03275500	-2.99089400	-3.67353600
H	-1.26400600	-1.12636000	-4.55945600
H	2.98936100	2.76402700	-1.07186600
H	-0.48729800	3.35262800	1.49961800
H	1.65593500	3.55248400	-3.01457500
H	-1.85036000	3.99767500	-0.49999400
H	-0.76279000	4.17669900	-2.73524700
H	-0.64322200	-1.20689600	2.41253300
H	-4.68502400	-1.11966400	0.88175300
H	-1.32583800	-2.90475200	4.12845100
H	-5.37023700	-2.81823300	2.58081600
H	-3.69331800	-3.71729400	4.19978600
H	-3.79508200	1.19719700	-1.88734000
H	-3.15518500	1.81223200	2.35054800
H	-5.42033500	3.08774300	-1.91366600
H	-4.80092900	3.68269100	2.32911500
H	-5.92588700	4.34024700	0.19108200
H	2.95510000	-3.40389300	0.25600200
H	1.94752800	0.03555600	2.67689900
H	4.04680800	-4.47862200	2.22814800
H	3.03941100	-1.04034700	4.66094700
H	4.08623400	-3.30173400	4.43290700
H	4.49094100	-0.48178300	0.29930300
H	1.54127900	0.17546900	-2.80085600
H	6.24365500	0.50657700	-1.16607600
H	3.30664600	1.10084900	-4.29003800
H	5.66670100	1.28626700	-3.47227900

intermediate **B** with SMD(toluene)

C	4.92236000	0.70132200	-2.88529500
C	5.24439200	0.26607200	-1.59099700
C	4.24736400	-0.23698000	-0.74259800
C	2.91040800	-0.30230500	-1.18063200
C	2.58730000	0.16911600	-2.47260000
C	3.59000600	0.64820400	-3.32451700
P	1.53272500	-0.77495200	-0.06309800
C	2.36070100	-1.56609300	1.37808700
C	2.96227400	-2.83846600	1.29173800
C	3.55559000	-3.41264100	2.42331600
C	3.55316100	-2.72382000	3.64762400
C	2.95759000	-1.45746400	3.73841100
C	2.36059500	-0.88079700	2.60763300
C	0.81597900	-2.24284200	-0.89304600
C	-0.13555200	-3.12144600	-0.24897100
C	-0.51437900	-4.13703800	-1.18727400
C	0.18446200	-3.89046200	-2.41590100
C	1.00669100	-2.72771700	-2.23879100
Fe	-0.95892900	-2.27347800	-1.89758800
C	-1.75925800	-1.34414200	-3.53954100
C	-1.45823000	-0.39929900	-2.50441400
C	-2.19907100	-0.77624300	-1.31928000
C	-2.95293700	-1.96636800	-1.63962000
C	-2.67880300	-2.30998400	-3.00597000
P	-2.01744400	0.14216500	0.23944000
C	-3.39999700	1.35476200	0.17368900
C	-4.06780700	1.67117900	-1.02480400
C	-5.02037500	2.70164900	-1.05184400
C	-5.31847900	3.42096800	0.11385500
C	-4.65726200	3.10725900	1.31384800
C	-3.69987300	2.08722600	1.34282200
Pd	0.12519600	1.03198300	0.33524500
C	1.29761300	2.81716800	0.25307100
C	1.95252500	3.03744200	-1.00167600
C	1.22297400	3.53215400	-2.07143500
C	-0.14668900	3.89872000	-1.92227000
C	-0.77739700	3.76222100	-0.69622600
C	-0.08800100	3.20585800	0.43275100
C	-2.56117900	-1.02438600	1.55358300
C	-1.59956500	-1.45091500	2.49000000
C	-1.94969000	-2.35154200	3.50605000
C	-3.26769700	-2.82286200	3.59827800
C	-4.23677300	-2.38434300	2.68097700
C	-3.88883700	-1.48403500	1.66474800
Br	2.48203700	2.86079000	1.87468800
H	-1.23769200	-4.93432500	-1.00586500
H	-0.50468400	-3.02100000	0.77180700
H	1.65733800	-2.28196900	-2.99131900
H	0.08839700	-4.46770500	-3.33772700
H	-3.58591200	-2.52579200	-0.94980700
H	-0.78198300	0.45680100	-2.56434500
H	-3.07168000	-3.18073900	-3.53509400

H	-1.33721600	-1.34752900	-4.54632000
H	3.01186000	2.78865300	-1.10715300
H	-0.48800400	3.38108600	1.43748400
H	1.71676700	3.65115700	-3.04231300
H	-1.81181200	4.09554900	-0.55996900
H	-0.69121100	4.31816600	-2.77502800
H	-0.57492500	-1.06865400	2.40890100
H	-4.65016600	-1.12889000	0.96171900
H	-1.19272600	-2.67943500	4.22655600
H	-5.26903600	-2.74224800	2.76159000
H	-3.54487500	-3.52465500	4.39235500
H	-3.84069300	1.11312300	-1.93868000
H	-3.17375700	1.85685700	2.27684500
H	-5.53345000	2.93980700	-1.99019100
H	-4.88549800	3.66506600	2.22872100
H	-6.06244100	4.22446700	0.09017800
H	2.96125100	-3.37820000	0.33871100
H	1.89578400	0.11032700	2.66460800
H	4.02076600	-4.40189200	2.35135000
H	2.95493400	-0.91575200	4.69040200
H	4.01538800	-3.17748800	4.53117100
H	4.50823300	-0.57282200	0.26585500
H	1.54365300	0.17140000	-2.80305300
H	6.28005400	0.31515900	-1.23729100
H	3.32638300	1.00070600	-4.32773700
H	5.70479000	1.08936800	-3.54622200

intermediate **B**^{dtbpf}

P	0.16904200	1.86637200	0.17049600
C	-1.60144300	2.04444300	-0.29859400
C	-2.07758900	1.62194200	-1.60007600
C	-3.46159900	1.96926000	-1.73047800
C	-3.87056900	2.59687300	-0.50892800
C	-2.73542300	2.64760300	0.36757700
Fe	-3.15370300	0.71684100	-0.14864400
C	-4.28812500	-0.17673200	1.29640800
C	-2.90865600	-0.54506700	1.41316300
C	-2.47985600	-1.18638700	0.18643100
C	-3.63423800	-1.18915200	-0.68741200
C	-4.73498600	-0.56880400	-0.00695400
P	-0.77737300	-1.86399000	0.00520300
Pd	1.04017000	-0.34308600	0.17401500
C	3.22316100	-0.40676800	-0.02148700
C	4.08565700	0.44144800	0.73758500
C	4.33228700	0.14582900	2.06786500
C	3.78940800	-1.02999500	2.65935200
C	3.05490100	-1.92347100	1.89709000
C	2.76172300	-1.66843300	0.51259600
Br	3.46227300	-0.33633800	-2.00172900
H	-4.09733200	1.75393200	-2.59106200
H	-1.48481200	1.09567500	-2.34328700
H	-2.74938800	3.05683000	1.37669500
H	-4.87668900	2.94529200	-0.26773800
H	-3.67377800	-1.56758200	-1.70752500
H	-2.27621300	-0.34995800	2.27451600
H	-5.72705700	-0.39669800	-0.42876700
H	-4.87726400	0.34459900	2.05281100
H	4.52866300	1.32167600	0.26388000
H	2.59250700	-2.51875500	-0.15236500
H	4.94928100	0.82422000	2.66634200
H	2.73858000	-2.87908000	2.32932200
H	4.00332500	-1.25590800	3.70928200
C	0.27710600	2.50881000	1.97573700
C	-0.65384700	1.61662300	2.82286200
C	1.70451400	2.22612000	2.48530700
C	-0.06685700	3.99415200	2.18092200
H	-1.71820300	1.75403900	2.58414600
H	-0.39191300	0.55499600	2.66651700
H	-0.50391000	1.85583400	3.89246200
H	2.47798200	2.78607000	1.94074400
H	1.76636400	2.50928000	3.55328600
H	1.93816300	1.15216900	2.39371400
H	-0.09210700	4.21212300	3.26582600
H	0.69637000	4.65128000	1.73316500
H	-1.04595100	4.27001000	1.75807800
C	0.91405600	3.18797100	-1.02512300
C	2.34219400	3.53244500	-0.56890400
C	1.00753000	2.50449400	-2.40739500
C	0.06724600	4.46610400	-1.16500800
H	2.35145800	4.13050100	0.35709100
H	2.93447400	2.61769800	-0.40739300
H	2.84085900	4.12746300	-1.35651200

H	0.01431200	2.37004700	-2.86298400
H	1.59758800	3.14873900	-3.08622300
H	1.50850600	1.52509000	-2.33966300
H	0.52891300	5.11790100	-1.93151600
H	-0.95968900	4.23573000	-1.49368700
H	0.01063700	5.04214400	-0.22939300
C	-0.74398800	-2.63309100	-1.75362400
C	-1.76676900	-3.75888000	-1.99105500
C	-0.96688100	-1.46405600	-2.73808100
C	0.67741300	-3.15745100	-2.05106500
H	-1.50462800	-4.66610500	-1.42224600
H	-2.79350400	-3.47142000	-1.71618700
H	-1.76472200	-4.03167800	-3.06363700
H	-0.21553000	-0.67576600	-2.55123100
H	-0.83087800	-1.83317400	-3.77172000
H	-1.96891400	-1.01887500	-2.65932300
H	0.70497600	-3.52257000	-3.09491000
H	1.41683800	-2.34461500	-1.96031400
H	0.97892400	-3.99035400	-1.39961000
C	-0.87109500	-3.28004700	1.32020700
C	0.19349000	-4.35273200	1.02434200
C	-0.51509400	-2.61390800	2.66883300
C	-2.25664200	-3.94272300	1.42886900
H	-0.06019800	-4.95690300	0.13840900
H	1.18943900	-3.90822800	0.87290600
H	0.25760000	-5.04105000	1.88790400
H	-1.31110000	-1.93437800	3.01080300
H	-0.39491900	-3.39594900	3.44247100
H	0.42425700	-2.03961300	2.59489600
H	-2.22857200	-4.70952000	2.22701600
H	-3.03761400	-3.21075500	1.69035300
H	-2.55346800	-4.44273800	0.49400000

intermediate $\mathbf{B}^{dppb}$

C	-1.87968200	1.08346300	-2.07806800
C	1.10651600	0.16672800	-3.01241000
C	-1.50111100	0.25659100	-3.32095900
C	-0.21553400	-0.58831800	-3.25967700
H	1.15219400	1.10921600	-3.58817900
H	-1.13153100	1.87585100	-1.89383300
H	-1.42699100	0.95801100	-4.17476700
H	-2.85015600	1.58340200	-2.25292500
H	1.95026400	-0.45490600	-3.35862100
H	-2.33957700	-0.42547700	-3.55405200
H	-0.13053800	-1.11726800	-4.22711700
H	-0.31965100	-1.37118400	-2.48310800
P	-1.97283200	0.09504400	-0.49656900
P	1.45070300	0.48553800	-1.19533100
Pd	0.07063200	-0.60758600	0.32095100
C	-2.89940600	1.21329200	0.62643300
C	-2.84136000	2.61655200	0.50765500
C	-3.57129200	0.65012100	1.73339000
C	-3.44721400	3.43598600	1.47185300
H	-2.31266300	3.07968600	-0.33018800
C	-4.18321800	1.47043500	2.68709000
H	-3.61170000	-0.43964800	1.84154100
C	-4.12085800	2.86837800	2.56181800
H	-3.39159200	4.52504900	1.36599700
H	-4.70841100	1.01831800	3.53552300
H	-4.59460200	3.51003700	3.31222300
C	-3.20667600	-1.20520300	-0.91689300
C	-2.78796700	-2.54906900	-0.89295100
C	-4.53334900	-0.89813500	-1.28088300
C	-3.68111200	-3.57457200	-1.23717000
H	-1.75714900	-2.77732200	-0.59194800
C	-5.42505200	-1.92386500	-1.61949100
H	-4.86936600	0.14508600	-1.28629500
C	-4.99892200	-3.26232200	-1.60055200
H	-3.34834300	-4.61773000	-1.21526200
H	-6.45662400	-1.68109400	-1.89699700
H	-5.69900400	-4.06224500	-1.86479500
C	1.21524300	2.29641700	-0.93286900
C	1.53663200	3.27307800	-1.89668100
C	0.69148200	2.70519500	0.31133200
C	1.31820800	4.63132700	-1.62843800
H	1.96527700	2.97510800	-2.85979700
C	0.48291400	4.06500700	0.58155700
H	0.43872900	1.93995900	1.05790200
C	0.78917200	5.02905500	-0.38987200
H	1.56527700	5.38279500	-2.38623900
H	0.06842600	4.36597400	1.54928000
H	0.61814500	6.09128400	-0.18406700

C	3.27035100	0.29099000	-1.04523400
C	3.83646100	-0.96500200	-1.35696800
C	4.08275500	1.27346300	-0.44305600
C	5.18715200	-1.22072400	-1.09723400
H	3.20873000	-1.75869500	-1.77719200
C	5.43405100	1.00969800	-0.17643100
H	3.65523400	2.24531000	-0.17789100
C	5.99159900	-0.23443000	-0.50365800
H	5.60946200	-2.20101700	-1.34242900
H	6.05361600	1.78311900	0.29075900
H	7.04621800	-0.43903300	-0.29105400
C	1.31429400	-1.50463000	1.79543000
C	-0.01890400	-1.44002800	2.35800200
C	2.36662100	-0.66452200	2.28164100
C	-0.26183100	-0.41428900	3.33208000
H	-0.69385700	-2.29912700	2.29680100
C	2.06357500	0.32082300	3.20662700
H	3.37696700	-0.78242800	1.88058600
C	0.74179100	0.45831600	3.72386500
H	-1.25811700	-0.34869300	3.78240600
H	2.85252400	1.00455400	3.53795400
H	0.52921400	1.23634600	4.46449800
Br	1.83601400	-3.22376800	0.92858300

transition state **TS1**

C	-4.81931700	0.07998100	-3.14585300
C	-5.22432200	0.43149500	-1.85044800
C	-4.27109400	0.64107400	-0.84201700
C	-2.90086400	0.48864400	-1.12159800
C	-2.50300200	0.10733700	-2.41935300
C	-3.45344100	-0.07972200	-3.42862800
P	-1.57178600	0.65142200	0.14461200
C	-2.48924800	0.97474100	1.71042500
C	-3.10234200	2.21344700	1.99109400
C	-3.77757900	2.40459900	3.20390700
C	-3.84566400	1.36456500	4.14520300
C	-3.23041600	0.13373700	3.87403700
C	-2.54929500	-0.05983400	2.66365100
C	-0.93694500	2.32824300	-0.21775900
C	-0.00091000	3.03742400	0.62498500
C	0.26270600	4.31237400	0.02304300
C	-0.48911600	4.39391400	-1.19765700
C	-1.22919100	3.17371500	-1.34914200
Fe	0.77625500	2.79105100	-1.23491300
C	1.56676400	2.55359900	-3.10913600
C	1.34687800	1.28590500	-2.47825000
C	2.10572500	1.25375200	-1.24821600
C	2.78770900	2.52177300	-1.12751500
C	2.45395700	3.31626700	-2.27564900
P	2.02900300	-0.16369100	-0.11349200
C	3.44931400	-1.20491400	-0.65503500
C	4.37784600	-0.79933700	-1.63208900
C	5.42159900	-1.65819400	-2.01003900
C	5.54892300	-2.92021000	-1.41297300
C	4.62253100	-3.33009900	-0.43948800
C	3.57230500	-2.48250600	-0.06896700
Pd	-0.07948800	-1.12817200	0.05911300
C	-1.37233500	-2.78439700	-0.07763100
C	-2.78206700	-2.72355200	-0.17517300
C	-3.37060400	-3.02630100	-1.40031500
C	-2.58739200	-3.43704300	-2.50387700
C	-1.20451400	-3.57154800	-2.36936400
C	-0.57324800	-3.29541100	-1.13453800
C	2.64458400	0.52500000	1.48283800
C	1.74665500	0.59753800	2.56522700
C	2.16050000	1.13468700	3.79247300
C	3.47723700	1.59198600	3.94942100
C	4.38450100	1.49742600	2.88184100
C	3.97335800	0.95999900	1.65457500
Br	-0.48441000	-3.17318700	1.87392800
H	0.94849000	5.06946400	0.40765900
H	0.43222800	2.66031700	1.55208000
H	-1.88841100	2.91111300	-2.17733900
H	-0.47578500	5.22504300	-1.90535500
H	3.41010100	2.83038200	-0.28693700
H	0.70993000	0.47507700	-2.83506900
H	2.78492700	4.34003200	-2.46092200
H	1.11060300	2.89337200	-4.04058900
H	-3.38052500	-2.37430600	0.67053700
H	0.48203600	-3.53714000	-0.97457900
H	-4.45381400	-2.91022200	-1.51263400
H	-0.59854800	-3.94046300	-3.20447000

H	-3.07304400	-3.67228900	-3.45619400
H	0.72542400	0.21881800	2.43259200
H	4.68833200	0.86460400	0.82974000
H	1.45420600	1.18737700	4.62789700
H	5.41833100	1.83661200	3.00932800
H	3.80269400	2.00947300	4.90839000
H	4.28001000	0.18661800	-2.09850800
H	2.83235300	-2.80863300	0.67241900
H	6.13853200	-1.33779100	-2.77404300
H	4.71358100	-4.31823800	0.02425900
H	6.36479600	-3.58802800	-1.70960700
H	-3.04370600	3.02682100	1.25995200
H	-2.04014400	-1.00906800	2.45821100
H	-4.25040200	3.36949100	3.41767400
H	-3.26926800	-0.67758200	4.60872900
H	-4.37191800	1.51866100	5.09349700
H	-4.59500100	0.91587300	0.16667100
H	-1.44190400	-0.07624100	-2.61915900
H	-6.28942400	0.54469800	-1.62026400
H	-3.12970500	-0.37776400	-4.43144400
H	-5.56617300	-0.08283300	-3.93031600

transition state **TS1** with SMD(toluene)

C	-4.58485000	0.08406200	-3.43017800
C	-5.07343400	0.59035200	-2.21636900
C	-4.21239000	0.75658000	-1.12128600
C	-2.85336100	0.40687900	-1.23042600
C	-2.37652400	-0.12804300	-2.44365800
C	-3.23266700	-0.27486800	-3.54194000
P	-1.63346500	0.58203600	0.14455300
C	-2.68570600	0.82932600	1.64078500
C	-3.30220800	2.06098500	1.94561400
C	-4.08586400	2.18952400	3.10081100
C	-4.25910600	1.09453200	3.96224700
C	-3.63827400	-0.12906600	3.67029700
C	-2.84976600	-0.25971800	2.51861800
C	-1.04793600	2.29245100	-0.13101900
C	-0.14057400	2.99117500	0.75155600
C	0.07893200	4.30358400	0.21634700
C	-0.67270800	4.42134900	-1.00139700
C	-1.36881900	3.18526300	-1.21891600
Fe	0.64479300	2.86206700	-1.11571000
C	1.42352900	2.70405300	-3.00418800
C	1.25930000	1.41461800	-2.40087700
C	2.03329300	1.38566100	-1.17903500
C	2.66814300	2.67601600	-1.03739400
C	2.28940700	3.48195200	-2.16292200
P	2.03414100	-0.05527400	-0.07250800
C	3.45880200	-1.04956100	-0.69222700
C	4.22469700	-0.67716800	-1.81294300
C	5.26681600	-1.50419900	-2.26104300
C	5.55629600	-2.70170000	-1.59244900
C	4.79515000	-3.07754500	-0.47255300
C	3.74746000	-2.26220300	-0.02968600
Pd	-0.03682000	-1.12002000	0.09920500
C	-1.19850300	-2.84882000	-0.07484200
C	-2.59197800	-2.91445700	-0.30816400
C	-3.02915000	-3.31951800	-1.56758600
C	-2.11035600	-3.70666000	-2.57152600
C	-0.74099100	-3.71615900	-2.30057000
C	-0.26001700	-3.33610300	-1.02520400
C	2.69092800	0.60020600	1.51981800
C	1.83764400	0.60655500	2.64001300
C	2.29031400	1.10323300	3.87095000
C	3.60113800	1.58728600	3.99209500
C	4.46369900	1.56299500	2.88406400
C	4.01430300	1.06610000	1.65321100
Br	-0.46236700	-3.15840800	1.98383600
H	0.73775500	5.06342300	0.64132800
H	0.30307600	2.58333800	1.66048400
H	-2.01355400	2.94364700	-2.06497100
H	-0.68612300	5.28758800	-1.66611900
H	3.29090100	2.99089200	-0.19924900
H	0.64652500	0.59209000	-2.77203300
H	2.57762200	4.52227800	-2.32770200
H	0.94260100	3.04642200	-3.92247200
H	-3.30103700	-2.58149900	0.45442800
H	0.78752200	-3.49906000	-0.75330300
H	-4.10238900	-3.30358000	-1.78694900
H	-0.02711800	-4.06994600	-3.05301000
H	-2.47968600	-4.02493400	-3.55187300
H	0.82060600	0.20910100	2.53549600

H	4.69525200	1.02919100	0.79575700
H	1.61962600	1.10367100	4.73689000
H	5.49264900	1.92619100	2.98184200
H	3.95696900	1.97262500	4.95379800
H	4.00572600	0.25962700	-2.33557800
H	3.14259400	-2.56488800	0.83355300
H	5.85611100	-1.20736500	-3.13577000
H	5.01450800	-4.01368300	0.05241100
H	6.37066900	-3.34458400	-1.94354200
H	-3.16373300	2.91987200	1.28058400
H	-2.33210200	-1.20221300	2.30751800
H	-4.56084700	3.14960300	3.33123900
H	-3.75494400	-0.98321900	4.34611300
H	-4.86950500	1.19944300	4.86583600
H	-4.60214200	1.15310400	-0.17845900
H	-1.33368400	-0.46119500	-2.50392500
H	-6.13136300	0.85820700	-2.11931000
H	-2.84837700	-0.69395200	-4.47798300
H	-5.26031400	-0.04387000	-4.28309100

transition state **TS1**$^{\mathbf{dtbpf}}$

P	-0.64520200	1.83776000	-0.09848000
C	1.08166000	2.23315000	0.38062300
C	1.60792700	1.70471100	1.62181900
C	2.89344000	2.28850100	1.86819700
C	3.19087800	3.16844800	0.77494200
C	2.08361800	3.13657600	-0.13859800
Fe	2.86965700	1.26781200	0.10239800
C	4.09219600	0.82910300	-1.47746300
C	2.84116600	0.13972000	-1.58012200
C	2.65995500	-0.70057500	-0.41490600
C	3.83325300	-0.49720300	0.40938500
C	4.70377700	0.43928400	-0.24271400
P	1.15458600	-1.73886900	-0.18401000
Pd	-0.90779200	-0.50985100	0.08178200
C	-2.98134100	-0.88491300	0.05172600
C	-4.07688800	0.00101700	-0.01415700
C	-4.89940500	-0.03202300	-1.14155200
C	-4.67728400	-0.96875900	-2.17347000
C	-3.66072700	-1.92101400	-2.03919100
C	-2.84266300	-1.93195000	-0.89086400
Br	-2.43549800	-1.50032900	2.13253600
H	3.54789800	2.06622400	2.71287300
H	1.10402800	0.95821600	2.23516400
H	2.02882800	3.69198600	-1.07459500
H	4.11451500	3.73433700	0.63766000
H	4.02990500	-0.94469800	1.38181400
H	2.13230700	0.24672900	-2.39526500
H	5.64887300	0.81139600	0.15711000
H	4.48703500	1.54758200	-2.19777200
H	-4.25472600	0.71039400	0.79657200
H	-2.13599400	-2.74677400	-0.70817300
H	-5.72088700	0.68855400	-1.22406700
H	-3.52390300	-2.69755500	-2.80003600
H	-5.33105300	-0.98154700	-3.05131900
C	-0.89502100	2.52671000	-1.86868300
C	0.16563600	1.85330400	-2.75703300
C	-2.27051900	2.02486500	-2.35917200
C	-0.79279100	4.05465500	-2.02438000
H	1.18536500	2.19066700	-2.51729200
H	0.11600700	0.75884100	-2.63189900
H	-0.04167500	2.09228800	-3.81706400
H	-3.10999100	2.45256700	-1.79233600
H	-2.39891200	2.30728300	-3.42119700
H	-2.33361800	0.92594300	-2.28116500
H	-0.87901800	4.31236800	-3.09738400
H	-1.59855800	4.58508300	-1.49420300
H	0.17038000	4.44881500	-1.66292700
C	-1.54413400	3.02452300	1.14775000
C	-2.95945500	3.38454700	0.65704400
C	-1.67653900	2.22313300	2.45900600
C	-0.75586800	4.31900400	1.42744800
H	-2.94594000	4.07717500	-0.19921000
H	-3.53315700	2.49175000	0.36973900
H	-3.50221200	3.88681500	1.47983900
H	-0.69032000	1.98081600	2.88677900
H	-2.22362200	2.83151400	3.20420600
H	-2.21744400	1.27467000	2.30792200
H	-1.33897700	4.94559800	2.12972600
H	0.21948000	4.10842900	1.89422200
H	-0.58019800	4.91210900	0.51623300
C	1.55801100	-2.80603500	1.35947600

C	2.87242000	-3.60320500	1.27346100
C	1.57510100	-1.80429700	2.53941800
C	0.39430000	-3.78308400	1.63966700
H	2.79120700	-4.42164000	0.53858000
H	3.73994700	-2.98461300	0.99691800
H	3.08196200	-4.06577900	2.25678500
H	0.59049300	-1.31185100	2.62667000
H	1.76938000	-2.35778200	3.47706200
H	2.34254900	-1.02325600	2.43496600
H	0.47441200	-4.13274600	2.68570600
H	-0.58230000	-3.28381900	1.53121900
H	0.42129700	-4.67092000	0.99286500
C	1.24531900	-2.84672600	-1.77036600
C	0.48058500	-4.16489500	-1.54738900
C	0.51619800	-2.07126800	-2.88981800
C	2.68565200	-3.16324300	-2.21467400
H	1.02931000	-4.85441700	-0.88737000
H	-0.52243200	-3.99641400	-1.12119400
H	0.35107900	-4.66960800	-2.52289900
H	1.06430500	-1.16559700	-3.19109000
H	0.43223100	-2.71737900	-3.78414200
H	-0.49683300	-1.76848600	-2.57260600
H	2.65268500	-3.79083300	-3.12596100
H	3.25114800	-2.24874400	-2.45365900
H	3.24344000	-3.71789900	-1.44400400

transition state **TS1**^{dppb}

C	-1.81313400	1.15161100	-2.07290800
C	0.92405400	-0.42996800	-3.03199100
C	-1.60304000	0.24503000	-3.29890100
C	-0.54458900	-0.86551600	-3.18666400
H	1.19419600	0.35873400	-3.75776400
H	-0.94269600	1.81291100	-1.91134500
H	-1.36146800	0.89322500	-4.16411500
H	-2.68631400	1.80852200	-2.23690000
H	1.57398700	-1.29215000	-3.26089000
H	-2.56695600	-0.24053700	-3.53778100
H	-0.61325400	-1.47494300	-4.10691500
H	-0.80090800	-1.54059100	-2.34690600
P	-2.01985400	0.22909100	-0.45945600
P	1.44031000	0.05828000	-1.29624800
Pd	-0.02672600	-0.59021200	0.38997800
C	-2.70293500	1.54514900	0.62899500
C	-1.90881900	2.69159300	0.85099400
C	-3.91867400	1.40808600	1.32677800
C	-2.33388300	3.68731600	1.73727500
H	-0.94508800	2.79588800	0.34107000
C	-4.33539000	2.40507200	2.22157700
H	-4.53839600	0.51864700	1.17374900
C	-3.54870800	3.54726000	2.42783800
H	-1.70848900	4.57287800	1.89531000
H	-5.28211100	2.28629900	2.75993300
H	-3.87785500	4.32347600	3.12685500
C	-3.44046800	-0.89058000	-0.78516400
C	-3.32253100	-2.22847100	-0.36249400
C	-4.61388500	-0.47224800	-1.44606900
C	-4.36627800	-3.13673900	-0.59387600
H	-2.40307800	-2.54535700	0.14661100
C	-5.65352300	-1.38188300	-1.67831000
H	-4.71868000	0.56904000	-1.77106900
C	-5.53002700	-2.71469800	-1.25231700
H	-4.26785300	-4.17529100	-0.26076200
H	-6.56360200	-1.05208500	-2.19118800
H	-6.34437800	-3.42396300	-1.43569300
C	1.62649400	1.89174000	-1.27381000
C	1.69901500	2.71170300	-2.41611200
C	1.67560400	2.49397700	0.00331900
C	1.80458200	4.10483800	-2.28591800
H	1.67101400	2.26901600	-3.41698300
C	1.80343600	3.88265500	0.13066200
H	1.60250300	1.86375700	0.89750000
C	1.85877200	4.69338100	-1.01414300
H	1.84832800	4.73166600	-3.18318100
H	1.84516200	4.33088100	1.12911200
H	1.94114200	5.78106600	-0.91593200
C	3.18073500	-0.52669900	-1.23814200
C	3.38534300	-1.92505900	-1.21776700
C	4.28468100	0.32875600	-1.05917600
C	4.67078300	-2.45211900	-1.05131100
H	2.52502000	-2.60092500	-1.29328200
C	5.56980200	-0.20441100	-0.87843300
H	4.13813000	1.41329400	-1.05489400

C	5.76836600	-1.59231400	-0.87756800
H	4.81415700	-3.53778900	-1.03526500
H	6.42084700	0.47130200	-0.73896200
H	6.77242800	-2.00488400	-0.73297500
C	1.26228700	-0.97702200	1.99219900
C	0.39257700	-0.23327000	2.83388800
C	2.66191500	-0.78888600	2.02132300
C	0.93139700	0.86183300	3.54490700
H	-0.65415600	-0.52739100	2.96071500
C	3.16309400	0.28513200	2.75633300
H	3.31998500	-1.42172200	1.42165300
C	2.30334200	1.12757500	3.49959700
H	0.26545800	1.47448200	4.16242200
H	4.23990200	0.48613500	2.73215800
H	2.72044800	1.96273800	4.07175200
Br	0.49493600	-3.01440200	1.57722500

intermediate **C**

C	-5.30259700	0.22925200	-2.55972300
C	-5.40653200	0.09987400	-1.16815000
C	-4.28448700	0.29409000	-0.35191400
C	-3.04351800	0.61606500	-0.92914000
C	-2.93627500	0.71925800	-2.33136900
C	-4.06390200	0.53935300	-3.14081200
P	-1.49957000	0.68600100	0.05284600
C	-1.96627100	0.87030500	1.81938200
C	-2.90248900	1.83958200	2.23267100
C	-3.14917600	2.03482900	3.59698300
C	-2.44827900	1.28398800	4.55533600
C	-1.49997200	0.33548200	4.14785000
C	-1.26190400	0.12512600	2.78254700
C	-0.89795400	2.39397700	-0.23880600
C	0.02208700	3.04575600	0.66438100
C	0.31186200	4.35020600	0.14729100
C	-0.42997200	4.52073800	-1.06858600
C	-1.18032000	3.32125800	-1.31100700
Fe	0.80324400	2.89115600	-1.20838700
C	1.47959400	2.58738700	-3.11712100
C	1.23613900	1.35088200	-2.43714300
C	2.05464100	1.31751000	-1.24157900
C	2.82066300	2.54324600	-1.21713200
C	2.45032000	3.32451400	-2.36072500
P	1.96273100	-0.14596600	-0.16835300
C	3.40945400	-1.10745100	-0.76634300
C	4.68237200	-0.97415000	-0.18341900
C	5.77110400	-1.68137900	-0.71244400
C	5.59316000	-2.51824200	-1.82374700
C	4.32312400	-2.65256700	-2.40505900
C	3.23071000	-1.95561200	-1.87542000
Pd	-0.18459300	-1.16767000	-0.14941000
C	-1.99157100	-2.13257900	-0.13759400
C	-2.69340000	-2.46591800	1.03265600
C	-3.94960800	-3.09273400	0.95497500
C	-4.51162100	-3.40070800	-0.29127900
C	-3.80342400	-3.09208000	-1.46310800
C	-2.55269400	-2.46210800	-1.38571100
C	2.40842000	0.28612100	1.55687500
C	2.71025500	1.57395900	2.03550300
C	2.94909700	1.78132300	3.40301200
C	2.89827600	0.70630200	4.30034200
C	2.60999300	-0.58365200	3.82598100
C	2.36061200	-0.79632500	2.46549000
Br	0.85616700	-3.43783900	0.13795900
H	0.99216700	5.07765700	0.59343800
H	0.41228100	2.60529900	1.58192000
H	-1.84192900	3.13996500	-2.15738700
H	-0.41125300	5.39913800	-1.71611200
H	3.55121000	2.83515600	-0.46303100
H	0.53577800	0.56407600	-2.72743000
H	2.82664700	4.32125600	-2.59818400
H	0.98693600	2.92364400	-4.03086800
H	-2.26946000	-2.24475500	2.01662800
H	-2.01993800	-2.21502100	-2.31086600
H	-4.48617400	-3.34358100	1.87770300
H	-4.22788500	-3.33668000	-2.44353400
H	-5.49249300	-3.88527600	-0.35072700
H	2.73982200	2.42118200	1.34798000
H	2.11223300	-1.79969000	2.09403800
H	3.17626800	2.78992400	3.76482200
H	2.56995200	-1.43000500	4.51986500
H	3.08361000	0.87220700	5.36689500
H	4.82032400	-0.32531800	0.68801400

H	2.23102900	-2.08558400	-2.30192400
H	6.76003700	-1.58028700	-0.25242100
H	4.17786700	-3.31806800	-3.26231600
H	6.44408500	-3.07394100	-2.23224500
H	-3.42840000	2.44551800	1.48714500
H	-0.51888800	-0.61014600	2.44948000
H	-3.88330400	2.78259500	3.91541300
H	-0.94012500	-0.24303600	4.88966700
H	-2.63901400	1.44593800	5.62164100
H	-4.36652800	0.15512500	0.72924400
H	-1.96050600	0.90769300	-2.79225800
H	-6.36377600	-0.17033300	-0.71095200
H	-3.97088200	0.62136600	-4.22894600
H	-6.18152000	0.07131600	-3.19340700

intermediate **C**$^{\mathbf{carbonate}}$

C	-0.71450000	4.94066600	-2.92207100
C	-0.57393400	5.23285300	-1.55845400
C	-0.01825600	4.28767500	-0.68433800
C	0.40689100	3.03915500	-1.17145500
C	0.24010200	2.74034400	-2.54008600
C	-0.30680300	3.68954500	-3.41197800
P	1.00571900	1.69247500	-0.07436400
C	1.39926100	2.49013400	1.53762900
C	2.24833500	3.61419700	1.60547100
C	2.60514400	4.14844700	2.84916900
C	2.13542200	3.55344200	4.03215500
C	1.31113600	2.42186900	3.96834300
C	0.94151800	1.89146600	2.72395600
C	2.72778700	1.47833100	-0.67385600
C	3.73522400	0.80268900	0.10925200
C	4.96003200	0.80425700	-0.63540900
C	4.72542600	1.48719000	-1.87544100
C	3.35355800	1.90999200	-1.90314800
Fe	3.45658700	-0.10888800	-1.68712400
C	2.97516000	-0.97726000	-3.47939900
C	1.87283600	-1.00318100	-2.56586500
C	2.28479100	-1.71030200	-1.37060000
C	3.64834300	-2.14149400	-1.58112600
C	4.07127500	-1.67341900	-2.86920700
P	1.08848400	-1.85571600	-0.01119800
C	0.38419600	-3.52250800	-0.32691400
C	1.02685400	-4.68114000	0.14993200
C	0.51539900	-5.94516100	-0.17088400
C	-0.63685100	-6.05561200	-0.96465000
C	-1.27873700	-4.89960100	-1.43181400
C	-0.77449500	-3.62982900	-1.11763300
Pd	-0.39899800	-0.04300800	0.24027300
C	-1.86600400	1.33730200	0.68943900
C	-2.44095200	1.32636100	1.97821800
C	-3.55432500	2.12944500	2.28533300
C	-4.12559900	2.95790100	1.30429800
C	-3.57707200	2.96412800	0.00986800
C	-2.46169900	2.15982200	-0.29062800
C	1.94530800	-2.05619100	1.59775400
C	3.33568400	-2.07078000	1.80969500
C	3.84986500	-2.16088300	3.11305000
C	2.98199400	-2.24407000	4.20981100
C	1.59286700	-2.23812100	4.00142400
C	1.07060200	-2.14108100	2.70665400
H	5.89945400	0.34655000	-0.32045800
H	3.57202800	0.37083600	1.09697800
H	2.86494600	2.45162500	-2.71288000
H	5.45358200	1.63913300	-2.67424900
H	4.26146300	-2.71521400	-0.88666200
H	0.88657400	-0.55354700	-2.70200700
H	5.06649200	-1.80567300	-3.29784500
H	2.98855200	-0.48665800	-4.45407000
H	-2.04094900	0.65470700	2.74475100
H	-2.06596900	2.17104800	-1.31092500
H	-3.97648000	2.10664000	3.29750500
H	-4.00799400	3.60644000	-0.76907300
H	-4.98213600	3.59765000	1.54814300
H	4.02186400	-1.98993400	0.96452400
H	-0.01446500	-2.11845300	2.53320900
H	4.93448100	-2.16570300	3.26726900
H	0.90871000	-2.30292800	4.85434900
H	3.38645000	-2.31308200	5.22543600
H	1.91949500	-4.59370200	0.77911100
H	-1.30802400	-2.72319800	-1.43243800
H	1.01380600	-6.84554700	0.20494800
H	-2.19192200	-4.98208500	-2.03110700

H	-1.03998300	-7.04488700	-1.20812200
H	2.63623100	4.06211000	0.68397500
H	0.31081400	0.99904800	2.65379400
H	3.25945900	5.02557300	2.89789600
H	0.95505000	1.94504100	4.88744800
H	2.42206200	3.96987300	5.00395100
H	0.06135200	4.51250700	0.38290900
H	0.51811900	1.75003600	-2.91625500
H	-0.90899300	6.19896500	-1.16637400
H	-0.42847500	3.44708800	-4.47315000
H	-1.15123700	5.68053400	-3.60126900
O	-1.60934200	-1.45563900	1.18794800
C	-2.69280300	-1.64012800	0.43087500
O	-2.65293300	-1.18414900	-0.78639700
O	-3.71251900	-2.19925500	0.93968900
Cs	-5.38716900	-0.53860200	-0.52922500

intermediate **C**$^{\mathbf{dtbpf}}$

P	-0.82643800	1.70730300	0.27174000
C	0.87456000	2.42039900	0.36291900
C	1.79310300	2.00444800	1.39797400
C	3.01273900	2.74589900	1.28097700
C	2.87536800	3.63580100	0.16711500
C	1.57107200	3.44217100	-0.40118800
Fe	2.59379200	1.70223500	-0.42855000
C	3.06082700	1.33254900	-2.38234400
C	2.01849400	0.47121400	-1.90860200
C	2.51187500	-0.28702400	-0.77641900
C	3.88089200	0.13189100	-0.56841200
C	4.20900300	1.12932900	-1.54754500
P	1.41685100	-1.52320100	0.01574400
Pd	-0.89860000	-0.64615600	0.13980900
C	-2.84881700	-0.53513200	-0.41389200
C	-3.97046000	-0.50101700	0.42955400
C	-5.26699800	-0.56262600	-0.10740900
C	-5.45884400	-0.69877400	-1.49014700
C	-4.34194400	-0.79063500	-2.33298800
C	-3.04559000	-0.70472600	-1.79813700
Br	-1.66146700	-2.97436500	0.84746900
H	3.89514600	2.62852800	1.91204100
H	1.58900300	1.23009800	2.13061100
H	1.20492700	3.96381200	-1.28300700
H	3.63678500	4.31865400	-0.21384000
H	4.55212700	-0.21381200	0.21671900
H	0.99872400	0.41920400	-2.28926800
H	5.15969100	1.66085900	-1.61986400
H	2.97928800	2.04465400	-3.20526400
H	-3.84291200	-0.46413000	1.51545800
H	-2.18568700	-0.77051500	-2.47442900
H	-6.13157400	-0.52415800	0.56550300
H	-4.47408200	-0.92301600	-3.41306000
H	-6.47098100	-0.75456200	-1.90498300
C	-1.66118600	2.84367400	-1.04082800
C	-0.96183100	2.48068900	-2.37213800
C	-3.17198500	2.59986100	-1.25241100
C	-1.50576100	4.34240500	-0.69375500
H	0.12919800	2.61825800	-2.34482500
H	-1.16812200	1.42753300	-2.62810600
H	-1.37179300	3.11408700	-3.18026400
H	-3.77211200	2.84900600	-0.36834900
H	-3.49398800	3.27012200	-2.07181200
H	-3.39700100	1.56991800	-1.54960500
H	-1.78958100	4.93820700	-1.58145300
H	-2.19439200	4.62334300	0.11973800
H	-0.49113000	4.63788600	-0.39486400
C	-1.48767700	2.17529500	2.03654200
C	-3.00797600	2.42579400	2.00693700
C	-1.21081200	0.96460800	2.95485200
C	-0.80321600	3.43015900	2.61687900
H	-3.25158100	3.39508900	1.54341500
H	-3.55460600	1.63227500	1.47810600
H	-3.37380500	2.45712400	3.05004900
H	-0.13378400	0.74988600	3.04541600
H	-1.59385300	1.18751800	3.96839800
H	-1.70052600	0.04799400	2.58168400
H	-1.29267200	3.67603500	3.57798700
H	0.26759300	3.27344200	2.81401700
H	-0.90447600	4.30396900	1.95461400
C	2.39300500	-2.19973900	1.52237000
C	2.66857400	-1.01822500	2.47308800
C	1.50854400	-3.19338600	2.30505100
C	3.72744500	-2.87731700	1.15131800

H	3.27356600	-0.22378200	2.01065100
H	1.71730100	-0.58439700	2.82470400
H	3.20941100	-1.39669100	3.36009400
H	1.27435800	-4.10098800	1.73233500
H	2.05557500	-3.49321500	3.21921500
H	0.55104800	-2.73288300	2.59542900
H	4.25296700	-3.15306700	2.08515500
H	3.57313800	-3.80281300	0.57638100
H	4.39529900	-2.22173500	0.57131300
C	1.44226300	-2.87033900	-1.39150300
C	1.19613400	-4.29473300	-0.85663200
C	0.28618800	-2.52799400	-2.35656100
C	2.77242100	-2.84147700	-2.17344700
H	2.04070600	-4.67173600	-0.25827700
H	0.27105400	-4.34551500	-0.26073400
H	1.08322000	-4.96867500	-1.72662800
H	0.37216800	-1.50714200	-2.76455700
H	0.31705700	-3.23130400	-3.21018700
H	-0.68969900	-2.63248400	-1.85408800
H	2.74806600	-3.65274200	-2.92521900
H	2.91884500	-1.89033400	-2.70904100
H	3.64778800	-3.00973700	-1.52721900

intermediate **C**$^{\mathbf{dppb}}$

C	1.84973000	1.28599300	1.95980500
C	-1.11747500	0.61116200	2.82570300
C	1.45753400	0.55630400	3.25517600
C	0.12311200	-0.20548800	3.23335600
H	-1.10749100	1.62486900	3.26591400
H	1.15519700	2.11774200	1.74489100
H	1.43117700	1.30845200	4.06696400
H	2.85782600	1.72118900	2.08049100
H	-2.02532700	0.11377000	3.20625900
H	2.25775600	-0.15900600	3.51879900
H	-0.05061700	-0.60948400	4.24725100
H	0.19934400	-1.08059700	2.55824400
P	1.86396500	0.18807000	0.45473800
P	-1.41221100	0.73550400	0.98042600
Pd	-0.22165400	-0.79519700	-0.20036800
C	2.58747500	1.26401100	-0.84677800
C	2.99692300	2.59148400	-0.61486700
C	2.67366800	0.73022500	-2.15109500
C	3.49061100	3.37287800	-1.66989300
H	2.92033400	3.03384700	0.38196600
C	3.17070800	1.51490100	-3.19843200
H	2.33546600	-0.29817100	-2.33248000
C	3.57907500	2.83765700	-2.96224000
H	3.80191200	4.40549900	-1.47844400
H	3.23339900	1.09085200	-4.20614800
H	3.96369200	3.45005800	-3.78497300
C	3.20917200	-0.98030300	0.89662400
C	2.87859700	-2.28026200	1.32208000
C	4.55646100	-0.57122900	0.87983000
C	3.88766900	-3.15615600	1.74487500
H	1.83505100	-2.60956500	1.28805200
C	5.56254700	-1.45349600	1.29506800
H	4.81781000	0.43351700	0.52867300
C	5.22836500	-2.74500800	1.73126800
H	3.62578800	-4.16904200	2.06799800
H	6.61014400	-1.13447100	1.27241800
H	6.01646800	-3.43515100	2.05118000
C	-0.97534400	2.43967700	0.44779100
C	-1.17885100	3.57483600	1.25861800
C	-0.45020500	2.60107400	-0.84917700
C	-0.83854800	4.84764800	0.78293800
H	-1.60879600	3.46886700	2.26037700
C	-0.11970100	3.87701500	-1.32614000
H	-0.28799300	1.71431300	-1.47356700
C	-0.30700400	4.99953300	-0.50848500
H	-0.99239800	5.72519200	1.41988200
H	0.30187400	3.98570400	-2.32996900
H	-0.03908200	5.99620300	-0.87485100
C	-3.24220800	0.72250400	0.84973600
C	-3.94499400	-0.39012000	1.35747000
C	-3.93946100	1.71112500	0.13165300
C	-5.32227000	-0.50807800	1.15047400
H	-3.40864400	-1.19068900	1.87658400
C	-5.32243600	1.59100000	-0.06787400
H	-3.40186600	2.56918000	-0.28275400
C	-6.01433600	0.48207100	0.43574500
H	-5.85243300	-1.38725100	1.52947900
H	-5.85704200	2.36515100	-0.62853100

H	-7.09135900	0.38270300	0.26479100
C	-1.96445400	-1.67843500	-0.77491800
C	-2.82093100	-1.07814200	-1.71526700
C	-2.34458200	-2.90088100	-0.19140200
C	-4.04382000	-1.67888400	-2.05366800
H	-2.54826800	-0.12424600	-2.18117000
C	-3.57298000	-3.49717100	-0.52335200
H	-1.67736800	-3.40156900	0.51904300
C	-4.42889100	-2.88549000	-1.45090400
H	-4.70159200	-1.19505700	-2.78499300
H	-3.85610600	-4.44956400	-0.05925700
H	-5.38733100	-3.34987500	-1.70816900
Br	0.97518000	-2.50856100	-1.60733700

intermediate **D**

Si	2.16021900	3.57415400	0.77761000
Br	3.03061200	2.20573700	-2.60226300
N	3.00370100	1.92381200	0.55044200
N	4.06232200	1.88785800	1.23680900
C	4.97586300	0.82841100	1.03920500
C	5.35384500	0.42218500	-0.26000800
C	5.60826700	0.28418300	2.17525500
C	6.35225500	-0.54996600	-0.40590200
H	4.89835300	0.90505500	-1.13241200
C	6.56943100	-0.71814400	2.01367600
H	5.31854600	0.64614500	3.16617700
C	6.94886300	-1.13128800	0.72391100
H	6.66052200	-0.85397900	-1.41132800
H	7.03894000	-1.16901100	2.89400200
H	7.72259800	-1.89656300	0.60179600
C	1.79044500	-0.77095800	-2.36044400
C	0.89404400	-0.37954100	-3.37570700
C	2.38351800	-2.04589100	-2.44833000
C	0.56339400	-1.25758900	-4.41913100
H	0.46701800	0.62702600	-3.37054700
C	2.05851800	-2.92361700	-3.49801300
H	3.09572900	-2.37902700	-1.68748400
C	1.13296100	-2.53988600	-4.47806100
H	-0.13557600	-0.93188200	-5.19889100
H	2.52549300	-3.91457900	-3.53782000
H	0.86959200	-3.22578400	-5.29063300
Pd	2.29231300	0.49664900	-0.86563900
P	1.30791100	-0.96771100	0.57510300
C	-0.34923100	-1.63690800	0.22680900
C	-1.71105600	0.92363700	-1.44650900
C	-1.43890600	-1.82682400	1.16796300
C	-0.71962800	-2.31135300	-0.99989900
Fe	-2.06980600	-0.86774500	-0.51913000
C	-2.48757900	0.05944200	-2.28606200
C	-2.43719400	1.11821100	-0.22583300
C	-2.45841100	-2.59289000	0.51653400
H	-1.47705600	-1.45247000	2.19042200
C	-2.01290100	-2.89192900	-0.81222900
H	-0.12405300	-2.35402900	-1.90953900
C	-3.69471200	-0.28071600	-1.58717500
C	-3.67191200	0.37162800	-0.29768800
H	-2.18057400	-0.32345100	-3.26040200
H	-2.13475100	1.74020500	0.61770100
H	-3.42536200	-2.86383700	0.94025300
H	-2.58276500	-3.42673000	-1.57380500
H	-4.47393100	-0.95633200	-1.94146900
C	1.10913600	-0.21630200	2.24471100
C	2.07629600	-0.41748100	3.24747000
C	-0.00363200	0.60457000	2.51360900
C	1.91792100	0.17900600	4.50602400
H	2.94607300	-1.05089900	3.04779500
C	-0.17113000	1.17764600	3.78052100
H	-0.75268800	0.75994800	1.73350800
C	0.79020300	0.96661100	4.78080100
H	2.67469700	0.01535400	5.28065200
H	-1.05419900	1.79282200	3.98276300
H	0.66344100	1.41956800	5.76946500
C	2.23699400	-2.51559700	0.94792200
C	1.56405600	-3.67823900	1.37332000
C	3.63847900	-2.52892500	0.83327800
C	2.29164000	-4.83352100	1.68604600
H	0.47330900	-3.68029000	1.45462400
C	4.36247800	-3.68634800	1.15044100
H	4.15718800	-1.63422600	0.47978800
C	3.69084500	-4.84041500	1.57696400
H	1.76173800	-5.73456000	2.01289500
H	5.45255100	-3.68157800	1.05358300

H	4.25512000	-5.74767300	1.81793900
C	0.47825700	3.42458000	-0.03204000
H	0.58234200	3.39220400	-1.12983900
H	-0.01176200	2.49391700	0.29732600
H	-0.17367400	4.27379600	0.24284700
C	3.27699600	4.86059200	-0.00998900
H	4.28649500	4.80281600	0.43465200
H	3.35829500	4.68891800	-1.09617900
H	2.87614400	5.87537800	0.17167500
C	2.06125000	3.84287800	2.63643600
H	1.47201300	3.05640200	3.13505400
H	3.07816800	3.83852700	3.06571600
H	1.59356800	4.82045500	2.85652100
H	-0.71942600	1.31733500	-1.67005100
P	-4.87961100	0.37572500	1.07452200
C	-6.02133700	1.72497900	0.51534400
C	-7.20128400	1.93565300	1.26090100
C	-5.74284300	2.57445300	-0.57259900
C	-8.09096600	2.95883600	0.91367300
H	-7.42635700	1.28515500	2.11465900
C	-6.63113100	3.60694700	-0.91184200
H	-4.83137100	2.42497500	-1.16019400
C	-7.80697700	3.80033300	-0.17415500
H	-9.00717200	3.10448800	1.49639300
H	-6.40291700	4.25919100	-1.76200800
H	-8.50005700	4.60458100	-0.44308600
C	-5.91924300	-1.10299000	0.66168800
C	-5.86795900	-2.21392000	1.52635400
C	-6.76430300	-1.15442300	-0.46592700
C	-6.61531600	-3.36915200	1.25179700
H	-5.24134300	-2.16463700	2.42461300
C	-7.51121400	-2.30777000	-0.74022900
H	-6.84173500	-0.28141900	-1.12313800
C	-7.43318100	-3.41932300	0.11398600
H	-6.56560600	-4.22669800	1.93159300
H	-8.16139600	-2.33859100	-1.62141900
H	-8.02020600	-4.31863500	-0.10104500

intermediate **D**[1]

Si	2.38667800	2.96549900	1.74860800
N	3.01609100	1.41568200	0.91388700
N	4.06829400	1.02176100	1.49620300
C	4.82106200	-0.07937100	1.04452100
C	4.86095900	-0.56443600	-0.27998300
C	5.63644600	-0.66935900	2.03854000
C	5.68567600	-1.65147700	-0.58745900
H	4.25434000	-0.08311400	-1.05945700
C	6.42463000	-1.77818500	1.72888100
H	5.60324000	-0.24749100	3.04723100
C	6.44979000	-2.27171000	0.41201800
H	5.72594300	-2.02156100	-1.61568600
H	7.03214400	-2.25320700	2.50590300
H	7.07821100	-3.13274800	0.16218000
Pd	2.10407900	0.69774400	-0.75289600
P	1.28588200	-1.33608800	0.24855300
C	-0.42039400	-1.91420000	-0.03550800
C	-1.58274700	1.06911700	-0.58999100
C	-1.43973700	-2.28304000	0.92671100
C	-0.93116700	-2.22891800	-1.35300700
Fe	-2.11735200	-0.89074100	-0.40218500
C	-2.26750600	0.61823200	-1.76524900
C	-2.45246800	0.87878500	0.53663400
C	-2.56166000	-2.79903100	0.20147200
H	-1.37073000	-2.18772500	2.00976200
C	-2.24903400	-2.76800400	-1.19816700
H	-0.40191000	-2.03209400	-2.28687000
C	-3.56633800	0.15153900	-1.37305900
C	-3.69397600	0.30618700	0.05966400
H	-1.83133200	0.58382800	-2.76488000
H	-2.23957600	1.12803100	1.57804700
H	-3.50216700	-3.13100900	0.64013300
H	-2.91668600	-3.06351800	-2.00905600
H	-4.31784100	-0.28790100	-2.02954900
C	1.38335400	-1.19354700	2.07583300
C	2.47655500	-1.68141300	2.81572900
C	0.38577700	-0.44714000	2.73887100
C	2.55159000	-1.45535100	4.19784600
H	3.27184100	-2.23936800	2.31389000
C	0.45519300	-0.23846200	4.12118300
H	-0.45466500	-0.04944000	2.16247400
C	1.53883900	-0.74473900	4.85671400
H	3.40769900	-1.84198700	4.76112300

H	-0.33617400	0.32749200	4.62391900
H	1.59653400	-0.57730200	5.93716600
C	2.19005900	-2.87779500	-0.19294800
C	2.15994000	-4.02239700	0.62903900
C	2.81595500	-2.94183100	-1.45163200
C	2.79083100	-5.20164600	0.21311100
H	1.63718300	-3.99420900	1.59061900
C	3.42786300	-4.13124400	-1.87125200
H	2.79621700	-2.06241300	-2.10636700
C	3.42862800	-5.25721900	-1.03633800
H	2.77331200	-6.08457700	0.86112200
H	3.90533200	-4.17514200	-2.85606900
H	3.91408300	-6.18345200	-1.36233000
C	0.56106900	3.13404600	1.36602600
H	0.38786200	3.36416400	0.30269300
H	0.01327000	2.21361000	1.62387800
H	0.13988000	3.96027700	1.96895200
C	3.39641000	4.41638500	1.11824100
H	4.47561000	4.19135600	1.17495500
H	3.14801500	4.67741300	0.07667400
H	3.19480500	5.29397400	1.76164400
C	2.68346800	2.70077400	3.58623100
H	2.21701000	1.76453300	3.93571700
H	3.76419900	2.64808300	3.80028600
H	2.25155400	3.54079600	4.16150300
H	-0.54796900	1.41769900	-0.56783400
P	-5.09159800	-0.09517500	1.16728400
C	-6.06614500	1.47946600	1.06716600
C	-7.30862500	1.52575800	1.73525000
C	-5.60847100	2.63314800	0.40177500
C	-8.08408800	2.69102800	1.72010500
H	-7.67221000	0.63630700	2.26393000
C	-6.38219100	3.80420600	0.39785400
H	-4.64636600	2.61263500	-0.12019200
C	-7.62128500	3.83635800	1.05214900
H	-9.05062900	2.70874000	2.23569600
H	-6.01489400	4.69378400	-0.12582200
H	-8.22484400	4.75032000	1.04465900
C	-6.11724500	-1.16796400	0.05351500
C	-6.20832600	-2.54226700	0.34721500
C	-6.80785800	-0.66403500	-1.06790300
C	-6.94091000	-3.40490200	-0.48156200
H	-5.70927800	-2.93387000	1.24115900
C	-7.54286700	-1.52431800	-1.89432600
H	-6.77002100	0.40840100	-1.28777300
C	-7.60515700	-2.89723400	-1.60710800
H	-7.00049600	-4.47229400	-0.24249500
H	-8.07199600	-1.12198300	-2.76518600
H	-8.18096600	-3.56773300	-2.25408900
C	2.73655600	2.43326600	-1.63955500
C	1.78746300	3.35297100	-2.12627200
C	4.10302600	2.74880600	-1.75330200
C	2.19772300	4.57311900	-2.68779600
H	0.71936400	3.11266700	-2.09018500
C	4.51466700	3.95964900	-2.33595800
H	4.86876300	2.05809000	-1.38206300
C	3.56302200	4.88083400	-2.79483000
H	1.44471200	5.27947800	-3.05645200
H	5.58420500	4.18541000	-2.41939300
H	3.88189300	5.82960100	-3.23993300
Br	1.23167000	0.04444900	-3.01591000

intermediate **D**^{2}

Br	-3.73088300	-0.86836500	-3.11661500
N	-3.56718300	-1.52834800	0.01677400
C	-1.49881400	1.29680100	-2.20471700
C	-0.51593000	0.83988400	-3.10580500
C	-1.76400100	2.67743300	-2.13852200
C	0.21515700	1.74672400	-3.88891900
H	-0.32541200	-0.23271100	-3.20913500
C	-1.03443500	3.58544600	-2.92582500
H	-2.53695600	3.05984800	-1.46476200
C	-0.03175300	3.12575900	-3.79136500
H	0.97955100	1.37281000	-4.58016200
H	-1.24969800	4.65782600	-2.85308400
H	0.54349600	3.83410600	-4.39747200
Pd	-2.47705900	-0.05663200	-1.07234600
P	-1.42859900	0.89805500	0.71308700
C	0.34412000	1.28434900	0.75933800
C	1.77962100	-1.70683700	0.12358200
C	1.15596200	1.23613700	1.95980700
C	1.11539100	1.95803000	-0.26355200

Fe	2.08806600	0.30157700	0.41077700
C	2.14954700	-1.06647600	-1.10760200
C	2.81730500	-1.47431200	1.08267600
C	2.40509400	1.87353800	1.67281800
H	0.86069300	0.78471200	2.90741800
C	2.37887200	2.31880800	0.30672900
H	0.79303000	2.14765300	-1.28605100
C	3.42723300	-0.44692900	-0.91596300
C	3.85534900	-0.69569800	0.44316900
H	1.55008000	-1.02487900	-2.01662600
H	2.82468500	-1.80858600	2.12156400
H	3.24748200	1.95182600	2.36225000
H	3.19975200	2.80189000	-0.22493700
H	3.96723200	0.15154500	-1.65008300
C	-1.63243600	-0.07028400	2.26644300
C	-2.15843100	0.48080400	3.44930500
C	-1.23703400	-1.42176000	2.25249900
C	-2.28598200	-0.31327300	4.59832100
H	-2.47371500	1.52791300	3.46973800
C	-1.35715900	-2.21116000	3.40170700
H	-0.84071800	-1.85024900	1.32845000
C	-1.88604400	-1.65718700	4.57859900
H	-2.70197800	0.12241000	5.51287200
H	-1.04997300	-3.26152500	3.37584200
H	-1.98869500	-2.27452900	5.47720000
C	-2.17327800	2.52796400	1.13381200
C	-1.39100600	3.66579600	1.40467400
C	-3.57818800	2.62746800	1.16463400
C	-2.01285300	4.88708700	1.70318900
H	-0.30018500	3.60104500	1.37341300
C	-4.19362300	3.84566900	1.47413400
H	-4.18860900	1.74864800	0.93992500
C	-3.41139400	4.98045000	1.74029800
H	-1.39811000	5.77070700	1.90495400
H	-5.28668400	3.90794000	1.49732000
H	-3.89157000	5.93724400	1.97142200
H	0.86180100	-2.26406800	0.30130700
P	5.37718100	-0.16019900	1.30895400
C	6.54757000	-1.49880300	0.78881700
C	7.90002300	-1.37149800	1.17237600
C	6.14708100	-2.64665900	0.07821800

C	8.83309300	-2.35850100	0.83409700
H	8.22300300	-0.48626900	1.73335100
C	7.08219000	-3.64067900	-0.24948800
H	5.10147200	-2.76036800	-0.22535700
C	8.42586500	-3.49880700	0.12286900
H	9.88109200	-2.24137600	1.13092600
H	6.75731400	-4.52764400	-0.80455500
H	9.15443500	-4.27405500	-0.13724700
C	5.94431900	1.20956300	0.19317200
C	5.84176100	2.53456200	0.65976700
C	6.46886700	0.97948800	-1.09544800
C	6.21677000	3.61173800	-0.15681500
H	5.46674800	2.72131800	1.67224500
C	6.84618000	2.05495700	-1.91049700
H	6.58429400	-0.04848700	-1.45584200
C	6.71447000	3.37321500	-1.44588400
H	6.12811200	4.63733900	0.21757100
H	7.24739100	1.86436200	-2.91187500
H	7.01075100	4.21229800	-2.08434900
N	-3.39394400	-2.76137700	0.12235900
C	-4.66138500	-0.98591700	0.78608300
C	-4.82186500	-1.33364000	2.13522300
C	-5.53437500	-0.08884800	0.14704800
C	-5.85538800	-0.73944200	2.86873100
H	-4.11858400	-2.03510800	2.59156500
C	-6.59143600	0.46217700	0.88286700
H	-5.38166800	0.13386300	-0.91498200
C	-6.74230400	0.15284700	2.24473600
H	-5.96898300	-0.97419700	3.93219500
H	-7.29491800	1.14172800	0.39099800
H	-7.55508000	0.60686600	2.82131700
Si	-2.21271700	-3.75416100	-0.84146400
C	-0.92193700	-2.84123000	-1.86275700
H	-1.32356900	-2.60327800	-2.86215100
H	-0.65613200	-1.87768100	-1.38799700
H	-0.00341700	-3.44883400	-1.96451600
C	-3.37383400	-4.77879500	-1.90679100
H	-4.12482600	-5.30730200	-1.29479700
H	-3.89463200	-4.11401700	-2.61827600
H	-2.79707400	-5.52673300	-2.48202100
C	-1.39132900	-4.81407000	0.49187700

H	-2.13792000	-5.19029400	1.21304700
H	-0.89630700	-5.68425800	0.02230400
H	-0.61763300	-4.25942300	1.05228800

intermediate **E**

Br	1.65715400	-0.54659900	3.79794100
N	1.80218900	-2.27874200	1.09015500
N	2.79640000	-2.96909900	0.82637200
C	4.08673400	-2.35786400	0.63327000
C	4.62177900	-1.44196300	1.56279900
C	4.83027900	-2.74370000	-0.49469800
C	5.90334000	-0.91463200	1.34542000
H	4.03135600	-1.17092900	2.44683000
C	6.09222700	-2.17833400	-0.72417800
H	4.38957700	-3.46339200	-1.19273000
C	6.63590000	-1.26834300	0.19960000
H	6.32431200	-0.20964000	2.07136800
H	6.65346000	-2.44701000	-1.62680500
H	7.62920600	-0.83793600	0.02691900
C	1.81088100	1.85383400	1.58463100
C	0.69384300	2.54783000	2.10550400
C	2.98410800	2.61514000	1.38984400
C	0.73180700	3.92540000	2.37936500
H	-0.23349600	2.00130200	2.31549600
C	3.03991600	3.99191600	1.66588000
H	3.88857000	2.12336900	1.01208200
C	1.90655200	4.65986200	2.15225900
H	-0.15964200	4.42671100	2.77983500
H	3.97302600	4.54487900	1.49442100
H	1.94003500	5.73613000	2.36244200
Pd	1.84257700	-0.21197800	1.25995800
P	1.78238600	0.09533000	-0.95545200
C	0.14374900	0.66524100	-1.51017500
C	-1.30126300	-1.60400000	0.52601200
C	-0.69663700	0.08736200	-2.53295100
C	-0.58126200	1.76445800	-0.91467300
Fe	-1.62828000	0.03554300	-0.70946800
C	-1.62540800	-0.44379000	1.30001800
C	-2.36785700	-1.83399700	-0.40221800
C	-1.92270800	0.82831700	-2.56796400
H	-0.44215700	-0.77354000	-3.15121800
C	-1.85080700	1.86593700	-1.57445300
H	-0.21498600	2.37709900	-0.08990400
C	-2.89997700	0.04758900	0.86824000
C	-3.38142400	-0.81699300	-0.19195000
H	-0.97255900	-0.02014200	2.06694100
H	-2.41953500	-2.62808000	-1.14996500
H	-2.77445200	0.60856700	-3.21392500
H	-2.64001200	2.57950000	-1.33310600
H	-3.40613300	0.93805700	1.24227300
C	2.05475500	-1.33052200	-2.10395700
C	3.00945400	-1.31010400	-3.13927000
C	1.28583300	-2.49730900	-1.91043400
C	3.19928400	-2.43508200	-3.95404400
H	3.61284500	-0.41358600	-3.30593100
C	1.47266500	-3.61670000	-2.72949100
H	0.55292700	-2.52895000	-1.10268500
C	2.43547200	-3.59336200	-3.75035100
H	3.95115200	-2.40414900	-4.75099200
H	0.87352400	-4.51704000	-2.55570000
H	2.58938200	-4.47449600	-4.38358300
C	2.95692800	1.34510700	-1.60951200
C	2.54993100	2.51083000	-2.27934300
C	4.32685900	1.13400900	-1.35041100
C	3.50386500	3.45542500	-2.68668400
H	1.48613100	2.68526500	-2.46759100
C	5.27664100	2.06964700	-1.77492600
H	4.64327100	0.24107500	-0.80135600
C	4.86667300	3.23683400	-2.43953600
H	3.17813500	4.36962300	-3.19571100
H	6.33765800	1.89222600	-1.56705800
H	5.60815200	3.97929900	-2.75599000
H	-0.37280900	-2.17416500	0.65699400
P	-4.92893400	-0.74634300	-1.15102600
C	-6.12351700	-1.49313000	0.05727600
C	-7.50363500	-1.43243700	-0.23368100
C	-5.70475800	-2.17287200	1.21768400
C	-8.44214000	-2.01836200	0.62401600
H	-7.84248100	-0.91018300	-1.13685500
C	-6.64629000	-2.76937800	2.07084100
H	-4.63753000	-2.22520600	1.45673100
C	-8.01553200	-2.69200400	1.78042000

H	-9.51081000	-1.95301200	0.38891600
H	-6.30415600	-3.29057300	2.97198900
H	-8.74875500	-3.15425300	2.45073400
C	-5.39605600	1.04216600	-0.96758300
C	-5.30622200	1.87568000	-2.09912800
C	-5.82464000	1.60176300	0.25392800
C	-5.59717800	3.24471300	-2.00749700
H	-5.00034700	1.44459700	-3.05869400
C	-6.11738200	2.96878500	0.34691100
H	-5.92767700	0.95871100	1.13444800
C	-5.99779600	3.79522700	-0.78166900
H	-5.51232200	3.88127400	-2.89528200
H	-6.43745000	3.39254000	1.30539400
H	-6.22285500	4.86477100	-0.70593000

intermediate (*Z*)-**F**

C	-3.20808300	-0.57623800	4.21018600
C	-3.49865300	-1.76014700	3.51321500
C	-2.90758000	-2.00738000	2.26680000
C	-2.01225600	-1.07122100	1.71271200
C	-1.73766700	0.11948500	2.40922400
C	-2.33019300	0.36580700	3.65493600
P	-1.18131500	-1.34788400	0.09579600
C	-2.49837200	-2.13292000	-0.92269800
C	-2.19237800	-3.18147000	-1.81279100
C	-3.18205000	-3.70568200	-2.65539100
C	-4.48600500	-3.19006700	-2.61880500
C	-4.79546400	-2.14690500	-1.73393500
C	-3.81019500	-1.61609100	-0.89303800
C	-0.09087500	-2.76856600	0.45267100
C	0.97473000	-3.20772100	-0.42335900
C	1.66026400	-4.29392300	0.21438300
C	1.03478200	-4.53152300	1.48397900
C	-0.04315600	-3.59760100	1.63572200
Fe	1.70806000	-2.60146600	1.36084400
C	2.34015100	-1.86079600	3.16156200
C	1.74560100	-0.83771500	2.35246500
C	2.48496400	-0.75936000	1.11007000
C	3.53992600	-1.74588500	1.17103500
C	3.44341400	-2.42071800	2.43335900
P	2.01366100	0.35044900	-0.24777700
C	2.97573200	1.87207200	0.12721000
C	3.18312400	2.81513300	-0.90183100
C	3.79846000	4.03958500	-0.61646800
C	4.21407500	4.33621900	0.69077800
C	4.01681400	3.39877700	1.71465800
C	3.39935600	2.17122900	1.43733100
Pd	-0.28680800	0.68040300	-0.69332500
C	-2.24698500	1.19426700	-1.13937400
C	-2.71640200	0.96570500	-2.44948400
C	-4.03532700	1.27578400	-2.81662600
C	-4.92005800	1.82060300	-1.87360400
C	-4.46648800	2.06604500	-0.56921200
C	-3.14255600	1.76001700	-0.20900000
C	2.87968500	-0.31546800	-1.72429500
C	4.28541100	-0.35932900	-1.81211100
C	4.90070300	-0.88063300	-2.95758000
C	4.11902600	-1.34678600	-4.02710300
C	2.72011300	-1.27896100	-3.95625600
C	2.10140200	-0.75962900	-2.81012800
H	2.53008600	-4.81923100	-0.18367400
H	1.23372100	-2.76081700	-1.38384200
H	-0.69154000	-3.49577400	2.50614700
H	1.34768700	-5.26990900	2.22458000
H	4.25148800	-1.96603200	0.37507700
H	0.86575200	-0.24428000	2.60260200
H	4.07753100	-3.24511500	2.76466000
H	1.98858700	-2.18041700	4.14392300
H	-2.04655900	0.53020300	-3.20062100
H	-2.81678300	1.97655800	0.81337200
H	-4.37481200	1.08342400	-3.84135900
H	-5.14197400	2.50422700	0.17548700
H	-5.95223800	2.05835200	-2.15523700
H	4.89429300	0.03121900	-0.98952800
H	1.00893700	-0.67491000	-2.74743000
H	5.99363100	-0.91520700	-3.02131700
H	2.10841100	-1.61930900	-4.79830800
H	4.60355900	-1.74872900	-4.92335900
H	2.84467400	2.59394400	-1.91859900
H	3.24663700	1.44266100	2.24000100
H	3.94662300	4.76888400	-1.41964100
H	4.34479600	3.62201700	2.73564700

H	4.69139500	5.29705600	0.91047100
H	-1.18056200	-3.59710300	-1.84295000
H	-4.05745200	-0.78650300	-0.22412000
H	-2.93222800	-4.52365700	-3.33990300
H	-5.80668800	-1.72835200	-1.70403800
H	-5.25782600	-3.59961000	-3.27930500
H	-3.14949600	-2.92173700	1.71460300
H	-1.07732000	0.86091000	1.94234700
H	-4.19494200	-2.49041000	3.93946900
H	-2.11806700	1.30226600	4.18139600
H	-3.67794600	-0.38383800	5.18072000
N	0.21217400	2.37231500	-1.75004800
N	-0.04646400	3.53354400	-1.44728100
C	-0.66429500	3.89110800	-0.20621500
C	-1.70142600	4.83650000	-0.22561500
C	-0.21149200	3.34753500	1.00924600
C	-2.34165900	5.18045500	0.97003900
H	-2.01740300	5.25452900	-1.18592100
C	-0.82755600	3.73452900	2.20741600
H	0.63306200	2.65318700	1.00299600
C	-1.90688800	4.63116900	2.18895600
H	-3.18408300	5.88000100	0.95428700
H	-0.45891000	3.33728100	3.16017800
H	-2.39764700	4.91857400	3.12504400

intermediate (*Z*)-**F**$^{\mathbf{dtbpf}}$

P	-0.27417700	1.90169900	0.18055900
C	-2.11309100	1.92238600	-0.04270000
C	-2.66958100	1.31481100	-1.23816200
C	-4.08832800	1.50249700	-1.25887600
C	-4.44352200	2.23351000	-0.07982700
C	-3.24450300	2.50436900	0.66016700
Fe	-3.45779800	0.49999700	0.40903000
C	-3.80033800	-0.33577300	2.24391400
C	-2.48848200	-0.62188700	1.74766600
C	-2.59106500	-1.30838900	0.47191100
C	-4.01350600	-1.44614300	0.21236700
C	-4.74256600	-0.83788200	1.28863400
P	-1.02361800	-1.85976300	-0.32978100
Pd	0.80293900	-0.22424900	-0.39717200
C	2.55484900	0.79981400	-0.80738900
C	2.88471800	0.97693600	-2.16965900
C	4.06690000	1.62403600	-2.56400400
C	4.96502700	2.10322400	-1.59851700
C	4.67411500	1.90745600	-0.24108300
C	3.48742000	1.26171100	0.14447000
H	-4.77230400	1.12523800	-2.02076200
H	-2.09251100	0.75341700	-1.96681800
H	-3.21919400	3.04495800	1.60182900
H	-5.45240200	2.51884300	0.22416300
H	-4.47507900	-1.90288900	-0.65885400
H	-1.55077700	-0.33908900	2.21857700
H	-5.82934300	-0.75566100	1.34958000
H	-4.03356300	0.19809500	3.16659100
H	2.21222100	0.59385900	-2.94683400
H	3.31265000	1.10847100	1.21427000
H	4.28817600	1.74997200	-3.63069500
H	5.37544700	2.25285500	0.52789800
H	5.88781800	2.61028200	-1.90153700
N	1.85121500	-1.78589000	-1.26389400
N	2.92109300	-2.27833800	-0.90888800
C	3.58689800	-1.93211900	0.31087100
C	4.99057800	-1.91169000	0.30288800
C	2.87509300	-1.68257300	1.49631800
C	5.68105200	-1.57153100	1.47091500
H	5.51413300	-2.12841600	-0.63310500
C	3.57345600	-1.38893300	2.67393100
H	1.77917200	-1.69412700	1.46407900
C	4.97515200	-1.31510900	2.65936500
H	6.77444300	-1.51069300	1.46002700
H	3.02364800	-1.20940400	3.60457500
H	5.52039300	-1.07142800	3.57758600
C	-1.34645000	-2.29191500	-2.20357200
C	-2.84153100	-2.39432500	-2.54631200
C	-0.69353100	-1.19413800	-3.07851600
C	-0.63866500	-3.59921500	-2.61763800
H	-2.93958000	-2.65151900	-3.61770100
H	-3.34879600	-3.18281400	-1.96610000
H	-3.36294900	-1.43776100	-2.38226900
H	0.40141500	-1.22806200	-2.97351500
H	-0.95788400	-1.39161100	-4.13488000
H	-1.02567500	-0.17534200	-2.83502500

H	-0.78454500	-3.72561200	-3.70691500
H	0.44407300	-3.52850300	-2.42910100
H	-1.04978100	-4.49304300	-2.12467500
C	-0.83889000	-3.51186300	0.67534600
C	0.46667500	-4.26789800	0.35687700
C	-0.76923200	-3.12885200	2.17265400
C	-2.06336900	-4.42517100	0.46952800
H	0.55780300	-4.57402100	-0.69150900
H	1.35008800	-3.66845000	0.61610000
H	0.49031700	-5.18128800	0.98047500
H	-1.73944000	-2.78765800	2.56386500
H	-0.46747900	-4.02398500	2.74673700
H	-0.01677400	-2.34309900	2.35995500
H	-1.97822100	-5.29977300	1.14212100
H	-3.00214600	-3.90126400	0.71580300
H	-2.13570200	-4.80741000	-0.56097400
C	0.08049400	3.45744600	-0.94147400
C	1.52911200	3.99259000	-0.90222600
C	-0.18410800	2.96677200	-2.38339400
C	-0.88282400	4.61172300	-0.60191400
H	1.73305100	4.59179200	-0.00522300
H	2.28007300	3.19669300	-0.97932200
H	1.65586900	4.65984400	-1.77478400
H	-1.24390500	2.72295700	-2.55258800
H	0.08924200	3.77436600	-3.08701600
H	0.43306200	2.08388500	-2.61864900
H	-0.65435300	5.47151900	-1.25990700
H	-1.93740500	4.33537500	-0.75899100
H	-0.76372400	4.95313600	0.44149200
C	0.13707600	2.30269200	2.04084900
C	0.74411100	1.02149500	2.65151000
C	1.21131600	3.40019200	2.16160100
C	-1.08005000	2.72450400	2.87867500
H	0.05162000	0.16667100	2.63125700
H	1.65077800	0.71408100	2.11114700
H	1.01107700	1.21757800	3.70732200
H	0.83808800	4.38404700	1.83687600
H	1.49425600	3.48555500	3.22779500
H	2.11832500	3.15280400	1.59026600
H	-0.75424400	2.85774700	3.92759200
H	-1.49873900	3.68543200	2.53721500
H	-1.87488000	1.96298100	2.86433200

intermediate (*Z*)-**F**$^{\mathbf{dppb}}$

C	-3.48071000	1.59065800	3.99742900
C	-3.99674900	0.29680100	3.81880300
C	-3.40201800	-0.57698600	2.89940100
C	-2.28439300	-0.16137900	2.14746700
C	-1.77315000	1.13641000	2.33252400
C	-2.36801000	2.00898700	3.25406300
P	-1.43985800	-1.27087000	0.94603900
C	-2.79276800	-1.91929500	-0.11212600
C	-2.53265400	-3.05263700	-0.90989900
C	-3.47285500	-3.48902800	-1.85101300
C	-4.67655700	-2.78837300	-2.01912000
C	-4.93338000	-1.65190700	-1.23999100
C	-4.00016200	-1.21804300	-0.28998600
P	2.22667400	-0.63316900	0.72058300
C	3.53023400	0.64484700	0.51294700
C	4.24802700	0.70831100	-0.70013700
C	5.12287300	1.77351200	-0.94790500
C	5.29594800	2.78637800	0.00590100
C	4.58302100	2.73356900	1.21259600
C	3.70073600	1.67483700	1.46175600
Pd	0.15837300	-0.05408100	-0.29677700
C	-1.47736800	0.58676600	-1.40374100
C	-1.77323600	-0.09583000	-2.60242500
C	-2.88316200	0.25351100	-3.38649300
C	-3.72595000	1.30289800	-2.98824900
C	-3.43933500	2.00022900	-1.80679200
C	-2.32646800	1.64528900	-1.02465900
C	2.93454400	-2.07732900	-0.17807500
C	4.19883200	-2.61148200	0.14159400
C	4.69650200	-3.71329000	-0.56531700
C	3.93935300	-4.28621900	-1.60023800
C	2.68588100	-3.75192300	-1.93057900
C	2.18488800	-2.64956400	-1.22345200
H	-1.13252000	-0.92231600	-2.93256900
H	-2.12485200	2.22015500	-0.11503800
H	-3.09267500	-0.29887700	-4.31019500
H	-4.08013100	2.83171200	-1.48869600
H	-4.59567400	1.57589700	-3.59675700

H	4.80166500	-2.15550700	0.93527500
H	1.20969800	-2.21249500	-1.47337900
H	5.67998600	-4.12406600	-0.31279300
H	2.09761900	-4.18902700	-2.74426200
H	4.33204500	-5.14667400	-2.15257600
H	4.10521000	-0.06871600	-1.45604100
H	3.13237800	1.66574600	2.39849800
H	5.66643600	1.81422600	-1.89752400
H	4.70614500	3.52396900	1.96059400
H	5.97932300	3.61867000	-0.19238900
H	-1.57759500	-3.58344000	-0.81611900
H	-4.19668800	-0.31300600	0.29061900
H	-3.25855700	-4.37084800	-2.46445300
H	-5.85988300	-1.08600800	-1.38071100
H	-5.40620600	-3.12089600	-2.76497100
H	-3.81707900	-1.58086000	2.75484300
H	-0.90937700	1.44999100	1.73338300
H	-4.86843500	-0.03049300	4.39571000
H	-1.96612500	3.01954000	3.38275700
H	-3.95031200	2.27234600	4.71469300
N	1.29859800	0.78999600	-1.79391900
N	1.33947400	1.97468700	-2.11124700
C	0.65020900	2.99483300	-1.38111600
C	-0.02925700	3.98767100	-2.10304600
C	0.68921600	3.02864400	0.02379100
C	-0.73682100	4.97763800	-1.41214700
H	-0.02625000	3.94155200	-3.19606300
C	0.01792700	4.05176800	0.70661500
H	1.26148000	2.26367400	0.55652600
C	-0.71341500	5.01373900	-0.00714300
H	-1.31001600	5.72602700	-1.96947500
H	0.06770000	4.10050300	1.80058200
H	-1.25192000	5.80246000	0.52917700
C	-1.01748200	-2.78159900	1.99689300
C	0.48010000	-2.84955600	2.30807600
H	-1.34203800	-3.67452900	1.43678900
H	-1.62502200	-2.73437700	2.91845700
C	1.00563100	-1.60602300	3.06223800
H	0.70247500	-3.76895800	2.88064400
H	1.01947000	-2.95689800	1.34888300
C	2.35287200	-1.10413700	2.53097800
H	0.27710500	-0.77859900	2.96741900
H	1.08530400	-1.81525100	4.14481400
H	2.68073700	-0.21081800	3.08818800
H	3.14730900	-1.86444600	2.63819900

transition state **TS2**

C	-2.52306000	-0.30854000	4.65964000
C	-3.66151900	-0.24471800	3.83948000
C	-3.52543700	-0.03594800	2.46057500
C	-2.24369100	0.10140900	1.89118400
C	-1.10900800	0.06740400	2.72237900
C	-1.24665400	-0.14680000	4.10108400
P	-1.99320600	0.34079900	0.08017300
C	-3.29424900	1.55600900	-0.36375500
C	-3.98034800	1.44082500	-1.58659600
C	-4.87199700	2.44481000	-1.98868200
C	-5.07759800	3.57007200	-1.17795700
C	-4.39988400	3.68375000	0.04532600
C	-3.51531500	2.68025400	0.45583700
C	-2.62138600	-1.18627000	-0.68966400
C	-2.13559900	-1.66839800	-1.96519400
C	-2.82548000	-2.88645000	-2.27340000
C	-3.74067300	-3.16428100	-1.20254900
C	-3.62182500	-2.12110100	-0.22494600
Fe	-1.82332300	-3.01987900	-0.49293000
C	-1.47194600	-4.35724200	1.01349600
C	-0.82182300	-3.10358900	1.26435000
C	0.07900300	-2.84008800	0.16215000
C	-0.03158000	-3.94467500	-0.76340500
C	-0.98890100	-4.87282500	-0.23608700
P	1.07150500	-1.32962600	-0.08357800
C	2.13465800	-1.31233800	1.42408100
C	2.78797800	-0.10789900	1.74860900
C	3.63139800	-0.04034800	2.86398500
C	3.80957000	-1.16878200	3.67938000
C	3.15041000	-2.36797900	3.36790400
C	2.31838000	-2.44567300	2.24138600
Pd	0.11861400	0.82094100	-0.49976800
C	-0.49303300	2.77349000	-0.62686900
C	-1.06096900	3.30350200	-1.79755500
C	-1.45954800	4.64848900	-1.84683200

C	-1.28899100	5.47925600	-0.72871700
C	-0.70965700	4.95926100	0.43689900
C	-0.31132300	3.61148700	0.48773000
C	2.21069500	-1.87174400	-1.42518200
C	3.17875400	-2.87044300	-1.20156200
C	4.00756300	-3.28868500	-2.24962900
C	3.87564700	-2.71232300	-3.52375400
C	2.91820000	-1.71293900	-3.74561000
C	2.08791400	-1.28865400	-2.69856600
H	-2.65605200	-3.51169100	-3.15162700
H	-1.34867700	-1.18762400	-2.55038700
H	-4.15637700	-2.06651900	0.72330500
H	-4.38700700	-4.04046100	-1.12268500
H	0.50720900	-4.03037600	-1.70745500
H	-0.98436400	-2.45323200	2.12389700
H	-1.31439200	-5.79594800	-0.71880200
H	-2.22890400	-4.81868800	1.65004800
H	-1.21222000	2.66265600	-2.67239500
H	0.12973600	3.21339000	1.40838900
H	-1.90934700	5.04736900	-2.76325400
H	-0.56593400	5.60147000	1.31400600
H	-1.60468600	6.52764500	-0.76770900
H	3.28897000	-3.31324000	-0.20605800
H	1.36753800	-0.47506000	-2.84091500
H	4.76328900	-4.06120200	-2.07104500
H	2.82591900	-1.24727400	-4.73229000
H	4.52937600	-3.03705200	-4.34058600
H	2.61766200	0.77415300	1.11871400
H	1.80825600	-3.38450500	2.00286700
H	4.14490600	0.89993300	3.09073200
H	3.28561400	-3.25015800	4.00328400
H	4.45983100	-1.11488400	4.55934900
H	-3.80881600	0.57082000	-2.22826100
H	-2.97617500	2.78059400	1.40219600
H	-5.40209500	2.34938800	-2.94235500
H	-4.55033800	4.56391600	0.67862600
H	-5.76452500	4.36005300	-1.49967400
H	-4.41561100	0.03923100	1.82673100
H	-0.11822600	0.20978000	2.27388100
H	-4.65957000	-0.35143700	4.27815100
H	-0.35470200	-0.18074900	4.73548300
H	-2.63452500	-0.47074400	5.73712200
N	1.88507700	1.42627000	-1.08172800
N	2.98693900	1.68558400	-1.57168300
C	3.98869500	2.23273700	-0.66183600
C	5.33189600	1.91609400	-0.90818900
C	3.63433800	3.07768100	0.40359800
C	6.32665700	2.40530600	-0.05066700
H	5.56943600	1.27225500	-1.76221600
C	4.63583800	3.59376200	1.23567400
H	2.57427700	3.31439600	0.54947900
C	5.98110300	3.24825700	1.01901600
H	7.37547600	2.13523500	-0.21759200
H	4.37134300	4.27297900	2.05482300
H	6.76208000	3.64784000	1.67532400

transition state **TS2**^{dtbpf}

P	-1.13517500	1.75403000	0.30589900
C	-2.77662300	0.97773800	-0.01726700
C	-2.95371700	0.33369400	-1.30093200
C	-4.31658100	-0.07702700	-1.44465000
C	-5.01428700	0.31517400	-0.25630500
C	-4.08369800	0.96895900	0.61832500
Fe	-3.43885900	-0.91086900	0.20586400
C	-3.68067700	-2.06800300	1.87645600
C	-2.30593700	-1.73431400	1.65051800
C	-1.90859500	-2.21714200	0.34137100
C	-3.07731200	-2.86257700	-0.22072800
C	-4.15888700	-2.75996200	0.71699700
P	-0.21299900	-1.94722900	-0.30545600
Pd	0.60285700	0.39035400	-0.38816400
C	1.70489600	2.08874900	-0.68373600
C	1.93240600	2.49109300	-2.01435200
C	2.79810200	3.55795200	-2.30196200
C	3.45960000	4.23293800	-1.26461000
C	3.26907700	3.81397500	0.05905300
C	2.40493100	2.74257300	0.34452900
H	-4.73598000	-0.62107900	-2.29252100
H	-2.14338800	0.17072100	-2.00633600
H	-4.33957100	1.36516300	1.59688100
H	-6.06478100	0.12182200	-0.03050000
H	-3.15341300	-3.31969900	-1.20580000

H	-1.66962200	-1.17842000	2.33583100
H	-5.17554500	-3.12247400	0.55518300
H	-4.26332100	-1.80913200	2.76200600
H	1.44163800	1.96080100	-2.83640900
H	2.28917400	2.42349600	1.38474800
H	2.96021700	3.85775600	-3.34370200
H	3.79838000	4.31534000	0.87756200
H	4.13281000	5.06734300	-1.48877100
N	2.34988200	-0.39109700	-0.83499500
N	3.49391100	-0.70794100	-1.17855700
C	4.48437200	-0.86790500	-0.10640000
C	5.63706500	-1.60197600	-0.41881000
C	4.32786600	-0.29430500	1.16464800
C	6.62489300	-1.79497800	0.55687800
H	5.73134500	-2.01411200	-1.43003000
C	5.32634500	-0.46504300	2.12912300
H	3.41351600	0.27771100	1.35661500
C	6.47250700	-1.22354500	1.83082000
H	7.51852300	-2.38564400	0.32539000
H	5.21963900	-0.00535200	3.11916500
H	7.25148500	-1.36030300	2.58913600
C	-0.20063900	-2.75266200	-2.05582300
C	-0.54088300	-4.25617200	-2.04357200
C	-1.21302700	-1.98596200	-2.93282700
C	1.17451500	-2.54076800	-2.72549400
H	-1.48934200	-4.48426700	-1.53289000
H	-0.62640600	-4.60655900	-3.08936300
H	0.25522800	-4.84769300	-1.56532800
H	-0.89679700	-0.93352800	-3.03698700
H	-1.21418800	-2.43820800	-3.94177900
H	-2.24095300	-2.00951900	-2.54206600
H	1.09474800	-2.87451600	-3.77704700
H	1.46060900	-1.47689400	-2.69899200
H	1.98045300	-3.11481600	-2.24944200
C	0.77809200	-3.10695700	0.89990800
C	2.09173200	-3.60964800	0.27340200
C	1.14858100	-2.25204200	2.12738800
C	-0.06508300	-4.31302200	1.36139100
H	1.92041000	-4.32947000	-0.54198800
H	2.70982700	-2.78291500	-0.10053500
H	2.66899400	-4.13065000	1.05933800
H	0.25606700	-1.92831100	2.68651500
H	1.76810300	-2.85867800	2.81385700
H	1.72396300	-1.36289300	1.82294600
H	0.55507000	-4.93218700	2.03682300
H	-0.96212500	-4.00105400	1.91933400
H	-0.38606800	-4.95043300	0.52346700
C	-1.51444700	3.43242000	-0.62610300
C	-0.44218500	4.53901500	-0.51315100
C	-1.63124600	3.05939000	-2.12095100
C	-2.85729500	4.01359900	-0.13729500
H	-0.37711200	4.97283700	0.49209600
H	0.55403400	4.20068100	-0.81974400
H	-0.74684400	5.35119300	-1.19957600
H	-2.52541400	2.45237100	-2.32891100
H	-1.71130500	3.99056600	-2.71106800
H	-0.73880700	2.51087800	-2.46444000
H	-3.06712500	4.93819000	-0.70701800
H	-3.69788800	3.32085000	-0.29757700
H	-2.82493800	4.28760700	0.93126300
C	-0.97994300	2.11295900	2.21768500
C	0.00991300	1.07838400	2.78392100
C	-0.40500700	3.51318000	2.50232700
C	-2.31064900	1.97166500	2.97519700
H	-0.34359100	0.05026800	2.63127600
H	0.99850400	1.14718400	2.30328900
H	0.12805300	1.24584700	3.87127900
H	-1.12210000	4.31374200	2.26218300
H	-0.18566600	3.57820300	3.58461800
H	0.53138600	3.69782300	1.95559600
H	-2.12499900	2.16622800	4.04827800
H	-3.06378200	2.69928400	2.63180600
H	-2.72518000	0.95575900	2.88385200

transition state **TS2**$^{\mathbf{dppb}}$

C	3.84515500	-2.99438500	3.48757900
C	4.67670300	-2.02660500	2.90212100
C	4.14183100	-1.08361200	2.01432600
C	2.76808100	-1.10596700	1.70352500
C	1.94060900	-2.07749800	2.29359400
C	2.47618700	-3.01812100	3.18370900
P	2.00184300	0.18564100	0.63318700

C	3.29826300	0.53621400	-0.61942100
C	3.52533800	1.85461400	-1.06605900
C	4.43083100	2.10380200	-2.10578100
C	5.11697000	1.04098300	-2.71353600
C	4.89231300	-0.27250000	-2.27565500
C	3.98677900	-0.52825600	-1.23896100
C	2.09503500	1.68370800	1.74554600
C	-0.95089400	1.80442200	2.66062200
P	-1.13902800	1.53738600	0.80879500
C	-2.95476100	1.44695400	0.58139400
C	-3.61130800	2.17862400	-0.42368200
C	-4.97419800	1.96064400	-0.67283500
C	-5.68818800	1.01472900	0.07573200
C	-5.03582300	0.27646500	1.07346900
C	-3.67397800	0.48426700	1.32014000
Pd	-0.01915900	-0.26987900	-0.20874200
C	0.96161600	-1.69004100	-1.33974300
C	1.30083500	-1.32764700	-2.65808200
C	1.94837600	-2.23651900	-3.50893500
C	2.27607500	-3.52157600	-3.05134700
C	1.93091300	-3.89596600	-1.74500600
C	1.27072600	-2.98795600	-0.89771100
C	-0.67704400	3.14968000	0.04436900
C	-1.10724600	4.38627700	0.56772700
C	-0.72961200	5.58369200	-0.05353400
C	0.07666500	5.55795600	-1.20331600
C	0.49778300	4.33127400	-1.73598200
C	0.11896400	3.13130700	-1.11702200
H	1.06884500	-0.32225600	-3.02497300
H	1.00396100	-3.30775200	0.11348800
H	2.20399400	-1.93432800	-4.53115500
H	2.17097200	-4.90115200	-1.37879900
H	2.78975900	-4.22957700	-3.71125600
H	-1.74866100	4.41519100	1.45556800
H	0.43771800	2.16113700	-1.52032400
H	-1.06745900	6.54083000	0.35807700
H	1.12225800	4.30351900	-2.63521500
H	0.37124900	6.49645500	-1.68506400
H	-3.05476600	2.90438200	-1.02431400
H	-3.16209500	-0.13213200	2.06725900
H	-5.47606300	2.52846000	-1.46343600
H	-5.57772000	-0.49073700	1.63533100
H	-6.74849000	0.83667700	-0.13111000
H	2.98844500	2.69339300	-0.61216100
H	3.80244700	-1.55842300	-0.91811800
H	4.60057200	3.13282200	-2.44106700
H	5.41387400	-1.10941600	-2.75147200
H	5.82222100	1.23660200	-3.52826300
H	4.79383600	-0.33347300	1.55334700
H	0.87329500	-2.08434800	2.03737800
H	5.74651500	-2.00806200	3.13657600
H	1.82499400	-3.77426000	3.63514900
H	4.26669800	-3.73204400	4.17882100
N	-1.69018400	-0.80094000	-1.06079400
N	-2.73669200	-1.05582900	-1.64755700
C	-3.66041800	-1.99772000	-0.99628200
C	-4.94746400	-2.07096700	-1.54253500
C	-3.31240300	-2.76665800	0.12307900
C	-5.90978500	-2.89754500	-0.94594800
H	-5.17102700	-1.45416100	-2.41978500
C	-4.26849100	-3.60288800	0.70808100
H	-2.29176600	-2.67234700	0.51466000
C	-5.57198200	-3.66375200	0.18093600
H	-6.92425900	-2.94670500	-1.35761800
H	-4.00463900	-4.21566700	1.57875200
H	-6.32038200	-4.31600400	0.64476100
H	3.15483000	1.99274900	1.78499300
H	1.53924300	2.49429100	1.24057200
H	-1.93468400	1.55333900	3.09103000
H	-0.78320800	2.87992400	2.84997800
C	1.57610400	1.46031800	3.17618100
H	2.25060900	0.75238700	3.69060100
H	1.66586000	2.42432400	3.71330300
C	0.13774900	0.94131300	3.31671800
H	-0.08659400	0.85916500	4.39612100
H	0.06901200	-0.08275100	2.90435700

intermediate (*E*)-**F**

C	-2.35313100	1.99979300	4.44870100
C	-3.52399700	1.75905800	3.71248200
C	-3.44243000	1.29599700	2.39242300
C	-2.18510300	1.06078500	1.80148000

C	-1.01422500	1.32354000	2.53841100	H	-1.96193000	-2.14652800	-1.73762500
C	-1.09879100	1.78621200	3.85905400	H	-3.83869300	-1.33142700	2.16049100
P	-2.02750900	0.43200400	0.07813000	H	-4.37075900	-3.90902000	1.49080100
C	-3.34442300	1.36069000	-0.80870900	H	0.07234600	-4.61970200	-0.11803200
C	-4.15258100	0.72748300	-1.77316600	H	-0.27812300	-1.17012900	2.59780200
C	-5.09519500	1.46744400	-2.50043000	H	-1.33461200	-5.40591900	2.06663600
C	-5.24066800	2.84360400	-2.27098500	H	-1.54752900	-3.27739600	3.74676700
C	-4.43739300	3.47802800	-1.31186400	H	-0.97596400	2.37544300	-2.85988600
C	-3.48950300	2.74607900	-0.58716700	H	0.45725800	3.42056000	1.09310600
C	-2.75218700	-1.23954500	0.17987500	H	-1.50814200	4.75711600	-3.29694200
C	-2.54921000	-2.26082800	-0.82595000	H	-0.16800400	5.79315900	0.68717900
C	-3.21077000	-3.45611700	-0.39086400	H	-1.14507100	6.47954600	-1.51460600
C	-3.82219100	-3.18960200	0.87993500	H	2.24903100	-4.04078600	-1.20214600
C	-3.54452600	-1.82835100	1.23599800	H	-0.14920000	-0.80827300	-2.77602700
Fe	-1.80541500	-2.84501200	0.96422900	H	2.15861800	-5.19530200	-3.41376200
C	-1.01159600	-3.29521200	2.79636500	H	-0.23617100	-1.95964500	-5.00850400
C	-0.34013400	-2.18128000	2.19433900	H	0.91087000	-4.16146200	-5.31607300
C	0.19986300	-2.61511400	0.92293100	H	3.50112100	-1.08621400	-1.73517800
C	-0.15191400	-4.00712100	0.75526500	H	2.39309900	-1.97068600	2.35910300
C	-0.89726000	-4.41820100	1.90971900	H	5.88202900	-0.87511500	-1.04215600
P	1.02682200	-1.48651900	-0.23487000	H	4.77623100	-1.75504900	3.04670600
C	2.79013600	-1.56209000	0.26304700	H	6.52807600	-1.19663100	1.34922100
C	3.78166900	-1.24922000	-0.69035900	H	-4.05263000	-0.34816800	-1.94978800
C	5.12073900	-1.12966600	-0.29840000	H	-2.85126900	3.25413400	0.14245500
C	5.48271300	-1.30859300	1.04443100	H	-5.72171000	0.96405400	-3.24479700
C	4.49951900	-1.61508800	1.99615800	H	-4.53415700	4.55393600	-1.13386100
C	3.15733500	-1.73749400	1.61065000	H	-5.97781700	3.42043300	-2.83984700
Pd	0.22383300	0.73192400	-0.48907200	H	-4.35588900	1.13201100	1.81081500
C	-0.23569600	2.71554900	-0.84110600	H	-0.03839100	1.17800300	2.05436100
C	-0.77542900	3.11875300	-2.07864400	H	-4.50445600	1.94086100	4.16573800
C	-1.08931300	4.46513500	-2.32648500	H	-0.18208300	1.99172800	4.42168100
C	-0.88729900	5.43058900	-1.32900100	H	-2.42018600	2.36750600	5.47827700
C	-0.34159500	5.04377500	-0.09482300	N	2.16431800	1.29408000	-0.70811300
C	-0.00542500	3.69929800	0.13831500	N	2.63844400	1.75093000	0.34122500
C	1.00891700	-2.37610600	-1.84164700	C	4.05224500	2.03844800	0.32506400
C	1.67983500	-3.60133700	-2.02836200	C	4.68953300	2.12452000	1.57155600
C	1.63741200	-4.24219500	-3.27348600	C	4.78425600	2.21144900	-0.86407200
C	0.93809900	-3.65961500	-4.34292300	C	6.07091000	2.35026500	1.63302600
C	0.29123600	-2.42732300	-4.17048500	H	4.08660800	1.98945100	2.47499100
C	0.33062400	-1.78478600	-2.92461700	C	6.15920200	2.45576900	-0.79599900
H	-3.21385300	-4.41092300	-0.91937300	H	4.25106300	2.14334000	-1.81714100

C	6.80786100	2.51675500	0.45047900
H	6.57442400	2.40030500	2.60470100
H	6.73457600	2.60147800	-1.71747000
H	7.88601500	2.70496700	0.49805200

intermediate (*E*)-**F**$^{\mathbf{dtbpf}}$

P	1.34869000	1.65028000	-0.43405200
C	2.94707300	0.76059200	-0.20332500
C	3.23477400	0.26185800	1.12612200
C	4.55908400	-0.27877200	1.15624400
C	5.11705900	-0.12867300	-0.15471300
C	4.14183500	0.51273600	-0.98882200
Fe	3.38702400	-1.22031800	-0.23038500
C	3.44978400	-2.66261200	-1.67754800
C	2.12603800	-2.14313600	-1.50190400
C	1.71072400	-2.35960300	-0.12738100
C	2.81842000	-3.02132900	0.52780900
C	3.88052200	-3.19787100	-0.42081600
P	0.04353200	-1.87282100	0.46485200
Pd	-0.60957600	0.47934600	0.36403000
C	-1.51273600	2.25425400	0.86349500
C	-1.37832800	2.75441200	2.17483900
C	-2.02127800	3.94198700	2.56495800
C	-2.80809900	4.65269900	1.64591300
C	-2.97819200	4.14554900	0.34821500
C	-2.34853700	2.94800100	-0.02974900
H	5.03841400	-0.75430200	2.01356700
H	2.52959300	0.28296800	1.95167000
H	4.29053100	0.74311900	-2.04024100
H	6.10023700	-0.47386900	-0.48073200
H	2.86695300	-3.31301100	1.57589900
H	1.54039600	-1.64370900	-2.27119900
H	4.85781700	-3.63419800	-0.20651400
H	4.03563800	-2.61944100	-2.59715900
H	-0.76305000	2.21852500	2.90789800
H	-2.53328000	2.53407600	-1.02971100
H	-1.90305300	4.31439400	3.58974000
H	-3.61004400	4.68090300	-0.37092100
H	-3.29910200	5.58574500	1.94389600
N	-2.54057800	-0.19716900	0.47833400
N	-3.11394500	0.02655900	-0.59966500
C	-4.47182600	-0.44635300	-0.71255300
C	-4.99700200	-0.54988400	-2.01060200
C	-5.26028800	-0.78000100	0.40509100
C	-6.30534600	-1.01505500	-2.19710500
H	-4.36061300	-0.26622900	-2.85528000
C	-6.57022200	-1.22925800	0.21230600
H	-4.82282400	-0.66918600	1.40209300
C	-7.09405700	-1.35424600	-1.08683900
H	-6.71427100	-1.10721600	-3.20918300
H	-7.19408000	-1.48035900	1.07765000
H	-8.12122900	-1.70616600	-1.23126400
C	-0.01454800	-2.30841700	2.33377500
C	0.18098400	-3.80169700	2.65568200
C	1.07152700	-1.46813400	3.03575100
C	-1.36239300	-1.82231400	2.91546500
H	1.10622700	-4.21672700	2.22584200
H	0.23319600	-3.92530700	3.75403600
H	-0.66452200	-4.40946200	2.29727300
H	0.89487800	-0.39592800	2.83697400
H	0.99721900	-1.63127700	4.12691200
H	2.09274000	-1.72428600	2.71692500
H	-1.34299400	-1.99027700	4.00884200
H	-1.50722400	-0.74679100	2.72284700
H	-2.22947200	-2.35405900	2.50296500
C	-1.02471400	-3.16900700	-0.49878700
C	-2.38236400	-3.41108900	0.19145000
C	-1.29479900	-2.55855300	-1.89093500
C	-0.29369100	-4.51413000	-0.68040200
H	-2.27484100	-3.99583500	1.11911900
H	-2.90158600	-2.46926900	0.42635000
H	-3.02254900	-3.99507600	-0.49548500
H	-0.36780200	-2.45740900	-2.47772800
H	-1.96695200	-3.23427900	-2.45238100
H	-1.77782200	-1.57176600	-1.81692500
H	-0.95614500	-5.19980600	-1.24237900
H	0.63966500	-4.40000600	-1.25480900
H	-0.05071000	-4.99503900	0.27952300
C	1.94625800	3.37789300	0.23736500
C	0.88804200	4.50076700	0.15880600
C	2.26161300	3.15758500	1.73485400
C	3.22296000	3.83973900	-0.49051400

H	0.74085800	4.87630300	-0.86154100
H	-0.08301700	4.19419400	0.56744600
H	1.25947000	5.34617100	0.76752700
H	3.15302800	2.53000100	1.88574100
H	2.45962600	4.14066700	2.19978000
H	1.40529300	2.70189000	2.25964000
H	3.58032000	4.77835500	-0.02576700
H	4.03431900	3.09692800	-0.41965600
H	3.03194000	4.05321800	-1.55607100
C	0.97479000	1.80121600	-2.32755200
C	-0.07097100	0.72104400	-2.67379300
C	0.33334700	3.16398400	-2.65057800
C	2.20260700	1.59783900	-3.23107100
H	0.28353300	-0.28668800	-2.41910400
H	-1.02495100	0.88069300	-2.13959800
H	-0.26919900	0.74979300	-3.76278900
H	1.06269300	3.98817400	-2.61245300
H	-0.06373400	3.11818600	-3.68192400
H	-0.50536900	3.39277100	-1.97473800
H	1.89940600	1.77257300	-4.28064400
H	3.02003900	2.29911800	-2.99782600
H	2.58431500	0.56682100	-3.16311000

intermediate (*E*)-**F**$^{\mathbf{dppb}}$

C	4.54970300	-2.63131800	3.14873700
C	5.20880500	-1.51368600	2.61021900
C	4.51887000	-0.62965100	1.77188300
C	3.16109600	-0.85821900	1.46548700
C	2.50701500	-1.97839600	2.00781700
C	3.20077800	-2.86247000	2.84680000
P	2.20665300	0.31559600	0.42362100
C	3.32208900	0.62860400	-0.99938800
C	3.29809600	1.87697200	-1.65654000
C	4.07961900	2.09176900	-2.79934200
C	4.89168300	1.06420900	-3.30258700
C	4.91836700	-0.17995600	-2.65514200
C	4.13780100	-0.40127800	-1.51307200
C	2.37599300	1.91067000	1.39068100
C	-0.55569200	1.94466000	2.66003000
P	-0.99160700	1.53858100	0.88158000
C	-2.82245900	1.43217700	0.92247000
C	-3.61971700	2.04826100	-0.06115800
C	-5.00038300	1.80929600	-0.10147200
C	-5.59810100	0.94594700	0.82675100
C	-4.80693800	0.31509700	1.79729900
C	-3.42736000	0.54880700	1.84103900
Pd	0.02963500	-0.35477600	-0.11852600
C	0.72169400	-2.11778500	-0.95868000
C	1.52155600	-2.15195300	-2.11720700
C	1.97570400	-3.37229300	-2.64903300
C	1.63667800	-4.58250100	-2.02784000
C	0.81442300	-4.56495900	-0.89004300
C	0.35169300	-3.34443600	-0.37268900
C	-0.67238500	3.06658400	-0.09091300
C	-0.95735300	4.36228300	0.38488600
C	-0.69424700	5.48090800	-0.41641600
C	-0.15435900	5.31599300	-1.70245600
C	0.11766300	4.02923400	-2.18874900
C	-0.13950100	2.90824100	-1.38602300
H	1.79654500	-1.22069500	-2.62476300
H	-0.33635400	-3.35079400	0.48391900
H	2.59587100	-3.37337400	-3.55408700
H	0.52208400	-5.50750700	-0.41066500
H	1.99568100	-5.53409000	-2.43622400
H	-1.39550500	4.49918400	1.37961600
H	0.07307500	1.89419800	-1.74932800
H	-0.91522700	6.48520200	-0.03935000
H	0.53439800	3.89403500	-3.19233600
H	0.04998100	6.19309500	-2.32574200
H	-3.15905600	2.71262600	-0.79885500
H	-2.81518100	0.01428200	2.57596500
H	-5.61002000	2.29373600	-0.87163500
H	-5.26141800	-0.38293900	2.50730500
H	-6.67302500	0.74559400	0.78029600
H	2.66435600	2.68645900	-1.28102900
H	4.15377700	-1.37833700	-1.02176500
H	4.05378900	3.06728800	-3.29717400
H	5.54666700	-0.98865700	-3.04339600
H	5.50149300	1.23277800	-4.19646700
H	5.03846200	0.23470400	1.34298700
H	1.45942500	-2.15777900	1.74725300
H	6.26419800	-1.33407800	2.84189800

H	2.68627300	-3.73817700	3.25586000
H	5.09298000	-3.32428200	3.80023300
N	-1.79120600	-0.91535500	-0.78690100
N	-2.40816200	-1.73837400	-0.09986800
C	-3.78022000	-1.99428000	-0.48468400
C	-4.49499400	-2.88660500	0.32895900
C	-4.41125700	-1.35902200	-1.56900800
C	-5.84848900	-3.13898400	0.06848500
H	-3.96941500	-3.35992800	1.16481200
C	-5.76039400	-1.61646900	-1.82683900
H	-3.82306500	-0.66346100	-2.17483400
C	-6.48438400	-2.50203900	-1.00820000
H	-6.40916500	-3.83286900	0.70451700
H	-6.25958800	-1.12187300	-2.66812700
H	-7.54290700	-2.69652000	-1.21311700
H	3.41149800	2.27832500	1.27836300
H	1.71619000	2.64731500	0.89640100
H	-1.45607300	1.68694700	3.24210600
H	-0.41710200	3.03645500	2.76043900
C	2.03098700	1.77448100	2.88249100
H	2.80454400	1.15270200	3.36786200
H	2.10504100	2.78028800	3.33990700
C	0.65642200	1.17447900	3.21326200
H	0.56479100	1.13228000	4.31409300
H	0.60765800	0.12676100	2.85773500

intermediate (*E*)-**F**^{2c}

C	6.85952000	1.69602100	0.37402100
C	6.13112300	1.64862700	1.56676800
C	4.73507600	1.57334100	1.49535300
C	4.09253000	1.52093500	0.24925600
C	4.84395500	1.57729100	-0.93992500
C	6.23617700	1.67240100	-0.88122300
N	2.65821400	1.38761200	0.26734700
N	2.13274200	0.99197900	-0.78326200
Pd	0.14247900	0.65495800	-0.53651400
C	-0.09107700	2.67265800	-0.91706400
C	-0.61165800	3.11540200	-2.14923000
C	-0.77347200	4.48500300	-2.41520700
C	-0.43555700	5.43649200	-1.44134400
C	0.09276500	5.00906100	-0.21301000
C	0.27633700	3.63853000	0.03789600
P	-2.11048700	0.63597200	0.09581500
C	-3.02371000	-0.93569300	0.25408600
C	-2.97947300	-1.99026600	-0.73668100
C	-3.76608200	-3.08887800	-0.25698600
C	-4.29761300	-2.72939300	1.02676300
C	-3.84533400	-1.40648000	1.34633100
Fe	-2.25232800	-2.63152000	1.04098300
C	-1.50922800	-4.29203300	1.98145400
C	-1.46371800	-3.15476800	2.85587200
C	-0.68349000	-2.13407700	2.22045900
C	-0.23571900	-2.64188700	0.94053200
C	-0.75477100	-3.98379600	0.80126000
P	0.68525000	-1.63507800	-0.25814500
C	0.52641100	-2.54859100	-1.84331700
C	1.05547500	-3.84296900	-2.01932200
C	0.90943000	-4.49976000	-3.24818900
C	0.24668600	-3.86614500	-4.31188600
C	-0.25963200	-2.56834600	-4.15028100
C	-0.11584100	-1.90925200	-2.92088600
C	-2.13247500	1.30485500	1.81131100
C	-3.33139100	1.70259500	2.43567600
C	-3.31255600	2.19090700	3.74905400
C	-2.09790000	2.29489700	4.44512600
C	-0.89924900	1.91900500	3.82169800
C	-0.91487800	1.43087500	2.50763800
C	-3.33774100	1.70036600	-0.76654400
C	-4.25367000	1.15193600	-1.68564800
C	-5.12728900	1.98731700	-2.39551800
C	-5.09593700	3.37505600	-2.19367900
C	-4.18501800	3.92510200	-1.27964800
C	-3.30531300	3.09708700	-0.57271300
C	2.44046600	-1.90072800	0.20422700
C	3.43865300	-1.73342300	-0.77821200
C	4.79169700	-1.75929400	-0.41741000
C	5.16288500	-1.93708300	0.92322900
C	4.17334600	-2.09589900	1.90403700
C	2.81728600	-2.07448300	1.54936400
H	-3.90273500	-4.04464200	-0.76575600
H	-2.41459900	-1.96267200	-1.66905100
H	-4.04518900	-0.86235500	2.26946900

H	-4.90815700	-3.36684200	1.66904800
H	-0.63026400	-4.62691100	-0.07000600
H	-0.49152400	-1.13354700	2.60932500
H	-2.05570700	-5.21920100	2.16360300
H	-1.96570900	-3.06411000	3.82054700
H	-0.91674400	2.38790300	-2.91123500
H	0.72667700	3.32294000	0.98723000
H	-1.18030100	4.80797100	-3.38091600
H	0.37158000	5.74580600	0.55031800
H	-0.57475200	6.50510900	-1.64093300
H	1.59593500	-4.32548200	-1.19784700
H	-0.48450800	-0.88436800	-2.78181100
H	1.32094800	-5.50619000	-3.38033100
H	-0.75801700	-2.06342000	-4.98442300
H	0.13832200	-4.38103500	-5.27246800
H	3.15395300	-1.57184500	-1.82220300
H	2.04957500	-2.19449300	2.32055400
H	5.55955900	-1.62224300	-1.18517700
H	4.45674600	-2.23385900	2.95303700
H	6.22115700	-1.94097100	1.20327000
H	-4.29201100	0.06894700	-1.84006700
H	-2.58258900	3.53745700	0.12120500
H	-5.83869300	1.54989300	-3.10442800
H	-4.14395200	5.00796100	-1.12366900
H	-5.77943900	4.02639100	-2.74891800
H	-4.27639700	1.64463500	1.88507500
H	0.02007000	1.15962800	1.99752100
H	-4.24798400	2.49931200	4.22825500
H	0.05349500	2.01732400	4.35250800
H	-2.08585200	2.68262500	5.46944500
H	4.12175400	1.53145000	2.40058200
H	4.30963500	1.53849400	-1.89391900
H	6.66137400	1.67401600	2.52307700
H	6.85093400	1.72968100	-1.78485500
F	8.20795200	1.77693900	0.43216100

intermediate (*E*)-**F**^{2d}

C	6.78833400	1.24877500	0.52231700
C	5.99306400	1.24975100	1.68733300
C	4.60339000	1.26457400	1.58055500
C	3.98727600	1.25156400	0.31721300
C	4.77864700	1.26066500	-0.84301400
C	6.17397800	1.26743100	-0.74860800
N	2.55085800	1.20220400	0.29498300
N	2.02352100	0.84939800	-0.76965400
Pd	0.01140300	0.62545100	-0.54455900
C	-0.10336300	2.65239800	-0.93456000
C	-0.60741100	3.12147200	-2.16404600
C	-0.69078100	4.49732500	-2.43418000
C	-0.28999600	5.43073400	-1.46665600
C	0.22218700	4.97697500	-0.24083500
C	0.32737900	3.59889800	0.01349900
P	-2.23807600	0.73300700	0.09200500
C	-3.24016800	-0.78297500	0.25737400
C	-3.25675800	-1.84020700	-0.73137900
C	-4.10231200	-2.89259400	-0.24824300
C	-4.61042000	-2.50178500	1.03591200
C	-4.08444800	-1.20548500	1.35209800
Fe	-2.56296100	-2.51826800	1.04579600
C	-1.91165800	-4.21453400	1.98986300
C	-1.79826700	-3.07847600	2.85978200
C	-0.96362700	-2.10622900	2.21758500
C	-0.55028600	-2.64303800	0.93799200
C	-1.14515500	-3.95380400	0.80584100
P	0.42358100	-1.69193900	-0.26503700
C	0.20592100	-2.59635100	-1.84886000
C	0.65314500	-3.92109200	-2.02574600
C	0.46229700	-4.56826100	-3.25363200
C	-0.16346500	-3.89535100	-4.31562700
C	-0.58705400	-2.56827800	-4.15351700
C	-0.39828800	-1.91880000	-2.92510700
C	-2.21312800	1.40555100	1.80646500
C	-3.38458500	1.86968000	2.43710100
C	-3.33105800	2.35981800	3.74885500
C	-2.10811300	2.39964400	4.43702100
C	-0.93563700	1.95780700	3.80714800
C	-0.98587200	1.46757400	2.49468800
C	-3.40689900	1.86534700	-0.76505300
C	-4.35923700	1.36986500	-1.67725900
C	-5.18505000	2.25363000	-2.38571500
C	-5.06924100	3.63777100	-2.18959900
C	-4.12177900	4.13526700	-1.28265600

C	-3.28949900	3.25842300	-0.57706400
C	2.16111200	-2.05912600	0.19262200
C	3.16476700	-1.94147000	-0.79130100
C	4.51517600	-2.03644700	-0.43251400
C	4.87943700	-2.23500400	0.90697200
C	3.88469100	-2.34648200	1.88887100
C	2.53099100	-2.25524500	1.53667900
H	-4.29274600	-3.84028000	-0.75485400
H	-2.69218000	-1.84563500	-1.66435600
H	-4.25193000	-0.64959500	2.27466300
H	-5.25434900	-3.10306300	1.68051300
H	-1.06081900	-4.60633800	-0.06325500
H	-0.71302100	-1.11683600	2.60161800
H	-2.50927700	-5.10848100	2.17749700
H	-2.29062300	-2.95583000	3.82590100
H	-0.96167700	2.41065800	-2.92060700
H	0.76695900	3.26022400	0.96015700
H	-1.08609000	4.84038700	-3.39783400
H	0.54976700	5.69868500	0.51757400
H	-0.36817800	6.50506600	-1.66913500
H	1.16525700	-4.43560300	-1.20551900
H	-0.70115000	-0.87261900	-2.78505700
H	0.80971500	-5.59854800	-3.38635700
H	-1.05578000	-2.03371000	-4.98642000
H	-0.30736100	-4.40330700	-5.27528100
H	2.88698500	-1.76239700	-1.83428600
H	1.75950800	-2.33587700	2.30920300
H	5.28767800	-1.93490900	-1.20110500
H	4.16267300	-2.49854600	2.93733200
H	5.93655400	-2.28842000	1.18610100
H	-4.46285500	0.29053900	-1.82746900
H	-2.53736300	3.65708500	0.11085700
H	-5.92543800	1.85719800	-3.08909400
H	-4.01444200	5.21428000	-1.13144300
H	-5.71536300	4.32706300	-2.74383100
H	-4.33475300	1.86226700	1.89237600
H	-0.06973700	1.14513800	1.97994000
H	-4.24530900	2.72000500	4.23285800
H	0.02461100	2.00602800	4.33133200
H	-2.06846300	2.78900600	5.46006200
H	3.96652800	1.26164300	2.47066400
H	4.27511000	1.25649300	-1.81460700
H	6.49477100	1.24090900	2.65951600
H	6.77561400	1.28735200	-1.66151500
O	8.13952500	1.23751700	0.72284700
C	8.98931600	1.23865700	-0.41485800
H	10.01843200	1.22391000	-0.02620700
H	8.82334800	0.34455600	-1.04716300
H	8.84314600	2.14702600	-1.03088600

intermediate (*E*)-**F**^{2e}

C	6.44818900	1.09589000	0.24694800
C	5.70945300	1.10181400	1.44173500
C	4.31810300	1.22413200	1.39430800
C	3.66525800	1.30904600	0.15599700
C	4.40549600	1.34408000	-1.03953900
C	5.79654800	1.24237700	-0.99205400
N	2.22336800	1.30509100	0.18860200
N	1.67058800	0.92145100	-0.85426000
Pd	-0.31901900	0.65075600	-0.56693300
C	-0.51705000	2.66713500	-0.96360500
C	-1.05678700	3.10082200	-2.19081200
C	-1.19576000	4.46918500	-2.47520500
C	-0.81406900	5.42845600	-1.52548100
C	-0.26582400	5.00940200	-0.30311100
C	-0.10625200	3.63939000	-0.03334300
P	-2.54769400	0.69164600	0.14560900
C	-3.48517600	-0.85383000	0.39552100
C	-3.52768000	-1.93620700	-0.56490900
C	-4.31010600	-2.99993700	-0.00636400
C	-4.75232800	-2.59160700	1.29634300
C	-4.24878700	-1.27294500	1.54931800
Fe	-2.70811300	-2.54793500	1.18273800
C	-1.95287700	-4.20482500	2.12033400
C	-1.82350900	-3.04817100	2.96036100
C	-1.05667400	-2.06604300	2.25254400
C	-0.70276300	-2.61728900	0.96135900
C	-1.26454900	-3.94715400	0.88874700
P	0.16791300	-1.66154500	-0.31262000
C	-0.14644100	-2.57054900	-1.87694300
C	0.31027500	-3.88774400	-2.08340500
C	0.04562500	-4.54039100	-3.29454300

C	-0.66306100	-3.88008800	-4.31130000
C	-1.09672500	-2.55993100	-4.12148300
C	-0.83494800	-1.90515300	-2.90942900
C	-2.49266100	1.41256900	1.83887100
C	-3.65903100	1.86474300	2.48733600
C	-3.58447300	2.38914900	3.78471600
C	-2.34632600	2.47344700	4.44087200
C	-1.17988800	2.04122400	3.79337300
C	-1.25107400	1.51778400	2.49488300
C	-3.78219700	1.75382800	-0.70887900
C	-4.73887900	1.19577200	-1.57936500
C	-5.61927800	2.02622900	-2.28657200
C	-5.55390400	3.41866500	-2.13053900
C	-4.60239300	3.97828900	-1.26505200
C	-3.71580900	3.15498800	-0.56100800
C	1.94123300	-2.00097100	0.01508100
C	2.86693400	-1.85292000	-1.03866300
C	4.24147700	-1.94312600	-0.78464200
C	4.70881900	-2.16411700	0.51903600
C	3.79073600	-2.30921700	1.56946900
C	2.41302300	-2.22542000	1.32253700
H	-4.50108400	-3.96540300	-0.47794100
H	-3.02100800	-1.95033100	-1.53028500
H	-4.37829400	-0.69823600	2.46643200
H	-5.33732500	-3.19487600	1.99305600
H	-1.21106200	-4.61506000	0.02896600
H	-0.81144700	-1.06289500	2.60260100
H	-2.51172700	-5.11140200	2.35960100
H	-2.26260600	-2.92006800	3.95110300
H	-1.39474700	2.36805200	-2.93354500
H	0.35737200	3.32989600	0.91132100
H	-1.61857100	4.78530700	-3.43623200
H	0.04692700	5.75189100	0.44114800
H	-0.93487300	6.49647700	-1.73939700
H	0.88806100	-4.39182300	-1.30123900
H	-1.14734800	-0.86461200	-2.74981800
H	0.40072600	-5.56474700	-3.45022300
H	-1.63052600	-2.03484100	-4.92051000
H	-0.86391700	-4.39208800	-5.25841500
H	2.51007000	-1.65773100	-2.05441800
H	1.70223300	-2.33424900	2.14820100
H	4.95370400	-1.82181600	-1.60670400
H	4.14812300	-2.48190500	2.59033400
H	5.78536200	-2.20187300	0.71306200
H	-4.80402300	0.10954700	-1.69734700
H	-2.96280900	3.60198200	0.09547000
H	-6.36260800	1.58151300	-2.95709900
H	-4.53537800	5.06440500	-1.14487200
H	-6.24285900	4.06618500	-2.68345200
H	-4.62230800	1.81920400	1.96815200
H	-0.34079200	1.20334200	1.96552300
H	-4.49465500	2.74047100	4.28256000
H	-0.20879400	2.12310000	4.29277100
H	-2.29053500	2.88916400	5.45266000
H	3.71207900	1.21663700	2.30505000
H	3.86387300	1.42620500	-1.98619800
H	6.22679900	1.00202100	2.40037500
H	6.38711300	1.26299900	-1.91307400
C	7.92953400	0.83775200	0.29170700
F	8.19711000	-0.50259500	0.27887900
F	8.58178600	1.36980500	-0.76974700
F	8.50678800	1.33152400	1.41464100

intermediate (*E*)-**F**[2f]

C	-6.61771000	-2.38196300	0.38393100
C	-5.82301800	-2.13932100	1.51369900
C	-4.45969700	-1.88966100	1.34174100
C	-3.87263700	-1.86065500	0.06495100
C	-4.68408400	-2.10230200	-1.05799400
C	-6.04788500	-2.36785600	-0.90112500
N	-2.47082700	-1.57773400	-0.00888100
N	-2.04827600	-1.13131800	-1.08786700
Pd	-0.09820400	-0.61326100	-0.78336000
C	0.35694700	-2.56251500	-1.29602600
C	1.08282200	-2.85020800	-2.46892100
C	1.40562600	-4.17366900	-2.81052400
C	1.02471900	-5.23142900	-1.97120600
C	0.29701700	-4.95783900	-0.80240700
C	-0.04364900	-3.63374900	-0.47722900
P	2.03866700	-0.49339400	0.14964600
C	2.81794300	1.11726200	0.50029200
C	2.80435500	2.22093600	-0.43656000

C	3.45229100	3.34090000	0.18100600
C	3.86819400	2.94486400	1.49652600
C	3.48194100	1.57840500	1.69856700
Fe	1.84597000	2.70814600	1.27889700
C	0.88348100	4.25505000	2.21372000
C	0.83044700	3.06794600	3.01882300
C	0.20222200	2.03409500	2.25013700
C	-0.14335100	2.58426900	0.95630200
C	0.28455800	3.96487600	0.94312300
P	-0.86457900	1.59811000	-0.38771900
C	-0.67084400	2.65626200	-1.87508100
C	-1.32339900	3.89909900	-2.00069000
C	-1.14185100	4.67285200	-3.15450300
C	-0.31786800	4.20838900	-4.19254400
C	0.31582200	2.96211600	-4.08227600
C	0.13558600	2.18544800	-2.92871900
C	1.79976500	-1.24433500	1.81573700
C	2.89261500	-1.59020800	2.63549600
C	2.67380000	-2.14766400	3.90277700
C	1.36509200	-2.37518300	4.35773200
C	0.27380600	-2.05405600	3.53753300
C	0.48898400	-1.49591800	2.26982400
C	3.44875300	-1.45310500	-0.53595300
C	4.45840500	-0.81585900	-1.28363300
C	5.47954900	-1.57063100	-1.87733800
C	5.50253400	-2.96539200	-1.72992900
C	4.49860600	-3.60346200	-0.98639400
C	3.47346200	-2.85600400	-0.39536300
C	-2.66641600	1.65924400	-0.04757700
C	-3.56520500	1.43568100	-1.11182000
C	-4.93370500	1.28613400	-0.85591000
C	-5.41782700	1.34295500	0.45971300
C	-4.52698600	1.55728200	1.52042500
C	-3.15612200	1.71072300	1.27080000
H	3.57251300	4.33144800	-0.26099200
H	2.34430700	2.20632100	-1.42531300
H	3.62054800	0.99839800	2.61096000
H	4.35827000	3.58416700	2.23328300
H	0.20363600	4.64731700	0.09685100
H	0.04558700	1.00121500	2.56306800
H	1.33576400	5.20566700	2.50220300
H	1.23183700	2.95561500	4.02732800
H	1.42330900	-2.03598100	-3.12006400
H	-0.63996200	-3.43555200	0.42290000
H	1.97159500	-4.37680100	-3.72749000
H	-0.01277000	-5.77869800	-0.14400900
H	1.28861100	-6.26349100	-2.22886400
H	-1.98371800	4.24927700	-1.19998600
H	0.60216800	1.19607500	-2.83338400
H	-1.65011700	5.63854800	-3.24850600
H	0.94262700	2.58853700	-4.89879900
H	-0.18140000	4.81454400	-5.09452200
H	-3.18945200	1.36568700	-2.13713300
H	-2.46550800	1.86528700	2.10534000
H	-5.62346400	1.10556300	-1.68646600
H	-4.89616500	1.59271900	2.55071200
H	-6.48569700	1.20530700	0.65702800
H	4.45134200	0.27317600	-1.39547300
H	2.68085700	-3.36428200	0.16218800
H	6.26245300	-1.06491600	-2.45298600
H	4.50029400	-4.69267200	-0.87574400
H	6.30074100	-3.55370000	-2.19524100
H	3.91507900	-1.44155700	2.27181600
H	-0.36731300	-1.27054900	1.61770500
H	3.52840900	-2.41457000	4.53393600
H	-0.75212400	-2.24597600	3.86910600
H	1.19892700	-2.81693000	5.34624900
H	-4.20370600	-2.07051800	-2.04080200
H	-6.24093300	-2.13537500	2.52456900
H	-6.67242100	-2.56668200	-1.77850400
H	-7.68571100	-2.58766000	0.51098500
F	-3.70070900	-1.63210100	2.42786900

intermediate (*E*)-**F**^{2g}

C	-6.51454300	-2.29000000	-0.03630500
C	-5.77393000	-2.00034400	1.12096800
C	-4.38803100	-1.77549100	1.03117800
C	-3.76102600	-1.83025600	-0.23981800
C	-4.51534700	-2.11553400	-1.38631500
C	-5.89166900	-2.35397300	-1.29083700
N	-2.35501600	-1.57347000	-0.27806000
N	-1.89374600	-1.09842500	-1.32660800

Pd	0.03709500	-0.58124400	-0.90243100
C	0.54459500	-2.50561500	-1.45902400
C	1.36540000	-2.74210200	-2.57985900
C	1.72732800	-4.04881000	-2.94625400
C	1.29059600	-5.14180700	-2.18268600
C	0.46860300	-4.91967200	-1.06653300
C	0.09013300	-3.61187100	-0.71837500
P	2.09213100	-0.47992200	0.20250200
C	2.82162400	1.11480800	0.70542000
C	2.88885300	2.26574600	-0.17020300
C	3.46279500	3.35513100	0.56430900
C	3.75208600	2.89349800	1.89224800
C	3.36070900	1.51681900	1.98503600
Fe	1.76314000	2.66003700	1.46609700
C	0.70152700	4.15558800	2.37595800
C	0.58195900	2.93045400	3.11490900
C	0.03878300	1.93238600	2.24101500
C	-0.18463500	2.54297400	0.94741000
C	0.22957900	3.92438400	1.04123600
P	-0.77392300	1.61572800	-0.49864800
C	-0.42989700	2.72806000	-1.91869100
C	-1.07042900	3.97435700	-2.06730300
C	-0.77321400	4.78859800	-3.16816100
C	0.15516700	4.36100600	-4.13117200
C	0.77833000	3.11132100	-4.00000400
C	0.48255200	2.29435200	-2.89937300
C	1.73162100	-1.31790500	1.80554800
C	2.76435100	-1.69144500	2.68899900
C	2.46019300	-2.31771500	3.90538400
C	1.12492400	-2.58865900	4.24448100
C	0.09512800	-2.24074000	3.35860600
C	0.39444600	-1.61141400	2.14201800
C	3.56820600	-1.39147600	-0.40683100
C	4.63450800	-0.70944100	-1.02533200
C	5.71178300	-1.42539700	-1.56546800
C	5.73495600	-2.82612800	-1.49321800
C	4.67474600	-3.50888000	-0.87870400
C	3.59380300	-2.80008800	-0.34176000
C	-2.59969400	1.69360900	-0.33172300
C	-3.38833900	1.40733300	-1.46670700
C	-4.77770700	1.28545500	-1.34398500
C	-5.39349800	1.43956400	-0.09288300
C	-4.61338500	1.72596500	1.03585100
C	-3.22118900	1.84506500	0.92149100
H	3.61603500	4.36771900	0.18719400
H	2.52837300	2.30194100	-1.19893500
H	3.41466100	0.89081600	2.87567900
H	4.16187500	3.49594200	2.70518900
H	0.22341200	4.64629200	0.22430200
H	-0.14306500	0.88547300	2.48710900
H	1.11611500	5.09289600	2.75161300
H	0.88674300	2.77159300	4.15068600
H	1.74981300	-1.90035500	-3.16865900
H	-0.57873500	-3.45288200	0.13775700
H	2.36725200	-4.21148000	-3.82180000
H	0.11486600	-5.76829900	-0.46808600
H	1.58446600	-6.16104000	-2.45851700
H	-1.81152200	4.29547100	-1.32732500
H	0.93906900	1.30143700	-2.79075400
H	-1.27263400	5.75699400	-3.28043100
H	1.48700300	2.76653700	-4.76036700
H	0.38174800	4.99848200	-4.99254100
H	-2.91035100	1.26629400	-2.44062800
H	-2.61609500	2.05304200	1.80912400
H	-5.38094800	1.05002500	-2.22634000
H	-5.08931500	1.85256000	2.01452300
H	-6.47796500	1.32492100	0.00174500
H	4.62744100	0.38409500	-1.07730200
H	2.76028200	-3.34209600	0.11550900
H	6.53816600	-0.88487600	-2.03986800
H	4.67676400	-4.60254700	-0.82801400
H	6.57715300	-3.38409800	-1.91650700
H	3.80873700	-1.50978200	2.41326200
H	-0.41618800	-1.36821800	1.43979000
H	3.26889900	-2.60533400	4.58602600
H	-0.94887300	-2.47066500	3.59534900
H	0.89175300	-3.08630800	5.19217900
H	-3.98513100	-2.14078000	-2.34337500
H	-6.28324900	-1.94732200	2.08637200
H	-6.47564000	-2.58991100	-2.18657100
H	-7.59106000	-2.47173200	0.05340300
O	-3.59093900	-1.46427100	2.09284400

C	-4.19660700	-1.32295300	3.36789000
H	-4.95610000	-0.51761500	3.36681700
H	-4.66928900	-2.26655600	3.70328000
H	-3.38361400	-1.05658900	4.05994000

intermediate (*E*)-**F**$^{\mathbf{2h}}$

C	-6.70346000	-1.29860700	-0.65788200
C	-6.01860200	-1.41663500	0.55884900
C	-4.61535400	-1.40772100	0.58356200
C	-3.90044100	-1.25070500	-0.62300900
C	-4.59006100	-1.13921700	-1.84116300
C	-5.98702800	-1.17133200	-1.85880200
N	-2.46693600	-1.20402000	-0.53307900
N	-1.87253700	-0.66261000	-1.47909600
Pd	0.07718600	-0.44377400	-0.93595800
C	0.34701700	-2.38047600	-1.60051200
C	1.11333500	-2.64031500	-2.75389900
C	1.30176900	-3.95439000	-3.21311300
C	0.74282800	-5.03268700	-2.51117000
C	-0.02970200	-4.78501800	-1.36538900
C	-0.23704900	-3.46702400	-0.92418100
P	2.13276200	-0.65997100	0.15629500
C	2.99950900	0.79476200	0.83728500
C	3.22941600	2.02123100	0.10334800
C	3.87890300	2.95220600	0.97947200
C	4.05231900	2.31783500	2.25493200
C	3.51374900	0.99142500	2.17412000
Fe	2.07282400	2.35569800	1.73155000
C	1.13966200	3.85590300	2.76621900
C	0.84712100	2.58065600	3.35717200
C	0.24036700	1.75286700	2.35758300
C	0.15275500	2.52097500	1.13352300
C	0.71310600	3.82702900	1.39700800
P	-0.44514900	1.81325100	-0.42543900
C	0.16696200	2.95330700	-1.72830600
C	-0.25683700	4.29468600	-1.81003700
C	0.23834900	5.12622200	-2.82329500
C	1.14751800	4.62256900	-3.76756700
C	1.55287500	3.28122300	-3.70627500
C	1.06010600	2.44739100	-2.69229600
C	1.74308600	-1.64321700	1.66276100
C	2.76081900	-2.22517200	2.44527600
C	2.43264800	-2.95067200	3.59822000
C	1.08873100	-3.10828900	3.97331400
C	0.07117800	-2.54728900	3.18864000
C	0.39657200	-1.82070600	2.03469000
C	3.51127800	-1.60674300	-0.60855200
C	4.60891300	-0.94360700	-1.19150400
C	5.60941600	-1.67524800	-1.84610000
C	5.52392300	-3.07325800	-1.92392300
C	4.43216900	-3.73724200	-1.34536900
C	3.42649800	-3.01191800	-0.69551000
C	-2.24895800	2.14237900	-0.39252000
C	-2.96564000	2.08393100	-1.60690200
C	-4.36363100	2.15842600	-1.59997500
C	-5.06034600	2.27004000	-0.38701400
C	-4.35157900	2.32195900	0.82082500
C	-2.95087100	2.25894500	0.82047100
H	4.15649200	3.97700200	0.72690300
H	2.92783800	2.21402400	-0.92668600
H	3.45774000	0.26713200	2.98675800
H	4.48250000	2.77774900	3.14656900
H	0.82980800	4.62904000	0.66786900
H	-0.07777300	0.71690800	2.47910300
H	1.63687400	4.69248200	3.26063300
H	1.07843400	2.27698900	4.37943500
H	1.59063400	-1.81513600	-3.29628000
H	-0.87571900	-3.28596900	-0.05074900
H	1.90163400	-4.13592700	-4.11289700
H	-0.48031600	-5.62059500	-0.81602000
H	0.90164000	-6.05989600	-2.85875700
H	-0.98667200	4.67887800	-1.08919900
H	1.34880500	1.38924400	-2.63909000
H	-0.09181100	6.16893200	-2.88236400
H	2.24553700	2.88006400	-4.45359700
H	1.52894400	5.27459000	-4.56064500
H	-2.42697400	1.97327600	-2.55291500
H	-2.40389900	2.28413800	1.76746600
H	-4.91331300	2.11021900	-2.54558500
H	-4.88888600	2.40214000	1.77164900
H	-6.15459900	2.30522800	-0.38453300
H	4.68828500	0.14612900	-1.12479200

H	2.56835600	-3.53662200	-0.26416500
H	6.46143000	-1.15002900	-2.29132600
H	4.35029300	-4.82708400	-1.41018500
H	6.30671000	-3.64371700	-2.43535400
H	3.80803900	-2.12236700	2.14123700
H	-0.39862000	-1.40518300	1.40129100
H	3.22802000	-3.40210200	4.20116200
H	-0.98073200	-2.68501200	3.45480600
H	0.83559000	-3.68231100	4.87127200
H	-3.99502400	-1.02158200	-2.75150500
H	-6.56968100	-1.52120400	1.49676700
H	-6.52416600	-1.09282400	-2.81054800
H	-7.79804700	-1.31718400	-0.66771300
C	-3.88432400	-1.57459200	1.89758000
F	-4.75330700	-1.64924800	2.94098200
F	-3.14260400	-2.71303900	1.93475000
F	-3.04001300	-0.54772100	2.18033600

transition state (*E*)-**TS3**

C	2.49803500	2.48064900	-4.27593100
C	3.64634000	2.21088200	-3.51426000
C	3.53051100	1.59736400	-2.25952900
C	2.26156100	1.24515300	-1.75976000
C	1.11195600	1.53449900	-2.52009100
C	1.23101700	2.14449900	-3.77680100
P	2.05021500	0.43068100	-0.11945800
C	3.36568000	1.22638100	0.89158300
C	4.46608100	0.52164900	1.41641700
C	5.40259800	1.18135200	2.22682000
C	5.25088500	2.54494900	2.51609000
C	4.15614400	3.25110600	1.99164900
C	3.21521000	2.59731700	1.18901100
C	2.73925300	-1.22852000	-0.42441300
C	2.64841200	-2.32942400	0.51076800
C	3.23623300	-3.48534300	-0.10163700
C	3.68426900	-3.11608100	-1.41465200
C	3.38183500	-1.72868500	-1.61817000
Fe	1.67802000	-2.76710300	-1.21309300
C	0.66874500	-3.10266300	-2.96089900
C	0.08167700	-2.02557100	-2.21943500
C	-0.31284100	-2.53349500	-0.92309800
C	0.04544400	-3.93244600	-0.87833100
C	0.64871200	-4.27650500	-2.13391000
P	-1.01917900	-1.47229800	0.37121000
C	-2.82052200	-1.53206000	0.03191200
C	-3.68455300	-0.95835100	0.98776800
C	-5.05822300	-0.87881400	0.73647000
C	-5.58141900	-1.35028700	-0.47715900
C	-4.72609300	-1.91495500	-1.43340600
C	-3.34964800	-2.00751300	-1.18311900
Pd	-0.17022200	0.70320500	0.49795200
C	-0.37023700	2.78189600	0.88844600
C	-0.15024500	3.08766600	2.24860300
C	0.41158100	4.32221200	2.60963700
C	0.71582200	5.27209500	1.62276200
C	0.43703400	4.99111300	0.26917700
C	-0.12083300	3.76275000	-0.09463600
C	-0.85038400	-2.47176000	1.90511300
C	-1.57062000	-3.66594100	2.10600400
C	-1.40580200	-4.39435600	3.29176700
C	-0.53017400	-3.93222400	4.28749300
C	0.17253600	-2.73233500	4.10244300
C	0.00826400	-2.00139700	2.91711500
H	3.29480800	-4.48076800	0.34208000
H	2.18857000	-2.29020000	1.49848500
H	3.56720600	-1.15071500	-2.52400200
H	4.14196000	-3.78365200	-2.14714600
H	-0.08587400	-4.59372200	-0.02131000
H	-0.02807200	-0.99247900	-2.55075800
H	1.05712100	-5.25368900	-2.39857500
H	1.09145700	-3.03022300	-3.96441800
H	-0.42138700	2.35447300	3.01667900
H	-0.38554900	3.55287900	-1.13665700
H	0.60310900	4.54624300	3.66509400
H	0.64971300	5.74245200	-0.49996600
H	1.14306300	6.24053400	1.90440000
H	-2.26958800	-4.01334300	1.33730000
H	0.53264500	-1.04934000	2.76335200
H	-1.96747500	-5.32244600	3.44381200
H	0.84148300	-2.35949700	4.88538300
H	-0.40679400	-4.50224800	5.21460800
H	-3.27247500	-0.55277300	1.91806500

H	-2.68377800	-2.44761800	-1.93227100
H	-5.72045000	-0.42110700	1.47736100
H	-5.13016500	-2.28155100	-2.38336100
H	-6.65325300	-1.26257600	-0.68090600
H	4.59268400	-0.54122700	1.18784500
H	2.36064300	3.15115300	0.78849200
H	6.25659800	0.62580100	2.62981000
H	4.02536400	4.31551900	2.21368500
H	5.98337500	3.05667900	3.14966700
H	4.42476500	1.39787300	-1.65891600
H	0.12266600	1.29949400	-2.10264700
H	4.63540300	2.48486400	-3.89727700
H	0.33098800	2.36883100	-4.35911000
H	2.59167900	2.96439000	-5.25422800
N	-1.95533900	1.77782500	0.69484800
N	-2.42775300	1.91436400	-0.47902600
C	-3.83573200	2.03895400	-0.55401800
C	-4.44067500	1.75170500	-1.79424200
C	-4.63659300	2.45754500	0.53360200
C	-5.82863700	1.85334400	-1.93876700
H	-3.80113100	1.43091300	-2.62225700
C	-6.01918200	2.57621500	0.37255200
H	-4.14486300	2.68509100	1.48371200
C	-6.62460300	2.26793100	-0.85914300
H	-6.29364300	1.61109900	-2.90083500
H	-6.63628300	2.91444900	1.21315100
H	-7.70965200	2.35962100	-0.97752700

transition state (*E*)-**TS3**$^{\mathbf{dtbpf}}$

P	1.46786400	1.59475800	-0.46048100
C	3.00738500	0.62438000	-0.17087700
C	3.21884900	0.10814000	1.16686400
C	4.50697600	-0.51193900	1.24006200
C	5.11127500	-0.40590400	-0.05482300
C	4.20406400	0.29345300	-0.91924700
Fe	3.31986600	-1.38895100	-0.16902600
C	3.31848800	-2.86205800	-1.58708400
C	2.02529800	-2.26163700	-1.44601800
C	1.56991800	-2.42524100	-0.07755000
C	2.62629200	-3.13383700	0.61270500
C	3.69377000	-3.39426000	-0.31142300
P	-0.07350000	-1.81208600	0.47304300
Pd	-0.52989300	0.54775900	0.26470700
C	-1.85606900	2.12836400	0.79132200
C	-1.75754300	2.45640600	2.16091700
C	-2.05118100	3.75981400	2.59426800
C	-2.48423300	4.72783300	1.67568400
C	-2.64624800	4.38240600	0.31885100
C	-2.35380900	3.08710700	-0.11406600
H	4.92955600	-1.01239100	2.11296100
H	2.48752300	0.16738300	1.96733600
H	4.39679500	0.50683300	-1.96711600
H	6.07989900	-0.81424500	-0.34999300
H	2.64048100	-3.40350900	1.66778300
H	1.48267000	-1.74660900	-2.23559300
H	4.63863400	-3.88401600	-0.06872100
H	3.92110100	-2.87559600	-2.49672300
H	-1.44699600	1.69287300	2.88264100
H	-2.54729700	2.77617500	-1.14826300
H	-1.95190400	4.01608700	3.65523200
H	-3.01784100	5.12737200	-0.39418500
H	-2.72505500	5.74031600	2.01663600
N	-2.61540900	0.41085200	0.50634600
N	-3.11629600	0.36965500	-0.66480400
C	-4.38653500	-0.24640000	-0.76571800
C	-4.82916600	-0.58346000	-2.06235700
C	-5.21175200	-0.52789700	0.34844400
C	-6.06344500	-1.21727700	-2.24176900
H	-4.17958900	-0.34491200	-2.91055200
C	-6.44840100	-1.14876900	0.15672700
H	-4.85631500	-0.24385100	1.34319300
C	-6.87843900	-1.50447100	-1.13456600
H	-6.39615800	-1.48508100	-3.25079300
H	-7.08995500	-1.35824800	1.02052000
H	-7.84797500	-1.99376800	-1.27633000
C	-0.21877600	-2.26895800	2.33176500
C	-0.09904000	-3.77109200	2.64767700
C	0.88183800	-1.47615600	3.06685900
C	-1.56683900	-1.73207200	2.86810300
H	0.82567800	-4.22059700	2.25304600
H	-0.10224100	-3.91008300	3.74564300
H	-0.95284300	-4.33718300	2.24263200

H	0.74971200	-0.39507700	2.87945800
H	0.78582800	-1.65235800	4.15436100
H	1.89827800	-1.76327800	2.75902700
H	-1.52222000	-1.71563300	3.97320200
H	-1.76723300	-0.71064900	2.50328300
H	-2.41929400	-2.36100000	2.57788800
C	-1.21732000	-2.99102000	-0.54066400
C	-2.59916100	-3.10598200	0.12954400
C	-1.40986500	-2.31701700	-1.91650400
C	-0.61501900	-4.39432500	-0.74093000
H	-2.56852700	-3.74268500	1.02849200
H	-3.00906400	-2.12322700	0.40465000
H	-3.30247800	-3.57220000	-0.58453000
H	-0.47112300	-2.28428800	-2.49176700
H	-2.13887800	-2.90508600	-2.50500200
H	-1.79723700	-1.29082800	-1.80876400
H	-1.32755300	-5.00495600	-1.32798500
H	0.33577200	-4.35588800	-1.29672700
H	-0.43401100	-4.91400400	0.21302600
C	2.05732000	3.25996600	0.33889800
C	0.93441500	4.31513300	0.35281800
C	2.36833300	2.95698200	1.82260800
C	3.31989400	3.80825800	-0.34750400
H	0.67914400	4.70032400	-0.64172400
H	0.02276300	3.91460900	0.81725700
H	1.27532200	5.17240000	0.96288800
H	3.28489900	2.36172500	1.94924400
H	2.51315200	3.91743200	2.35067000
H	1.52451900	2.43058600	2.30417700
H	3.68621100	4.69279000	0.20807400
H	4.13043000	3.05945000	-0.36427900
H	3.11854300	4.13040100	-1.38297800
C	1.23069300	1.85731000	-2.36098400
C	0.22792100	0.78181900	-2.82725100
C	0.56959200	3.22309100	-2.63171500
C	2.51135500	1.73936000	-3.20523100
H	0.61138900	-0.23256000	-2.65256600
H	-0.73834400	0.87216800	-2.29753800
H	0.04890800	0.89718900	-3.91355100
H	1.26201300	4.06496500	-2.47492700
H	0.25083500	3.24616400	-3.69057700
H	-0.32519700	3.37261000	-2.00667500
H	2.27724400	2.02783900	-4.24753200
H	3.32019200	2.39696800	-2.84819900
H	2.87786600	0.70097400	-3.22783300

transition state (*E*)-**TS3**$^{\mathbf{dppb}}$

C	4.38701900	-2.94673000	3.13542200
C	5.12700200	-1.91704200	2.53112300
C	4.47846400	-0.94720000	1.75611400
C	3.08136600	-1.00222900	1.57894600
C	2.34688300	-2.04075000	2.18084100
C	2.99721400	-3.00794700	2.96034800
P	2.16665400	0.25811500	0.60001300
C	3.27060300	0.56173100	-0.82899100
C	3.64820300	1.85346900	-1.24852500
C	4.38899800	2.02493300	-2.42758500
C	4.76034300	0.91349400	-3.19751100
C	4.38449300	-0.37559600	-2.78530600
C	3.63974700	-0.55250400	-1.61474900
C	2.35431700	1.79156500	1.64223500
C	-0.72344900	1.94855500	2.62418900
P	-1.02956300	1.58873200	0.80400100
C	-2.85770300	1.58818600	0.67771100
C	-3.55095100	2.33148400	-0.29834600
C	-4.93260500	2.16288700	-0.46303500
C	-5.63512500	1.24791600	0.33407100
C	-4.95046000	0.49741100	1.30092400
C	-3.57072800	0.65774400	1.46596100
Pd	0.02044800	-0.32167700	-0.04243900
C	0.19494900	-2.08327100	-1.20609400
C	0.59383000	-1.83435900	-2.53602900
C	1.39666700	-2.76472100	-3.21424400
C	1.78324900	-3.95445000	-2.57965600
C	1.33297800	-4.23026900	-1.27165400
C	0.52090900	-3.31473600	-0.59852700
C	-0.51334400	3.11629200	-0.08292300
C	-0.86289700	4.41119800	0.35208200
C	-0.41998900	5.53657800	-0.35527500
C	0.37351800	5.38035500	-1.50375200
C	0.71812400	4.09589700	-1.94818100
C	0.27467000	2.96890600	-1.24129300

H	0.27518100	-0.90947900	-3.02932500
H	0.11350900	-3.54544500	0.39217900
H	1.71959800	-2.55892700	-4.24119800
H	1.61094200	-5.17316100	-0.78646600
H	2.40812800	-4.68256000	-3.10809700
H	-1.49173400	4.54003100	1.24015100
H	0.54349200	1.95712000	-1.57150000
H	-0.69563300	6.53949900	-0.01176100
H	1.33729200	3.96611000	-2.84195100
H	0.72040400	6.26277800	-2.05215700
H	-3.00772000	3.03897700	-0.93301800
H	-3.03887500	0.03027600	2.19032300
H	-5.46104600	2.74651100	-1.22481700
H	-5.48657000	-0.24266800	1.90286300
H	-6.71090400	1.10467800	0.19138500
H	3.36041700	2.73248900	-0.66425100
H	3.33343100	-1.55859200	-1.30884800
H	4.67837400	3.03366500	-2.74243600
H	4.66289600	-1.24943400	-3.38386700
H	5.33827000	1.05046000	-4.11765300
H	5.05746700	-0.15082300	1.27495400
H	1.26385200	-2.08549700	2.01764700
H	6.21360700	-1.87301200	2.66272400
H	2.41882500	-3.81480800	3.42277600
H	4.89771800	-3.70534700	3.73828400
N	-1.53812300	-1.37385000	-0.92650700
N	-2.17160100	-2.08176500	-0.08144000
C	-3.56292500	-2.21339200	-0.32241400
C	-4.28988000	-2.98030500	0.61288400
C	-4.24367300	-1.60239500	-1.40066400
C	-5.67484100	-3.12935800	0.47929800
H	-3.73964500	-3.44321700	1.43850300
C	-5.62459800	-1.76335900	-1.52967700
H	-3.66495600	-1.00138300	-2.10716400
C	-6.34950100	-2.52235100	-0.59263400
H	-6.23213000	-3.72579400	1.21049000
H	-6.14976800	-1.28505100	-2.36450600
H	-7.43363600	-2.63833000	-0.69820000
H	3.41932900	2.08676100	1.66544400
H	1.80170800	2.59074800	1.11446000
H	-1.67951400	1.72321400	3.12647500
H	-0.54161100	3.03095400	2.75627400
C	1.83641100	1.62642700	3.08249600
H	2.51407100	0.93865000	3.62088600
H	1.92645400	2.60881200	3.58523500
C	0.39920600	1.10621500	3.25975900
H	0.20641500	1.04450600	4.34681000
H	0.32447700	0.07138300	2.87293600

transition state (*E*)-**TS3**[2c]

C	-6.65912700	1.55922100	-0.66120800
C	-5.87729600	1.26790100	-1.78326800
C	-4.48472600	1.31379700	-1.66454400
C	-3.88043600	1.62268700	-0.42884900
C	-4.69488900	1.91918800	0.68909200
C	-6.08581900	1.89654300	0.57198900
N	-2.46683300	1.63920800	-0.38880800
N	-1.95495900	1.54149000	0.77285300
Pd	-0.07921500	0.64793800	0.52316300
C	-0.47095500	2.69227800	0.94106700
C	-0.25022100	3.00424900	2.29991400
C	0.19644200	4.28398400	2.66407100
C	0.38373400	5.26937900	1.68290400
C	0.10283600	4.97631300	0.33233200
C	-0.34075700	3.70318700	-0.03478300
P	2.14387700	0.60615600	-0.14297400
C	2.98434500	-0.97262900	-0.49053200
C	3.02118000	-2.09082400	0.42754300
C	3.70426900	-3.17503500	-0.21630300
C	4.08477200	-2.74462700	-1.53203200
C	3.64508100	-1.39029600	-1.70579700
Fe	2.05924300	-2.59531300	-1.28268200
C	1.16166000	-4.18246500	-2.21063300
C	1.04698000	-2.99806600	-3.01450800
C	0.37470600	-1.99725500	-2.23929200
C	0.06251000	-2.56422700	-0.94498900
C	0.55737300	-3.92166100	-0.93567600
P	-0.71017100	-1.59941900	0.38599200
C	-0.40747800	-2.60303100	1.89638000
C	-0.99852200	-3.86773800	2.08764700
C	-0.73549700	-4.59659300	3.25522300
C	0.10968900	-4.06538200	4.24278400

C	0.68287400	-2.79708300	4.06795400
C	0.42030500	-2.06571500	2.90071100
C	2.24690000	1.46516000	-1.77087800
C	3.46733400	1.94852500	-2.28177500
C	3.50302000	2.59142200	-3.52651500
C	2.32208800	2.75979500	-4.26719200
C	1.10212500	2.29197800	-3.75701600
C	1.06292600	1.65217300	-2.51012700
C	3.39175400	1.50964100	0.86266700
C	4.55794300	0.90414500	1.36904800
C	5.43797300	1.63912500	2.17794600
C	5.16408300	2.97976000	2.48413100
C	4.00346500	3.58718000	1.97804400
C	3.11858900	2.85771100	1.17688900
C	-2.50645700	-1.82751400	0.09079000
C	-3.39544800	-1.37846100	1.08930000
C	-4.77704300	-1.42817300	0.87586300
C	-5.28614200	-1.90419600	-0.34217500
C	-4.40634400	-2.34349800	-1.34121800
C	-3.02081100	-2.30751200	-1.12876400
H	3.86878900	-4.16652500	0.20920000
H	2.58185300	-2.10994800	1.42506300
H	3.75346400	-0.78372900	-2.60537500
H	4.58823800	-3.35378600	-2.28509800
H	0.51252000	-4.60748500	-0.08916000
H	0.15675300	-0.97418200	-2.54716300
H	1.65756800	-5.10986800	-2.50338200
H	1.43661500	-2.86684200	-4.02540700
H	-0.43009900	2.23992000	3.06441700
H	-0.60692200	3.47980600	-1.07351500
H	0.38970200	4.51444600	3.71780900
H	0.22381400	5.75260500	-0.43189300
H	0.72079500	6.27198100	1.96724900
H	-1.67492100	-4.27072400	1.32594300
H	0.84260600	-1.06291300	2.75636800
H	-1.19675600	-5.57959700	3.39961300
H	1.32702600	-2.37201500	4.84503100
H	0.30999900	-4.63623200	5.15583600
H	-2.99904700	-0.97104800	2.02560400
H	-2.33652400	-2.65160300	-1.91087700
H	-5.45997700	-1.07098100	1.65262300
H	-4.79922000	-2.71344100	-2.29452400
H	-6.36705900	-1.92011200	-0.51471800
H	4.78010100	-0.13993300	1.12712700
H	2.21277900	3.33491100	0.79043400
H	6.34385900	1.16072800	2.56632400
H	3.77740900	4.63260700	2.21328500
H	5.85287200	3.55035300	3.11641700
H	4.38611200	1.82768400	-1.69747600
H	0.10832100	1.31327900	-2.08379600
H	4.45440100	2.96753600	-3.91816000
H	0.17571400	2.43617400	-4.32309800
H	2.35258600	3.26677700	-5.23767800
H	-3.83877400	1.08998100	-2.51858700
H	-4.20743100	2.16657500	1.63622300
H	-6.36573000	1.01079900	-2.72760900
H	-6.73805700	2.13496300	1.41796700
F	-8.00853000	1.51963800	-0.77018300

transition state (*E*)-**TS3[2d]**

C	-6.57665200	1.13390100	-0.73752200
C	-5.72755500	0.90562900	-1.83997200
C	-4.34898800	1.04522200	-1.70158200
C	-3.78225300	1.38818100	-0.45485700
C	-4.63682200	1.62650000	0.64171700
C	-6.02345300	1.51258700	0.50384200
N	-2.37394000	1.49214400	-0.38888600
N	-1.87362300	1.42334200	0.77832300
Pd	0.05383700	0.62341200	0.53604300
C	-0.44461500	2.64259100	0.95731700
C	-0.24494300	2.95919900	2.31892700
C	0.13508500	4.25784900	2.69148400
C	0.27494700	5.25770700	1.71686400
C	0.01221800	4.95783600	0.36406500
C	-0.36394700	3.66569800	-0.01167500
P	2.26900800	0.70297200	-0.14371500
C	3.18843100	-0.82786300	-0.50857800
C	3.29463100	-1.94806000	0.40164500
C	4.02780700	-2.99043700	-0.25628600
C	4.37069800	-2.53255800	-1.57313000
C	3.85738200	-1.20265100	-1.73328200
Fe	2.34306100	-2.49393900	-1.30178500

C	1.52268900	-4.12184300	-2.22989700
C	1.33580400	-2.94090400	-3.02544400
C	0.61831000	-1.98237300	-2.23741500
C	0.35050400	-2.57247600	-0.94357700
C	0.91809800	-3.90101700	-0.94744300
P	-0.45995800	-1.65597200	0.39980800
C	-0.08850200	-2.65113900	1.90088500
C	-0.60978900	-3.94582900	2.09402800
C	-0.29395400	-4.66420300	3.25507600
C	0.53479800	-4.09309100	4.23428900
C	1.03838000	-2.79574000	4.05782900
C	0.72272200	-2.07496700	2.89720200
C	2.31587200	1.57360900	-1.76836000
C	3.50499800	2.12446100	-2.28466100
C	3.49794400	2.77367900	-3.52664400
C	2.30495700	2.88098500	-4.25933600
C	1.11541500	2.34567500	-3.74365400
C	1.11879700	1.69958000	-2.49939100
C	3.47882900	1.66359900	0.85599600
C	4.68584900	1.12080000	1.33730700
C	5.53396100	1.89548000	2.14330600
C	5.18747000	3.21397700	2.47149400
C	3.98583100	3.75897900	1.99067900
C	3.13256200	2.98931600	1.19283600
C	-2.24357400	-1.97989400	0.11672000
C	-3.14841200	-1.57050200	1.11788100
C	-4.52698800	-1.68342700	0.90940000
C	-5.01870700	-2.18372800	-0.30590000
C	-4.12344300	-2.58666300	-1.30637700
C	-2.74034400	-2.48754000	-1.09906100
H	4.24969100	-3.97416900	0.16115400
H	2.86785400	-1.99750100	1.40364400
H	3.92344300	-0.58550900	-2.62981100
H	4.89793600	-3.10944500	-2.33532100
H	0.91849800	-4.59295100	-0.10467200
H	0.34098000	-0.97129600	-2.53709000
H	2.06500500	-5.01943500	-2.53320300
H	1.70731300	-2.78309200	-4.03937300
H	-0.38826500	2.18272400	3.07902600
H	-0.61488600	3.43534400	-1.05269200
H	0.31351400	4.49144200	3.74726400
H	0.09484200	5.74353900	-0.39577200
H	0.55988000	6.27450300	2.00784400
H	-1.27344000	-4.38092900	1.33877300
H	1.08981500	-1.05085100	2.75101500
H	-0.70079200	-5.67080500	3.40086500
H	1.66946900	-2.34038000	4.82848100
H	0.77664600	-4.65618300	5.14213300
H	-2.76767900	-1.14118400	2.05086500
H	-2.04402700	-2.80067900	-1.88354500
H	-5.22230000	-1.35371200	1.68733900
H	-4.50276900	-2.97413400	-2.25816800
H	-6.09827400	-2.24328300	-0.47750100
H	4.96434300	0.09443300	1.07838700
H	2.19412300	3.41711900	0.82714600
H	6.47186700	1.46572500	2.51217800
H	3.70263300	4.78625700	2.24346400
H	5.85152200	3.81565200	3.10149700
H	4.43270000	2.05078100	-1.70662100
H	0.18634300	1.30886600	-2.06824200
H	4.42509100	3.20250000	-3.92233700
H	0.17874400	2.44207000	-4.30300700
H	2.30175000	3.39308100	-5.22765000
H	-3.67738500	0.86742200	-2.54697300
H	-4.18783700	1.90475700	1.59950200
H	-6.17966300	0.61675000	-2.79347600
H	-6.66599200	1.71690100	1.36486600
O	-7.91522700	0.96618800	-0.96812000
C	-8.81281700	1.18472800	0.10883600
H	-9.82043700	0.99245100	-0.28919200
H	-8.61408500	0.49370500	0.95174100
H	-8.75863300	2.22687500	0.47993600

transition state (*E*)-**TS3**^{2e}

C	6.30758800	1.01252400	0.39389700
C	5.53140300	0.79600000	1.54594900
C	4.14958600	0.98447600	1.50231200
C	3.52080800	1.36256100	0.29804000
C	4.30811900	1.60764900	-0.85184500
C	5.68996800	1.43736700	-0.79892400
N	2.11408000	1.48109100	0.31952900
N	1.55264500	1.40761300	-0.82304900

Pd	-0.35410900	0.63679700	-0.51777600
C	0.12776000	2.66270800	-0.94275400
C	-0.10829000	2.99910200	-2.29220700
C	-0.48694600	4.30693200	-2.63350000
C	-0.59064500	5.29038300	-1.63816100
C	-0.29395900	4.96831800	-0.29778600
C	0.08381400	3.66736800	0.04608000
P	-2.56641500	0.69382500	0.18152200
C	-3.47981300	-0.84171000	0.53705300
C	-3.59244200	-1.95509000	-0.38110400
C	-4.31611600	-3.00404700	0.27659700
C	-4.64659000	-2.55731600	1.60020600
C	-4.13527000	-1.22755400	1.76575800
Fe	-2.62133700	-2.51031600	1.30804100
C	-1.78665300	-4.14739200	2.20847700
C	-1.59804800	-2.97648000	3.01793400
C	-0.89122600	-2.00448900	2.23706100
C	-0.63257700	-2.57623800	0.93305600
C	-1.19450400	-3.90729100	0.92397100
P	0.16214200	-1.64242800	-0.40630400
C	-0.24371300	-2.59482100	-1.92490400
C	0.26164100	-3.88973000	-2.15551000
C	-0.07741600	-4.57682900	-3.32879900
C	-0.91280500	-3.97392800	-4.28293000
C	-1.40009000	-2.67597300	-4.06930200
C	-1.06173500	-1.98643300	-2.89625000
C	-2.60166400	1.55170000	1.81215100
C	-3.78841600	2.09593700	2.34086000
C	-3.77356900	2.73580300	3.58754700
C	-2.57552500	2.84033900	4.31235000
C	-1.38851100	2.31134200	3.78454800
C	-1.39981000	1.67427200	2.53572900
C	-3.77586800	1.66530600	-0.80708200
C	-4.96732900	1.11693400	-1.31933400
C	-5.81608100	1.89962400	-2.11683400
C	-5.48557300	3.23131300	-2.40541400
C	-4.29969500	3.78199700	-1.89302400
C	-3.44579800	3.00474700	-1.10328400
C	1.94873500	-1.98127000	-0.16753800
C	2.83340500	-1.59637200	-1.19634700
C	4.21493800	-1.73374300	-1.02402200
C	4.73105800	-2.23410600	0.18127800
C	3.85513800	-2.61388000	1.20812600
C	2.46879000	-2.48985100	1.03821200
H	-4.53974800	-3.98510300	-0.14607700
H	-3.17641100	-1.99646600	-1.38797100
H	-4.19390200	-0.61786700	2.66785300
H	-5.16426800	-3.14150400	2.36330500
H	-1.19991800	-4.58815300	0.07233000
H	-0.61496100	-0.99687200	2.54891500
H	-2.32267600	-5.05116500	2.50432700
H	-1.96143000	-2.83398600	4.03702700
H	0.00503800	2.23276500	-3.06727100
H	0.36237200	3.41772800	1.07554100
H	-0.69235500	4.56024000	-3.67956900
H	-0.35001500	5.74267200	0.47574400
H	-0.87523000	6.31396300	-1.90459500
H	0.93222000	-4.34912100	-1.42112200
H	-1.41655300	-0.96244600	-2.72131300
H	0.31729700	-5.58344100	-3.50409400
H	-2.03584700	-2.19565300	-4.82064000
H	-1.17215000	-4.51251500	-5.20067400
H	2.43571300	-1.17114600	-2.12417000
H	1.78838300	-2.78666100	1.84284000
H	4.89656000	-1.42514000	-1.82237700
H	4.25259600	-3.00391700	2.15146700
H	5.81392500	-2.30471500	0.32097000
H	-5.23388200	0.08028800	-1.09074200
H	-2.52012700	3.43783700	-0.71170200
H	-6.74202000	1.46564300	-2.50991700
H	-4.03009000	4.82008800	-2.11434400
H	-6.15011700	3.83930400	-3.02865700
H	-4.72013900	2.02456500	1.76910200
H	-0.47045200	1.28640400	2.09591400
H	-4.69869900	3.15940700	3.99323800
H	-0.44845000	2.40495000	4.33855300
H	-2.56652400	3.34496400	5.28445500
H	3.52516200	0.81338000	2.38383200
H	3.80493700	1.92103000	-1.77043900
H	6.01606900	0.47603300	2.47303000
H	6.30396000	1.62804700	-1.68471500
C	7.77529200	0.69999700	0.41180400

F	8.47607500	1.43959000	-0.48301600
F	8.01570200	-0.61077600	0.09392300
F	8.33425600	0.89999200	1.63177300

transition state (*E*)-**TS3**^{2f}

C	-6.22852600	-2.28605500	1.13027900
C	-5.27353800	-1.91406900	2.09004300
C	-3.94251000	-1.77424800	1.70225900
C	-3.52454300	-1.98035800	0.36893000
C	-4.50368200	-2.33821700	-0.58332800
C	-5.83922500	-2.49914000	-0.20302300
N	-2.15043800	-1.85254200	0.10142400
N	-1.81415200	-1.64906500	-1.10876600
Pd	-0.02284800	-0.59641300	-0.80926800
C	-0.17441900	-2.61752900	-1.45885500
C	0.09830300	-2.72363500	-2.83849600
C	0.70566700	-3.88551100	-3.34208600
C	0.98875000	-4.95956900	-2.48396300
C	0.64467900	-4.87929700	-1.11918500
C	0.04710600	-3.72137200	-0.61260800
P	2.06815600	-0.42696000	0.17628000
C	2.65551500	1.17825400	0.80668600
C	2.69697200	2.38966500	0.01725900
C	3.15086600	3.45697000	0.86059100
C	3.38441800	2.92135300	2.17228900
C	3.08146300	1.51933200	2.14485800
Fe	1.44681100	2.60251600	1.59934500
C	0.25726800	3.98429900	2.52597300
C	0.15830000	2.71264200	3.18576800
C	-0.28291000	1.74446700	2.22570300
C	-0.46712600	2.42058000	0.95970600
C	-0.12713000	3.81094300	1.15436600
P	-0.92793500	1.54138400	-0.56031900
C	-0.59826100	2.75896400	-1.89535900
C	-1.34055200	3.94926900	-2.02867100
C	-1.04655500	4.84717100	-3.06362400
C	-0.01670000	4.56090500	-3.97436200
C	0.71285500	3.36849600	-3.85748900
C	0.41854000	2.46717500	-2.82444700
C	1.96440600	-1.42789200	1.72354200
C	3.11643700	-1.86176000	2.40816200
C	2.99301100	-2.59714700	3.59536900
C	1.72172500	-2.90557800	4.10610500
C	0.57189300	-2.48892300	3.41925200
C	0.69128600	-1.76009000	2.22768200
C	3.57022500	-1.09537900	-0.65101600
C	4.79738400	-0.40509800	-0.68830200
C	5.89677100	-0.95975400	-1.36097900
C	5.78145200	-2.20447700	-1.99643000
C	4.55929900	-2.89507300	-1.96138600
C	3.45703200	-2.34298000	-1.29933000
C	-2.75929900	1.47472500	-0.47633600
C	-3.45236600	1.05307900	-1.63094800
C	-4.83503800	0.85077900	-1.58680100
C	-5.53865000	1.04045300	-0.38664200
C	-4.85280000	1.44428700	0.76588300
C	-3.46791700	1.66356100	0.72461700
H	3.25981800	4.50162800	0.56377700
H	2.40800200	2.47504400	-1.03042800
H	3.12622200	0.82883600	2.98753600
H	3.70028600	3.48911700	3.04955700
H	-0.12672400	4.57851300	0.37977300
H	-0.44203600	0.68001600	2.40093000
H	0.60269900	4.91585100	2.97834200
H	0.41029900	2.50707500	4.22747600
H	-0.15621900	-1.89457300	-3.50889400
H	-0.25839600	-3.65606600	0.43809700
H	0.94613500	-3.95720500	-4.40890300
H	0.84042400	-5.72873000	-0.45484800
H	1.44908500	-5.87138300	-2.87953200
H	-2.15485800	4.16164400	-1.32731300
H	0.96250200	1.51864500	-2.73004000
H	-1.62604800	5.77126000	-3.16460800
H	1.50498300	3.13494000	-4.57683500
H	0.20825200	5.26385600	-4.78366100
H	-2.89975800	0.87017400	-2.55880900
H	-2.93581500	1.97149500	1.62969300
H	-5.36450700	0.51988900	-2.48638000
H	-5.39521300	1.58224100	1.70707100
H	-6.61649900	0.85427700	-0.34713800
H	4.89197100	0.56519600	-0.19031200
H	2.50395400	-2.87943000	-1.28360800

H	6.84785900	-0.41642500	-1.38544700
H	4.45700600	-3.86555100	-2.45859000
H	6.64149200	-2.63449100	-2.52114700
H	4.10868300	-1.62941000	2.00568600
H	-0.21203300	-1.47774900	1.66784300
H	3.89246100	-2.93465100	4.12197200
H	-0.42661100	-2.73517500	3.79576800
H	1.63007800	-3.48178900	5.03331000
H	-5.54399800	-1.73032000	3.13397300
H	-6.58550900	-2.79195600	-0.94932500
H	-7.27488500	-2.40867500	1.42807500
F	-3.02408500	-1.40045200	2.62406800
H	-4.17286500	-2.49287400	-1.61424000

transition state (*E*)-**TS3**[2g]

C	-6.08015900	-2.47278400	0.73540800
C	-5.22737000	-1.87686600	1.67993500
C	-3.86466700	-1.70508000	1.38783200
C	-3.35876200	-2.12964800	0.12743000
C	-4.23176800	-2.70677200	-0.81223000
C	-5.58608700	-2.88850000	-0.50896300
N	-1.97613900	-1.97251100	-0.08734600
N	-1.59546700	-1.78246900	-1.28166200
Pd	0.12052200	-0.61686300	-0.89450700
C	0.12855300	-2.65149400	-1.52120100
C	0.49002900	-2.76107000	-2.88031500
C	1.18634900	-3.89562500	-3.32849100
C	1.46993800	-4.94441200	-2.43981000
C	1.03751200	-4.86589700	-1.10028500
C	0.35325000	-3.73371400	-0.64788700
P	2.13046400	-0.34331000	0.22579000
C	2.60736100	1.29515700	0.86662000
C	2.63085700	2.49737900	0.06266300
C	2.98516100	3.59698400	0.91212200
C	3.17573400	3.09032300	2.24214600
C	2.94517400	1.67428800	2.21977800
Fe	1.28993600	2.66472900	1.57362800
C	-0.02146800	3.97953800	2.42935700
C	-0.08962700	2.70183300	3.08283200
C	-0.42375700	1.71471900	2.09838300
C	-0.56920800	2.38308200	0.82385600
C	-0.31748100	3.78929600	1.03775200
P	-0.91600300	1.47782200	-0.71265400
C	-0.57958900	2.71401100	-2.03098400
C	-1.37716700	3.86124700	-2.21178100
C	-1.07382300	4.77331500	-3.23169400
C	0.02105800	4.54380400	-4.08049000
C	0.80700100	3.39333900	-3.91705700
C	0.50329300	2.47790800	-2.89936000
C	1.97582700	-1.32650400	1.78185900
C	3.10222900	-1.68602600	2.54756900
C	2.94270200	-2.40991600	3.73751600
C	1.65972000	-2.78283500	4.16948900
C	0.53707400	-2.44168100	3.40081900
C	0.69194700	-1.72316300	2.20677300
C	3.71608500	-0.94889800	-0.48824100
C	4.91026400	-0.20284900	-0.44623500
C	6.07886700	-0.71036100	-1.03397500
C	6.06603100	-1.96330900	-1.66363600
C	4.87716700	-2.70908400	-1.70837400
C	3.70655400	-2.20434100	-1.13110800
C	-2.74774200	1.35080100	-0.69462500
C	-3.36548100	0.70983700	-1.78845400
C	-4.74515700	0.47655900	-1.77994400
C	-5.51960800	0.86496500	-0.67659400
C	-4.91003100	1.50169400	0.41350900
C	-3.52940100	1.74418900	0.40848900
H	3.05696100	4.64198700	0.60540100
H	2.39235100	2.55506400	-0.99950700
H	2.97961200	0.99772600	3.07418500
H	3.41672800	3.68417200	3.12592900
H	-0.31469900	4.55771500	0.26390700
H	-0.53161900	0.64159900	2.25622600
H	0.24713800	4.92693000	2.90067400
H	0.11682600	2.50756300	4.13686600
H	0.23486200	-1.95529200	-3.57839700
H	-0.01809100	-3.67227500	0.38162400
H	1.49569600	-3.96645800	-4.37754700
H	1.23331700	-5.69671100	-0.41268500
H	1.99940400	-5.83580100	-2.79321000
H	-2.24136500	4.02867500	-1.55952200
H	1.09110600	1.55996000	-2.77080400

H	-1.69691000	5.66365500	-3.36996000
H	1.65075200	3.20302700	-4.58894800
H	0.25312700	5.25726900	-4.87853100
H	-2.75561500	0.36824900	-2.63121800
H	-3.05406500	2.23135900	1.26531000
H	-5.21437200	-0.03599100	-2.62560200
H	-5.51237300	1.80605500	1.27668500
H	-6.59375600	0.65525000	-0.65999300
H	4.92506100	0.77437400	0.04692200
H	2.78057100	-2.78418100	-1.17818700
H	7.00336300	-0.12352400	-0.99677800
H	4.85426500	-3.68627700	-2.20241900
H	6.97992400	-2.35652700	-2.12205900
H	4.10455900	-1.40516900	2.20549700
H	-0.18887100	-1.50173800	1.58618000
H	3.82305100	-2.68893100	4.32691900
H	-0.46754300	-2.74517100	3.71448300
H	1.53918800	-3.35177300	5.09796600
H	-5.63373800	-1.54384600	2.63823900
H	-6.25433200	-3.35598700	-1.23985000
H	-7.13846300	-2.60819800	0.98266600
H	-3.80907500	-3.01515500	-1.77298000
O	-2.96390800	-1.11287600	2.22702200
C	-3.43877600	-0.58515000	3.45550800
H	-4.20565400	0.19488600	3.28664900
H	-3.86155900	-1.37705700	4.10388900
H	-2.56417400	-0.13621400	3.94931800

transition state (*E*)-**TS3**$^{\mathbf{2h}}$

C	-6.55581500	-1.36648600	-0.31759300
C	-5.81373400	-1.25204700	0.86491900
C	-4.41268400	-1.30950700	0.83447500
C	-3.73715000	-1.45686900	-0.40482700
C	-4.49557400	-1.57343500	-1.59121200
C	-5.88940400	-1.53844100	-1.54331600
N	-2.33345500	-1.51077300	-0.37302200
N	-1.73310000	-1.26403000	-1.47095700
Pd	0.10403400	-0.50112600	-0.86239700
C	-0.23778600	-2.46251400	-1.62051100
C	0.20785700	-2.60338800	-2.95045400
C	0.68545100	-3.84588200	-3.39802200
C	0.67763400	-4.95348700	-2.53649100
C	0.16973400	-4.82453000	-1.22724400
C	-0.30755500	-3.59005700	-0.77808300
P	2.16584000	-0.62718500	0.19951600
C	2.95272200	0.85946400	0.89762500
C	3.19593000	2.07897500	0.15696100
C	3.76328100	3.04115300	1.05654400
C	3.86632600	2.43406600	2.35349500
C	3.36950200	1.09177200	2.26152000
Fe	1.92718700	2.41558600	1.70003800
C	0.90971000	3.93490200	2.61687500
C	0.61071900	2.67925600	3.24601400
C	0.07307800	1.79794200	2.25267800
C	0.03080400	2.51273800	0.99520400
C	0.55462200	3.83896200	1.23012600
P	-0.49307300	1.73669200	-0.55952900
C	0.12211400	2.87873900	-1.86182300
C	-0.39322600	4.18010700	-2.02280900
C	0.10951500	5.01451100	-3.03029100
C	1.12101200	4.55373100	-3.88832700
C	1.62218000	3.25114500	-3.74679900
C	1.11985300	2.41433800	-2.74010700
C	1.91055200	-1.67255800	1.69449300
C	2.98734000	-2.27424900	2.37499600
C	2.74869100	-3.05014000	3.51743800
C	1.43689800	-3.23294400	3.98479400
C	0.36028800	-2.64747700	3.30272000
C	0.59583200	-1.87454000	2.15702000
C	3.56634600	-1.46325300	-0.65117700
C	4.82624000	-0.86120800	-0.83250400
C	5.83261900	-1.53503700	-1.54125600
C	5.59177200	-2.81132400	-2.06952300
C	4.33726200	-3.41567100	-1.88751700
C	3.32766300	-2.74592300	-1.18817300
C	-2.30725000	2.00257700	-0.58875400
C	-2.99816500	1.65167800	-1.76733400
C	-4.39511200	1.71097500	-1.80643500
C	-5.11722700	2.09855400	-0.66698100
C	-4.43436800	2.44676600	0.50634900
C	-3.03377700	2.40319200	0.54762300
H	4.03131400	4.06857400	0.80396100

H	2.96329900	2.24299000	-0.89548100
H	3.28266500	0.37423500	3.07793700
H	4.22438400	2.92101800	3.26250700
H	0.69135500	4.61116800	0.47250100
H	-0.24200100	0.76563600	2.40269700
H	1.36530800	4.80151800	3.09971900
H	0.79460800	2.42381900	4.29091900
H	0.18636400	-1.74040400	-3.62559100
H	-0.75591400	-3.48206500	0.21630200
H	1.05580300	-3.95092100	-4.42398600
H	0.13826800	-5.69654300	-0.56417800
H	1.03946800	-5.92533300	-2.88923700
H	-1.19733300	4.52990500	-1.36615000
H	1.48408500	1.38498600	-2.62666100
H	-0.29354800	6.02578000	-3.15190500
H	2.39783100	2.88253000	-4.42648000
H	1.50882100	5.20751900	-4.67689700
H	-2.43795700	1.30962800	-2.64403900
H	-2.50460200	2.66203700	1.46939400
H	-4.92530300	1.42893300	-2.72146400
H	-4.99338900	2.74541400	1.39962700
H	-6.21153500	2.11641400	-0.69211900
H	5.02131900	0.13202500	-0.41601700
H	2.35018600	-3.21928000	-1.05440100
H	6.81054900	-1.05996500	-1.67645100
H	4.13698900	-4.41100400	-2.29813400
H	6.37944200	-3.33425100	-2.62263600
H	4.00906500	-2.14016400	2.00288000
H	-0.24629700	-1.44322500	1.59969900
H	3.58850600	-3.51843900	4.04238000
H	-0.66826100	-2.79697700	3.64472200
H	1.25466000	-3.84336600	4.87598100
H	-3.95148500	-1.69509500	-2.53175900
H	-6.31948100	-1.12323900	1.82527100
H	-6.46720300	-1.64304100	-2.46868100
H	-7.64921000	-1.33092200	-0.28091200
C	-3.62974200	-1.23886600	2.12230900
F	-4.44208400	-1.02906200	3.19233400
F	-2.95016800	-2.39133500	2.37977900
F	-2.70982300	-0.23589000	2.14392500

transition state (*Z*)-**TS3**

C	-3.51708400	1.07571300	4.01617900
C	-4.19131400	0.01836700	3.38314200
C	-3.66863800	-0.55216500	2.21499900
C	-2.45800600	-0.07343500	1.67517300
C	-1.79688000	0.99500500	2.30651300
C	-2.32067100	1.56673800	3.47437700
P	-1.72626500	-0.78149100	0.13691100
C	-3.23335400	-1.07398500	-0.87825300
C	-3.58737100	-2.33800100	-1.38802200
C	-4.71169600	-2.47362500	-2.21618100
C	-5.49141800	-1.35376200	-2.53942500
C	-5.14214100	-0.09224100	-2.03099300
C	-4.01832800	0.05015900	-1.21032400
C	-1.27320400	-2.46668100	0.66343900
C	-0.49445200	-3.37766800	-0.14800600
C	-0.25776000	-4.56763000	0.61660200
C	-0.87462700	-4.40156400	1.90226800
C	-1.50170100	-3.11154300	1.93606500
Fe	0.48884900	-2.91467300	1.55989100
C	1.41536600	-2.28358500	3.27097200
C	1.25777500	-1.20906900	2.33513200
C	1.93572600	-1.57436300	1.10955300
C	2.50596700	-2.88737600	1.30575800
C	2.18197700	-3.31856300	2.63569800
P	1.91944300	-0.51671100	-0.36847900
C	3.39933500	0.54301100	-0.12210600
C	3.75712700	1.41967800	-1.16813800
C	4.79735200	2.33823000	-0.98893100
C	5.48193400	2.40084700	0.23444600
C	5.12929800	1.53200400	1.27737100
C	4.09222800	0.60527100	1.10304100
Pd	-0.08599300	0.60816900	-0.75524900
C	-1.16059100	2.33810200	-1.48278100
C	-1.75826200	2.13167600	-2.74908800
C	-2.99176400	2.71317200	-3.06551100
C	-3.64117900	3.53204700	-2.12855400
C	-3.05094800	3.75654300	-0.87076100
C	-1.82119000	3.17366800	-0.54321200
C	2.43290600	-1.63351900	-1.73477800
C	3.72432300	-2.19314100	-1.79900800

C	4.06875600	-3.04294900	-2.85856400
C	3.13272000	-3.33001300	-3.86517600
C	1.85382400	-2.75564400	-3.81906800
C	1.50687900	-1.90515700	-2.75976300
H	0.32407300	-5.43058000	0.28812300
H	-0.12628300	-3.17622500	-1.15425100
H	-2.02825700	-2.66968800	2.78240500
H	-0.84193700	-5.11712800	2.72606500
H	3.05078100	-3.46086600	0.55527900
H	0.69609400	-0.28760400	2.49343900
H	2.44162300	-4.28406100	3.07397700
H	0.99194600	-2.32123400	4.27605500
H	-1.21530400	1.53296200	-3.48789100
H	-1.38812300	3.34915600	0.44336900
H	-3.44358500	2.53320900	-4.04714300
H	-3.55956200	4.38749400	-0.13298100
H	-4.60193400	3.99721700	-2.37434100
H	4.46083800	-1.94837300	-1.02585500
H	0.51946400	-1.42726000	-2.71772500
H	5.07358400	-3.47664500	-2.90483100
H	1.12890600	-2.96080900	-4.61391800
H	3.40706600	-3.99099800	-4.69434200
H	3.20636300	1.39451000	-2.11346200
H	3.81513000	-0.06755600	1.92074700
H	5.05844700	3.02233400	-1.80283700
H	5.66171200	1.57662700	2.23374500
H	6.28641700	3.13036700	0.37644700
H	-2.98843500	-3.21745000	-1.13132700
H	-3.74653800	1.03793100	-0.82323000
H	-4.98131900	-3.46166200	-2.60539200
H	-5.74124200	0.79000400	-2.28045200
H	-6.36877800	-1.46303000	-3.18597400
H	-4.20398500	-1.36458400	1.71157600
H	-0.87912600	1.38776200	1.85544900
H	-5.13263000	-0.35773600	3.79850000
H	-1.79858300	2.40448800	3.94906600
H	-3.93302200	1.52317500	4.92536100
N	0.58750900	2.32511100	-1.83257600
N	1.28566800	3.27736200	-1.37934000
C	1.19644300	3.76590000	-0.05985200
C	1.23584300	5.16350900	0.13377000
C	1.15871500	2.91352300	1.06368200
C	1.17248600	5.69391500	1.42602700
H	1.29452500	5.80919200	-0.74787500
C	1.12161200	3.45028400	2.35525500
H	1.16803600	1.82817400	0.89637800
C	1.11409500	4.84167400	2.54299900
H	1.17428200	6.78025100	1.56753000
H	1.11093200	2.77858600	3.22159400
H	1.08347800	5.26135600	3.55416000

transition state **TS4**

Si	0.19892900	4.32966500	0.75934200
Br	1.64927100	3.38956200	-0.96791500
N	0.92837200	1.72862600	1.38354700
N	1.30464600	1.59804900	2.55605600
C	2.46141700	0.83339900	2.90625900
C	3.68838800	1.09123400	2.25835200
C	2.40831800	-0.05027300	3.99902600
C	4.85951700	0.47392100	2.71919400
H	3.71586900	1.80966200	1.43140100
C	3.57487000	-0.69943900	4.41846500
H	1.44798900	-0.23266700	4.49049300
C	4.80563600	-0.42764300	3.79327900
H	5.81416000	0.69145600	2.22853900
H	3.52764000	-1.41463100	5.24651100
H	5.71948400	-0.91715900	4.14600400
C	2.89672600	0.37473200	-1.97409800
C	2.48963700	0.65286000	-3.29676000
C	4.20830000	-0.11273000	-1.78919300
C	3.34844100	0.43981400	-4.38881500
H	1.48685400	1.04932900	-3.49115900
C	5.07514900	-0.32302500	-2.87382300
H	4.56584200	-0.34261300	-0.77801800
C	4.64419500	-0.05544500	-4.18128100
H	3.00342400	0.66711700	-5.40482300
H	6.08681500	-0.70621800	-2.69503000
H	5.31372800	-0.22645400	-5.03169000
Pd	1.82765900	0.89092600	-0.27738300
P	1.40230500	-1.29225000	0.05715800
C	0.19608900	-1.94027100	-1.13556400
C	-1.23324600	1.02487200	-2.36680800

C	-1.01882100	-2.64520800	-0.79779000
C	0.25832600	-1.84018100	-2.57701800
Fe	-1.42141800	-0.95357900	-1.87273100
C	-2.27540500	0.40197400	-3.13291700
C	-1.55240600	0.87271500	-0.98050900
C	-1.69813800	-2.97518800	-2.01384800
H	-1.35803000	-2.86914200	0.21090300
C	-0.90667900	-2.48831700	-3.10975900
H	1.04749200	-1.34075700	-3.13959300
C	-3.24304300	-0.13492900	-2.21926400
C	-2.80383300	0.15822000	-0.87165300
H	-2.30705300	0.31645000	-4.22078200
H	-0.93014400	1.22031400	-0.15473300
H	-2.66426800	-3.47826000	-2.08158500
H	-1.16370300	-2.55895100	-4.16818600
H	-4.14120000	-0.69004100	-2.49027000
C	0.60175000	-1.75857900	1.64973000
C	0.98610400	-2.89713300	2.38549300
C	-0.47095800	-0.97280900	2.11008200
C	0.32455300	-3.22104500	3.57761500
H	1.80336100	-3.52977300	2.02869000
C	-1.13847300	-1.30535700	3.29540600
H	-0.79234100	-0.09743200	1.54376000
C	-0.73430200	-2.42376800	4.03944400
H	0.63672500	-4.10404300	4.14560400
H	-1.97707500	-0.68427400	3.62489700
H	-1.24747100	-2.67846700	4.97300100
C	2.84058600	-2.41716800	-0.06035500
C	3.02607800	-3.28870900	-1.14743300
C	3.83396200	-2.32128000	0.93430800
C	4.19807500	-4.05272000	-1.23847700
H	2.26277900	-3.36176600	-1.92673400
C	4.99788800	-3.09241900	0.84305700
H	3.69791100	-1.63776200	1.77625700
C	5.18548900	-3.95599100	-0.24771500
H	4.34059900	-4.72335300	-2.09231300
H	5.76253000	-3.00895400	1.62274300
H	6.10120000	-4.55174600	-0.32592400
C	0.02205000	5.98790000	-0.14984900
H	0.99899800	6.47666000	-0.31187500
H	-0.46966100	5.86740700	-1.13174500
H	-0.60315800	6.66707700	0.46251300
C	1.15313100	4.66006200	2.34197800
H	2.20527500	4.90426400	2.11244900
H	0.69894600	5.54359100	2.83002500
H	1.12455600	3.80074200	3.03133900
C	-1.54402700	3.64424100	0.93292200
H	-1.97399300	3.39263100	-0.05076700
H	-1.58539800	2.75673400	1.58153000
H	-2.16805900	4.44800400	1.37109500
H	-0.33712700	1.50953900	-2.75337000
P	-3.63631100	-0.16791900	0.72322200
C	-5.11950000	0.92310600	0.55563100
C	-6.23036600	0.69624100	1.39699700
C	-5.13731600	2.03844300	-0.30531100
C	-7.33764800	1.55228000	1.36296000
H	-6.23017200	-0.16470700	2.07590100
C	-6.24352100	2.90096400	-0.32922900
H	-4.28493700	2.22808600	-0.96538500
C	-7.34740900	2.66020400	0.50042600
H	-8.19622300	1.35593200	2.01457100
H	-6.24314800	3.76219200	-1.00647300
H	-8.21223400	3.33174200	0.47652700
C	-4.38381400	-1.83537700	0.41482700
C	-3.82675000	-2.93600500	1.09568800
C	-5.45183300	-2.05569200	-0.47885000
C	-4.29407700	-4.23632700	0.85460000
H	-3.02005600	-2.76719800	1.81809900
C	-5.92036500	-3.35444400	-0.71817000
H	-5.92347700	-1.20311000	-0.97921900
C	-5.33635300	-4.44848400	-0.05924300
H	-3.84668000	-5.08311900	1.38618700
H	-6.74701300	-3.51455700	-1.41916500
H	-5.70392400	-5.46281900	-0.24830400

intermediate **G**

C	-1.08249700	-2.51640000	4.44043600
C	-1.07648800	-3.54688100	3.48716000
C	-1.22631800	-3.24784400	2.12641300
C	-1.37277200	-1.91026400	1.70934000
C	-1.39580600	-0.88354700	2.67115000
C	-1.24704000	-1.18455700	4.03186400

P	-1.50665100	-1.46496500	-0.06750900
C	-2.48507100	-2.84880600	-0.77072300
C	-2.04523900	-3.53965300	-1.91616100
C	-2.84250800	-4.54613700	-2.47956700
C	-4.08081800	-4.86862200	-1.90537700
C	-4.52184400	-4.18139000	-0.76407700
C	-3.73306500	-3.17288900	-0.19829100
C	0.16352000	-1.73712300	-0.71866100
C	0.68188500	-1.02513200	-1.86776900
C	2.04065800	-1.43391400	-2.06418900
C	2.37641900	-2.38839800	-1.04295600
C	1.22375800	-2.57948200	-0.21099700
Fe	1.77462200	-0.62445500	-0.20632900
C	3.16116500	-0.14678300	1.19571100
C	1.84095800	-0.00810200	1.74019900
C	1.12862700	0.94896000	0.93964900
C	1.99776900	1.39301800	-0.10801600
C	3.27116700	0.72241700	0.04431100
Pd	-2.26240300	0.71564400	-0.38608900
C	-4.15071600	0.09360100	0.04947600
C	-5.00292000	-0.30825100	-1.00158100
C	-6.29682000	-0.78633300	-0.73799800
C	-6.76369500	-0.87807900	0.58175900
C	-5.92870900	-0.47525500	1.63503600
C	-4.63589300	0.00993300	1.37168300
H	2.71361700	-1.04468800	-2.82964700
H	0.13018500	-0.28640400	-2.45251300
H	1.16240300	-3.21814800	0.67062000
H	3.35291200	-2.85332800	-0.89901000
H	1.73590400	2.09722500	-0.89934800
H	1.43698500	-0.55789300	2.59167000
H	3.93862500	-0.82088700	1.55647800
H	-4.65428300	-0.26301200	-2.03954300
H	-4.00521500	0.31520000	2.21353000
H	-6.93819300	-1.09790000	-1.57080900
H	-6.28220300	-0.53780500	2.67121900
H	-7.77067500	-1.25722800	0.78802100
H	-1.07663300	-3.29202900	-2.36259600
H	-4.08910200	-2.63335800	0.68511800
H	-2.49201000	-5.08148300	-3.36856700
H	-5.49078400	-4.42190500	-0.31439900
H	-4.70232300	-5.65474000	-2.34747000
H	-1.24089500	-4.05309900	1.38413400
H	-1.53549900	0.15212600	2.33968600
H	-0.96024400	-4.58827100	3.80587000
H	-1.26689800	-0.37909600	4.77351000
H	-0.96941600	-2.75375400	5.50361200
N	-1.98915600	2.55483400	-0.74787500
N	-2.24115400	3.73774700	-0.85217500
C	-3.56173800	4.28641300	-0.65333400
C	-3.66929200	5.68089800	-0.78698800
C	-4.68554000	3.49942900	-0.34796900
C	-4.91438600	6.29628400	-0.61061400
H	-2.76881700	6.25453700	-1.02728800
C	-5.92452200	4.12337000	-0.17504100
H	-4.57580700	2.41462200	-0.25012900
C	-6.04248400	5.51845800	-0.30469100
H	-5.00674400	7.38271900	-0.71217100
H	-6.80434600	3.51553500	0.06215500
H	-7.01682400	5.99977500	-0.16777500
P	4.67716600	0.98290000	-1.09731300
C	5.68707300	2.19258100	-0.12339300
C	5.65678500	-0.56469200	-0.79694400
C	6.97490700	2.51493300	-0.60337500
C	5.20914200	2.84252200	1.03073300
C	6.37411600	-0.80160200	0.39386400
C	5.67886800	-1.54315700	-1.80981500
C	7.77325200	3.44816600	0.06809000
H	7.35600900	2.02216500	-1.50589800
C	6.00680400	3.78690100	1.69535100
H	4.21142600	2.60518100	1.41364800
C	7.06548400	-2.00639200	0.57873300
H	6.39312300	-0.03319000	1.17433400
C	6.36836300	-2.75064300	-1.62456000
H	5.15322400	-1.35281900	-2.75215600
C	7.29012000	4.08948500	1.22022500
H	8.77423700	3.68107700	-0.31141100
H	5.62244200	4.28406200	2.59283700
C	7.05842400	-2.98627000	-0.42668900
H	7.61568500	-2.18078700	1.50995200
H	6.37464600	-3.50340900	-2.42038700
H	7.91215700	4.82416300	1.74261600

H	7.60135100	-3.92631000	-0.28098200
H	0.08830300	1.25767000	1.06196600

intermediate **G**^{dtbpf}

P	-1.34468000	-1.98727300	0.16041000
C	0.37500100	-1.86267600	-0.46236800
C	0.66140600	-0.90086700	-1.51170600
C	1.97113600	-1.15243000	-2.02424000
C	2.52329000	-2.26029100	-1.29655700
C	1.55278400	-2.69895900	-0.33955000
Fe	2.01991300	-0.72834500	-0.02535700
C	3.46883500	-0.39201900	1.38817500
C	2.16513000	-0.28464300	1.97113900
C	1.44375000	0.74234300	1.27215800
C	2.30313100	1.25871600	0.25350200
C	3.58048900	0.57493800	0.31549000
Pd	-2.17536000	0.21253500	-0.03517300
C	-4.15761000	-0.22790700	-0.07559500
C	-4.85184600	-0.27126900	-1.30604700
C	-6.23018300	-0.53868400	-1.35189100
C	-6.94943100	-0.76327700	-0.16825500
C	-6.27765800	-0.70687100	1.06177100
C	-4.89867500	-0.43908100	1.10712900
H	2.46951000	-0.56991600	-2.79996500
H	-0.02057400	-0.10920600	-1.83087000
H	1.69419400	-3.52228300	0.35819700
H	3.51617500	-2.69181900	-1.43043600
H	2.04257900	2.03529600	-0.46745400
H	1.79200200	-0.89249600	2.79676000
H	4.23653800	-1.09529700	1.70671900
H	-4.31530900	-0.09431800	-2.24538100
H	-4.40371700	-0.38955700	2.08408400
H	-6.74347800	-0.56997700	-2.32044900
H	-6.82846600	-0.86971400	1.99601500
H	-8.02382100	-0.97398300	-0.20378200
N	-1.73631000	2.04965800	-0.14055500
N	-1.87012900	3.25537100	-0.20401000
C	-3.15231000	3.91178400	-0.26015400
C	-3.11996100	5.31166100	-0.37822900
C	-4.37401900	3.21931300	-0.19564200
C	-4.32230800	6.02637100	-0.43747500
H	-2.14737700	5.81144700	-0.42224000
C	-5.56904600	3.94229700	-0.25497900
H	-4.37198000	2.12924600	-0.10383700
C	-5.54725800	5.34286700	-0.37600400
H	-4.30569600	7.11733400	-0.53099100
H	-6.52360200	3.40731400	-0.20675400
H	-6.48769600	5.90254400	-0.42158800
P	4.99715300	1.29463100	-0.61634100
H	0.40803600	1.04797000	1.43025100
C	-2.02621600	-3.35331500	-0.99929300
C	-3.53679800	-3.57917200	-0.77602000
C	-1.83893800	-2.78470300	-2.42486300
C	-1.26323000	-4.68196800	-0.86372200
H	-3.74859000	-4.13601300	0.14728400
H	-4.09213900	-2.62965900	-0.74989600
H	-3.92407700	-4.17913200	-1.62060100
H	-0.77631300	-2.70454000	-2.70405900
H	-2.33497100	-3.46117000	-3.14491600
H	-2.30247800	-1.78602500	-2.51587700
H	-1.63424900	-5.39333700	-1.62565200
H	-0.18035000	-4.55033300	-1.02488700
H	-1.42161600	-5.14553700	0.12482400
C	-1.32059900	-2.55738200	1.98356800
C	-0.12686800	-3.45702700	2.34658800
C	-1.24987100	-1.25873500	2.81625100
C	-2.64092700	-3.27406200	2.32653800
H	-0.09722500	-4.37715600	1.74068300
H	0.82642000	-2.92034900	2.22223300
H	-0.21258600	-3.75624300	3.40837100
H	-2.12894400	-0.61778900	2.62890200
H	-1.21982200	-1.51378200	3.89249600
H	-0.35159800	-0.67357600	2.57472500
H	-2.70582100	-3.38183400	3.42533900
H	-3.51934000	-2.70026100	1.98807000
H	-2.68709600	-4.28471100	1.88994500
C	6.14256600	-0.16412800	-1.11812600
C	6.17692100	-1.37591900	-0.17166800
C	5.58625300	-0.61303500	-2.48839300
C	7.57348400	0.36194500	-1.34673500
H	6.52720700	-1.10926100	0.83804900
H	5.18065000	-1.83771300	-0.08618800

H	6.86916100	-2.14013800	-0.57631200
H	5.62679200	0.20972800	-3.22252400
H	6.18210700	-1.46071400	-2.87826900
H	4.53992700	-0.94064300	-2.40347800
H	8.16738500	-0.41932500	-1.85810700
H	7.57839300	1.26069400	-1.98837900
H	8.08698700	0.60349600	-0.40170400
C	5.81586400	2.26386300	0.84092900
C	4.68650400	3.10768300	1.47433100
C	6.46929400	1.40620000	1.93603500
C	6.84385200	3.24317100	0.23197100
H	4.12140100	3.66898000	0.70851600
H	3.97455500	2.48558100	2.04023800
H	5.13366800	3.84060100	2.17237800
H	7.33982600	0.84232200	1.56143300
H	6.82713000	2.05921000	2.75600700
H	5.74830600	0.69318500	2.37027500
H	7.21220100	3.92578100	1.02155700
H	7.71739700	2.72857300	-0.19661700
H	6.38156600	3.85492800	-0.56234100

intermediate **G^{dppb}**

C	-1.23083900	-3.72774200	4.23047000
C	-0.89292300	-4.44365600	3.07156000
C	-0.64743300	-3.76532500	1.87026200
C	-0.74192900	-2.36020700	1.81985600
C	-1.08577500	-1.64821600	2.98597600
C	-1.32557600	-2.32901200	4.18720300
P	-0.36983700	-1.39117800	0.30589000
C	-0.86129300	-2.47766900	-1.08504700
C	-0.10206800	-2.51153100	-2.27288500
C	-0.54663800	-3.25319200	-3.37583000
C	-1.75482300	-3.96253200	-3.30858800
C	-2.52045200	-3.92217300	-2.13421300
C	-2.08194900	-3.18198400	-1.02971700
Pd	-1.36754700	0.71687700	0.32003800
C	-3.13135900	-0.20916500	-0.09278600
C	-3.56109100	-0.32590900	-1.43214800
C	-4.75508300	-0.99274500	-1.75038900
C	-5.54103800	-1.56193200	-0.73708800
C	-5.12650300	-1.44944600	0.59865000
C	-3.93430900	-0.77686600	0.91881600
H	-2.95514800	0.09551400	-2.24222800
H	-3.63071800	-0.70727100	1.96900000
H	-5.06579900	-1.07509600	-2.79860500
H	-5.73267000	-1.88713700	1.40088200
H	-6.46999000	-2.08691100	-0.98578200
H	0.83417000	-1.94946500	-2.34609900
H	-2.69970500	-3.13496100	-0.12755400
H	0.05449600	-3.27460100	-4.29124200
H	-3.47420800	-4.45679600	-2.07812200
H	-2.10147900	-4.53999300	-4.17220600
H	-0.39420700	-4.32805200	0.96610500
H	-1.17203300	-0.55532300	2.93475600
H	-0.82155800	-5.53616200	3.10282400
H	-1.59548800	-1.76710900	5.08755600
H	-1.42558400	-4.26195300	5.16655600
N	-1.38718300	2.60111500	0.52771100
N	-1.82912000	3.73201900	0.57620200
C	-3.22314100	4.04607200	0.36806000
C	-3.55979000	5.40787900	0.44818500
C	-4.20219300	3.07344000	0.10217200
C	-4.88988800	5.80189200	0.25926000
H	-2.76604400	6.13152200	0.65814400
C	-5.52754000	3.47689600	-0.08534600
H	-3.91440700	2.01893700	0.04405800
C	-5.87450400	4.83722000	-0.00761100
H	-5.16040900	6.86132200	0.31994400
H	-6.29468500	2.72332800	-0.29384400
H	-6.91513200	5.14546000	-0.15547800
C	1.47856400	-1.35217800	0.21325900
C	2.24766800	-2.68134500	0.27471500
H	1.79150900	-0.65738600	1.01090700
H	1.68667400	-0.82374700	-0.73267700
C	3.64496900	-2.56587400	-0.36558700
H	2.32280200	-3.02037400	1.32416200
H	1.68743300	-3.46397900	-0.26888200
C	4.57106000	-1.50664300	0.28717300
H	4.13937100	-3.55294800	-0.34022900
H	3.50037900	-2.33268900	-1.43889300
H	4.03273500	-0.91098400	1.04718500
H	5.41749500	-1.99692900	0.79981400

P	5.30099700	-0.33851700	-0.99725400
C	3.74405500	0.56653500	-1.44839500
C	3.12642800	1.50288600	-0.59229800
C	3.09790100	0.21502600	-2.65166900
C	1.87116900	2.03746700	-0.90820800
H	3.62501100	1.79542500	0.33853800
C	1.83664300	0.74754200	-2.96722500
H	3.58370300	-0.49044200	-3.33604800
C	1.21651700	1.64892200	-2.08998200
H	1.38217500	2.73910700	-0.22574200
H	1.33701400	0.45520900	-3.89712600
H	0.22699700	2.05546100	-2.32210700
C	6.12057000	0.93258700	0.06305400
C	6.53459300	2.13109000	-0.56094600
C	6.40768100	0.75445600	1.43118600
C	7.19625500	3.12708100	0.16513900
H	6.32371400	2.28491800	-1.62588300
C	7.08257300	1.74960100	2.15600800
H	6.10328100	-0.16177400	1.94601100
C	7.47522900	2.93877800	1.52864700
H	7.49946100	4.05341900	-0.33479500
H	7.29529200	1.59344900	3.21931700
H	7.99699200	3.71599900	2.09694700

intermediate $\mathbf{G^{2c}}$

C	-0.79223900	-2.77667100	4.41506200
C	-0.72181500	-3.79253000	3.44874900
C	-0.88534400	-3.48540500	2.09141600
C	-1.11056500	-2.15364500	1.69093400
C	-1.19759700	-1.14273100	2.66564500
C	-1.03488500	-1.45187500	4.02293200
P	-1.26521400	-1.69568600	-0.08067500
C	-2.16348900	-3.12213300	-0.80461800
C	-1.68826700	-3.76550800	-1.96341300
C	-2.42917000	-4.80452800	-2.54438300
C	-3.64608600	-5.20651300	-1.97456800
C	-4.12212100	-4.56683200	-0.81975800
C	-3.38961300	-3.52647900	-0.23627200
C	0.42010600	-1.86148200	-0.72787300
C	0.89733800	-1.11237300	-1.87150500
C	2.28291400	-1.42524800	-2.05698000
C	2.67566700	-2.35584000	-1.03409100
C	1.53229100	-2.62847800	-0.21196000
Fe	1.94568600	-0.63966000	-0.20084800
C	3.30014200	-0.06240700	1.19535900
C	1.97651800	-0.02971200	1.74850400
C	1.18764700	0.87389300	0.95790200
C	2.01223000	1.38838200	-0.09368400
C	3.33473700	0.81823400	0.04798500
Pd	-2.13933400	0.44282100	-0.38167100
C	-3.99126700	-0.28518300	0.04109100
C	-4.81407200	-0.72728200	-1.01725600
C	-6.08162700	-1.27623500	-0.76388600
C	-6.55036600	-1.40026500	0.55257800
C	-5.74431200	-0.95920400	1.61303200
C	-4.47824200	-0.40308300	1.36007800
H	2.93366900	-0.98758400	-2.81559100
H	0.30093400	-0.41313200	-2.46089300
H	1.50844700	-3.27109600	0.66858100
H	3.68085800	-2.75180800	-0.88179500
H	1.69006000	2.07419700	-0.87887400
H	1.62178600	-0.61309600	2.59965000
H	4.13098400	-0.67500200	1.54692400
H	-4.46196000	-0.65792900	-2.05270600
H	-3.86979800	-0.06865700	2.20716300
H	-6.70037100	-1.61754900	-1.60207900
H	-6.09970500	-1.04686800	2.64670200
H	-7.53658800	-1.83418200	0.75081400
H	-0.73635100	-3.45508800	-2.40657500
H	-3.77231800	-3.02435300	0.65795300
H	-2.05150900	-5.30276800	-3.44374700
H	-5.07480200	-4.87005500	-0.37327300
H	-4.22369600	-6.01778200	-2.43049500
H	-0.84891000	-4.28033700	1.33884000
H	-1.39792600	-0.11314100	2.34699900
H	-0.54426700	-4.82911000	3.75457100
H	-1.10487700	-0.65871100	4.77471900
H	-0.66812000	-3.02055800	5.47551200
N	-1.97056000	2.29914300	-0.72645500
N	-2.29586900	3.46670400	-0.81209400
C	-3.64348300	3.92844500	-0.60279300
C	-3.83959200	5.31640700	-0.71078000

C	-4.71958900	3.07070900	-0.30970300
C	-5.11456000	5.85866200	-0.52271400
H	-2.97886600	5.95113500	-0.94198300
C	-5.99560100	3.60434800	-0.12218600
H	-4.54555200	1.99246200	-0.23073200
C	-6.17360200	4.99073000	-0.23075300
H	-5.30294800	6.93316700	-0.59809400
H	-6.85265700	2.96391500	0.10590300
P	4.71022800	1.18276600	-1.10280500
C	5.59129600	2.51716100	-0.16755000
C	5.83346000	-0.25205100	-0.74997600
C	6.82211700	2.97934700	-0.68136500
C	5.06798800	3.12177300	0.99153700
C	6.57851800	-0.36908800	0.44150200
C	5.93872500	-1.26761000	-1.72035500
C	7.52276800	4.00627800	-0.03805200
H	7.23616500	2.52316400	-1.58861500
C	5.76628900	4.15969500	1.62777100
H	4.11299400	2.77611100	1.40008400
C	7.38254800	-1.49415200	0.66862700
H	6.52869200	0.42944600	1.18968500
C	6.74042300	-2.39581700	-1.49215400
H	5.39005800	-1.16891400	-2.66365000
C	6.99509400	4.60184800	1.11907400
H	8.48116900	4.34838800	-0.44381100
H	5.34762400	4.62062500	2.52922700
C	7.46008400	-2.51288900	-0.29418200
H	7.95401700	-1.57557400	1.59979300
H	6.81096700	-3.17877300	-2.25509400
H	7.53985800	5.40981000	1.61906700
H	8.09077200	-3.39038100	-0.11556200
H	0.12830400	1.10391700	1.09016000
F	-7.40591100	5.50604300	-0.04884600

intermediate **G**[2d]

C	-0.33179700	-2.95915900	4.41115100
C	-0.15910300	-3.95424800	3.43609600
C	-0.36062100	-3.65535000	2.08198300
C	-0.72734600	-2.35186800	1.69368600
C	-0.91694800	-1.36366300	2.67718300
C	-0.71562100	-1.66448600	4.03119900
P	-0.93891500	-1.89353500	-0.07242800
C	-1.67562800	-3.40422900	-0.81003800
C	-1.12972700	-3.98359900	-1.97156000
C	-1.75007500	-5.09349000	-2.56248900
C	-2.91692700	-5.63114300	-1.99997600
C	-3.46382100	-5.05538500	-0.84292300
C	-2.85204000	-3.94476700	-0.24970100
C	0.75275900	-1.87884500	-0.72579900
C	1.14370100	-1.07643900	-1.86567700
C	2.54999100	-1.25632500	-2.07109700
C	3.04072400	-2.15750000	-1.06415200
C	1.93792400	-2.54452700	-0.23254200
Fe	2.16417100	-0.52610900	-0.20178400
C	3.46497400	0.15641000	1.19755400
C	2.14483000	0.06766500	1.75296700
C	1.27660000	0.90379500	0.97122700
C	2.05045200	1.49852700	-0.07657600
C	3.41884300	1.04646500	0.05768200
Pd	-2.04302500	0.13822800	-0.34143300
C	-3.80660100	-0.79566000	0.06386500
C	-4.57735800	-1.30774200	-1.00238900
C	-5.77427900	-2.00120700	-0.76037000
C	-6.22391700	-2.20279500	0.55317500
C	-5.47101900	-1.69329000	1.62199900
C	-4.27628200	-0.99313100	1.37991200
H	3.14766200	-0.75144900	-2.83167700
H	0.47654500	-0.42919300	-2.43832900
H	1.98514500	-3.19597400	0.64050400
H	4.08025500	-2.45986600	-0.92855400
H	1.66760200	2.15918200	-0.85610000
H	1.84509300	-0.55211700	2.59950800
H	4.34683000	-0.38405500	1.54302100
H	-4.23822500	-1.17859500	-2.03649700
H	-3.70821000	-0.60819600	2.23368100
H	-6.35215000	-2.39395200	-1.60531300
H	-5.81272000	-1.83973900	2.65372500
H	-7.15459100	-2.74899500	0.74261500
H	-0.21666200	-3.56750900	-2.40964000
H	-3.29142600	-3.49363000	0.64565300
H	-1.31718000	-5.54046000	-3.46396300
H	-4.37917100	-5.46391600	-0.40226400

H	-3.40051400	-6.49779300	-2.46345400
H	-0.24399500	-4.43579800	1.32244700
H	-1.22872000	-0.35947200	2.36632900
H	0.12845700	-4.96865300	3.73264000
H	-0.86606900	-0.88937100	4.79003300
H	-0.17739900	-3.19699500	5.46904100
N	-2.08149600	2.00597300	-0.66172500
N	-2.53855100	3.12794400	-0.75449500
C	-3.92496100	3.44106700	-0.56594500
C	-4.27911200	4.79202300	-0.70092500
C	-4.90926600	2.47684300	-0.26363500
C	-5.60840000	5.19738600	-0.53669600
H	-3.49405000	5.51716600	-0.93685500
C	-6.23114500	2.87172400	-0.09997500
H	-4.62153400	1.42533900	-0.16224700
C	-6.59274200	4.23318600	-0.23446200
H	-5.86752400	6.25329200	-0.64502800
H	-7.01396900	2.14375100	0.13326800
P	4.75002300	1.53907600	-1.09661900
C	5.53591000	2.92167000	-0.14672500
C	5.98933700	0.19339900	-0.78348700
C	6.73091900	3.47418000	-0.65599900
C	4.97111500	3.47827400	1.01688800
C	6.74626400	0.10739000	0.40319300
C	6.17560300	-0.78336500	-1.78098900
C	7.35696300	4.54312700	-0.00447300
H	7.17646000	3.05548500	-1.56641500
C	5.59417500	4.55810200	1.66164900
H	4.04315300	3.06220100	1.42214100
C	7.64198700	-0.95236000	0.59902000
H	6.63337500	0.87933100	1.17214300
C	7.06969800	-1.84625200	-1.58452500
H	5.61629000	-0.70591300	-2.71997300
C	6.78839100	5.09062400	1.15703800
H	8.28877500	4.95545800	-0.40693200
H	5.14402500	4.98078400	2.56670100
C	7.80076400	-1.93494200	-0.39107400
H	8.22154100	-1.01073100	1.52693300
H	7.20247200	-2.59997600	-2.36832600
H	7.27444100	5.93116800	1.66384300
H	8.50297200	-2.76128200	-0.23690900
H	0.20068600	1.03493400	1.10515800
O	-7.91156200	4.51478900	-0.05530700
C	-8.34134000	5.86488100	-0.18010500
H	-9.42601100	5.85536300	0.00098200
H	-7.84897700	6.51588700	0.56749700
H	-8.14086000	6.26000100	-1.19438000

intermediate $\mathbf{G^{2e}}$

C	0.23200300	-3.08888700	4.42797000
C	0.50898600	-4.06893500	3.46171500
C	0.28898600	-3.80075600	2.10419600
C	-0.20068400	-2.54193000	1.70370300
C	-0.49371400	-1.57039500	2.67821100
C	-0.27435200	-1.84091300	4.03578700
P	-0.44074000	-2.12720500	-0.06832900
C	-1.05359700	-3.69950700	-0.78647900
C	-0.46677800	-4.24608700	-1.94377100
C	-0.99794400	-5.40992600	-2.51786300
C	-2.11505000	-6.03307600	-1.94265900
C	-2.70197400	-5.48995200	-0.78941800
C	-2.17945300	-4.32651800	-0.21284200
C	1.24096400	-1.96222200	-0.72223600
C	1.55812200	-1.12798800	-1.86262700
C	2.97712200	-1.16406100	-2.05418900
C	3.54801100	-2.00610100	-1.03864800
C	2.48336400	-2.50106200	-0.21452100
Fe	2.50109100	-0.47030000	-0.19154900
C	3.72755600	0.35196500	1.20015400
C	2.42821500	0.12008700	1.76299000
C	1.47077400	0.85706600	0.98596500
C	2.16899600	1.53112800	-0.06680500
C	3.57876000	1.23068200	0.06043800
Pd	-1.70173800	-0.19120200	-0.37851400
C	-3.38300700	-1.25970000	0.02626100
C	-4.09291600	-1.85269500	-1.03944200
C	-5.24023100	-2.62557400	-0.79735200
C	-5.69613300	-2.82631200	0.51407900
C	-5.00075600	-2.23783400	1.58122400
C	-3.85613400	-1.45809900	1.34018800
H	3.52672700	-0.60442700	-2.81249000
H	0.83427400	-0.55537300	-2.44591000

H	2.58900800	-3.14168700	0.66143600
H	4.61182700	-2.19952500	-0.89275900
H	1.71173900	2.14615300	-0.84354000
H	2.20210700	-0.52671600	2.61215300
H	4.66529900	-0.08827600	1.54070200
H	-3.74536400	-1.72481700	-2.07079300
H	-3.33227000	-1.01109300	2.19177600
H	-5.77342800	-3.08016900	-1.64040200
H	-5.34853300	-2.38359500	2.61081200
H	-6.58797500	-3.43363800	0.70327200
H	0.40798400	-3.76253200	-2.39087300
H	-2.64957100	-3.90147900	0.67967600
H	-0.53432500	-5.83175100	-3.41606700
H	-3.57882300	-5.96654700	-0.33907300
H	-2.52869000	-6.94158300	-2.39333200
H	0.48647700	-4.57151200	1.35149800
H	-0.89920300	-0.60304700	2.35977000
H	0.89248100	-5.04807400	3.76781300
H	-0.50588700	-1.07919200	4.78763700
H	0.40112300	-3.30304000	5.48858900
N	-1.88473200	1.66164700	-0.72843000
N	-2.42116900	2.74850400	-0.82435600
C	-3.84057300	2.93771100	-0.63000900
C	-4.29963800	4.26027300	-0.73989900
C	-4.73061400	1.88564500	-0.35494200
C	-5.65876800	4.53826600	-0.56670400
H	-3.57629900	5.05019800	-0.96265700
C	-6.08689900	2.16594600	-0.18508600
H	-4.35209600	0.86136400	-0.27865000
C	-6.55233300	3.49068400	-0.28730300
H	-6.03209400	5.56226900	-0.65417400
H	-6.79018500	1.35347200	0.02090900
P	4.84511000	1.86948900	-1.09629800
C	5.46475500	3.33492700	-0.14782300
C	6.22783000	0.67304900	-0.77756200
C	6.57429400	4.03415800	-0.66989600
C	4.85380800	3.80929700	1.02884400
C	6.99260300	0.68060000	0.40715400
C	6.51869200	-0.28464800	-1.76861200
C	7.07397900	5.16743000	-0.01780600
H	7.05321900	3.68047100	-1.59086100
C	5.34906200	4.95301300	1.67414600
H	3.99042400	3.27947700	1.44389900
C	8.00055100	-0.27213200	0.60765200
H	6.79705300	1.44125100	1.17078100
C	7.52526100	-1.24066700	-1.56730800
H	5.95313700	-0.27605600	-2.70703700
C	6.46059000	5.63212900	1.15686200
H	7.94118700	5.69466100	-0.43030200
H	4.86435500	5.31077800	2.58924800
C	8.26449600	-1.23884600	-0.37557400
H	8.58564100	-0.25862200	1.53380100
H	7.73904100	-1.98067600	-2.34623900
H	6.84716200	6.52264800	1.66388900
H	9.05432000	-1.98107500	-0.21771500
H	0.38846200	0.87405300	1.12934700
C	-8.01225400	3.78722600	-0.04795800
F	-8.28289900	3.92628300	1.27649000
F	-8.81095000	2.79300200	-0.50696100
F	-8.40446400	4.93590200	-0.65136600

intermediate **G^{2f}**

C	-0.94253400	-2.76452300	4.40310600
C	-0.90845000	-3.77354800	3.42771500
C	-1.07015500	-3.44972300	2.07404300
C	-1.25684100	-2.10828700	1.68633000
C	-1.30755900	-1.10371100	2.67016600
C	-1.14686400	-1.42956200	4.02377600
P	-1.40820700	-1.62983400	-0.08013300
C	-2.35355200	-3.02211700	-0.81071700
C	-1.90615400	-3.66766900	-1.97935600
C	-2.68091600	-4.67977400	-2.56367600
C	-3.90404600	-5.05267700	-1.98740700
C	-4.35244600	-4.41066200	-0.82289400
C	-3.58608800	-3.39694500	-0.23603800
C	0.26608200	-1.84062300	-0.74215100
C	0.76061400	-1.09019800	-1.87742200
C	2.13157200	-1.45166700	-2.08171100
C	2.49838400	-2.41426300	-1.07900500
C	1.35316500	-2.65803900	-0.25061700
Fe	1.84121600	-0.68676500	-0.20862200
C	3.21642100	-0.19230000	1.19873000

C	1.89434400	-0.10772700	1.74960800
C	1.14890000	0.84181600	0.97060800
C	1.99947000	1.33491600	-0.07038800
C	3.29431400	0.70306900	0.06495600
Pd	-2.22427400	0.53682600	-0.35096100
C	-4.09376400	-0.14437800	0.07621400
C	-4.93621900	-0.55184300	-0.98052500
C	-6.21585900	-1.07017800	-0.72336500
C	-6.67750800	-1.19734300	0.59527600
C	-5.85202800	-0.78981500	1.65423300
C	-4.57374800	-0.26429700	1.39755000
H	2.79111700	-1.02657100	-2.83971100
H	0.18556100	-0.35884300	-2.44877900
H	1.31306000	-3.31493100	0.61874000
H	3.48933400	-2.85027500	-0.94393700
H	1.71211400	2.04672100	-0.84592200
H	1.51149000	-0.68727500	2.59103500
H	4.01676100	-0.84784100	1.54329800
H	-4.59063300	-0.47957100	-2.01797200
H	-3.95017500	0.04397900	2.24357600
H	-6.84972700	-1.38542100	-1.56048900
H	-6.20163300	-0.88015900	2.68966000
H	-7.67312900	-1.60790300	0.79641100
H	-0.94934100	-3.38025900	-2.42743300
H	-3.94734400	-2.89267300	0.66586700
H	-2.32478300	-5.17993700	-3.47072200
H	-5.30970300	-4.69099700	-0.37126200
H	-4.50798400	-5.84305400	-2.44592000
H	-1.06258700	-4.23885500	1.31454400
H	-1.47758300	-0.06555400	2.36161900
H	-0.76081500	-4.81768800	3.72356500
H	-1.18823400	-0.64119400	4.78268400
H	-0.81995400	-3.02133900	5.46067900
N	-2.00235300	2.38944600	-0.67328800
N	-2.28791700	3.56849000	-0.74569600
C	-3.62017400	4.05910900	-0.52841600
C	-3.80247300	5.45415800	-0.60756300
C	-4.72764700	3.23747200	-0.25307900
C	-5.06153300	6.02937600	-0.41531100
C	-5.99003200	3.80354300	-0.06056900
H	-4.57309700	2.15473100	-0.19396800
C	-6.15619000	5.19772100	-0.14133300
H	-5.16321600	7.11619700	-0.48347600
H	-6.84612900	3.15558700	0.15233200
H	-7.14474700	5.64399100	0.00899400
P	4.68803900	1.02896100	-1.07562600
C	5.65893800	2.25623900	-0.08432000
C	5.71875100	-0.48995100	-0.80068500
C	6.93245600	2.63221100	-0.56340700
C	5.16350100	2.86819500	1.08307600
C	6.44673800	-0.72104000	0.38478800
C	5.77027000	-1.45184800	-1.82827000
C	7.70041900	3.58087000	0.12176200
H	7.32651600	2.16957000	-1.47625000
C	5.93033500	3.82808700	1.76154900
H	4.17625000	2.58946600	1.46516600
C	7.17745400	-1.90530400	0.55003100
H	6.44267000	0.03587200	1.17664300
C	6.49922200	-2.63884800	-1.66270000
H	5.23647800	-1.26461500	-2.76664500
C	7.20008800	4.18407600	1.28707000
H	8.69080200	3.85583400	-0.25740300
H	5.53250300	4.29560500	2.66901500
C	7.19963500	-2.86979200	-0.46994400
H	7.73558400	-2.07552200	1.47729500
H	6.52808000	-3.37892700	-2.46985500
H	7.79810500	4.93098000	1.82005100
H	7.77328400	-3.79369600	-0.33954100
H	0.10007400	1.11553600	1.10307500
F	-2.75214900	6.24724800	-0.86951100

intermediate **G**^{2g}

C	-0.80071000	-3.08749800	4.33623900
C	-0.73947400	-4.06679600	3.33230800
C	-0.90971100	-3.70863500	1.98827400
C	-1.13297600	-2.36235800	1.63898400
C	-1.21100300	-1.38830600	2.65126200
C	-1.04113800	-1.74823000	3.99510500
P	-1.29690500	-1.83445100	-0.11297100
C	-2.18710400	-3.23935200	-0.88885700
C	-1.70454100	-3.84322700	-2.06582300
C	-2.43961400	-4.86419100	-2.68489700

C	-3.65852500	-5.28774100	-2.13534000
C	-4.14211900	-4.68749300	-0.96261800
C	-3.41541700	-3.66520700	-0.34095800
C	0.38922100	-1.97525500	-0.76715200
C	0.86212300	-1.19196000	-1.88912300
C	2.24691300	-1.49917100	-2.08998100
C	2.64385700	-2.46044700	-1.09737900
C	1.50332500	-2.75783900	-0.27975000
Fe	1.91817300	-0.77057000	-0.20933500
C	3.27251100	-0.23713200	1.20412100
C	1.94873400	-0.21868500	1.75747400
C	1.16094400	0.70849400	0.99355700
C	1.98675600	1.25315900	-0.04168400
C	3.30868100	0.67722800	0.08331200
Pd	-2.19398600	0.30608700	-0.32214500
C	-4.04126200	-0.44404600	0.10236200
C	-4.88224500	-0.84114500	-0.96011900
C	-6.13915500	-1.41604300	-0.71128800
C	-6.58138800	-1.61107500	0.60578700
C	-5.75952900	-1.21348000	1.67136400
C	-4.50419700	-0.63193600	1.42230300
H	2.89438900	-1.03924400	-2.83813300
H	0.26340700	-0.47440700	-2.45359500
H	1.48230500	-3.42685500	0.58101900
H	3.64962500	-2.86083400	-0.96127200
H	1.66576300	1.96244300	-0.80624500
H	1.59295700	-0.82690500	2.59059700
H	4.10197200	-0.86137100	1.53793400
H	-4.55186500	-0.71586800	-1.99759800
H	-3.88333000	-0.33402400	2.27414300
H	-6.77062700	-1.72176400	-1.55389300
H	-6.09419900	-1.35583900	2.70600100
H	-7.55910500	-2.06557400	0.80060700
H	-0.75130200	-3.51598500	-2.49371300
H	-3.80415800	-3.19400400	0.56737800
H	-2.05586700	-5.33138400	-3.59830800
H	-5.09653300	-5.00712100	-0.53145100
H	-4.23179300	-6.08479200	-2.62097100
H	-0.88000600	-4.47475600	1.20608100
H	-1.41093800	-0.34728800	2.37155200
H	-0.56373200	-5.11467300	3.59823800
H	-1.10426800	-0.98339700	4.77634400
H	-0.67131500	-3.37119700	5.38615000
N	-2.03155200	2.17230400	-0.59880100
N	-2.35043400	3.34241500	-0.63581200
C	-3.68265900	3.80989400	-0.39117600
C	-3.87997400	5.21711300	-0.45067700
C	-4.75561200	2.95117500	-0.10963300
C	-5.17261900	5.72903200	-0.22056300
C	-6.03375900	3.46861500	0.11648600
H	-4.56317700	1.87343200	-0.07274600
C	-6.23444200	4.85680300	0.05978300
H	-5.35519300	6.80560700	-0.25911300
H	-6.86580000	2.79190900	0.33444600
H	-7.23156900	5.27521100	0.23495500
P	4.68450700	1.07492600	-1.05535400
C	5.58304100	2.36038200	-0.06907400
C	5.79630900	-0.38309900	-0.76680700
C	6.82502600	2.81913600	-0.55862800
C	5.06125400	2.93458600	1.10604100
C	6.53831200	-0.56031400	0.41918100
C	5.89586600	-1.35406100	-1.78233300
C	7.53769200	3.81218700	0.12344800
H	7.23845000	2.38644900	-1.47760900
C	5.77187400	3.93883800	1.78153600
H	4.09775900	2.59114300	1.49613900
C	7.33243900	-1.70123100	0.59635700
H	6.49389800	0.20400900	1.20259700
C	6.68802000	-2.49797300	-1.60453800
H	5.34954200	-1.20813600	-2.72082000
C	7.01147400	4.37738400	1.29640300
H	8.50468400	4.15147200	-0.26397700
H	5.35425500	4.37597500	2.69527600
C	7.40379200	-2.67569100	-0.41175200
H	7.90105400	-1.82981400	1.52397000
H	6.75370500	-3.24590000	-2.40226800
H	7.56591000	5.15875100	1.82715100
H	8.02663400	-3.56576800	-0.27199200
H	0.10104800	0.93384100	1.13000100
O	-2.79381500	5.97772600	-0.72738800
C	-2.95354400	7.38856600	-0.80089500
H	-3.30151700	7.80685400	0.16307100

H	-1.95774900	7.79110500	-1.03642100
H	-3.66472600	7.67369500	-1.59970700

intermediate $\mathbf{G}^{2h}$

C	-1.33382800	-1.01536900	4.53799500
C	-1.19355900	-2.33229800	4.07115200
C	-1.15896800	-2.59270100	2.69439000
C	-1.25384000	-1.53027000	1.77457800
C	-1.40970500	-0.21463900	2.24878600
C	-1.44667000	0.04391400	3.62522200
P	-1.16172300	-1.80668100	-0.03844900
C	-2.04929600	-3.39281300	-0.27614100
C	-1.45846000	-4.45979000	-0.97974400
C	-2.18631500	-5.63525900	-1.21361100
C	-3.50487700	-5.75187600	-0.74997900
C	-4.09613800	-4.68940600	-0.04882300
C	-3.37681600	-3.51194300	0.18716000
C	0.57797600	-2.19881200	-0.34637700
C	1.22328400	-1.93950500	-1.61674200
C	2.60886700	-2.27664400	-1.48415700
C	2.83469400	-2.73338500	-0.13999700
C	1.58723600	-2.68642700	0.56650400
Fe	2.06818100	-0.84054400	-0.13374300
C	3.27021700	0.22823500	1.10166500
C	1.89397500	0.47149200	1.42511100
C	1.25167900	1.00761500	0.25778500
C	2.22014100	1.07850400	-0.79443100
C	3.48571300	0.60307200	-0.27888400
Pd	-1.90615700	0.11362600	-1.14849700
C	-3.84017300	-0.32477300	-0.74458400
C	-4.60061900	-1.05022500	-1.68319500
C	-5.93041400	-1.40625700	-1.40228900
C	-6.51818700	-1.04564200	-0.18069200
C	-5.76566300	-0.32351800	0.75829200
C	-4.43698100	0.03563000	0.47981000
H	3.36641000	-2.15590100	-2.25997200
H	0.73181900	-1.53766900	-2.50500800
H	1.42764600	-2.93884000	1.61520800
H	3.79800500	-3.02325600	0.28235100
H	2.03259300	1.41987000	-1.81376300
H	1.41140500	0.26192800	2.38091800
H	4.02076100	-0.20510500	1.76348100
H	-4.15829900	-1.35080400	-2.63937800
H	-3.87506900	0.60682400	1.22460600
H	-6.50608600	-1.97378900	-2.14303000
H	-6.21369000	-0.03118200	1.71519300
H	-7.55482800	-1.32447800	0.03806700
H	-0.42794800	-4.36996400	-1.33914200
H	-3.84598700	-2.68388700	0.72784100
H	-1.71924700	-6.46321300	-1.75777200
H	-5.12685700	-4.77136800	0.31159400
H	-4.07185500	-6.67056700	-0.93487100
H	-1.06975100	-3.62200300	2.33038300
H	-1.51172700	0.60468100	1.52847000
H	-1.11749600	-3.16094300	4.78333700
H	-1.57306700	1.07318600	3.97691900
H	-1.36593400	-0.81773600	5.61479000
N	-1.83448500	1.93294200	-1.72883000
N	-2.38833000	2.99522500	-1.48212600
C	-3.76264800	3.01250800	-1.06684200
C	-4.11944200	3.61481600	0.15679000
C	-4.74430700	2.43532400	-1.88641000
C	-5.46615700	3.62166200	0.54953200
C	-6.08608700	2.46562600	-1.49261000
H	-4.43652000	1.95617500	-2.81923700
C	-6.44837100	3.05787000	-0.27546800
H	-5.73513500	4.06098800	1.51353600
H	-6.84633800	2.00192100	-2.12845800
H	-7.49559200	3.06994100	0.04138500
P	5.00508300	0.50558300	-1.29491900
C	5.83520100	2.09078300	-0.81619500
C	6.00661100	-0.69508800	-0.29503600
C	7.14807800	2.31392300	-1.28442200
C	5.20170000	3.09646500	-0.06114100
C	6.58476000	-0.36394200	0.94783200
C	6.19233700	-1.99410800	-0.80682800
C	7.81858600	3.50556700	-0.98522200
H	7.64944300	1.54224200	-1.88090500
C	5.87163600	4.29560300	0.22694000
H	4.18254500	2.93983100	0.30643600
C	7.30074900	-1.32347700	1.67606400
H	6.47490000	0.65296700	1.33998000

C	6.90638200	-2.95613700	-0.07711300
H	5.77638600	-2.25001100	-1.78770300
C	7.18057900	4.50235800	-0.22928600
H	8.84056400	3.66082900	-1.34808200
H	5.36664800	5.06930500	0.81565700
C	7.45731200	-2.62319400	1.16876500
H	7.74192700	-1.05561000	2.64242700
H	7.04098500	-3.96314600	-0.48682100
H	7.70244800	5.43768600	-0.00049000
H	8.01927800	-3.37093900	1.73861300
H	0.20217000	1.29563100	0.16789000
C	-3.05172900	4.14734800	1.08104700
F	-3.57927900	4.65086800	2.22400600
F	-2.18401800	3.15686100	1.46043500
F	-2.30620000	5.12206800	0.52175500

transition state **TS5**

C	-1.19738700	-1.84791700	4.36281000
C	-1.20803900	-2.82041100	3.35034100
C	-1.48241900	-2.45554900	2.02535100
C	-1.73888500	-1.10925200	1.70094900
C	-1.74734400	-0.14391100	2.72444800
C	-1.47369000	-0.50880900	4.04915300
P	-1.99026700	-0.54936600	-0.03285300
C	-2.94566700	-1.91950500	-0.80112300
C	-2.68701000	-2.27738000	-2.13903300
C	-3.45510300	-3.26897900	-2.76396300
C	-4.48224100	-3.91350800	-2.05838500
C	-4.74254500	-3.55892500	-0.72661700
C	-3.98497000	-2.56206700	-0.09934100
C	-0.36164500	-0.78444900	-0.80615100
C	0.24077400	0.15206600	-1.72340700
C	1.48942800	-0.39466900	-2.15997900
C	1.66894700	-1.67239300	-1.52690800
C	0.53186800	-1.91751600	-0.69048300
Fe	1.45304600	-0.20407100	-0.12114200
C	2.83926200	-0.54467300	1.31977000
C	1.57561600	-0.22522300	1.91665200
C	1.17866700	1.07555200	1.45073100
C	2.19849200	1.56109900	0.57121800
C	3.23879500	0.56229400	0.47813300
Pd	-3.06473600	1.51111200	-0.01145500
C	-4.73441200	0.50855700	0.57646500
C	-5.63756900	0.09211400	-0.42307700
C	-6.81972500	-0.58700200	-0.08850800
C	-7.11457600	-0.87241000	1.25287500
C	-6.22830900	-0.45433100	2.25636000
C	-5.05196300	0.23884300	1.92085600
H	2.19626500	0.09540900	-2.82998900
H	-0.18393700	1.11143000	-2.01047100
H	0.38051100	-2.79280600	-0.05908100
H	2.53782400	-2.32362500	-1.63038100
H	2.19473700	2.50748500	0.03203200
H	0.99781400	-0.87347500	2.57670800
H	3.38719200	-1.47770000	1.45107500
H	-5.41118000	0.27405000	-1.47995800
H	-4.38063000	0.55799600	2.72456200
H	-7.50476600	-0.90633200	-0.88254100
H	-6.45145500	-0.66439600	3.30919500
H	-8.03128000	-1.41223000	1.51501500
H	-1.87967800	-1.78266400	-2.68923300
H	-4.21117400	-2.27362400	0.93172300
H	-3.24627100	-3.54189800	-3.80396100
H	-5.55009900	-4.04815100	-0.17236700
H	-5.08028200	-4.69005300	-2.54724100
H	-1.51145700	-3.22069200	1.24304500
H	-1.97786500	0.89720300	2.46538400
H	-1.00737200	-3.86894700	3.59483800
H	-1.48070200	0.25172600	4.83675400
H	-0.98436500	-2.13660000	5.39746900
N	-3.85328500	3.36047400	0.41663700
N	-2.94046600	4.03557700	0.06731800
C	-1.78293200	3.27462600	-0.98026200
C	-2.26883400	3.01992400	-2.27928900
C	-0.43390600	3.62629400	-0.78576000
C	-1.38697900	3.04253600	-3.37252100
H	-3.33856200	2.82185800	-2.42912800
C	0.43378400	3.67136900	-1.88224000
H	-0.07617500	3.87478800	0.21947300
C	-0.03898700	3.37765900	-3.17581000
H	-1.75843600	2.81765000	-4.37812000
H	1.48726400	3.93594800	-1.73906900

H	0.64549000	3.42111500	-4.02915800
P	4.73930200	0.81250400	-0.54115900
C	6.03524900	0.87590800	0.77935900
C	5.03876600	-0.88864400	-1.21493600
C	7.38359800	0.66045700	0.42061000
C	5.73443600	1.22258300	2.11129200
C	5.38743200	-1.99376800	-0.41141600
C	4.88458400	-1.07833600	-2.60191900
C	8.40176500	0.76980400	1.37532300
H	7.63482300	0.39338300	-0.61261800
C	6.75783200	1.34012700	3.06385300
H	4.69403400	1.39323900	2.40669300
C	5.52698000	-3.26738000	-0.97785700
H	5.56070100	-1.84999800	0.66032600
C	5.02750800	-2.35249900	-3.17137600
H	4.64719000	-0.21683600	-3.23631000
C	8.09244100	1.11146100	2.70153300
H	9.44204000	0.58937300	1.08285100
H	6.50732200	1.60630800	4.09669400
C	5.33892400	-3.45112500	-2.35776900
H	5.78916500	-4.12054700	-0.34257700
H	4.89880100	-2.48646700	-4.25079200
H	8.88928100	1.19896100	3.44774300
H	5.44921600	-4.44766900	-2.79851200
H	0.24536300	1.58617300	1.68932200

transition state **TS5'**

C	5.06069000	2.48767000	-2.72093500
C	3.81429000	3.05293700	-2.41774500
C	2.90051000	2.34510900	-1.62507500
C	3.22464200	1.07226000	-1.12330600
C	4.47767400	0.50772300	-1.43460200
C	5.38887700	1.21374700	-2.23014300
P	1.97768500	0.23148000	-0.04834300
C	2.78215300	0.28511100	1.61722500
C	3.95178000	-0.45269200	1.89759600
C	4.54821100	-0.37667200	3.16197400
C	3.98602300	0.43526300	4.16049000
C	2.82695000	1.17340200	3.88621100
C	2.22732800	1.10215800	2.62007500
C	2.20772800	-1.52168500	-0.51888700
C	2.26958300	-2.66350400	0.36622400
C	2.61607100	-3.81492200	-0.41749900
C	2.73342700	-3.40308400	-1.78973400
C	2.45901200	-1.99569800	-1.85928900
Fe	0.87333500	-3.01001800	-1.07858900
C	-0.32994200	-3.91940700	-2.45311800
C	-0.76822000	-2.57767600	-2.20729300
C	-1.08031100	-2.46127200	-0.80037100
C	-0.81011300	-3.74032800	-0.18526900
C	-0.35071800	-4.63453500	-1.20699400
P	-1.67055600	-0.94877800	0.01915500
C	-3.48272800	-0.99588100	-0.29592300
C	-4.27209000	0.03032100	0.27026700
C	-5.64920600	0.07906300	0.02721200
C	-6.25459600	-0.88020600	-0.80134800
C	-5.47454500	-1.89279300	-1.37737400
C	-4.09583800	-1.95597000	-1.12416200
Pd	-0.19632300	0.96989600	-0.49760600
C	-0.57046600	2.19488200	1.12095100
C	-1.49663500	1.91665900	2.14706900
C	-1.78227100	2.84948600	3.15946900
C	-1.14047000	4.09502100	3.17555800
C	-0.21197100	4.39467000	2.16729800
C	0.06699600	3.45604000	1.16148600
C	-1.56946200	-1.49799000	1.77689000
C	-2.60938000	-2.19388400	2.42283500
C	-2.44513400	-2.62848500	3.74647100
C	-1.24603500	-2.37843700	4.43190000
C	-0.20891600	-1.68131900	3.79302800
C	-0.37646800	-1.24039900	2.47574500
H	2.73227500	-4.83254000	-0.04020600
H	2.10391200	-2.64232100	1.44367700
H	2.44398300	-1.37444600	-2.75526100
H	2.95221300	-4.05396900	-2.63820400
H	-0.92525500	-3.96711800	0.87483000
H	-0.83379300	-1.77611400	-2.94186300
H	-0.03692400	-5.66939900	-1.05722200
H	0.00201900	-4.31534100	-3.41458500
H	-2.00327000	0.94752900	2.18366000
H	0.80813600	3.71875900	0.39784800
H	-2.51072600	2.59461900	3.93878600

H	0.30417000	5.36242700	2.16446800
H	-1.35892300	4.82355400	3.96430300
H	-3.54493700	-2.39384000	1.89050800
H	0.40829400	-0.66025900	1.98578800
H	-3.25911700	-3.16654100	4.24450400
H	0.72494300	-1.46053700	4.32091700
H	-1.12535100	-2.71791100	5.46628800
H	-3.79850100	0.80064600	0.88833400
H	-3.49272500	-2.75361700	-1.56895400
H	-6.25057600	0.87680800	0.47643500
H	-5.94085100	-2.64357000	-2.02465500
H	-7.33066300	-0.83480500	-0.99984900
H	4.39210200	-1.09752900	1.13146300
H	1.31903100	1.67221000	2.40883700
H	5.45480000	-0.95539500	3.36948400
H	2.37865900	1.80979300	4.65628500
H	4.45337300	0.48973700	5.14968600
H	1.91403900	2.76258700	-1.39278600
H	4.73711800	-0.49054200	-1.06900800
H	3.54781400	4.04201200	-2.80504600
H	6.35906200	0.76621700	-2.47157600
H	5.77502600	3.03472200	-3.34541100
N	0.07944300	0.37620500	-2.49404800
N	-0.50516900	1.22480100	-3.06808200
C	-1.39035100	2.40815100	-1.97133100
C	-2.76648300	2.17617600	-1.82047300
C	-0.87695700	3.71311800	-1.95095300
C	-3.61485300	3.24869700	-1.51546700
H	-3.16643500	1.16138900	-1.90816900
C	-1.73112400	4.78372200	-1.64947800
H	0.18620600	3.89416000	-2.14560600
C	-3.09528600	4.55090900	-1.41574200
H	-4.68228100	3.06443900	-1.34974700
H	-1.32940400	5.80145300	-1.59061500
H	-3.75840800	5.38766800	-1.17220200

transition state **TS5**$^{\mathbf{dtbpf}}$

P	1.20165900	-1.83355800	0.65042600
C	-0.28002900	-2.43892300	-0.25217000
C	-1.61440800	-2.77034000	0.21111400
C	-2.36499000	-3.30453700	-0.88644800
C	-1.51638400	-3.31172700	-2.04124800
C	-0.24314500	-2.77309300	-1.66378300
Fe	-1.70299700	-1.41509500	-1.31345600
C	-1.14989400	0.10892600	-2.54236100
C	-1.28809600	0.56601500	-1.18955900
C	-2.65280600	0.34669800	-0.75263800
C	-3.32963500	-0.29976100	-1.85574100
C	-2.41276500	-0.43368300	-2.95024000
P	-3.25043800	1.09410200	0.81683600
Pd	2.08759400	-0.02777700	-0.49840900
C	2.55562700	1.23030200	1.05112600
C	1.72525500	2.31220100	1.41231100
C	2.15275300	3.30022000	2.31601600
C	3.43439100	3.23341500	2.87974700
C	4.28322600	2.17211800	2.52814100
C	3.84505900	1.18671900	1.62921500
H	-3.40586000	-3.63006200	-0.84714000
H	-2.00649900	-2.62254000	1.21376800
H	0.59510600	-2.58317200	-2.33397200
H	-1.79902500	-3.62827700	-3.04659700
H	-4.36592400	-0.63668700	-1.86327900
H	-0.48149400	0.97266600	-0.57375700
H	-2.62752300	-0.90258100	-3.91240200
H	-0.23694000	0.11410100	-3.13680700
H	0.72232800	2.40361500	0.97766600
H	4.52905400	0.36618700	1.37439600
H	1.48030800	4.12706300	2.57588600
H	5.29032300	2.10994200	2.95852000
H	3.77072500	4.00142300	3.58528300
N	2.02824600	-0.81083400	-2.42811100
N	2.51513700	0.11347500	-3.00660100
C	2.79942100	1.49091200	-2.01401800
C	1.79807900	2.48291400	-2.03980100
C	4.13448200	1.82981600	-1.71926500
C	2.11507200	3.78979400	-1.65278200
H	0.77469400	2.22312800	-2.32934700
C	4.43962300	3.13745500	-1.32939900
H	4.91083000	1.05711500	-1.74146900
C	3.43000000	4.11369500	-1.28255900
H	1.33069400	4.55380200	-1.62279700
H	5.46340900	3.39484000	-1.03855100

H	3.67151300	5.13153400	-0.96050100
C	0.59677100	-1.56799100	2.43949000
C	1.69828600	-1.00335400	3.36114300
C	-0.48797000	-0.47718900	2.30681200
C	0.04335700	-2.85260600	3.09263400
H	2.45191200	-1.75577900	3.63558500
H	2.20377000	-0.13946000	2.90899600
H	1.21138000	-0.66385500	4.29405900
H	-1.25507300	-0.70491600	1.55438600
H	-0.99112200	-0.33555900	3.28048600
H	-0.02648500	0.48170100	2.02427500
H	-0.45537600	-2.57331700	4.03939800
H	-0.68607700	-3.39169900	2.47119100
H	0.85767200	-3.55146500	3.34644500
C	2.38646600	-3.34289300	0.56322600
C	1.63138900	-4.68278900	0.65030300
C	3.12937100	-3.26839100	-0.78620600
C	3.43588800	-3.23430700	1.68587500
H	1.08270700	-4.79525500	1.59749900
H	0.91513600	-4.79508200	-0.17980800
H	2.36374800	-5.50943600	0.58336700
H	3.71611400	-2.33653300	-0.86387900
H	3.82212900	-4.12779900	-0.85799300
H	2.44470100	-3.30947800	-1.64675100
H	4.23526900	-3.97510500	1.49895400
H	3.89904800	-2.23254500	1.71150300
H	3.00765900	-3.44936100	2.67745900
C	-3.53024400	2.89592800	0.17753800
C	-4.42276700	3.65436100	1.17846800
C	-2.12533200	3.54114800	0.20453100
C	-4.10017500	3.00618800	-1.24702400
H	-5.47139200	3.31539700	1.14141800
H	-4.05508400	3.54256700	2.21406800
H	-4.41414100	4.73306000	0.93173700
H	-1.44071200	3.05546700	-0.51044000
H	-2.20428000	4.60778500	-0.08068500
H	-1.67407300	3.48215700	1.21001500
H	-4.18719400	4.07420700	-1.52730000
H	-3.43829200	2.51777300	-1.98119000
H	-5.10065700	2.55476700	-1.33430100
C	-4.94781000	0.26901300	1.15149600
C	-6.09822800	0.57051600	0.17507900
C	-4.66479900	-1.24848700	1.18473400
C	-5.36896200	0.68780600	2.57993400
H	-6.38581300	1.63431500	0.20099900
H	-5.84966700	0.31208000	-0.86626700
H	-6.99005200	-0.01927900	0.46384000
H	-3.88785300	-1.48150900	1.93434300
H	-5.58635600	-1.78971100	1.47188100
H	-4.31904200	-1.62874000	0.21154400
H	-6.25324100	0.09881200	2.88989500
H	-4.55605700	0.49711600	3.30218700
H	-5.63876000	1.75363000	2.64272800

transition state **TS5**$^{\mathbf{dppb}}$

C	1.41145300	4.18447100	1.71984000
C	0.10368800	4.10747400	2.21927000
C	-0.70523500	3.00255700	1.91361500
C	-0.20679300	1.96539500	1.10350900
C	1.11189700	2.04585500	0.60918400
C	1.91457400	3.14968500	0.91667500
P	-1.21239600	0.50402400	0.63100700
C	-2.87899000	0.85604600	1.30299400
C	-3.41023000	0.22389300	2.44199100
C	-4.71687900	0.50811600	2.86508200
C	-5.50293500	1.42845100	2.15852600
C	-4.98180100	2.06163300	1.01891500
C	-3.68306600	1.76928600	0.58777500
Pd	-1.26190300	-0.10939800	-1.60188700
C	-0.03141300	1.38349900	-2.23695200
C	-0.38832100	2.73768400	-2.06461600
C	0.47779100	3.77431000	-2.44585400
C	1.73564200	3.47740600	-2.99077900
C	2.10172800	2.13687300	-3.18317300
C	1.21826900	1.10370400	-2.82636300
H	-1.34611600	2.99829300	-1.59853700
H	1.52140100	0.06714500	-3.01178000
H	0.17541000	4.81756100	-2.29652800
H	3.07840000	1.88999900	-3.61647100
H	2.42284900	4.28412000	-3.26920100
H	-2.80908600	-0.49820400	3.00015600
H	-3.28567600	2.24017000	-0.31853600

H	-5.12065200	0.00666600	3.75109500
H	-5.59383200	2.77550900	0.45765400
H	-6.52235200	1.64993100	2.49182600
H	-1.72773400	2.95014300	2.30083200
H	1.49644500	1.26410400	-0.05180900
H	-0.29337100	4.91180800	2.84782200
H	2.92507600	3.20932500	0.50183900
H	2.03571100	5.05461700	1.94932400
N	-1.28160700	-1.19845200	-3.34958800
N	-1.97494800	-2.08934600	-2.97294400
C	-2.83052800	-1.76246900	-1.48855500
C	-2.76478800	-2.70087500	-0.44081400
C	-3.97785800	-0.96383800	-1.66183300
C	-3.81837300	-2.77728400	0.47718800
H	-1.88064200	-3.33727500	-0.33434500
C	-5.03118600	-1.05184800	-0.73850500
H	-4.03635200	-0.27616500	-2.51433500
C	-4.95086100	-1.95449900	0.33030500
H	-3.75866100	-3.47943800	1.31657000
H	-5.91224000	-0.41249900	-0.85330600
H	-5.77162900	-2.02103500	1.05106600
C	-0.48234500	-0.85985200	1.65402500
C	-0.07756100	-0.57411900	3.11674000
H	0.40553100	-1.14714900	1.06345400
H	-1.17158900	-1.71759800	1.57281500
C	1.24861500	-1.27798000	3.46519800
H	0.03476300	0.51302500	3.28454600
H	-0.87213000	-0.91593100	3.80369300
C	2.44879700	-0.61747500	2.74579800
H	1.41109000	-1.25683900	4.55692600
H	1.16057900	-2.34624500	3.18910200
H	2.09473200	0.05904800	1.94753700
H	3.02287200	0.00596200	3.45409100
P	3.64378500	-1.85113500	1.98390500
C	2.52568500	-2.55194300	0.67582000
C	2.21809300	-1.87878500	-0.52624900
C	1.92266800	-3.80168000	0.92722300
C	1.32333400	-2.44078400	-1.44615100
H	2.68120100	-0.91099300	-0.74566200
C	1.01587700	-4.35718800	0.01111000
H	2.17496200	-4.34405100	1.84597500
C	0.71805600	-3.67913700	-1.17950700
H	1.09735700	-1.91560500	-2.37854300
H	0.55511300	-5.32878800	0.22123900
H	0.02121500	-4.11006400	-1.90598400
C	4.68245500	-0.73574000	0.93746300
C	5.52387600	-1.33876100	-0.02421000
C	4.70438000	0.66756100	1.06285100
C	6.33770800	-0.56068200	-0.85479700
H	5.52486500	-2.42991000	-0.13151000
C	5.53099500	1.44615600	0.23733700
H	4.06782600	1.16838000	1.79746700
C	6.34382300	0.83790600	-0.72759100
H	6.97190100	-1.04680300	-1.60404600
H	5.53253000	2.53631100	0.34757100
H	6.98137100	1.44791300	-1.37608400

transition state **TS5’** ^{dppb}

C	5.75901600	-1.61401600	-0.43640000
C	5.30353200	-0.30400100	-0.65170500
C	4.03534300	0.08630000	-0.20809600
C	3.20458500	-0.83419600	0.46803700
C	3.66791400	-2.14794100	0.68321900
C	4.93865900	-2.53327200	0.23247600
P	1.50314800	-0.32393000	0.92050100
C	1.76834300	1.27818100	1.78121700
C	2.84728000	1.45585900	2.67052400
C	2.98888300	2.65996300	3.37257600
C	2.05779900	3.69599700	3.19347200
C	0.98561300	3.52731800	2.30603500
C	0.84548700	2.32350800	1.60193500
P	-2.04426000	0.35353700	0.37596600
C	-3.66769800	-0.44165800	0.02918600
C	-4.12817500	-0.41554200	-1.30541800
C	-5.30880000	-1.07448000	-1.66401600
C	-6.04344200	-1.78418100	-0.70059600
C	-5.59282500	-1.81962600	0.62595800
C	-4.41447200	-1.15174000	0.99178600
Pd	0.01274900	-0.32573300	-0.82436700
C	1.34472600	0.88245500	-1.86432600
C	1.57320100	2.25537500	-1.64139400
C	2.54630800	2.96433300	-2.36842300

C	3.31489200	2.31405400	-3.34401100
C	3.08761800	0.95281700	-3.59582500
C	2.11673000	0.24955600	-2.86514200
C	-2.41491800	2.12055500	-0.02167500
C	-1.86424300	2.65554600	-1.20257300
C	-2.09544100	3.99478300	-1.54889300
C	-2.87062300	4.81190200	-0.71417800
C	-3.42647200	4.28371800	0.46258700
C	-3.20679500	2.94313400	0.80511800
H	0.99849000	2.79884800	-0.88658000
H	1.97117100	-0.81483700	-3.08064400
H	2.70181100	4.03083200	-2.16482600
H	3.67355900	0.42982000	-4.36098200
H	4.07768400	2.86326400	-3.90717700
H	-1.23444800	2.01608600	-1.83255000
H	-3.65781100	2.53899500	1.71735300
H	-1.65673300	4.39896000	-2.46719000
H	-4.03796400	4.91842000	1.11334700
H	-3.04412200	5.86074500	-0.97812900
H	-3.55363600	0.12378200	-2.06615900
H	-4.08253200	-1.19373900	2.03340200
H	-5.65255800	-1.04152000	-2.70321300
H	-6.16131300	-2.36787900	1.38520800
H	-6.96218400	-2.30811800	-0.98423200
H	3.58133500	0.65361300	2.80412300
H	0.02042700	2.19214600	0.89518400
H	3.83077300	2.79229700	4.06059100
H	0.25883600	4.33165100	2.15131700
H	2.17396500	4.63662900	3.74238900
H	3.68344800	1.10542300	-0.39537600
H	3.03700400	-2.87880500	1.19572400
H	5.93296500	0.42020100	-1.17903100
H	5.28694000	-3.55711000	0.40721500
H	6.75013600	-1.91747300	-0.79008700
N	-0.99639900	-0.84252200	-2.58313100
N	-1.26854500	-1.96858400	-2.39615200
C	-0.61082800	-2.67914700	-0.75731800
C	0.67254700	-3.23723400	-0.90278000
C	-1.55135000	-3.28953300	0.08934800
C	1.04452900	-4.34770500	-0.12342300
H	1.38448000	-2.80869600	-1.61821900
C	-1.17528600	-4.39501800	0.86585300
H	-2.57380300	-2.90123200	0.14405900
C	0.12666200	-4.92102200	0.76943300
H	2.05038600	-4.76998400	-0.22814900
H	-1.90311300	-4.85574400	1.54424100
H	0.41300700	-5.79161600	1.36909500
C	1.06615900	-1.47599200	2.31904200
C	0.00310000	-1.00205100	3.32670000
H	2.00758100	-1.66439100	2.86665200
H	0.75647900	-2.42899200	1.85334700
C	-1.47006100	-0.96581600	2.86690300
H	0.06332500	-1.71405800	4.17017800
H	0.29140200	-0.01750500	3.74174500
C	-1.96337900	0.36049600	2.23577700
H	-1.65630000	-1.81640900	2.18778400
H	-2.09202800	-1.15023800	3.76121000
H	-2.96266500	0.62142000	2.62737400
H	-1.29388200	1.19247800	2.51394000

transition state **TS5[2c]**

C	1.26468300	-3.62694300	-3.20384700
C	1.27130200	-4.07384500	-1.87291000
C	1.53284400	-3.17590500	-0.82928500
C	1.77991100	-1.81800100	-1.11002800
C	1.79167700	-1.38185200	-2.44748200
C	1.53136100	-2.27997200	-3.49083400
P	2.01078100	-0.57238400	0.22218500
C	2.97702100	-1.47440100	1.49951700
C	2.70732900	-1.24538800	2.86321800
C	3.48381500	-1.86945400	3.84891100
C	4.53029100	-2.72876400	3.48193300
C	4.80144400	-2.95816700	2.12506600
C	4.03541000	-2.33036400	1.13505800
C	0.37932400	-0.47129900	1.01553900
C	-0.22383500	0.76862200	1.43723300
C	-1.47701500	0.46789700	2.05852200
C	-1.65862800	-0.95761200	2.03535000
C	-0.51880100	-1.54203600	1.39243300
Fe	-1.42936900	-0.23859400	0.13523700
C	-2.80435200	-1.17187000	-1.02810600
C	-1.53503900	-1.13856700	-1.69371100

C	-1.14088300	0.23699700	-1.83311500
C	-2.16851700	1.05170500	-1.25942900
C	-3.21039500	0.18893100	-0.75201400
Pd	3.05350000	1.31393500	-0.65496600
C	4.74031700	0.18507500	-0.77600900
C	5.64490200	0.23567900	0.30431500
C	6.83838800	-0.50299800	0.27912300
C	7.14321100	-1.31505100	-0.82326500
C	6.25542100	-1.36480200	-1.90793800
C	5.06755000	-0.61299000	-1.88803200
H	-2.18140700	1.20179600	2.45053000
H	0.21008700	1.75329900	1.28445200
H	-0.36927400	-2.60416500	1.19910200
H	-2.53076900	-1.49919500	2.40376400
H	-2.17147100	2.13815400	-1.18513400
H	-0.95195400	-2.00679700	-2.00355400
H	-3.35282200	-2.07138500	-0.74848700
H	5.41155800	0.83684600	1.19052200
H	4.39595600	-0.66596400	-2.75077400
H	7.52465500	-0.45315000	1.13247900
H	6.48627600	-1.98928400	-2.77915500
H	8.06892000	-1.90088200	-0.83957700
H	1.88494800	-0.58274600	3.15271300
H	4.26934200	-2.49504800	0.07876800
H	3.26636200	-1.68712300	4.90678800
H	5.62370700	-3.61804300	1.82991100
H	5.13486800	-3.21766500	4.25334900
H	1.55846100	-3.53296100	0.20524400
H	2.01266600	-0.32763900	-2.65672600
H	1.07740200	-5.12784900	-1.64726800
H	1.54078400	-1.92875300	-4.52780400
H	1.06175200	-4.33155400	-4.01720100
N	3.82839900	2.83652100	-1.79420800
N	2.91301900	3.59470800	-1.75776200
C	1.74856200	3.31405700	-0.50860700
C	2.20733300	3.62679800	0.78865300
C	0.39867200	3.53420600	-0.84671800
C	1.31083100	4.07867400	1.76958600
H	3.27440500	3.52624400	1.02585300
C	-0.49937600	4.00170100	0.11653000
H	0.05633100	3.34465500	-1.86976500
C	-0.02880400	4.26167900	1.41291100
H	1.63568400	4.30331900	2.78955600
H	-1.55772200	4.16779600	-0.10550500
P	-4.72171300	0.85360900	0.04105000
C	-6.00887100	0.29115900	-1.16443500
C	-5.00549700	-0.35728000	1.41592200
C	-7.35546200	0.23299200	-0.74427400
C	-5.70549500	0.01022400	-2.51124200
C	-5.33311000	-1.71262000	1.20532800
C	-4.85815400	0.10970000	2.73642500
C	-8.36890300	-0.11697700	-1.64458300
H	-7.60881600	0.45251200	0.29953000
C	-6.72428800	-0.33197000	-3.41313400
H	-4.66656900	0.05117100	-2.85442200
C	-5.45931600	-2.58907700	2.29082100
H	-5.50032800	-2.07710100	0.18626700
C	-4.98776100	-0.76608000	3.82476800
H	-4.63635700	1.16924600	2.90739700
C	-8.05689400	-0.40015900	-2.98384100
H	-9.40762400	-0.16736500	-1.29996800
H	-6.47169700	-0.55203300	-4.45627100
C	-5.27861700	-2.11956100	3.60246500
H	-5.70517500	-3.64213400	2.11521100
H	-4.86450000	-0.39047200	4.84619400
H	-8.85006900	-0.67215000	-3.68833800
H	-5.37834400	-2.80655100	4.44958100
H	-0.20522100	0.59682200	-2.26195200
F	-0.90099300	4.70656500	2.34127800

transition state **TS5[2d]**

C	1.55219300	-3.60796900	-3.27780500
C	1.56900400	-4.07390300	-1.95355000
C	1.77721800	-3.17988100	-0.89454300
C	1.96280900	-1.80783900	-1.15283300
C	1.96561000	-1.35190800	-2.48372900
C	1.75631300	-2.24556700	-3.54239800
P	2.12099300	-0.56923400	0.19768400
C	3.09246200	-1.45103600	1.48713700
C	2.79059600	-1.23774700	2.84676400
C	3.56765200	-1.84195700	3.84428400
C	4.64724900	-2.66638200	3.49369100

C	4.95113300	-2.87945500	2.14120700	H	1.39090700	-4.30918700	-4.10337500
C	4.18465100	-2.27058300	1.13973900	N	3.95367600	2.98294100	-1.56246200
C	0.47159800	-0.55857900	0.96425400	N	3.02708100	3.72977600	-1.54880800
C	-0.20135000	0.64848300	1.37068600	C	1.72521100	3.29899700	-0.49817900
C	-1.43427500	0.28443600	1.99734200	C	1.92838600	3.64542000	0.85822600
C	-1.53640300	-1.14942000	1.98874400	C	0.43259500	3.40523500	-1.04316600
C	-0.36359700	-1.67478100	1.35282200	C	0.84647000	3.98658900	1.67380400
Fe	-1.34948500	-0.44209100	0.08234800	H	2.94451100	3.63968700	1.27173500
C	-2.71328800	-1.44349400	-1.03152300	C	-0.65339400	3.76861100	-0.24085400
C	-1.45042600	-1.40970100	-1.71223000	H	0.27532000	3.20982200	-2.10998000
C	-1.08605400	-0.03359200	-1.90773700	C	-0.45669000	4.03623200	1.13216100
C	-2.12139800	0.78161100	-1.35296700	H	0.97978500	4.21305200	2.73582400
C	-3.14125700	-0.07979300	-0.80055700	H	-1.65171200	3.84035800	-0.67895400
Pd	3.12820600	1.37726700	-0.59825500	P	-4.62362600	0.60357600	0.02286200
C	4.84973100	0.29286200	-0.69443700	C	-5.94413200	0.13267400	-1.18508700
C	5.73978100	0.36572900	0.39702400	C	-4.94187400	-0.63841900	1.36032600
C	6.95120100	-0.34324100	0.38922500	C	-7.29105700	0.16054000	-0.76342800
C	7.29250900	-1.14676300	-0.70896500	C	-5.65879500	-0.17130700	-2.53054300
C	6.42183700	-1.21745100	-1.80622900	C	-5.30738300	-1.97547300	1.10101400
C	5.21455700	-0.49661900	-1.80168100	C	-4.78861400	-0.22175300	2.69665600
H	-2.17563000	0.98209400	2.38671600	C	-8.32504500	-0.12695400	-1.66233600
H	0.17929000	1.65335000	1.21005900	H	-7.52885100	0.39759300	0.28032000
H	-0.15215800	-2.72886700	1.17339600	C	-6.69785600	-0.45039100	-3.43123800
H	-2.37743400	-1.73484900	2.36318800	H	-4.61894100	-0.19848200	-2.87238800
H	-2.13759300	1.86871700	-1.31955400	C	-5.46486700	-2.88479000	2.15485300
H	-0.85021500	-2.27602700	-1.99486400	H	-5.47912400	-2.29889800	0.06885000
H	-3.23879800	-2.34229100	-0.70873500	C	-4.94924700	-1.13042500	3.75328900
H	5.48072500	0.96338200	1.27847500	H	-4.53784200	0.82417700	2.90650700
H	4.55613200	-0.56799600	-2.67340700	C	-8.03179000	-0.43230700	-3.00108700
H	7.62410400	-0.27662900	1.25215700	H	-9.36481000	-0.11113600	-1.31761100
H	6.68103200	-1.83465700	-2.67479800	H	-6.46050800	-0.68953400	-4.47372300
H	8.23305500	-1.70885600	-0.71236100	C	-5.27778200	-2.46632800	3.48261500
H	1.94344100	-0.60174700	3.12432000	H	-5.74039900	-3.92343000	1.94171700
H	4.44605700	-2.41995800	0.08772700	H	-4.82086900	-0.79412400	4.78767800
H	3.32506000	-1.67095000	4.89862200	H	-8.84094200	-0.65506600	-3.70468600
H	5.79997800	-3.51043000	1.85833200	H	-5.40201100	-3.17895000	4.30492600
H	5.25247800	-3.13973000	4.27428500	H	-0.16449100	0.32977500	-2.36151700
H	1.80993400	-3.55058800	0.13491900	O	-1.46014000	4.33692100	2.00016300
H	2.14117400	-0.28581000	-2.67562500	C	-2.80216600	4.33116000	1.51464200
H	1.42443900	-5.13933600	-1.74498800	H	-3.08457100	3.34006900	1.11020200
H	1.75871000	-1.87908000	-4.57417600	H	-3.43482600	4.56615700	2.38287800

H	-2.95209900	5.10233400	0.73557000

transition state **TS5**[2e]

C	1.62479300	-4.75385500	-1.74176200
C	1.62818000	-4.69649200	-0.33908300
C	1.82002900	-3.47248900	0.31589700
C	2.00093600	-2.29196800	-0.43063500
C	2.01733200	-2.36073300	-1.83578000
C	1.82572700	-3.58452900	-2.49024200
P	2.13424700	-0.64314400	0.37019500
C	3.09987100	-0.96917900	1.89971900
C	2.77376300	-0.27741300	3.08302600
C	3.54422900	-0.45890000	4.23943700
C	4.64039800	-1.33428800	4.22532400
C	4.96735300	-2.02415500	3.04871000
C	4.20768600	-1.83986500	1.88681500
C	0.47794300	-0.35011000	1.05264800
C	-0.19696700	0.92391200	1.00914800
C	-1.44184500	0.79343800	1.70050000
C	-1.54996000	-0.55520500	2.18151100
C	-0.37072600	-1.26667800	1.78521300
Fe	-1.32113700	-0.54550800	0.14851900
C	-2.63904400	-1.89520600	-0.59519600
C	-1.35837600	-2.05751100	-1.21989200
C	-1.01294100	-0.81516800	-1.85602400
C	-2.08141200	0.10937700	-1.62960000
C	-3.10009800	-0.54672300	-0.84434200
Pd	3.12715500	0.85873100	-1.10002400
C	4.86455900	-0.14451900	-0.78343000
C	5.73445300	0.34662600	0.21102200
C	6.95787700	-0.28989900	0.47276800
C	7.32748200	-1.43420100	-0.24941900
C	6.47377400	-1.92378300	-1.24865700
C	5.25442600	-1.27825600	-1.51987100
H	-2.18545300	1.58052100	1.80976600
H	0.18379800	1.81697200	0.52095200
H	-0.16257500	-2.31878100	1.97931100
H	-2.40072800	-0.97708000	2.71855000
H	-2.12891300	1.14415400	-1.96457200
H	-0.73746700	-2.95392400	-1.18562100
H	-3.16164400	-2.65042900	-0.00791200
H	5.45175400	1.22011900	0.80934100
H	4.60858300	-1.67898800	-2.30748400
H	7.61710500	0.10427300	1.25478900
H	6.75537200	-2.81257600	-1.82561100
H	8.27723200	-1.93807000	-0.03878800
H	1.91248200	0.39862600	3.10000600
H	4.48481600	-2.36252900	0.96621900
H	3.28296100	0.08172000	5.15541500
H	5.82793700	-2.70055400	3.02593700
H	5.24005100	-1.47715300	5.13062900
H	1.84243600	-3.43563900	1.40971300
H	2.18768100	-1.44020800	-2.40816300
H	1.48580500	-5.61019700	0.24763900
H	1.83777600	-3.62569500	-3.58426400
H	1.47596800	-5.71210500	-2.25043800
N	3.89062600	1.95757100	-2.66245300
N	2.93860400	2.62640400	-2.90365800
C	1.71150800	2.71604700	-1.70557900
C	2.05117800	3.50409200	-0.58643700
C	0.38136400	2.69265300	-2.17135900
C	1.05370300	4.19874700	0.11353700
H	3.09960300	3.57655800	-0.27077900
C	-0.60658800	3.40104600	-1.48795800
H	0.13078900	2.12508000	-3.07385300
C	-0.27418300	4.14879700	-0.33864700
H	1.30438400	4.78485500	1.00194300
H	-1.64467200	3.38345300	-1.83471500
P	-4.65792400	0.29647000	-0.37525500
C	-5.89163500	-0.78206700	-1.23554000
C	-4.88933400	-0.25963700	1.37733700
C	-7.23477600	-0.76552300	-0.80070600
C	-5.55712400	-1.54194000	-2.37409000
C	-5.14864300	-1.59594500	1.74530300
C	-4.76965800	0.71464300	2.38748500
C	-8.21235000	-1.50652300	-1.47573400
H	-7.51324500	-0.17615700	0.08068100
C	-6.54036900	-2.27684400	-3.05323900
H	-4.52081800	-1.56574100	-2.72673900
C	-5.23841200	-1.95484900	3.09650000
H	-5.29103200	-2.35385600	0.96776400
C	-4.86091700	0.35652500	3.74088600

H	-4.59917100	1.75987800	2.10481400
C	-7.86878700	-2.26515400	-2.60593300
H	-9.24803300	-1.49059600	-1.11887000
H	-6.26290800	-2.86627200	-3.93414700
C	-5.08644400	-0.98070200	4.09733900
H	-5.43223300	-2.99739000	3.37183400
H	-4.75918200	1.12296300	4.51661800
H	-8.63400800	-2.84339500	-3.13461400
H	-5.15702200	-1.26349900	5.15307700
H	-0.08430100	-0.60144500	-2.38566200
C	-1.37334500	4.85815900	0.40753500
F	-0.89891900	5.67894800	1.37184400
F	-2.14790900	5.60111100	-0.41963200
F	-2.21316200	3.97154900	1.02050900

transition state **TS5**^{2f}

C	-1.21348400	-1.79842800	4.41140100
C	-1.22991700	-2.80274700	3.43050700
C	-1.50074600	-2.47835900	2.09434900
C	-1.74858000	-1.14123600	1.72779700
C	-1.75013100	-0.14316200	2.71936300
C	-1.47945000	-0.46794600	4.05508000
P	-1.99616100	-0.63773600	-0.02285400
C	-2.95445200	-2.02870700	-0.74645900
C	-2.68904700	-2.44213200	-2.06689600
C	-3.46265300	-3.45028100	-2.65777100
C	-4.50217100	-4.05527600	-1.93550300
C	-4.76939400	-3.64493700	-0.62118900
C	-4.00606800	-2.63177200	-0.02800000
C	-0.36737100	-0.88360000	-0.78777700
C	0.23492900	0.04176000	-1.71627000
C	1.48553400	-0.50757300	-2.14317300
C	1.66627400	-1.77627400	-1.49248300
C	0.52830200	-2.01265100	-0.65423200
Fe	1.44682500	-0.29140500	-0.10642900
C	2.84287400	-0.63194000	1.32201500
C	1.58491400	-0.31029400	1.93055500
C	1.18882000	0.99272500	1.47259100
C	2.20057100	1.47699800	0.58494200
C	3.23646200	0.47568000	0.47790500
Pd	-3.05944900	1.43308900	-0.08181100
C	-4.73951500	0.47745800	0.53590700
C	-5.64052800	0.02177500	-0.44763800
C	-6.82552700	-0.63807900	-0.08530900
C	-7.12351400	-0.86343200	1.26669400
C	-6.23766300	-0.40520900	2.25292100
C	-5.05837100	0.26894700	1.89041700
H	2.19232100	-0.02578800	-2.81919900
H	-0.19545100	0.99247500	-2.02195700
H	0.37666200	-2.87957700	-0.01122300
H	2.53689500	-2.42672900	-1.58589700
H	2.19409800	2.43375200	0.06561400
H	1.01139800	-0.95860900	2.59450900
H	3.38905600	-1.56720600	1.44483900
H	-5.41094600	0.15682500	-1.51065000
H	-4.38743400	0.62198300	2.67991500
H	-7.51024200	-0.98940400	-0.86593300
H	-6.46343900	-0.56826600	3.31341600
H	-8.04264000	-1.38792800	1.55038700
H	-1.87226100	-1.97786700	-2.62957600
H	-4.23619200	-2.30057600	0.98931600
H	-3.24845100	-3.76715900	-3.68411900
H	-5.58629700	-4.10346000	-0.05453800
H	-5.10437900	-4.84474200	-2.39780200
H	-1.53298300	-3.26739400	1.33619500
H	-1.96963600	0.89159200	2.42778300
H	-1.03600900	-3.84420100	3.70842600
H	-1.48011100	0.31756600	4.81774200
H	-1.00296400	-2.05536500	5.45489700
N	-3.82064400	3.30277100	0.28178400
N	-2.89921200	3.96630800	-0.09582600
C	-1.80435400	3.21195300	-1.07843800
C	-2.25451800	2.91742600	-2.38503900
C	-0.44871200	3.53783700	-0.90116200
C	-1.35448800	2.89521900	-3.46159400
C	0.45512400	3.54750100	-1.96058300
C	-0.00684300	3.22439700	-3.25029000
H	-1.71018100	2.64326500	-4.46546000
H	1.50489200	3.79629200	-1.78022600
P	4.72592900	0.72551700	-0.55693300
C	6.03060500	0.82639300	0.75270200
C	5.04038200	-0.98365100	-1.20320000

C	7.37925200	0.62586500	0.38664400
C	5.73415400	1.18636100	2.08204200
C	5.40693400	-2.07036500	-0.38262200
C	4.88072000	-1.19960400	-2.58570700
C	8.40260500	0.76282800	1.33219400
H	7.62679500	0.34880100	-0.64486500
C	6.76266900	1.33167700	3.02522200
H	4.69356800	1.34617900	2.38276300
C	5.55835900	-3.35201900	-0.92756900
H	5.58464700	-1.90560600	0.68538400
C	5.03532900	-2.48179900	-3.13368000
H	4.63008600	-0.35216500	-3.23381500
C	8.09782100	1.11775000	2.65598300
H	9.44331000	0.59384900	1.03443200
H	6.51576300	1.60828600	4.05617200
C	5.36443100	-3.56222900	-2.30285900
H	5.83457100	-4.19069500	-0.27906500
H	4.90228700	-2.63616400	-4.20985600
H	8.89869700	1.22715400	3.39495500
H	5.48415300	-4.56497800	-2.72676000
H	0.26869600	1.51810400	1.72552300
H	0.69657200	3.23220900	-4.08870500
F	-0.01751000	3.83840100	0.34503900
H	-3.32494300	2.73299600	-2.54313000

transition state **TS5**^{2g}

C	1.09628300	0.86219100	4.59041400
C	1.05950400	2.04828100	3.84008400
C	1.35207600	2.02473500	2.46961500
C	1.67853100	0.80978700	1.83679600
C	1.73030600	-0.37292500	2.59856900
C	1.43608800	-0.34849600	3.96834600
P	1.97334900	0.68190800	0.02571200
C	2.81696500	2.25911700	-0.40448600
C	2.55609000	2.85994600	-1.65215200
C	3.24863800	4.01571300	-2.03701100
C	4.20255600	4.58408000	-1.17960500
C	4.46601600	3.98777200	0.06194400
C	3.78421900	2.82702200	0.44787100
C	0.33372200	0.98073300	-0.70868400
C	-0.25672600	0.19432700	-1.76492700
C	-1.49818800	0.80783600	-2.13200600
C	-1.68473500	1.97582000	-1.31497300
C	-0.56072400	2.08488800	-0.43506500
Fe	-1.49475000	0.30568000	-0.15014800
C	-2.87100500	0.40736100	1.34058400
C	-1.61794400	-0.05803200	1.85766400
C	-1.25532600	-1.25393000	1.14704700
C	-2.29076200	-1.52986700	0.19526000
C	-3.30340100	-0.50463300	0.30425100
Pd	3.23570700	-1.23550700	-0.37256100
C	4.82653400	-0.20355200	0.35887700
C	5.67573000	0.48045400	-0.53403800
C	6.79019800	1.19275400	-0.06377200
C	7.07113300	1.23983600	1.30996000
C	6.23947800	0.55314200	2.20681500
C	5.13001000	-0.16929600	1.73359300
H	-2.19785600	0.42897900	-2.87794300
H	0.16881500	-0.71489300	-2.18717800
H	-0.41200300	2.85701300	0.31982200
H	-2.54883100	2.64082700	-1.33231900
H	-2.30984900	-2.34176400	-0.53085300
H	-1.02319500	0.43279200	2.62867800
H	-3.39339000	1.31084500	1.65523900
H	5.45838800	0.48173200	-1.60808700
H	4.49614300	-0.69799500	2.45358100
H	7.43380900	1.72386900	-0.77483100
H	6.45330500	0.57567700	3.28214700
H	7.93557000	1.80328400	1.67861400
H	1.80679200	2.42436600	-2.32151700
H	4.01990200	2.35156500	1.40449500
H	3.03840400	4.47535500	-3.00876000
H	5.21928300	4.41512800	0.73169000
H	4.74199900	5.48843300	-1.48098100
H	1.33894200	2.95511300	1.89318900
H	2.00882200	-1.31157800	2.10205500
H	0.80589500	2.99677100	4.32544900
H	1.47760900	-1.27466000	4.55090600
H	0.86876700	0.88426100	5.66141700
N	4.24626900	-3.01471800	-0.36768300
N	3.36290800	-3.72696900	-0.75046700
C	2.05764200	-2.99576300	-1.42846100

C	2.22261900	-2.53702600	-2.75099000
C	0.80626300	-3.52824000	-1.02144000
C	1.14244000	-2.54746700	-3.64839500
C	-0.25916300	-3.57901700	-1.93142500
C	-0.08646700	-3.08168400	-3.23754600
H	1.26895800	-2.16488900	-4.66588200
H	-1.22446600	-4.00170500	-1.64057800
P	-4.81778600	-0.52719100	-0.72450900
C	-6.10816600	-0.74364200	0.58516400
C	-5.05092800	1.26657400	-1.12729900
C	-7.44527500	-0.39964900	0.29021100
C	-5.81828900	-1.32955600	1.83334400
C	-5.33885500	2.25138800	-0.16004400
C	-4.90209300	1.65674800	-2.47233800
C	-8.46165500	-0.61849500	1.22797400
H	-7.68849700	0.05436100	-0.67756600
C	-6.84014500	-1.55543500	2.76772600
H	-4.78654700	-1.60145500	2.07921600
C	-5.42632500	3.60077800	-0.52559500
H	-5.50476100	1.95507900	0.88103600
C	-4.99377100	3.00740200	-2.84073400
H	-4.70727500	0.89179700	-3.23259000
C	-8.16287400	-1.19868000	2.47102600
H	-9.49243400	-0.33603800	0.98718400
H	-6.59754500	-2.00710100	3.73602000
C	-5.24625900	3.98258700	-1.86550000
H	-5.64088300	4.35885800	0.23562600
H	-4.86982700	3.29769200	-3.88953000
H	-8.95832700	-1.37095400	3.20383700
H	-5.31556600	5.03819800	-2.14898600
H	-0.33480400	-1.82444300	1.27436200
H	-0.92886500	-3.12162700	-3.93570600
H	3.21647200	-2.20078600	-3.07174100
O	0.75904100	-3.97729600	0.26386300
C	-0.34619700	-4.78831100	0.65995900
H	-0.10724300	-5.14744700	1.67118100
H	-1.28458700	-4.20564400	0.69226800
H	-0.46678600	-5.64942300	-0.02286400

transition state **TS5**[2h]

C	1.13718700	1.59182700	4.49264300
C	1.16122400	2.68440800	3.61136200
C	1.45246400	2.48897800	2.25451800
C	1.71814900	1.19417000	1.76847500
C	1.70913800	0.10551500	2.66000300
C	1.41481500	0.30210600	4.01575800
P	1.99826000	0.85531800	-0.01607300
C	2.90235100	2.33884400	-0.61760800
C	2.63633500	2.83665300	-1.90869300
C	3.37329600	3.91673200	-2.41288800
C	4.37661300	4.51072300	-1.63271800
C	4.64544200	4.01596600	-0.34830700
C	3.91946900	2.93047400	0.15753200
C	0.36588200	1.10624400	-0.77451000
C	-0.25764600	0.20622100	-1.71434200
C	-1.48175400	0.80343200	-2.15718200
C	-1.62532600	2.07399400	-1.49974900
C	-0.49251200	2.26388000	-0.64339000
Fe	-1.48194500	0.56951000	-0.12768300
C	-2.92021500	0.92265100	1.25860400
C	-1.69681200	0.54326500	1.90441200
C	-1.32641900	-0.75983500	1.42699600
C	-2.31403200	-1.18515900	0.48253500
C	-3.31425900	-0.14807000	0.36919500
Pd	3.18879200	-1.12299300	-0.30151700
C	4.81226400	-0.15140100	0.42922900
C	5.72663800	0.41612600	-0.48014900
C	6.86164900	1.10146800	-0.01887500
C	7.09660300	1.23506300	1.35764700
C	6.19844300	0.66105400	2.26914800
C	5.06692600	-0.03493100	1.80836100
H	-2.20169900	0.34574400	-2.83783600
H	0.13961400	-0.76004400	-2.01498800
H	-0.31323300	3.12482800	0.00079600
H	-2.47123700	2.75544500	-1.59758300
H	-2.31116000	-2.12221900	-0.07261200
H	-1.12610200	1.15164300	2.60766000
H	-3.44309200	1.87220200	1.37757000
H	5.54746700	0.34736300	-1.55875400
H	4.38165000	-0.47534800	2.53936500
H	7.55790900	1.54303300	-0.74126500
H	6.37600400	0.75067000	3.34735800

H	7.97758700	1.77770300	1.71781600
H	1.84784000	2.38139800	-2.51706000
H	4.15475500	2.53391900	1.14983800
H	3.15860600	4.29793600	-3.41700800
H	5.43544900	4.46461400	0.26259200
H	4.94982900	5.35659300	-2.02707800
H	1.48528100	3.34607000	1.57449700
H	1.93742000	-0.89656100	2.27828700
H	0.95542800	3.69388700	3.98291000
H	1.40711900	-0.55234800	4.70026700
H	0.90992300	1.74783700	5.55246500
N	4.16544800	-2.93521800	-0.36590400
N	3.31979000	-3.63476400	-0.83026100
C	1.96052600	-2.92239400	-1.51617100
C	2.15461600	-2.45327800	-2.83080600
C	0.69303700	-3.41998800	-1.13705900
C	1.08853900	-2.44134200	-3.74179200
C	-0.36451400	-3.42226600	-2.05670300
C	-0.16847100	-2.93482900	-3.35938200
H	1.24126600	-2.05003400	-4.75310100
H	-1.34473000	-3.79966700	-1.75192200
P	-4.75033900	-0.29905500	-0.75281200
C	-6.05787000	-0.84414300	0.44087500
C	-5.23143000	1.47911300	-0.96560600
C	-7.39371400	-0.88388400	-0.01383500
C	-5.77171700	-1.27685400	1.74998900
C	-5.80873400	2.24831800	0.06562300
C	-5.00715100	2.08017300	-2.21958400
C	-8.42063100	-1.32594800	0.82828100
H	-7.62947900	-0.55607100	-1.03342900
C	-6.80170900	-1.73064200	2.58845300
H	-4.74045700	-1.25379200	2.11663800
C	-6.11998900	3.59815500	-0.14532700
H	-6.02019400	1.78117700	1.03361600
C	-5.31727600	3.43186600	-2.43110500
H	-4.58563600	1.48049000	-3.03435100
C	-8.12706400	-1.75338300	2.13335100
H	-9.45376300	-1.34237000	0.46416800
H	-6.56474000	-2.06295000	3.60518400
C	-5.86943900	4.19424200	-1.39161600
H	-6.56412400	4.18747500	0.66441000

H	-5.13561000	3.88729400	-3.41067500
H	-8.92952900	-2.10461500	2.79076200
H	-6.11635600	5.24871600	-1.55509700
H	-0.43494300	-1.31813200	1.70685100
H	-1.00014900	-2.93882500	-4.07031300
H	3.15195700	-2.11102400	-3.13253400
C	0.50411300	-3.99162900	0.24569700
F	1.07610300	-3.20342600	1.20386700
F	-0.80745300	-4.11941500	0.57301000
F	1.06241600	-5.21558300	0.37210200

intermediate **H**

C	4.65914400	1.73611800	-2.99507600
C	3.38636200	1.30828400	-3.40444300
C	2.53275800	0.67819300	-2.49148900
C	2.95385000	0.44137100	-1.16647300
C	4.22863900	0.87142300	-0.76229800
C	5.07352300	1.52129500	-1.67378000
P	1.74833300	-0.30853300	0.00250200
C	2.72048800	-0.55350200	1.54623900
C	3.47967000	-1.71547600	1.78754000
C	4.17537400	-1.86227900	2.99552000
C	4.12531200	-0.85110800	3.96734500
C	3.38052600	0.31320100	3.72558500
C	2.67712900	0.46163200	2.52270500
C	1.64857000	-2.02036800	-0.65098800
C	1.02772400	-3.13784100	0.02855000
C	1.10663800	-4.28282100	-0.83022500
C	1.76785600	-3.88974300	-2.04090800
C	2.10938100	-2.50183500	-1.93317700
Fe	0.09141300	-2.75305200	-1.72990200
C	-0.87094900	-2.29590600	-3.48322600
C	-0.97259900	-1.22408200	-2.53881900
C	-1.60624600	-1.73108400	-1.33978800
C	-1.89702900	-3.12846300	-1.56662500
C	-1.44121100	-3.46889900	-2.88377900
P	-1.75929300	-0.66070400	0.12356000
C	-3.52782400	-0.17786800	0.14244800
C	-4.27643800	-0.16266000	1.33385300
C	-5.57440600	0.36726500	1.34254100
C	-6.12628800	0.89850800	0.16946300

C	-5.37976600	0.89167700	-1.01885200
C	-4.08824000	0.35571200	-1.03529400
Pd	-0.17983400	1.08042500	0.10977000
C	0.94818900	2.79149200	0.33361500
C	0.99874200	3.40555400	1.60519200
C	1.79920000	4.53482300	1.84293700
C	2.55180800	5.09734600	0.80113700
C	2.47820500	4.52888700	-0.47839000
C	1.68136300	3.39478100	-0.70658900
C	-1.58919100	-1.74900100	1.59342000
C	-2.36464300	-2.90861900	1.79566400
C	-2.12496600	-3.72980500	2.90527900
C	-1.12316000	-3.39318400	3.83116200
C	-0.37541900	-2.21956300	3.65857600
C	-0.61436200	-1.39829800	2.54775200
H	0.69971300	-5.27111000	-0.60926000
H	0.57686800	-3.10947200	1.01958600
H	2.61772700	-1.90419700	-2.68928000
H	1.95499200	-4.52594200	-2.90779500
H	-2.34389800	-3.81855000	-0.85189300
H	-0.62604500	-0.19511700	-2.65973400
H	-1.48706900	-4.46161700	-3.33557900
H	-0.41364800	-2.23774900	-4.47228400
H	0.39273300	3.00579700	2.42849400
H	1.64138500	2.97885800	-1.71826900
H	1.82360800	4.98404700	2.84334100
H	3.04461300	4.96801100	-1.30848300
H	3.17562100	5.98002700	0.98171400
H	-3.16488700	-3.16148800	1.09306100
H	-0.03243300	-0.48178600	2.39679400
H	-2.72423300	-4.63487900	3.05198100
H	0.39974100	-1.94155200	4.38001700
H	-0.93576000	-4.04243100	4.69310600
H	-3.84128000	-0.55350800	2.25908900
H	-3.50225500	0.37173000	-1.95948400
H	-6.15028000	0.37495000	2.27429800
H	-5.79953300	1.32015000	-1.93451300
H	-7.13432500	1.32628300	0.18118500
H	3.52219700	-2.50616100	1.03233700
H	2.09902200	1.37048200	2.32654000
H	4.76026300	-2.77039400	3.17760400
H	3.34330100	1.11082400	4.47510800
H	4.66907000	-0.97017700	4.91073800
H	1.52466000	0.38146700	-2.79907800
H	4.55594400	0.71248500	0.26939500
H	3.04988900	1.48427000	-4.43175700
H	6.06035500	1.86424300	-1.34543900
H	5.32157500	2.24455600	-3.70351600
C	-1.67773200	2.45304000	-0.10100500
C	-2.55474800	2.79505900	0.94643700
C	-1.92071400	3.00202200	-1.37606300
C	-3.65789600	3.63357700	0.72144900
H	-2.39266200	2.39010500	1.95264700
C	-3.02585000	3.83833600	-1.60633500
H	-1.23680700	2.78227600	-2.20520000
C	-3.90240000	4.15373100	-0.55820000
H	-4.33469700	3.87434600	1.54970800
H	-3.19723400	4.25010800	-2.60844500
H	-4.76645800	4.80394000	-0.73515300

transition state **TS6**

C	4.27437100	1.56232600	-3.39475300
C	2.91726500	1.25965700	-3.59311200
C	2.19045400	0.61193900	-2.58779000
C	2.81492300	0.23247100	-1.38038000
C	4.17460500	0.53503600	-1.19002800
C	4.89637100	1.20192800	-2.19167500
P	1.75821600	-0.50472900	-0.06337000
C	2.94099600	-0.80785500	1.30899900
C	3.58762100	-2.04070900	1.52361500
C	4.46483700	-2.19406100	2.60679100
C	4.71125100	-1.11933200	3.47490400
C	4.07382200	0.11271100	3.26029000
C	3.18560200	0.26810900	2.18853100
C	1.45247800	-2.18135200	-0.72254600
C	0.78074600	-3.23594500	0.00497200
C	0.68064500	-4.37811300	-0.85687700
C	1.26844700	-4.03653900	-2.12106000
C	1.74554700	-2.68649400	-2.04207700
Fe	-0.26758100	-2.76369300	-1.66718600
C	-1.34139200	-2.27826600	-3.34397300
C	-1.28985100	-1.17692100	-2.42907600

C	-1.86082600	-1.60252600	-1.17037500
C	-2.25894100	-2.98201600	-1.32323500
C	-1.93905200	-3.39132800	-2.66118000
P	-1.84521800	-0.50340500	0.27921700
C	-3.49974800	0.28885200	0.24903800
C	-4.12207100	0.67707500	1.45306200
C	-5.30569900	1.42556800	1.42825700
C	-5.87088200	1.81174400	0.20403000
C	-5.24676700	1.44238900	-0.99617800
C	-4.06889500	0.68588700	-0.97697900
Pd	-0.03759300	0.97023000	0.25461300
C	0.91021000	2.81858900	0.54867200
C	1.02275800	3.15287900	1.92196400
C	2.11251200	3.89611000	2.39801200
C	3.10521100	4.34446700	1.51229000
C	2.98249500	4.05632900	0.14146300
C	1.89447800	3.31747100	-0.33652600
C	-1.93459600	-1.63508600	1.72539000
C	-3.02807700	-2.49289600	1.95985900
C	-3.02287400	-3.35359600	3.06585100
C	-1.93319500	-3.35737000	3.95184500
C	-0.85281000	-2.48864000	3.73841600
C	-0.85658600	-1.62672700	2.63221700
H	0.20301600	-5.32641400	-0.60408700
H	0.40970200	-3.16765000	1.02771200
H	2.23539500	-2.12514500	-2.83804900
H	1.31734400	-4.68029800	-3.00126400
H	-2.68477600	-3.61374200	-0.54346500
H	-0.89043700	-0.17818400	-2.61521600
H	-2.08769600	-4.39090800	-3.07434000
H	-0.96490200	-2.27970400	-4.36826600
H	0.25388200	2.81626300	2.62725900
H	1.82400500	3.10259800	-1.40755200
H	2.18432600	4.12679000	3.46748100
H	3.74652700	4.40436200	-0.56309700
H	3.95595800	4.92688300	1.88179000
H	-3.88901900	-2.47362200	1.28296500
H	-0.01996500	-0.93944400	2.45337800
H	-3.87379600	-4.02082400	3.24123100
H	-0.00656900	-2.47713000	4.43343500
H	-1.93325600	-4.03122500	4.81537300
H	-3.67773300	0.39067500	2.41257200
H	-3.58473900	0.40720100	-1.91811300
H	-5.78416700	1.71471400	2.37039800
H	-5.67561900	1.75059400	-1.95551900
H	-6.79150700	2.40462300	0.18567200
H	3.40435200	-2.87782200	0.84268700
H	2.67641500	1.22472600	2.02837600
H	4.96246800	-3.15635300	2.76995800
H	4.26123700	0.95716700	3.93211400
H	5.39895700	-1.24301700	4.31852600
H	1.12414900	0.40663800	-2.72580900
H	4.66744600	0.26076200	-0.25252900
H	2.41989900	1.54460400	-4.52654600
H	5.95265100	1.44093900	-2.02735700
H	4.84097300	2.08406500	-4.17324600
C	-0.97633100	2.79768200	-0.12059800
C	-1.94799000	3.35468500	0.74276800
C	-1.10479800	3.04352700	-1.51013300
C	-3.01895800	4.09829600	0.23518800
H	-1.87220000	3.19485300	1.82368500
C	-2.18384200	3.78219500	-2.01612200
H	-0.34609300	2.65604600	-2.20055700
C	-3.14697200	4.31597500	-1.14683800
H	-3.76896200	4.50179700	0.92449000
H	-2.26664900	3.94712800	-3.09692900
H	-3.98812400	4.89676600	-1.53969500

transition state **TS7**

C	4.32609100	-1.15360800	-3.31523300
C	4.42616800	-2.00470700	-2.20247600
C	3.41383200	-2.02200600	-1.23424300
C	2.29143100	-1.18403000	-1.37514800
C	2.21155400	-0.31093600	-2.47539600
C	3.21734400	-0.30565300	-3.45058800
P	0.90863300	-1.14606100	-0.16344200
C	1.67613900	-1.86146800	1.34709500
C	1.61086700	-3.22486200	1.69373500
C	2.22268100	-3.67894100	2.87169200
C	2.91269200	-2.78229000	3.70169600
C	2.98665400	-1.42387700	3.35454400
C	2.36112300	-0.96244000	2.19052800

C	-0.15778200	-2.48903500	-0.78638900
C	-1.22956500	-3.13639900	-0.06052300
C	-1.87867700	-4.05218600	-0.95415900
C	-1.23237400	-3.96568400	-2.23315200
C	-0.17297300	-3.00419200	-2.13433700
Fe	-1.96669600	-2.14292100	-1.66724200
C	-2.71912300	-1.13474500	-3.28361500
C	-2.08681300	-0.23726500	-2.36351700
C	-2.74161500	-0.36457700	-1.07945700
C	-3.78465400	-1.35526900	-1.22593700
C	-3.76689400	-1.82311800	-2.58268900
P	-2.17695500	0.58912200	0.36923800
C	-3.49621300	1.86473800	0.54211000
C	-3.47347300	2.68279400	1.69072000
C	-4.40952800	3.71088500	1.84557900
C	-5.37183400	3.94340700	0.84853100
C	-5.39650500	3.13585800	-0.29649500
C	-4.46554600	2.09716100	-0.45015100
Pd	0.05725900	1.05175400	0.13603700
C	-2.56617800	-0.49189700	1.81648200
C	-3.88023500	-0.88732900	2.13641500
C	-4.11218400	-1.75137900	3.21486700
C	-3.03818000	-2.21365700	3.99376800
C	-1.73201300	-1.79283100	3.70450200
C	-1.50049900	-0.92858100	2.62467700
H	-2.73961000	-4.67671900	-0.70905400
H	-1.49795200	-2.94995200	0.97929400
H	0.50230600	-2.69410100	-2.93263900
H	-1.51531100	-4.51335200	-3.13392400
H	-4.44163400	-1.71511700	-0.43426300
H	-1.23243600	0.41522900	-2.55490900
H	-4.41197000	-2.60048100	-2.99693900
H	-2.43081000	-1.29335000	-4.32412000
H	-4.72265100	-0.50465200	1.55000500
H	-0.48500200	-0.58954600	2.39216400
H	-5.13543500	-2.06057100	3.45473800
H	-0.88915500	-2.13277400	4.31535000
H	-3.22442700	-2.88997500	4.83506100
H	-2.71116400	2.51236500	2.46018600
H	-4.48751400	1.46923300	-1.34652500
H	-4.38521100	4.33897400	2.74264700

H	-6.14456100	3.31308600	-1.07693900
H	-6.09881600	4.75407400	0.96564800
H	1.08390600	-3.92949100	1.04298200
H	2.39436400	0.10145500	1.92726800
H	2.16542600	-4.74015600	3.13780400
H	3.52879600	-0.71743400	3.99184900
H	3.39179600	-3.14233700	4.61857500
H	3.49612200	-2.68276800	-0.36543100
H	1.36125000	0.37457200	-2.54563800
H	5.29823000	-2.65746200	-2.08758600
H	3.14513300	0.38031800	-4.30104000
H	5.12106100	-1.14207900	-4.06881500
Si	0.53946300	3.29517600	-0.95545400
C	-1.29223400	3.81645800	-1.14192000
H	-1.78724800	3.98196900	-0.17073000
H	-1.31248600	4.77029700	-1.70788300
H	-1.88232100	3.06994400	-1.70046900
C	1.21463300	3.11095400	-2.71666100
H	0.55902500	2.47052200	-3.33491900
H	1.25674700	4.10710500	-3.19698400
H	2.23261800	2.68669800	-2.69875000
C	1.35649900	4.77785900	-0.09333000
H	2.45754300	4.70514700	-0.11095600
H	1.05783500	5.71657500	-0.59748000
H	1.03542700	4.84259000	0.96244900
N	1.81300600	1.98433800	0.19655800
N	2.89712100	1.92200400	-0.46004300
C	4.07395000	1.77964500	0.32138700
C	5.20213900	1.24788800	-0.33472300
C	4.15000500	2.12249500	1.68941400
C	6.38444900	1.02679800	0.37987200
H	5.11224400	0.98965300	-1.39401400
C	5.34280700	1.91675100	2.38915000
H	3.26428500	2.55057100	2.16876100
C	6.46087500	1.36090300	1.74208600
H	7.25328200	0.59286900	-0.12719200
H	5.40788300	2.19429200	3.44769500
H	7.39103500	1.19747800	2.29684000

intermediate **I**

C	4.13026900	-1.14605100	-3.39785200

C	4.25139200	-2.07625300	-2.35259700
C	3.29148200	-2.11982300	-1.33242500
C	2.20418100	-1.22638500	-1.35336600
C	2.10446600	-0.27524400	-2.38547900
C	3.05595800	-0.24456200	-3.41287900
P	0.88626000	-1.22495800	-0.07032100
C	1.70370800	-2.02094700	1.37387600
C	1.58413400	-3.38947200	1.68345900
C	2.21810400	-3.90973000	2.82161600
C	2.98017100	-3.07320900	3.65073300
C	3.10543900	-1.70917800	3.34170900
C	2.46345400	-1.18070200	2.21522700
C	-0.22172700	-2.52785700	-0.70823500
C	-1.32172500	-3.14866400	-0.00121400
C	-1.97133300	-4.05520100	-0.90341500
C	-1.29601000	-3.99176600	-2.16852500
C	-0.21793800	-3.05415300	-2.05249000
Fe	-2.00054500	-2.14823500	-1.63071200
C	-2.71385800	-1.15382100	-3.27567200
C	-2.06593800	-0.25434600	-2.37004600
C	-2.73409500	-0.33917600	-1.08854700
C	-3.80009600	-1.30840600	-1.22278200
C	-3.78307900	-1.80226100	-2.57032700
P	-2.11462400	0.59401500	0.34241900
C	-3.36273800	1.91418200	0.61205200
C	-3.06617100	2.88048000	1.59384300
C	-3.95497000	3.93317100	1.83618700
C	-5.14223800	4.03562200	1.09194800
C	-5.43853700	3.07760900	0.11252700
C	-4.55340200	2.01548900	-0.12753400
Pd	0.12065300	1.09664100	0.12034700
C	-2.44730800	-0.47930800	1.81041100
C	-3.74249800	-0.92555800	2.14007300
C	-3.93384900	-1.79296800	3.22345000
C	-2.83886900	-2.20262400	4.00288600
C	-1.55454000	-1.72140100	3.71163000
C	-1.36316800	-0.85674400	2.62426000
H	-2.85170700	-4.65806200	-0.67352700
H	-1.60576600	-2.95474900	1.03287800
H	0.47988600	-2.76251600	-2.83805100
H	-1.57261800	-4.53772400	-3.07228000
H	-4.47267500	-1.63948500	-0.43187800
H	-1.19618200	0.37426200	-2.56970800
H	-4.44509300	-2.57055200	-2.97435600
H	-2.42166700	-1.33964000	-4.31044300
H	-4.60415200	-0.58126200	1.55833100
H	-0.36301200	-0.47561400	2.39228500
H	-4.94207600	-2.14522900	3.46720700
H	-0.69609700	-2.01767700	4.32329800
H	-2.99283300	-2.88355000	4.84686500
H	-2.12202200	2.81247000	2.14712900
H	-4.77734700	1.27711400	-0.90410900
H	-3.71610500	4.68428600	2.59649200
H	-6.35979700	3.15975500	-0.47426800
H	-5.83198300	4.86693100	1.27234600
H	0.99567700	-4.04668100	1.03575600
H	2.53550100	-0.10981400	1.98263200
H	2.11924400	-4.97488700	3.05809200
H	3.70051500	-1.05020600	3.98296800
H	3.47489800	-3.48365000	4.53766100
H	3.38803300	-2.84414700	-0.51702200
H	1.28740100	0.45458500	-2.35291400
H	5.09779800	-2.77121100	-2.33180600
H	2.97011400	0.50334500	-4.20797300
H	4.88367400	-1.11567200	-4.19259600
Si	-0.12834900	3.21880300	-0.95715600
C	-1.83902700	3.67238600	-1.68210000
H	-2.55233800	4.01135600	-0.91247900
H	-1.70119900	4.50097200	-2.40460000
H	-2.30523600	2.83011700	-2.22576800
C	1.04756200	3.21566200	-2.45966200
H	0.61805200	2.62260000	-3.28966700
H	1.19806500	4.24837200	-2.83067100
H	2.03514300	2.79303600	-2.19669500
C	0.38274100	4.64632900	0.19720800
H	1.40836000	4.49026400	0.57725000
H	0.35199900	5.61926300	-0.33119800
H	-0.29362100	4.71194800	1.06927500
N	1.99417500	1.62388000	0.48540500
N	2.94080600	1.73605300	-0.30480200
C	4.25619800	1.82380700	0.27029300
C	5.32822100	1.47921200	-0.56964400

C	4.49016300	2.22618200	1.59938100
C	6.63739500	1.51421500	-0.07289800
H	5.10699300	1.16829800	-1.59521400
C	5.80219700	2.27597300	2.08174200
H	3.63279400	2.49790100	2.22327900
C	6.87758800	1.91530000	1.25075700
H	7.47448100	1.23115000	-0.72036600
H	5.99353300	2.60110800	3.11082400
H	7.90275800	1.95435600	1.63458800

intermediate **J**

C	4.58844100	2.86737200	-2.52500900
C	4.47653600	3.16240600	-1.15918000
C	3.76541800	2.30979400	-0.30595400
C	3.15063000	1.14918500	-0.81426000
C	3.24640200	0.86981900	-2.19357800
C	3.97206900	1.71749500	-3.03978900
P	2.01165700	0.16141200	0.22470700
C	2.53585300	0.48701100	1.95742400
C	3.87213200	0.31070300	2.36968400
C	4.22411600	0.50860600	3.71032100
C	3.24496300	0.87359300	4.64928900
C	1.91243200	1.03792400	4.24609800
C	1.55812200	0.84585800	2.90277400
C	2.60942600	-1.55803200	0.03220700
C	2.04438800	-2.63125400	0.81734800
C	2.60026000	-3.86906100	0.35525700
C	3.51117900	-3.57461700	-0.71419700
C	3.52489600	-2.15290600	-0.91404100
Fe	1.63517600	-2.88097500	-1.15750700
C	1.17796300	-2.77785000	-3.15367900
C	0.50503700	-1.75840300	-2.40628000
C	-0.30357100	-2.39431200	-1.38648900
C	-0.13584100	-3.82356900	-1.54371200
C	0.78522500	-4.05013900	-2.61942100
P	-1.22443500	-1.34077100	-0.22845300
C	-2.90635600	-1.31811900	-0.96738500
C	-3.97555700	-2.10220300	-0.49418800
C	-5.22976700	-2.02670400	-1.11556200
C	-5.42467700	-1.16750900	-2.20926300
C	-4.36458100	-0.37569400	-2.67502000
C	-3.11170700	-0.43806800	-2.05059200
Pd	-0.25793600	0.78770100	0.00348500
C	-1.47874600	-2.18821300	1.37885400
C	-1.12823600	-3.51233900	1.70058200
C	-1.34853100	-4.00767400	2.99506000
C	-1.92894100	-3.19211200	3.97603200
C	-2.28020500	-1.86929600	3.66260500
C	-2.04769900	-1.36591100	2.37776000
H	2.35625300	-4.86144800	0.73852500
H	1.31299500	-2.50533900	1.61667900
H	4.11396600	-1.61457700	-1.65624000
H	4.08242400	-4.30414900	-1.29130600
H	-0.61935800	-4.60395300	-0.95703000
H	0.59147900	-0.67605800	-2.52958500
H	1.14009800	-5.02586200	-2.95644100
H	1.88411900	-2.61325200	-3.96923100
H	-0.65874800	-4.15321600	0.95244600
H	-2.28976200	-0.32219800	2.13812300
H	-1.06349800	-5.03770900	3.23503100
H	-2.72412900	-1.22175300	4.42626900
H	-2.09958900	-3.58276600	4.98477400
H	-3.82552800	-2.77056400	0.36013300
H	-2.28774900	0.19811400	-2.39153000
H	-6.05720900	-2.64225000	-0.74634600
H	-4.51570100	0.30672000	-3.51794800
H	-6.40599100	-1.10966800	-2.69235900
H	4.63291200	0.01894700	1.63741600
H	0.51920400	0.96427900	2.56894000
H	5.26430700	0.37470700	4.02652100
H	1.14560700	1.31643900	4.97665100
H	3.52351500	1.02612200	5.69766100
H	3.65847100	2.56474000	0.75183300
H	2.73645100	-0.00450200	-2.60946600
H	4.93178000	4.07154100	-0.75343500
H	4.04223600	1.48652600	-4.10833600
H	5.14167800	3.53901500	-3.18990000
C	0.44338900	2.73683300	-0.25659200
C	0.72861600	3.18676900	-1.56399200
C	0.69110600	3.62593100	0.80800300
C	1.25738800	4.46556500	-1.79825700
H	0.55284100	2.52456300	-2.42063800

C	1.22574700	4.90711100	0.58206700
H	0.46929600	3.32360300	1.83887500
C	1.51579100	5.33001400	-0.72283100
H	1.47799700	4.78493400	-2.82402400
H	1.41500200	5.57659700	1.43055900
H	1.93694400	6.32596800	-0.90196800
N	-2.05282900	1.60078400	0.45166400
N	-2.87482300	1.96002100	-0.37859100
Si	-4.48598900	2.57511600	0.27243200
C	-5.29819800	1.14075800	1.18436900
H	-5.41403900	0.27141600	0.51297300
H	-4.67103200	0.82934200	2.03901000
H	-6.29661600	1.41590700	1.57170400
C	-5.46416500	3.09088400	-1.24873900
H	-5.65604700	2.21805800	-1.89757500
H	-6.43797200	3.53547500	-0.97282100
H	-4.90008200	3.83670500	-1.83751900
C	-4.09886400	4.02387300	1.41174800
H	-3.58739200	4.83131300	0.85739400
H	-5.01061600	4.44420600	1.87455300
H	-3.42021300	3.68712400	2.21629400

intermediate **K**

Si	-1.24226000	-0.42225600	3.81946900
N	-1.79569600	0.65498800	2.37437100
N	-2.40414600	1.66168500	2.82354700
C	-3.23068600	2.42749500	1.97048900
C	-4.27043600	1.78458300	1.26197000
C	-3.13488000	3.83277500	2.00640600
C	-5.23537300	2.57802700	0.62365700
H	-4.32722200	0.68335300	1.23066600
C	-4.06834100	4.60043600	1.30392000
H	-2.31922600	4.29905500	2.56677800
C	-5.13637200	3.97767000	0.63314000
H	-6.06148500	2.08811900	0.09668600
H	-3.97769400	5.69176500	1.29771000
H	-5.88844800	4.58713200	0.12064600
C	-1.76912400	-1.24273300	-1.25551200
C	-1.26342900	-2.56263800	-1.20700600
C	-2.44187300	-0.84144500	-2.43170000
C	-1.40061700	-3.43733400	-2.30005500
H	-0.78157500	-2.92284600	-0.29243800
C	-2.58888400	-1.71587900	-3.52497200
H	-2.85855600	0.16793300	-2.50674700
C	-2.06072800	-3.01721800	-3.46883900
H	-0.98795200	-4.45225600	-2.23835900
H	-3.10416500	-1.37036300	-4.42999400
H	-2.15345700	-3.69223500	-4.32763400
Pd	-1.69044000	-0.12038300	0.43555700
P	-0.63580200	1.51720500	-0.71820200
C	0.89166200	1.07551000	-1.60341200
C	1.72455800	-1.38480500	0.37423700
C	2.15599600	1.77902300	-1.54868300
C	1.00869200	0.04010700	-2.60894400
Fe	2.38405100	-0.19844000	-1.14183800
C	2.32854600	-2.17655600	-0.65725300
C	2.70251700	-0.44520600	0.84219400
C	3.03657100	1.17749200	-2.50768800
H	2.39321600	2.60314800	-0.87521100
C	2.32984800	0.11229200	-3.16050700
H	0.22927600	-0.67403400	-2.87365800
C	3.68278400	-1.73217800	-0.82939900
C	3.92704400	-0.65269200	0.10088300
H	1.82237500	-2.94211600	-1.24739900
H	2.56030300	0.31631500	1.60976300
H	4.07751600	1.44932000	-2.68197100
H	2.74285100	-0.56052500	-3.91409200
H	4.39629200	-2.10756000	-1.56369100
C	-0.05761500	2.89073800	0.36604000
C	-0.21955100	4.24563300	0.02133000
C	0.56307900	2.55902600	1.58575800
C	0.23146200	5.25070800	0.88860400
H	-0.70697600	4.51386300	-0.92047700
C	1.02169500	3.56374800	2.44574000
H	0.66712100	1.50567800	1.86252000
C	0.85249200	4.91348600	2.10015000
H	0.09479700	6.30261700	0.61554400
H	1.50202800	3.29365100	3.39167100
H	1.20277100	5.70053700	2.77632800
C	-1.54587000	2.40838800	-2.04930300
C	-0.94162900	2.76182800	-3.27141900
C	-2.88730900	2.75812300	-1.81438400

C	-1.67751600	3.45929300	-4.24026200
H	0.10001700	2.49156400	-3.46562900
C	-3.61653300	3.46180500	-2.78016500
H	-3.35792200	2.46725100	-0.87329000
C	-3.01364700	3.81202400	-3.99790800
H	-1.20252300	3.72808700	-5.19000200
H	-4.65869100	3.73068600	-2.57768700
H	-3.58369100	4.35648700	-4.75856200
C	0.09743400	-1.66442000	3.39816300
H	-0.25500500	-2.37721600	2.63758900
H	1.03456300	-1.18768600	3.06601000
H	0.32561300	-2.22236400	4.32697600
C	-2.79573300	-1.18443200	4.55050200
H	-3.60482500	-0.43287800	4.58205700
H	-3.13310000	-2.04671200	3.95145000
H	-2.59860900	-1.51483900	5.58766100
C	-0.51068400	0.83902400	5.02870200
H	0.39904300	1.31447700	4.61861500
H	-1.23877900	1.63489000	5.25973400
H	-0.22723700	0.33580300	5.97210700
H	0.68063100	-1.42684100	0.69113200
P	5.43005700	0.34255300	0.40000500
C	6.29575600	-0.72447700	1.64516800
C	7.60229800	-0.35552200	2.03164800
C	5.69551800	-1.84179100	2.25719200
C	8.29892200	-1.09845200	2.99184500
H	8.07800900	0.51720900	1.56816100
C	6.39159300	-2.57810700	3.22847300
H	4.68213600	-2.13985800	1.96974700
C	7.69373900	-2.21247700	3.59594400
H	9.31573300	-0.80470800	3.27500000
H	5.91249700	-3.44578500	3.69538400
H	8.23592900	-2.79061800	4.35187600
C	6.43258700	-0.05973600	-1.10794900
C	6.64993000	0.96072800	-2.05420200
C	6.99059100	-1.33391300	-1.33934400
C	7.37764400	0.70694100	-3.22644600
H	6.25238100	1.96386400	-1.86045200
C	7.71965200	-1.58741200	-2.50878800
H	6.85633700	-2.12518800	-0.59377800
C	7.90902700	-0.56981400	-3.45751800
H	7.53773800	1.50943300	-3.95479700
H	8.14580700	-2.58215400	-2.67977500
H	8.48116500	-0.76977600	-4.36990600
O	-2.26648100	-1.77414100	1.53812600
C	-3.54557300	-2.11582600	1.30145000
O	-4.29742100	-1.26902700	0.68168300
O	-3.92025800	-3.27795900	1.64933400
Cs	-5.29762700	-3.42273000	-0.81679900

transition state **TS8**

Si	3.62098200	1.30926300	2.11167300
N	3.03167800	-0.51281400	1.73854600
N	3.26803700	-1.37591500	2.60732300
C	3.16592600	-2.75536400	2.30950800
C	3.59551900	-3.27715100	1.07466000
C	2.76396900	-3.62007600	3.34598300
C	3.57949200	-4.66084200	0.86473600
H	3.97107700	-2.58162300	0.31898400
C	2.72548100	-4.99727000	3.11786300
H	2.47087700	-3.18730300	4.30643100
C	3.13242200	-5.52147000	1.87822100
H	3.91855900	-5.06961100	-0.09219400
H	2.38311400	-5.67013700	3.91097500
H	3.11861700	-6.60352700	1.71013000
C	1.80607700	0.40628700	-2.10831600
C	1.14241900	1.65019900	-2.11623400
C	2.11321900	-0.18340000	-3.34880900
C	0.78065200	2.27600200	-3.31805100
H	0.89728800	2.13779000	-1.16638200
C	1.75948700	0.44330900	-4.55706900
H	2.62410200	-1.14966100	-3.39276000
C	1.08205800	1.67046800	-4.54837500
H	0.25382900	3.23931200	-3.29718600
H	2.00815600	-0.03937800	-5.50938500
H	0.79461800	2.15220800	-5.48937800
Pd	2.33286000	-0.32065200	-0.28989700
P	0.79590400	-1.92316900	-0.56542300
C	-0.85814900	-1.59790300	-1.27015300
C	-1.44674100	1.18965800	0.34968900
C	-2.08065700	-2.23133500	-0.80679400
C	-1.18507800	-0.85262500	-2.46692800

Fe	-2.27826300	-0.22127200	-0.86707100
C	-2.07658400	1.80641400	-0.78345700
C	-2.44020300	0.42343800	1.04696000
C	-3.13403300	-1.87280600	-1.70937000
H	-2.18113400	-2.84585000	0.08794600
C	-2.58320100	-1.02386500	-2.72593000
H	-0.49227200	-0.25381900	-3.05511200
C	-3.46079700	1.42911000	-0.78831700
C	-3.69892900	0.56803300	0.34873300
H	-1.57734100	2.40501800	-1.54588600
H	-2.28232000	-0.18751700	1.93633800
H	-4.18091700	-2.15876300	-1.61165700
H	-3.14140600	-0.55186000	-3.53626700
H	-4.20143300	1.70530000	-1.53935000
C	0.28094800	-2.69244000	1.02416900
C	0.04693700	-4.06817700	1.19034800
C	0.01249700	-1.80743400	2.08673600
C	-0.47795800	-4.54641200	2.39970700
H	0.28052400	-4.76686100	0.38220500
C	-0.51226200	-2.28874600	3.29115700
H	0.23584000	-0.74261200	1.96715000
C	-0.76793700	-3.66004200	3.44616600
H	-0.65284300	-5.62009400	2.52515900
H	-0.71333400	-1.59275500	4.11235400
H	-1.17835100	-4.03866400	4.38818500
C	1.35528200	-3.28528800	-1.67361300
C	0.45156900	-4.25147300	-2.16007300
C	2.69905400	-3.33234000	-2.08891400
C	0.89941200	-5.27067500	-3.01116800
H	-0.60624900	-4.19620300	-1.88446100
C	3.14290900	-4.34466100	-2.94973600
H	3.38922400	-2.55525800	-1.74197400
C	2.24534200	-5.32184100	-3.40429400
H	0.19096700	-6.02076100	-3.37854300
H	4.19002500	-4.36518300	-3.26968300
H	2.59062800	-6.11462000	-4.07643700
C	1.87287400	2.14948700	1.82884200
H	1.59538600	2.37097500	0.78040300
H	1.08640800	1.49467400	2.25468300
H	1.82997700	3.09390800	2.40748700
C	5.52551500	0.98421100	2.16757500
H	5.69479500	0.01529900	2.67699000
H	6.01431500	0.95422200	1.17988500
H	6.01575600	1.76409000	2.77992300
C	3.43289500	1.39983200	4.04753500
H	2.39201900	1.24719700	4.39277100
H	4.07458300	0.65676900	4.55486300
H	3.74943900	2.40307800	4.40107200
H	-0.38904200	1.25231600	0.60834100
P	-5.23552600	-0.25932600	0.89601800
C	-5.92011000	1.02636400	2.04419400
C	-7.18959500	0.78493000	2.61187300
C	-5.22442600	2.19573300	2.40691100
C	-7.75846600	1.70108600	3.50422700
H	-7.73736400	-0.12671300	2.34425900
C	-5.79149700	3.10713100	3.31127900
H	-4.23739900	2.39483500	1.97723200
C	-7.05886300	2.86585100	3.85901700
H	-8.74827200	1.50353600	3.93029600
H	-5.23917600	4.01305700	3.58485100
H	-7.50016600	3.57978400	4.56271900
C	-6.32971900	0.03263800	-0.57498200
C	-6.71301100	-1.07438700	-1.35641600
C	-6.79861800	1.31356100	-0.93323100
C	-7.51662400	-0.90489700	-2.49381900
H	-6.38808800	-2.07866800	-1.06059000
C	-7.60395600	1.48358500	-2.06694400
H	-6.53292300	2.17732800	-0.31410300
C	-7.95913200	0.37580400	-2.85333300
H	-7.80491900	-1.77511000	-3.09347700
H	-7.96010300	2.48392200	-2.33687300
H	-8.59020500	0.51066800	-3.73837600
O	3.76708500	1.14563700	0.07559400
C	4.03577100	2.43144900	-0.20472200
O	4.04645500	2.90315800	-1.36177400
O	4.23143100	3.11431400	0.89223100
Cs	3.11934800	5.38062400	-0.40521100

intermediate **L**

Si	3.79953600	1.72106200	1.57608900
N	3.16861500	-0.16456100	1.54319500
N	3.42339100	-0.87763300	2.53267100

C	3.36625700	-2.29093700	2.45802500
C	3.79222300	-2.98139400	1.30778000
C	3.02684400	-3.00015900	3.62614900
C	3.83270900	-4.38041600	1.31361300
H	4.12146700	-2.39606000	0.44485800
C	3.04787100	-4.39659600	3.61580500
H	2.73637000	-2.43635300	4.51702600
C	3.44899400	-5.09099600	2.46067600
H	4.16852500	-4.91864000	0.42159400
H	2.75559600	-4.95120200	4.51369800
H	3.48092700	-6.18568100	2.46273800
C	1.69576400	0.11755600	-2.33370400
C	1.02024600	1.34139800	-2.51149300
C	1.95070200	-0.66858300	-3.47320000
C	0.59087700	1.75690000	-3.78016700
H	0.82702200	1.98386000	-1.64625300
C	1.52685300	-0.25355200	-4.74841300
H	2.47082200	-1.62656600	-3.38232900
C	0.83430600	0.95484000	-4.90621800
H	0.06514300	2.71354000	-3.89305200
H	1.73322300	-0.88650700	-5.61933400
H	0.49511300	1.27414700	-5.89772100
Pd	2.34912500	-0.28655500	-0.45628100
P	0.89096000	-1.97617600	-0.40444100
C	-0.80816700	-1.81701400	-1.06374500
C	-1.44974700	1.16552800	0.14097700
C	-1.97823600	-2.41125900	-0.43952000
C	-1.22695600	-1.28541600	-2.34336900
Fe	-2.26858500	-0.44497100	-0.80707500
C	-2.13419900	1.57478900	-1.05307300
C	-2.39456400	0.49143200	0.98560700
C	-3.08892900	-2.24120800	-1.32793700
H	-2.00892300	-2.87855400	0.54463500
C	-2.62682000	-1.54712000	-2.49486000
H	-0.59246800	-0.76397100	-3.05720700
C	-3.50411100	1.16093300	-0.94889900
C	-3.67805200	0.48574100	0.31808900
H	-1.67872100	2.05711800	-1.91843200
H	-2.18839800	0.03534500	1.95448500
H	-4.11545100	-2.54691400	-1.12790800
H	-3.24397200	-1.23182600	-3.33792400
H	-4.27617500	1.29101800	-1.70773400
C	0.46718400	-2.52750400	1.29771200
C	0.30154700	-3.87411900	1.66357100
C	0.17908000	-1.51143200	2.22953500
C	-0.17592400	-4.19638000	2.94210100
H	0.55011200	-4.67023600	0.95639900
C	-0.29823600	-1.83781000	3.50358800
H	0.34726000	-0.46598800	1.95275600
C	-0.48648300	-3.18250800	3.85890600
H	-0.29762900	-5.24755500	3.22364800
H	-0.51621100	-1.04095200	4.22248900
H	-0.86031000	-3.43964000	4.85557400
C	1.46150800	-3.46472500	-1.32787200
C	0.57522700	-4.51493800	-1.64145400
C	2.79402300	-3.53442400	-1.77498800
C	1.03096200	-5.63298400	-2.35289100
H	-0.47524900	-4.45014100	-1.34093500
C	3.24527200	-4.64746500	-2.49602800
H	3.46805400	-2.69610400	-1.56719100
C	2.36628200	-5.70356300	-2.77769000
H	0.33643600	-6.44693100	-2.58653900
H	4.28315200	-4.68536600	-2.84320200
H	2.71735000	-6.57491100	-3.34070800
C	1.88949400	2.29058400	1.45064200
H	1.44729500	2.27857600	0.43258700
H	1.24309300	1.65712700	2.09073800
H	1.78683300	3.32064500	1.85737000
C	5.62096700	1.02824800	1.42520900
H	5.87958600	0.47281400	2.34733400
H	5.77619100	0.35385200	0.56381500
H	6.33057800	1.87196700	1.32618100
C	3.99123500	2.28530700	3.41282700
H	3.97552000	1.37340800	4.04193000
H	4.97051200	2.77195400	3.58260900
H	3.19685900	2.96071200	3.78316700
H	-0.38743100	1.29923400	0.34938500
P	-5.16944300	-0.28535200	1.04361300
C	-5.86127100	1.15040000	1.99259900
C	-7.10524300	0.97173200	2.63500600
C	-5.19177900	2.38071000	2.13817100
C	-7.67533400	2.00549700	3.38713700

H	-7.63234900	0.01507700	2.53660500
C	-5.75957400	3.41180400	2.90262700
H	-4.22465100	2.53237800	1.64811200
C	-7.00196100	3.23006600	3.52533200
H	-8.64546800	1.85424400	3.87316800
H	-5.22795300	4.36425200	3.00729200
H	-7.44421200	4.03701200	4.11939300
C	-6.31492900	-0.26683200	-0.41706700
C	-6.67817100	-1.49630500	-0.99978000
C	-6.84050800	0.92453500	-0.95879000
C	-7.51776800	-1.53689600	-2.12295100
H	-6.30858000	-2.42942300	-0.55898700
C	-7.68214100	0.88494600	-2.07814100
H	-6.59029900	1.88450900	-0.49419500
C	-8.01705700	-0.34512100	-2.66669600
H	-7.78955100	-2.50065200	-2.56708800
H	-8.08224500	1.81723600	-2.49180200
H	-8.67625400	-0.37389600	-3.54096500
O	3.68511200	1.34435900	-0.48742600
C	3.85439600	2.63230300	-0.71510900
O	3.68228700	3.23987900	-1.78512900
O	4.18379500	3.24405700	0.43137500
Cs	2.59936500	5.47684200	-0.38577200

transition state **TS9**

Si	-3.80983100	-1.62664600	2.09666300
N	-2.91446800	0.51872000	1.78892900
N	-3.11653800	1.39152700	2.64127000
C	-3.09208100	2.78278800	2.32862100
C	-3.59363900	3.26582000	1.10643800
C	-2.68133300	3.67838000	3.33237600
C	-3.63614500	4.64557700	0.87194900
H	-3.96672900	2.54190200	0.37564600
C	-2.70498900	5.05267200	3.08198700
H	-2.33039100	3.27395700	4.28615000
C	-3.17955800	5.54023400	1.85126200
H	-4.02890500	5.02493300	-0.07698000
H	-2.35380300	5.75148700	3.84866600
H	-3.21098500	6.61900300	1.66466800
C	-1.80805900	-0.45575500	-2.06484800
C	-1.14729900	-1.70441400	-2.04439900
C	-2.14086800	0.07724700	-3.32554900
C	-0.82174000	-2.38721000	-3.22716400
H	-0.86264600	-2.14820600	-1.08252600
C	-1.82346700	-0.60209500	-4.51519300
H	-2.64702800	1.04522400	-3.39775500
C	-1.15513400	-1.83416600	-4.47388800
H	-0.29009300	-3.34783000	-3.17966800
H	-2.09364500	-0.15886100	-5.48097500
H	-0.89573700	-2.35687000	-5.40114300
Pd	-2.32083600	0.34088200	-0.24410100
P	-0.77773500	1.91930900	-0.53281800
C	0.87245800	1.56689000	-1.24045600
C	1.49196800	-1.16894700	0.46217700
C	2.10010300	2.21136800	-0.80851300
C	1.18586200	0.79112300	-2.42103200
Fe	2.29941700	0.19922000	-0.81867700
C	2.08641500	-1.82328400	-0.66951500
C	2.50900000	-0.38919500	1.10874900
C	3.14342200	1.82902900	-1.71287100
H	2.21021100	2.85015300	0.06792000
C	2.58111300	0.95407100	-2.70081500
H	0.48455400	0.17910000	-2.98502100
C	3.47257800	-1.45667500	-0.72286300
C	3.74726000	-0.56440600	0.38141600
H	1.56345100	-2.44077400	-1.40052100
H	2.37947600	0.24930200	1.98327000
H	4.19112700	2.11794100	-1.63430100
H	3.12997400	0.46153300	-3.50533400
H	4.19099000	-1.76060500	-1.48468100
C	-0.23759000	2.76054200	1.01056500
C	-0.04283300	4.14790400	1.12120900
C	0.08713400	1.92307700	2.09585500
C	0.49931700	4.68570900	2.29742200
H	-0.32104400	4.80892600	0.29579900
C	0.63182500	2.46354100	3.26563000
H	-0.11004400	0.84898600	2.02175000
C	0.84752800	3.84704800	3.36513000
H	0.64221000	5.76824100	2.38022400
H	0.87926300	1.80506800	4.10507100
H	1.27203400	4.27176600	4.28093100
C	-1.34045100	3.24018600	-1.68982400

C	-0.43701200	4.18175000	-2.22297000
C	-2.68888800	3.28045600	-2.08964700
C	-0.88869300	5.16944700	-3.10848400
H	0.62314300	4.13283900	-1.95519300
C	-3.13702800	4.26213500	-2.98320300
H	-3.37768500	2.52042500	-1.70420400
C	-2.23903600	5.21430100	-3.48709700
H	-0.18017900	5.90022000	-3.51294200
H	-4.18814500	4.27795100	-3.29019300
H	-2.58762500	5.98276800	-4.18535000
C	-1.93487300	-2.06467300	1.82634800
H	-1.54243100	-1.74371000	0.84277500
H	-1.31776700	-1.57507200	2.60251300
H	-1.77442500	-3.15921600	1.93609900
C	-5.41585100	-0.58533500	2.26327300
H	-5.39604400	-0.02285200	3.21473400
H	-5.58066000	0.12652700	1.43845200
H	-6.26914800	-1.29005200	2.29863000
C	-3.93365300	-2.49412000	3.81905300
H	-3.54531600	-1.80577300	4.59682600
H	-4.97904400	-2.72856800	4.09378200
H	-3.33725900	-3.42452200	3.88904800
H	0.44087500	-1.21507400	0.74961300
P	5.30532500	0.26374600	0.86292300
C	6.00708000	-0.99339200	2.03238200
C	7.28809200	-0.74234100	2.56908300
C	5.31431300	-2.14986600	2.43944700
C	7.87063700	-1.63682100	3.47457800
H	7.83373600	0.15977700	2.26708700
C	5.89523000	-3.03930700	3.35676500
H	4.31846600	-2.35588600	2.03407200
C	7.17373500	-2.78881400	3.87362300
H	8.86907300	-1.43205000	3.87641400
H	5.34502400	-3.93538400	3.66494800
H	7.62582100	-3.48552300	4.58767800
C	6.35666100	-0.08509500	-0.62691100
C	6.72342900	0.99216800	-1.45638600
C	6.80794900	-1.38046000	-0.95449900
C	7.49296700	0.77885600	-2.60982100
H	6.41273100	2.00805700	-1.18619800
C	7.57971600	-1.59414100	-2.10409100
H	6.55486300	-2.22105400	-0.29925800
C	7.91820100	-0.51610900	-2.93771900
H	7.76843100	1.62630100	-3.24693500
H	7.92258000	-2.60536200	-2.34960200
H	8.52293700	-0.68501100	-3.83522300
O	-3.91844000	-1.04996400	-0.04156200
C	-4.25516500	-2.28812600	-0.26219900
O	-4.39292900	-2.87398400	-1.34712300
O	-4.40605900	-2.94037200	0.92383000
Cs	-3.08603600	-5.18781400	-0.40989500

Ph–Br (**1b**)

Br	0.000000	-0.000000	-0.011539
C	-0.000000	-0.000000	1.918645
C	1.224220	0.000000	2.599359
C	1.213842	0.000000	4.002007
C	-0.000000	0.000000	4.704590
C	-1.213842	0.000000	4.002007
C	-1.224220	-0.000000	2.599359
H	2.165332	0.000000	2.042954
H	2.165029	0.000000	4.544698
H	-0.000000	0.000000	5.799265
H	-2.165029	0.000000	4.544698
H	-2.165332	-0.000000	2.042954

Ph–Br (**1b**) with SMD(toluene)

Br	0.00000000	0.00000000	1.82316400
C	0.00000000	0.00000000	-0.11258900
C	0.00000000	1.22512000	-0.79105300
C	0.00000000	1.21409800	-2.19393900
C	0.00000000	0.00000000	-2.89609300
C	0.00000000	-1.21409800	-2.19393900
C	0.00000000	-1.22512000	-0.79105300
H	0.00000000	2.16802800	-0.23750400
H	0.00000000	2.16546700	-2.73640500
H	0.00000000	0.00000000	-3.99090900
H	0.00000000	-2.16546700	-2.73640500
H	0.00000000	-2.16802800	-0.23750400

PhN_2–$SiMe_3$ (**2b**)

C	-4.146390	-0.121496	0.615593

C	-3.179335	0.042048	1.626271
C	-1.821737	0.066946	1.301830
C	-1.429051	-0.072890	-0.044299
C	-2.392126	-0.236254	-1.055021
C	-3.753031	-0.260540	-0.724041
H	-5.209902	-0.140113	0.877315
H	-3.496448	0.149838	2.669573
H	-1.035795	0.191652	2.053275
H	-2.042387	-0.341208	-2.087116
H	-4.507574	-0.387539	-1.507461
N	-0.061580	-0.060771	-0.481396
N	0.796256	0.086438	0.433399
Si	2.530317	0.104777	-0.095142
C	3.338810	-1.309943	0.855867
H	3.147509	-1.210996	1.939346
H	2.942613	-2.287893	0.527448
H	4.433351	-1.316016	0.699068
C	2.698139	-0.105925	-1.960048
H	3.757648	-0.094098	-2.275603
H	2.249422	-1.061766	-2.284315
H	2.168097	0.704646	-2.491238
C	3.196357	1.768293	0.495284
H	4.287401	1.837750	0.329625
H	2.716225	2.603869	-0.045550
H	3.003327	1.904865	1.574358

2c

C	-3.32863400	1.16744400	-0.00002000
C	-3.81003100	-0.14631500	-0.00002600
C	-2.95461100	-1.26024400	0.00000100
C	-1.57704000	-1.04788000	0.00002100
C	-1.06769900	0.26747100	0.00002100
C	-1.94417100	1.36749000	0.00001600
N	0.32975700	0.57929500	0.00005600
N	1.10616900	-0.41735900	-0.00015000
Si	2.88058300	-0.04761900	-0.00002900
C	3.54869600	-0.88887900	-1.55084600
C	3.20957200	1.80765700	-0.00068400
C	3.54822100	-0.88765300	1.55166400
F	-5.14147500	-0.35317700	-0.00004400
H	-3.38789700	-2.26507500	0.00001500
H	-0.85812300	-1.87318200	0.00003800
H	-1.51076800	2.37253500	0.00003000
H	-4.03662900	2.00082000	-0.00003600
H	4.29319300	2.02680400	-0.00022700
H	2.75975900	2.28141300	-0.89140800
H	2.75882600	2.28216000	0.88916700
H	4.65159300	-0.82620900	1.58962700
H	3.14936000	-0.41103500	2.46543100
H	3.26290600	-1.95468400	1.57121400
H	3.26340100	-1.95593000	-1.56962300
H	3.15010800	-0.41300700	-2.46512200
H	4.65207700	-0.82745000	-1.58852600

2d

C	-2.90538400	1.03354200	0.00000100
C	-3.37900600	-0.29456100	0.00000300
C	-2.46271900	-1.37620900	0.00000400
C	-1.09682400	-1.13285100	0.00000400
C	-0.61445400	0.19669600	0.00000100
C	-1.52496300	1.26567600	0.00000000
N	0.76716000	0.54484900	0.00000200
N	1.57595500	-0.42988000	0.00000500
Si	3.33286100	-0.00517300	-0.00000100
C	4.03639000	-0.82189800	-1.54956500
C	3.61013700	1.85992100	-0.00002100
C	4.03640900	-0.82186000	1.54957600
O	-4.69396800	-0.64027100	0.00000400
H	-2.86805400	-2.39293700	0.00000500
H	-0.35970900	-1.94191600	0.00000400
H	-1.12163900	2.28354600	-0.00000200
H	-3.59788400	1.87875800	0.00000100
H	4.68692200	2.11093200	-0.00007200
H	3.14552600	2.32068500	-0.89005400
H	3.14561300	2.32069900	0.89005100
H	3.78462900	-1.89738300	1.57105600
H	5.13753300	-0.72586800	1.58359000
H	3.62585000	-0.35805500	2.46489100
H	3.78459900	-1.89742000	-1.57101400
H	3.62582100	-0.35811500	-2.46488700
H	5.13751400	-0.72591800	-1.58359900
C	-5.67010900	0.39522100	-0.00000500

H	-6.64689300	-0.10989500	-0.00000700
H	-5.58340900	1.03031700	0.90212900
H	-5.58340200	1.03030800	-0.90214600

2e

C	-1.87582100	1.09283200	0.03796400
C	-2.71535700	-0.04033800	0.03459400
C	-2.16821000	-1.33294700	0.02513000
C	-0.77817700	-1.49284900	0.01496400
C	0.05797400	-0.36468500	0.01467000
C	-0.49173500	0.93236300	0.02734900
N	1.47139600	-0.63144000	0.00325900
N	2.20699100	0.39307700	0.01027500
Si	3.99911600	0.09561700	-0.00657600
C	4.63430800	0.94600700	1.55204000
C	4.39907500	-1.74434500	-0.03231100
C	4.61109800	0.98428900	-1.55307000
H	-2.83056900	-2.20284300	0.03156600
H	-0.31205200	-2.48293700	0.00949500
H	0.19829000	1.78155300	0.03165300
H	-2.31981000	2.09299700	0.05602300
H	3.97482700	-2.24690200	0.85510900
H	3.96189700	-2.22475400	-0.92570100
H	5.49046900	-1.92016600	-0.04242000
H	4.22287400	0.50346300	-2.46910400
H	4.28156800	2.03857800	-1.55430500
H	5.71556600	0.96920900	-1.60274600
H	5.73941000	0.93050900	1.58449400
H	4.30446600	1.99970100	1.58446700
H	4.26027700	0.44230100	2.46166100
C	-4.21226700	0.14956700	-0.00615700
F	-4.64959600	0.38554800	-1.27037800
F	-4.60480000	1.20714600	0.74611700
F	-4.87770200	-0.94049200	0.44493900

2f

C	2.33209400	0.93130000	-0.00001200
C	3.69280500	0.60574400	0.00007300
C	4.06944900	-0.74296800	0.00006900
C	3.09355400	-1.75881500	-0.00002700
C	1.74048800	-1.42060900	-0.00012100
C	1.33860000	-0.06923100	-0.00009800
H	5.13304500	-1.00346100	0.00013500
N	-0.02548300	0.36238400	-0.00012200
N	-0.87688800	-0.57143400	-0.00022500
Si	-2.61570300	-0.05378600	-0.00000100
C	-3.34826100	-0.83767400	1.55190300
C	-2.78518100	1.82166100	-0.00104500
C	-3.34919300	-0.83953100	-1.55051300
H	3.39967000	-2.81010000	-0.00004000
H	0.94105700	-2.16870900	-0.00017600
F	1.97499200	2.22467900	-0.00002400
H	4.43153500	1.41225100	0.00014200
H	-2.91229000	-0.39873600	-2.46483900
H	-3.15283700	-1.92649600	-1.56799400
H	-4.44374200	-0.68728700	-1.58884800
H	-4.44279000	-0.68539300	1.59070000
H	-3.15188500	-1.92461500	1.57058700
H	-2.91081300	-0.39575700	2.46542600
H	-3.84603800	2.13290800	-0.00052100
H	-2.29415600	2.25572500	0.88802500
H	-2.29538200	2.25464300	-0.89132200

2g

C	1.26927000	-0.30395500	0.00003700
C	2.33102400	0.64737700	-0.00006800
C	3.66572600	0.16989900	-0.00014600
C	3.94336600	-1.19247600	-0.00007300
C	2.89288100	-2.13779900	0.00008300
C	1.58094800	-1.68674100	0.00013300
O	2.24406700	1.99686200	-0.00014100
N	-0.07610100	0.15698600	0.00005300
N	-0.97019800	-0.74123800	-0.00010400
Si	-2.68498500	-0.17949200	-0.00003700
C	-2.82688700	1.70338400	-0.00047600
C	-3.45208100	-0.93355600	-1.55041600
C	-3.45173800	-0.93279000	1.55089100
H	4.98595000	-1.52844100	-0.00011800
H	3.11408200	-3.21010100	0.00017100
H	0.72245200	-2.36596300	0.00023300
H	4.46455500	0.91719300	-0.00025000
H	-2.33845600	2.13485000	0.89169200

H	-2.33868800	2.13440800	-0.89298600
H	-3.88473100	2.02528400	-0.00041900
H	-3.28799200	-2.02576800	-1.57327300
H	-4.54179100	-0.74884700	-1.58393500
H	-3.00540500	-0.50379100	-2.46540600
H	-3.00485400	-0.50256900	2.46556500
H	-4.54144000	-0.74806000	1.58456100
H	-3.28764900	-2.02499100	1.57425600
C	1.01043000	2.72026600	0.00020700
H	1.31040000	3.78016500	0.00039800
H	0.40850300	2.48656800	-0.89242200
H	0.40877100	2.48616400	0.89291000

2h

C	2.01744800	0.01082700	0.00000100
C	3.29390400	0.59175600	-0.00002200
C	3.43196700	1.98671200	-0.00002000
C	2.29463800	2.81253000	0.00000200
C	1.02000600	2.24235100	0.00002000
C	0.87618000	0.84401600	0.00002000
H	4.43275400	2.43062600	-0.00003500
N	-0.39625000	0.18187900	0.00005100
N	-1.40603000	0.93676300	-0.00003500
Si	-2.99214800	0.03878900	-0.00000200
C	-2.69161000	-1.81819700	0.00022600
C	-3.88165500	0.63525700	1.55247100
C	-3.88150100	0.63489100	-1.55270400
H	2.40990000	3.90186000	0.00000600
H	0.10270000	2.83941900	0.00003500
H	4.17628500	-0.05276700	-0.00004100
H	-4.90862500	0.22785600	-1.59680500
H	-3.94848600	1.73743200	-1.56793900
H	-3.34818500	0.31122000	-2.46470500
H	-2.10290900	-2.11132500	0.88755500
H	-3.63878600	-2.38790600	0.00021700
H	-2.10277600	-2.11151400	-0.88695300
H	-4.90878300	0.22823000	1.59656800
H	-3.34842900	0.31180500	2.46460300
H	-3.94864500	1.73780100	1.56743600
C	1.86817500	-1.49948600	0.00000200
F	1.21014500	-1.95090400	1.09220900
F	1.21005000	-1.95089500	-1.09215400
F	3.08588300	-2.10711400	-0.00005400

$CsCO_3^-$

C	-2.02644200	0.01994100	0.00006300
O	-1.32715200	-1.11806900	0.00028400
O	-1.34198000	1.14158400	0.00026800
O	-3.28627700	-0.02750000	-0.00034000
Cs	1.08730800	-0.00159600	-0.00003800

$CsCO_3$–$SiMe_3$

C	0.36419100	0.72290700	-0.00009300
O	-0.49159900	1.64468500	-0.00001700
O	0.13123500	-0.53362800	-0.00031800
O	1.69054200	1.10169200	0.00010700
Cs	-2.64716800	-0.12597500	0.00003000
Si	2.89963800	-0.11550400	0.00002300
C	2.82455300	-1.16433800	-1.56619100
H	3.69057500	-1.85057300	-1.62517400
H	1.89592700	-1.75880800	-1.58296900
H	2.84248300	-0.51997200	-2.46465900
C	2.82415700	-1.16499600	1.56578100
H	3.69020400	-1.85120300	1.62472600
H	2.84179000	-0.52100700	2.46452500
H	1.89556100	-1.75952800	1.58204100
C	4.49114900	0.89972800	0.00045400
H	5.38320000	0.24540100	0.00043600
H	4.54704000	1.54998100	-0.89164900
H	4.54679400	1.54959500	0.89285300

Br^-

Br	0.00000000	0.00000000	0.00000000

(*E*)-PhN_2–Ph ((*E*)-**3bb**)

C	-4.530151	-0.029317	-0.411440
C	-3.840851	-0.030452	-1.633497
C	-2.441209	-0.030480	-1.644119
C	-1.724886	-0.029375	-0.430407
C	-2.420568	-0.028230	0.800247
C	-3.816019	-0.028208	0.802196
H	-5.625338	-0.029289	-0.400305

H	-4.396037	-0.031314	-2.577317
H	-1.870775	-0.031344	-2.578031
H	-1.837682	-0.027390	1.725160
H	-4.360154	-0.027321	1.753101
N	-0.314506	-0.029497	-0.550111
N	0.314508	-0.028571	0.550003
C	1.724889	-0.028682	0.430298
C	2.441212	-0.027739	1.644011
C	2.420570	-0.029645	-0.800356
C	3.840854	-0.027759	1.633388
H	1.870779	-0.027002	2.577923
C	3.816021	-0.029656	-0.802305
H	1.837684	-0.030366	-1.725268
C	4.530154	-0.028716	0.411331
H	4.396041	-0.027027	2.577208
H	4.360155	-0.030398	-1.753211
H	5.625340	-0.028731	0.400195

(*Z*)-PhN_2–Ph ((*Z*)-**3bb**)

C	-2.10040000	-1.26577100	1.18311900
C	-1.20421000	-0.19891000	1.06349500
C	-1.41801100	0.78306800	0.07243400
C	-2.54734800	0.70220800	-0.76581600
C	-3.41494100	-0.39051900	-0.66384100
C	-3.19625000	-1.37669100	0.31153900
H	-1.94026600	-2.02030200	1.96064500
H	-0.34823200	-0.11257000	1.73856600
H	-2.71688200	1.50006600	-1.49539600
H	-4.27691100	-0.46420800	-1.33529800
H	-3.88747000	-2.22059800	0.40439800
N	-0.62719400	1.97103300	-0.01966000
N	0.62719000	1.97103300	0.01966200
C	1.41800600	0.78306800	-0.07243500
C	2.54733700	0.70220600	0.76582500
C	1.20421600	-0.19890600	-1.06350200
C	3.41493300	-0.39051900	0.66385000
H	2.71686000	1.50005800	1.49541400
C	2.10041300	-1.26576100	-1.18312900
H	0.34824600	-0.11256200	-1.73858300
C	3.19625600	-1.37668200	-0.31154100
H	4.27689600	-0.46421200	1.33531700
H	1.94029000	-2.02028600	-1.96066200
H	3.88747800	-2.22058700	-0.40440000

(*E*)-**3bc**

C	-4.99811700	0.15705700	-0.00005500
C	-4.45920900	-1.13837100	0.00018700
C	-3.07086400	-1.31572000	0.00033400
C	-2.21473000	-0.19601500	0.00015600
C	-2.75911500	1.10875800	-0.00007400
C	-4.14438100	1.27687800	-0.00016400
N	-0.82930700	-0.48281300	0.00034700
N	-0.07240900	0.53453500	-0.00045000
C	1.31023800	0.24755300	-0.00024400
C	1.85430400	-1.05865600	-0.00050700
C	3.23593000	-1.23932000	-0.00032700
C	4.06917000	-0.10909400	0.00010500
C	3.55839600	1.19332600	0.00029900
C	2.17107300	1.36454800	0.00006600
F	5.40440600	-0.28804600	0.00026700
H	3.68835900	-2.23547800	-0.00056900
H	1.16451500	-1.90673600	-0.00083800
H	1.72166400	2.36201900	0.00017000
H	4.24690700	2.04276500	0.00059600
H	-2.61616300	-2.31112200	0.00057100
H	-2.07093600	1.95819500	-0.00014800
H	-5.12277000	-2.00939500	0.00030000
H	-4.57124400	2.28586000	-0.00030400
H	-6.08414700	0.29858300	-0.00012000

(*E*)-**3bd**

C	5.46690200	0.25636700	0.00002400
C	4.96751600	-1.05489500	-0.00012800
C	3.58531700	-1.27546400	-0.00014000
C	2.69310800	-0.18406300	-0.00001300
C	3.19828000	1.13656000	0.00014100
C	4.57786200	1.34812000	0.00016000
N	1.31738600	-0.51374400	-0.00002300
N	0.53057700	0.48345000	-0.00006100
C	-0.83921300	0.16329300	-0.00002900
C	-1.35826600	-1.15694000	0.00011500
C	-2.72920200	-1.36668600	0.00014400

C	-3.62184000	-0.26576100	0.00002900
C	-3.11652500	1.05046000	-0.00011300
C	-1.73229300	1.25153200	-0.00013800
O	-4.94380400	-0.58161500	0.00007100
H	-3.15586600	-2.37444500	0.00025800
H	-0.65032700	-1.99004500	0.00020200
H	-1.31080500	2.26149600	-0.00024500
H	-3.78827400	1.91217400	-0.00020000
H	3.16212500	-2.28476000	-0.00024800
H	2.48358700	1.96391200	0.00024300
H	5.65758900	-1.90527800	-0.00023100
H	4.97244900	2.37035700	0.00028600
H	6.54806400	0.43164000	0.00004200
C	-5.89650700	0.47582800	-0.00003500
H	-5.79517600	1.10861500	0.90211300
H	-5.79518600	1.10842600	-0.90231700
H	-6.88432400	-0.00721600	0.00002100

(*E*)-**3be**

C	6.16492900	-0.06706000	0.01804500
C	5.58846700	1.21209500	0.00624500
C	4.19566900	1.34774600	-0.00333600
C	3.37465900	0.20176200	-0.00120200
C	3.95669100	-1.08684100	0.01070900
C	5.34601000	-1.21291500	0.02025100
N	1.98233800	0.44678500	-0.01134100
N	1.25472800	-0.59088500	-0.01044700
C	-0.13784500	-0.33447400	-0.01854600
C	-0.96962000	-1.47096200	-0.02514200
C	-2.35999900	-1.32643700	-0.03293200
C	-2.92406900	-0.04101700	-0.03394700
C	-2.09566300	1.10005300	-0.03117400
C	-0.71023400	0.95806900	-0.02295000
H	-3.01071800	-2.20497400	-0.04416600
H	-0.49712700	-2.45754200	-0.02642800
H	-0.04191000	1.82279300	-0.02285100
H	-2.54929900	2.09581200	-0.04271500
H	3.71091900	2.32872900	-0.01258800
H	3.29427300	-1.95643700	0.01216400
H	6.22604400	2.10214400	0.00457500
H	5.80365500	-2.20810500	0.02956500
H	7.25465500	-0.17591000	0.02563100
C	-4.42231900	0.13089100	0.01015400
F	-5.07489400	-0.96062800	-0.45629600
F	-4.82783300	1.19456500	-0.72668100
F	-4.86282900	0.34347600	1.27767400

(*E*)-**3bf**

C	2.53557200	0.96315000	-0.00008600
C	1.58529900	-0.08395400	0.00003100
C	2.05919500	-1.41668700	0.00018700
C	3.42763800	-1.68383600	0.00021300
C	4.35355600	-0.62239000	0.00007900
C	3.90935800	0.70571700	-0.00007200
N	0.22845900	0.29179200	0.00008600
N	-0.58979400	-0.67775000	-0.00025300
C	-1.95213800	-0.29707400	-0.00010700
C	-2.88422600	-1.35432100	-0.00027300
C	-4.25655400	-1.07915400	-0.00014200
C	-4.70211900	0.25130200	0.00013800
C	-3.77170700	1.30855100	0.00028400
C	-2.40187900	1.04357700	0.00016300
H	5.42845400	-0.83049000	0.00010600
H	3.78410200	-2.71897400	0.00035700
H	1.30950500	-2.21262300	0.00029300
F	2.11400300	2.23678000	-0.00023500
H	4.60603000	1.54871100	-0.00017900
H	-2.50077200	-2.37928400	-0.00049600
H	-1.65362600	1.84085300	0.00026600
H	-4.98027000	-1.90087200	-0.00026200
H	-4.12659300	2.34499500	0.00048300
H	-5.77548500	0.46908600	0.00023200

(*E*)-**3bg**

C	-2.47075200	0.90811300	0.00032100
C	-2.04032000	-0.43948000	0.00003300
C	-2.99726400	-1.47572700	-0.00025000
C	-4.36400000	-1.17529700	-0.00028100
C	-4.78728200	0.16245300	-0.00000600
C	-3.83599700	1.19974400	0.00030200
N	-0.69043500	-0.85531900	0.00005600
N	0.16129600	0.08907900	-0.00003800

C	1.50681600	-0.31241600	0.00001300
C	2.52579600	0.68916800	-0.00008300
C	3.88219500	0.28076800	-0.00005500
C	4.23013500	-1.06511200	0.00006300
C	3.22745200	-2.06094500	0.00015900
C	1.89376900	-1.68051900	0.00013200
O	2.37282100	2.03301500	-0.00020600
H	5.28810100	-1.34825600	0.00007900
H	3.50144600	-3.12084200	0.00024500
H	1.08135800	-2.41194000	0.00019500
H	4.64062200	1.06891000	-0.00013100
H	-2.63436000	-2.50809700	-0.00045500
H	-1.71842700	1.70020200	0.00056400
H	-5.10172700	-1.98460700	-0.00051500
H	-4.16957100	2.24337500	0.00053500
H	-5.85626500	0.40041500	-0.00001600
C	1.11027400	2.70210100	-0.00012600
H	0.52018600	2.44479900	-0.89501400
H	0.52033300	2.44484600	0.89487400
H	1.36477500	3.77366200	-0.00017000

(*E*)-**3bh**

C	2.21662000	-0.06957500	-0.00000200
C	1.12491700	0.83441500	-0.00000200
C	1.37002700	2.22394100	-0.00000500
C	2.67978600	2.70526400	-0.00000900
C	3.76117000	1.80754400	-0.00001100
C	3.52784900	0.42573300	-0.00000700
N	-0.16585000	0.26108400	0.00000200
N	-1.12340000	1.09011300	-0.00000100
C	-2.40853500	0.50058200	-0.00000100
C	-3.49486000	1.39876400	0.00002000
C	-4.80634100	0.91053700	0.00002000
C	-5.03513600	-0.47385300	-0.00000300
C	-3.94996400	-1.37183000	-0.00002600
C	-2.63899600	-0.89514000	-0.00002500
H	4.78914600	2.18376900	-0.00001400
H	2.86506400	3.78475100	-0.00001100
H	0.50466100	2.89172600	-0.00000400
H	4.36402100	-0.27759200	-0.00000700
H	-3.27767600	2.47127900	0.00003700
H	-1.77546000	-1.56568700	-0.00004400
H	-5.65099800	1.60718100	0.00003800
H	-4.13721400	-2.45105300	-0.00004500
H	-6.06062700	-0.85839800	-0.00000300
C	1.97358700	-1.56645900	0.00000800
F	3.14874000	-2.25167400	0.00001300
F	1.28580100	-1.97548200	1.09134900
F	1.28580300	-1.97549800	-1.09133000

Me_3Si–Br

Si	1.001228	0.000187	0.000123
C	1.547283	-0.679802	1.666677
H	2.652293	-0.702478	1.722951
H	1.171953	-0.051169	2.492976
H	1.172437	-1.707350	1.816624
C	1.547527	1.783272	-0.244827
H	2.652547	1.843262	-0.251978
H	1.173466	2.183624	-1.203286
H	1.171629	2.427689	0.568969
C	1.547142	-1.103576	-1.421792
H	1.172677	-2.133682	-1.289475
H	1.171465	-0.720698	-2.386736
H	2.652156	-1.140258	-1.470209
Br	-1.303105	0.000490	0.000379

N_2

N	0.000000	0.000000	0.154541
N	0.000000	0.000000	1.263459

Ph–Ph (**4bb**)

C	-2.000387	-2.114529	-0.000170
C	-0.589179	-2.100921	-0.054618
C	0.120253	-0.893782	-0.055922
C	-0.564948	0.329005	-0.002675
C	-1.966576	0.332224	0.051823
C	-2.675555	-0.875182	0.052995
C	-2.753149	-3.395949	0.001144
C	-2.299016	-4.506160	-0.744410
C	-3.008011	-5.713556	-0.743293
C	-4.188588	-5.839483	0.003650
C	-4.652737	-4.745842	0.749342

C	-3.943323	-3.538692	0.747984
H	-0.042897	-3.049837	-0.069887
H	1.215035	-0.908425	-0.089436
H	-0.010257	1.273251	-0.003643
H	-2.512375	1.281397	0.084378
H	-3.770363	-0.860017	0.069221
H	-1.392992	-4.411032	-1.352003
H	-2.641228	-6.558143	-1.336613
H	-4.743280	-6.783729	0.004618
H	-5.568504	-4.835784	1.343620
H	-4.300821	-2.700070	1.354619

4bc

C	-1.18057700	0.00000000	0.00000000
C	-1.90729500	-1.15390300	-0.36771800
C	-3.30742700	-1.15438100	-0.36678600
C	-4.01474800	0.00000000	0.00000000
C	-3.30742700	1.15438100	0.36678600
C	-1.90729400	1.15390300	0.36771800
C	0.30495600	0.00000000	0.00000000
C	1.03341000	-1.15378000	0.36585700
C	2.43262700	-1.16295100	0.36848300
C	3.11509500	0.00000000	0.00000000
C	2.43262700	1.16295100	-0.36848300
C	1.03341000	1.15378000	-0.36585700
H	-1.36667200	-2.05250100	-0.68309500
H	-3.84912500	-2.05880600	-0.66404200
H	-5.10980400	0.00000000	0.00000000
H	-3.84912500	2.05880600	0.66404100
H	-1.36667200	2.05250100	0.68309500
H	0.49544300	-2.05417000	0.67903800
H	2.99958800	-2.05115000	0.66147200
H	2.99958800	2.05115000	-0.66147200
H	0.49544200	2.05417000	-0.67903800
F	4.46524300	0.00000000	0.00000000

4bd

C	-1.63433500	0.00131700	-0.00465300
C	-2.42358300	1.10574900	0.38765300
C	-3.82117900	1.02428600	0.40771600
C	-4.46644400	-0.16539300	0.03904500
C	-3.69773000	-1.27174300	-0.35195900
C	-2.30037100	-1.18844500	-0.37547800
C	-0.15283400	0.08842700	-0.02571600
C	0.50833600	1.29450900	-0.36004500
C	1.89870900	1.38190500	-0.38137200
C	2.68574900	0.25585400	-0.06403600
C	2.05160200	-0.95505500	0.27283200
C	0.65196500	-1.02479900	0.28759700
H	-1.93223700	2.03144100	0.70525500
H	-4.40975200	1.89265000	0.72337600
H	-5.55955100	-0.22968600	0.05574400
H	-4.18990200	-2.20345500	-0.65167600
H	-1.71362700	-2.05004300	-0.71111000
H	-0.08291900	2.17430200	-0.63437900
H	2.40632800	2.31239700	-0.65227400
H	2.63368200	-1.84205800	0.53528700
H	0.17677600	-1.96774100	0.57772200
O	4.03749100	0.43590900	-0.11051800
C	4.87404500	-0.67207000	0.19102600
H	4.70635800	-1.51038300	-0.51275500
H	5.90756200	-0.31007400	0.08733700
H	4.71376700	-1.03187100	1.22594900

4be

C	-2.29183900	0.00113000	-0.00376900
C	-3.02296400	1.15422900	-0.36445300
C	-4.42287900	1.14952200	-0.35479700
C	-5.12286400	-0.00747200	0.01785900
C	-4.41026900	-1.16020500	0.37970100
C	-3.01038600	-1.15641000	0.36789800
C	-0.80670300	0.00515200	-0.01444300
C	-0.07915500	-1.14694000	-0.38669600
C	1.31809400	-1.14668700	-0.39778300
C	2.02297700	0.01236400	-0.03486900
C	1.31758000	1.16891200	0.33428200
C	-0.07980300	1.16195100	0.34352700
H	-2.48723000	2.05481200	-0.68201400
H	-4.97007400	2.05175100	-0.64806900
H	-6.21783800	-0.01078500	0.02623500
H	-4.94743400	-2.06572300	0.68126400
H	-2.46427500	-2.05367700	0.67710900

H	-0.61782900	-2.04682300	-0.69945700
H	1.86672100	-2.04186500	-0.70463800
H	1.86592100	2.07238900	0.61615400
H	-0.61843700	2.06214200	0.65545900
C	3.52946400	-0.00137700	0.00659900
F	4.05380700	-0.84831800	-0.91362300
F	3.99633800	-0.40414200	1.21819100
F	4.05462900	1.22721200	-0.22461000

4bf

C	-0.83446000	-0.14658900	0.03669000
C	-1.57948700	-1.21984700	-0.49957900
C	-2.97922200	-1.19273700	-0.50591200
C	-3.66506400	-0.09029200	0.02394200
C	-2.93765900	0.98383000	0.55733700
C	-1.53800900	0.95950600	0.56268300
C	0.64932600	-0.21315900	0.06063600
C	1.32149900	-1.42573000	0.33515900
C	2.71839100	-1.51246500	0.34555100
C	3.49271000	-0.37427200	0.07620100
C	2.86114000	0.84479000	-0.20175200
C	1.46611900	0.90587400	-0.20339800
H	-1.05178300	-2.07443600	-0.93601000
H	-3.53602700	-2.03299100	-0.93443900
H	-4.75993400	-0.06669700	0.01817100
H	-3.46395600	1.84807700	0.97660700
H	-0.98254900	1.79939200	0.98716700
H	0.72068300	-2.31107500	0.56701200
H	3.20241600	-2.46758500	0.57243200
H	3.42787200	1.75333800	-0.42465600
H	4.58599400	-0.43042900	0.08381700
F	0.88950900	2.09655100	-0.49282800

4bg

C	-1.01536000	-0.29012600	-0.05006400
C	-1.60026500	0.79066900	-0.74644400
C	-2.98598400	0.98803800	-0.72840000
C	-3.81963600	0.11729000	-0.01050600
C	-3.25303100	-0.95717600	0.68961100
C	-1.86706000	-1.15770200	0.66879500
C	0.44735900	-0.55752200	-0.08937900
C	0.91778000	-1.87513900	-0.24777800
C	2.28537500	-2.18153000	-0.27182500
C	3.21956600	-1.14988600	-0.13139800
C	2.78907000	0.17514700	0.03201000
C	1.41518100	0.47709200	0.05274600
H	-0.95986200	1.47248700	-1.31082900
H	-3.41918100	1.82793900	-1.28271400
H	-4.90306800	0.27728700	0.00522900
H	-3.89152500	-1.63931200	1.26156000
H	-1.42994200	-1.98746300	1.23456100
H	0.18055200	-2.67460200	-0.37671600
H	2.61423000	-3.21662800	-0.40710300
H	3.53008600	0.96984600	0.14748000
H	4.29310500	-1.36609400	-0.14998600
O	0.93162600	1.74307800	0.22697000
C	1.85123700	2.80489200	0.43233800
H	1.24084300	3.70992000	0.56772500
H	2.46700500	2.63791300	1.33710200
H	2.51935300	2.93979900	-0.44030800

4bh

C	-1.12046400	0.52871400	0.07255900
C	-1.95852700	1.14942500	-0.87729400
C	-3.33412600	0.88769200	-0.89766600
C	-3.89694300	0.00966400	0.03969900
C	-3.07429800	-0.60355000	0.99553800
C	-1.69733500	-0.34870400	1.01322100
C	0.33244500	0.86891000	0.09861600
C	0.68792600	2.22913400	0.21966200
C	2.02435400	2.64144100	0.26178600
C	3.04955500	1.69116200	0.17665100
C	2.72330500	0.33794100	0.03753700
C	1.38206800	-0.08040100	-0.00578100
H	-1.51862000	1.82736400	-1.61660700
H	-3.96750900	1.36883600	-1.65058800
H	-4.97270300	-0.19495300	0.02611400
H	-3.50703300	-1.28369900	1.73688700
H	-1.06425000	-0.82519300	1.76576500
H	-0.11513000	2.96868600	0.30171900
H	2.26272400	3.70513200	0.36603400
H	3.51422200	-0.41033100	-0.05666800

H	4.09936700	1.99912000	0.20946600
C	1.11334900	-1.55940000	-0.19264500
F	0.75624900	-2.17013400	0.97341200
F	0.12625700	-1.80026300	-1.08399200
F	2.21539200	-2.21483900	-0.63757800

Cs_2CO_3

C	-0.00003300	1.20155500	0.00002400
O	0.00001800	-0.14940000	0.00009000
O	1.13141500	1.81520400	-0.00052100
O	-1.13131200	1.81521300	0.00050100
Cs	2.75807000	-0.31870900	0.00002400
Cs	-2.75808300	-0.31870000	-0.00003700

CsBr

Cs	0.00000000	0.00000000	1.23397300
Br	0.00000000	0.00000000	-1.93910100

S1

C	7.40186500	-0.91325700	0.35098300
C	6.74085500	0.21129900	0.88226600
C	5.37597800	0.40155600	0.64869600
C	4.65551100	-0.53407300	-0.11464500
C	5.31099200	-1.65803800	-0.63962700
C	6.68256700	-1.84664700	-0.41253300
H	8.47323300	-1.06091600	0.53530500
H	7.30506000	0.93785100	1.48266000
H	4.80112000	1.25896400	1.02341800
H	4.71209300	-2.36751900	-1.22390500
H	7.19481100	-2.72333200	-0.82913000
N	3.22131100	-0.41059900	-0.38679400
N	2.70088800	0.65107300	0.01464400
Si	0.74966300	1.01427500	-0.18493400
C	1.21758200	2.62698900	-1.11627100
H	2.30481400	2.82413000	-1.11171700
H	0.86976400	2.55621100	-2.16498100
H	0.68812600	3.48554800	-0.66406100
C	0.36003600	-0.55376800	-1.20717400
H	-0.46167200	-1.09346300	-0.70269400
H	-0.00290200	-0.24945000	-2.20872000
H	1.24214700	-1.20865100	-1.32152300
C	0.64268800	1.03602500	1.72938800
H	0.38912400	2.06131000	2.06195600
H	-0.19890000	0.37762700	2.01026600
H	1.57033200	0.72159400	2.24130600
O	-1.06572400	1.60305900	-0.32949300
C	-2.17379100	0.98607100	0.01340200
O	-2.13440100	-0.10242000	0.72202800
O	-3.29969700	1.47692400	-0.39473300
Cs	-4.65681700	-0.79564600	0.09593900

S2

C	4.83398300	0.74795400	0.43928800
C	3.85482100	0.25715500	1.31913700
C	2.50041200	0.56146800	1.11421700
C	2.11996300	1.39228900	0.03593600
C	3.10613300	1.91707600	-0.81965100
C	4.45213300	1.57374900	-0.63574500
H	5.89121300	0.50580800	0.59919500
H	4.15162800	-0.36193300	2.17574800
H	1.71128100	0.19639800	1.78257300
H	2.78874300	2.58525900	-1.62856400
H	5.21293300	1.95679700	-1.32705800
N	0.75155300	1.77580000	-0.14921600
N	-0.10742900	0.88175300	-0.01381200
Si	-2.12147200	1.36258900	-0.10057600
C	-1.84746600	3.16755600	0.55835600
H	-2.42250800	3.88750100	-0.05953600
H	-2.21959200	3.25327800	1.59857000
H	-0.78719500	3.48109700	0.54134600
C	-1.89479400	1.59193800	-2.03696000
H	-2.75854500	2.13383600	-2.47334000
H	-0.97963000	2.16926300	-2.27520900
H	-1.82710200	0.61520600	-2.56048000
O	-1.96510100	0.49238700	1.64743700
C	-1.95398400	-0.71234300	1.12594500
O	-2.17149800	-0.65308500	-0.18749200
O	-1.66849200	-1.78348100	1.72268800
Cs	0.17786700	-2.14328400	-0.46234800
C	-4.08010400	1.36948600	-0.05347700
H	-4.51202500	0.57329200	-0.69219200
H	-4.46980200	1.22050400	0.97305700

H	-4.47621600	2.34065000	-0.41907200

Phenyl anion

C	-0.00001500	1.37775400	-0.00000200
C	1.20584900	0.65418100	0.00003100
C	1.17735400	-0.75713200	-0.00002500
C	0.00002000	-1.56843600	-0.00000300
C	-1.17733700	-0.75715600	0.00002500
C	-1.20586600	0.65415200	-0.00003000
H	-0.00002900	2.47793400	0.00000400
H	2.16679700	1.20136600	0.00008600
H	2.17463200	-1.25037400	-0.00009400
H	-2.17460600	-1.25042100	0.00011100
H	-2.16682600	1.20131500	-0.00008200

Cs^{+}

Cs	0.00000000	0.00000000	0.00000000

CO_3^{2-}

C	0.00000000	0.00008900	0.00000000
O	-1.14168200	-0.65878200	0.00000000
O	1.14152200	-0.65905900	0.00000000
O	0.00016000	1.31777500	0.00000000

References

1 C. Chauvier, L. Finck, S. Hecht and M. Oestreich, *Organometallics*, 2019, **38**, 4679–4686.

2 W. C. Still, M. Kahn and A. Mitra, *J. Org. Chem.*, 1978, **43**, 2923–2925.

3 H. Lv, R. D. Laishram, J. Li, Y. Zhou, D. Xu, S. More, Y. Dai and B. Fan, *Green Chem.*, 2019, **21**, 4055–4061.

4 M. J. Frisch, G. W. Trucks, H. B. Schlegel, G. E. Scuseria, M. A. Robb, J. R. Cheeseman, G. Scalmani, V. Barone, G. A. Petersson, H. Nakatsuji, X. Li, M. Caricato, A. V. Marenich, J. Bloino, B. G. Janesko, R. Gomperts, B. Mennucci, H. P. Hratchian, J. V. Ortiz, A. F. Izmaylov, J. L. Sonnenberg, Williams, F. Ding, F. Lipparini, F. Egidi, J. Goings, B. Peng, A. Petrone, T. Henderson, D. Ranasinghe, V. G. Zakrzewski, J. Gao, N. Rega, G. Zheng, W. Liang, M. Hada, M. Ehara, K. Toyota, R. Fukuda, J. Hasegawa, M. Ishida, T. Nakajima, Y. Honda, O. Kitao, H. Nakai, T. Vreven, K. Throssell, J. A. Montgomery Jr., J. E. Peralta, F. Ogliaro, M. J. Bearpark, J. J. Heyd, E. N. Brothers, K. N. Kudin, V. N. Staroverov, T. A. Keith, R. Kobayashi, J. Normand, K. Raghavachari, A. P. Rendell, J. C. Burant, S. S. Iyengar, J. Tomasi, M. Cossi, J. M. Millam, M. Klene, C. Adamo, R. Cammi, J. W. Ochterski, R. L. Martin, K. Morokuma, O. Farkas, J. B. Foresman and D. J. Fox, *Gaussian 16, Rev. A.03*, Gaussian, Inc., Wallingford, CT, 2016.

5 J. Tao, J. P. Perdew, V. N. Staroverov and G. E. Scuseria, *Phys. Rev. Lett.*, 2003, **91**, 146401.

6 a) S. Grimme, S. Ehrlich and L. Goerigk, *J. Comput. Chem.*, 2011, **32**, 1456–1465; b) S. Grimme, J. Antony, S. Ehrlich and H. Krieg, *J. Chem. Phys.*, 2010, **132**, 154104.

7 F. Weigend and R. Ahlrichs, *Phys. Chem. Chem. Phys.*, 2005, **7**, 3297–3305.

8 a) A. Bergner, M. Dolg, W. Küchle, H. Stoll and H. Preuß, *Mol. Phys.*, 1993, **80**, 1431–1441; b) D. Andrae, U. Häußermann, M. Dolg, H. Stoll and H. Preuß, *Theor. Chim. Acta*, 1990, **77**, 123–141; c) G. Igel-Mann, H. Stoll and H. Preuss, *Mol. Phys.*, 1988, **65**, 1321–1328; d) L. von Szentpály, P. Fuentealba, H. Preuss and H. Stoll, *Chem. Phys. Lett.*, 1982, **93**, 555–559.

9 J.-D. Chai and M. Head-Gordon, *Phys. Chem. Chem. Phys.*, 2008, **10**, 6615–6620.

10 A. V. Marenich, C. J. Cramer and D. G. Truhlar, *J. Phys. Chem. B*, 2009, **113**, 6378–6396.

11 a) P. Pracht, F. Bohle and S. Grimme, *Phys. Chem. Chem. Phys.*, 2020, **22**, 7169–7192; b) S. Grimme, *J. Chem. Theory Comput.*, 2019, **15**, 2847–2862.

12 G. Luchini, J. V. Alegre-Requena, I. Funes-Ardoiz and R. S. Paton, *F1000Research*, 2020, **9**, 291.

13 S. Grimme, *Chem. – Eur. J.*, 2012, **18**, 9955–9964.

14 Y.-P. Li, J. Gomes, S. Mallikarjun Sharada, A. T. Bell and M. Head-Gordon, *J. Phys. Chem. C*, 2015, **119**, 1840–1850.

15 C. Y. Legault, *CYLview20*, Université de Sherbrooke, 2020.

16 E. D. Glendening, J. K. Badenhoop, A. E. Reed, J. E. Carpenter, J. A. Bohmann, C. M. Morales, C. R. Landis and F. Weinhold, *NBO 6.0*, Theoretical Chemistry Institute, University of Wisconsin, Madison, 2013.

17 *The PyMOL Molecular Graphics System, Version 3.2.0a*, Schrödinger, LLC.

18 a) T. Lu, *J. Chem. Phys.*, 2024, **161**; b) T. Lu and F. Chen, *J. Comput. Chem.*, 2012, **33**, 580–592.

19 W. Humphrey, A. Dalke and K. Schulten, *J. Mol. Graph.*, 1996, **14**, 33–38.

NMR Spectra

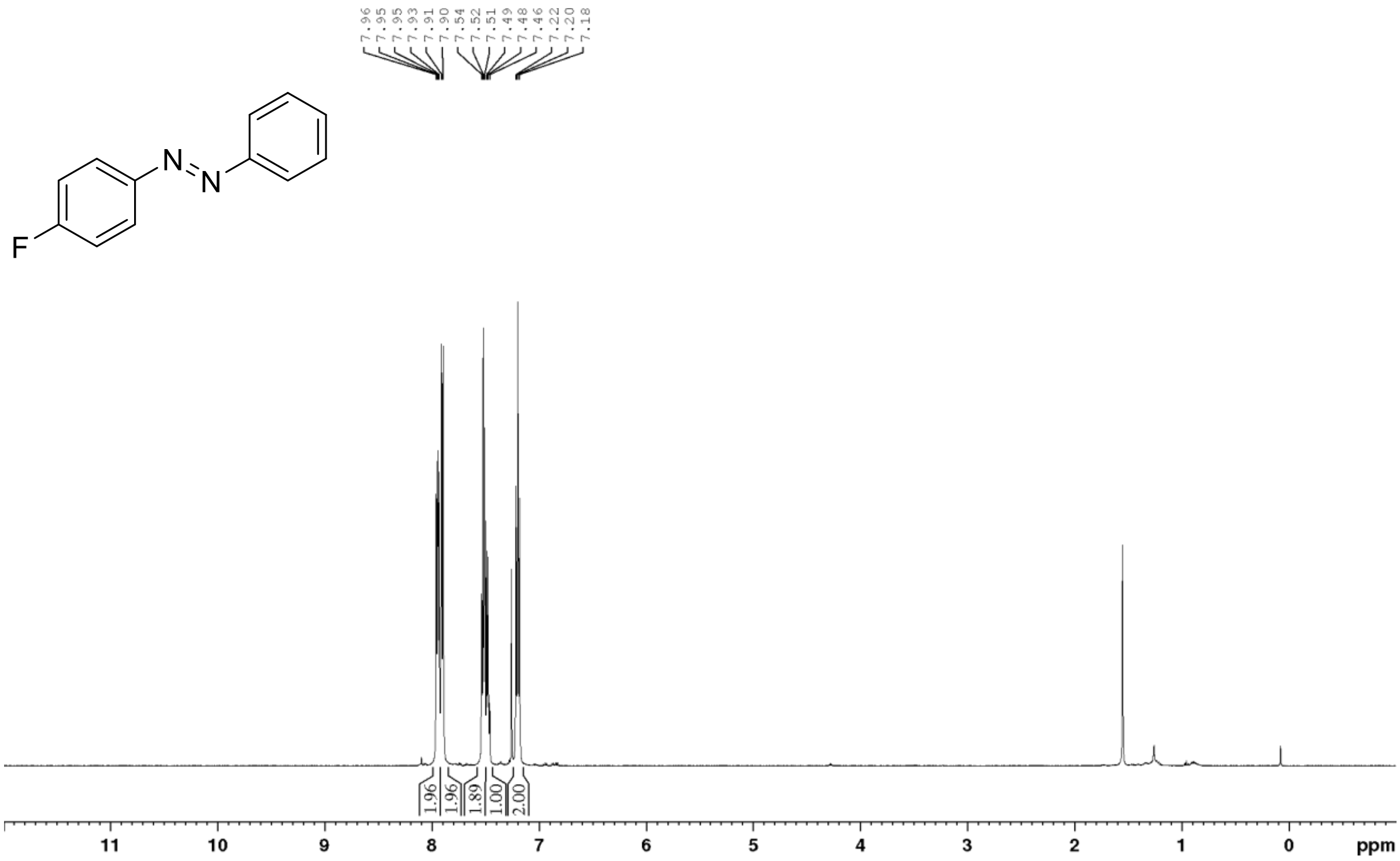

Figure S13. ^{1}H NMR (500 MHz, $CDCl_3$) of (*E*)-1-(4-Fluorophenyl)-2-phenyldiazene (**3bc**).

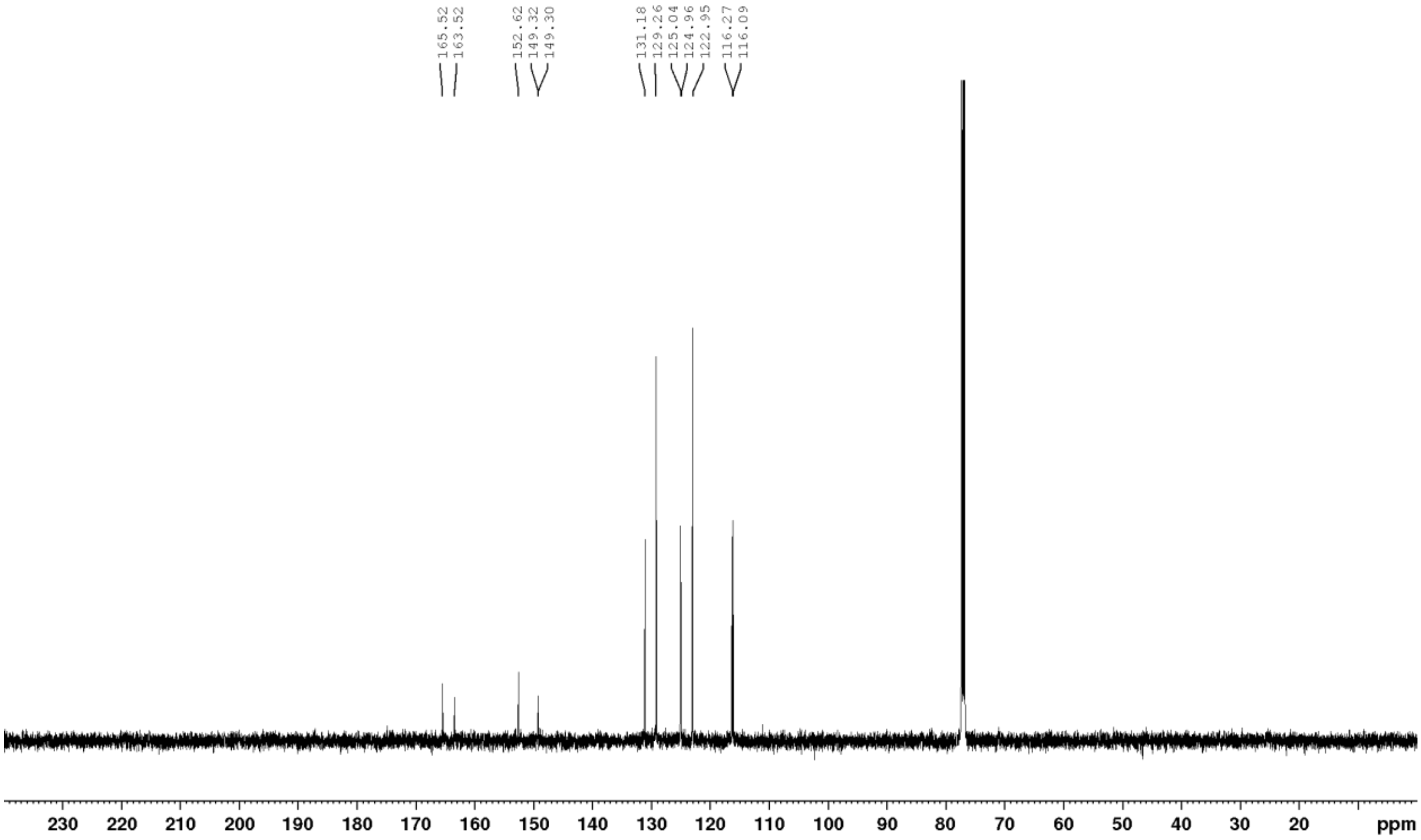

Figure S14. $^{13}C\{^{1}H\}$ NMR (126 MHz, $CDCl_3$) of (*E*)-1-(4-Fluorophenyl)-2-phenyldiazene (**3bc**).

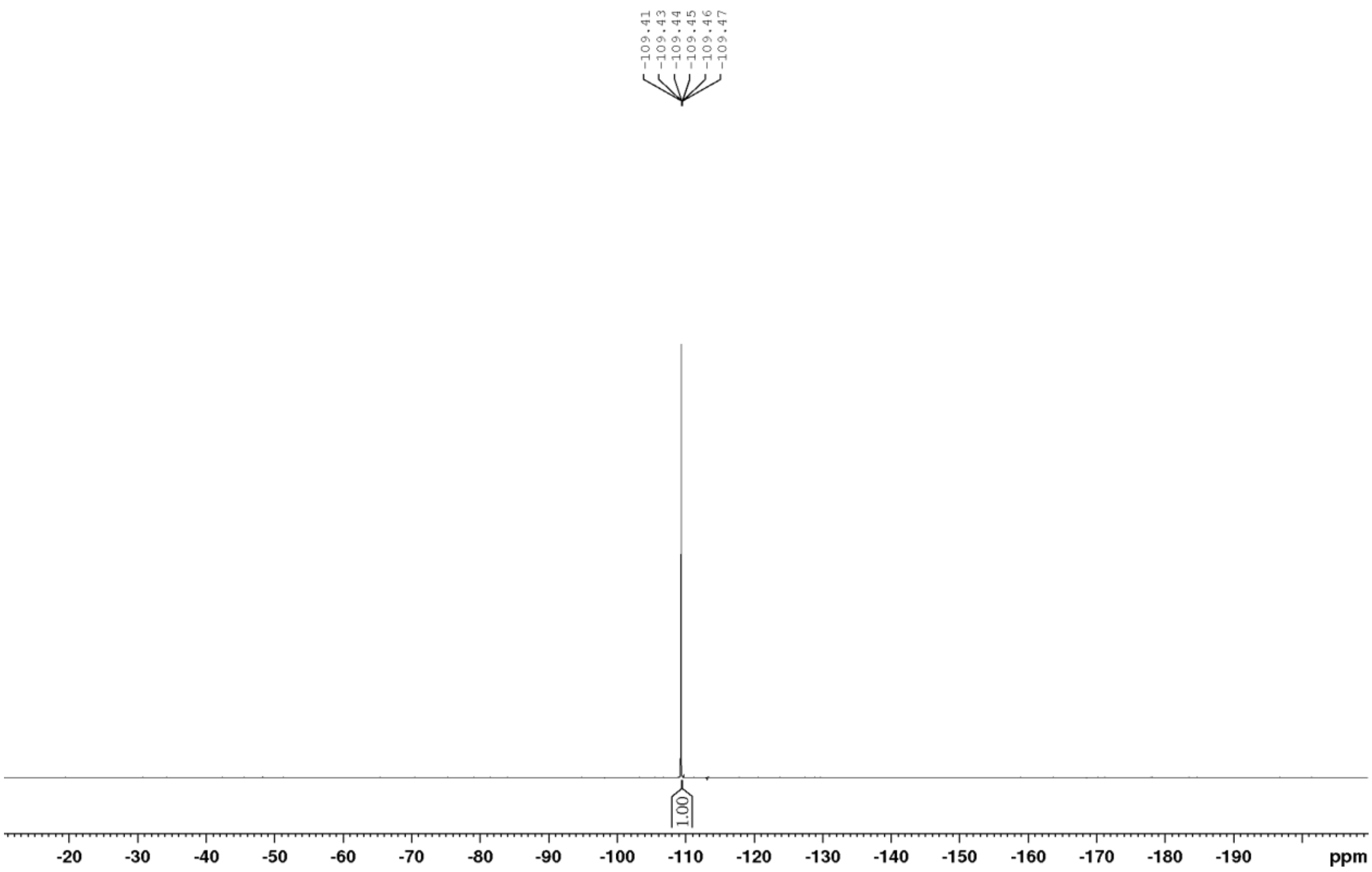

Figure S15. ^{19}F NMR (471 MHz, $CDCl_3$) of (*E*)-1-(4-Fluorophenyl)-2-phenyldiazene (**3bc**).

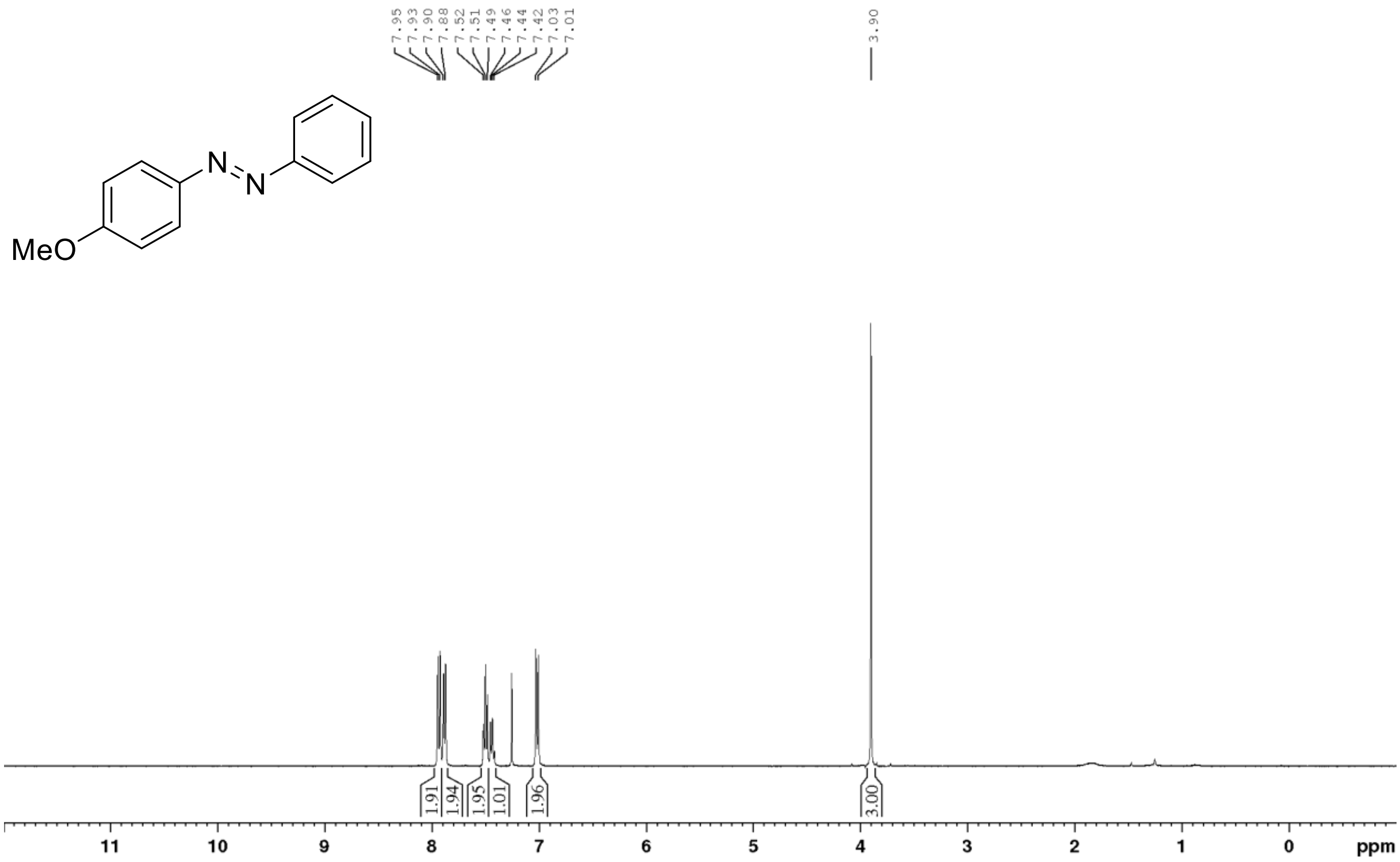

Figure S16. ^{1}H NMR (500 MHz, $CDCl_3$) of (*E*)-1-(4-Methoxyphenyl)-2-phenyldiazene (**3bd**).

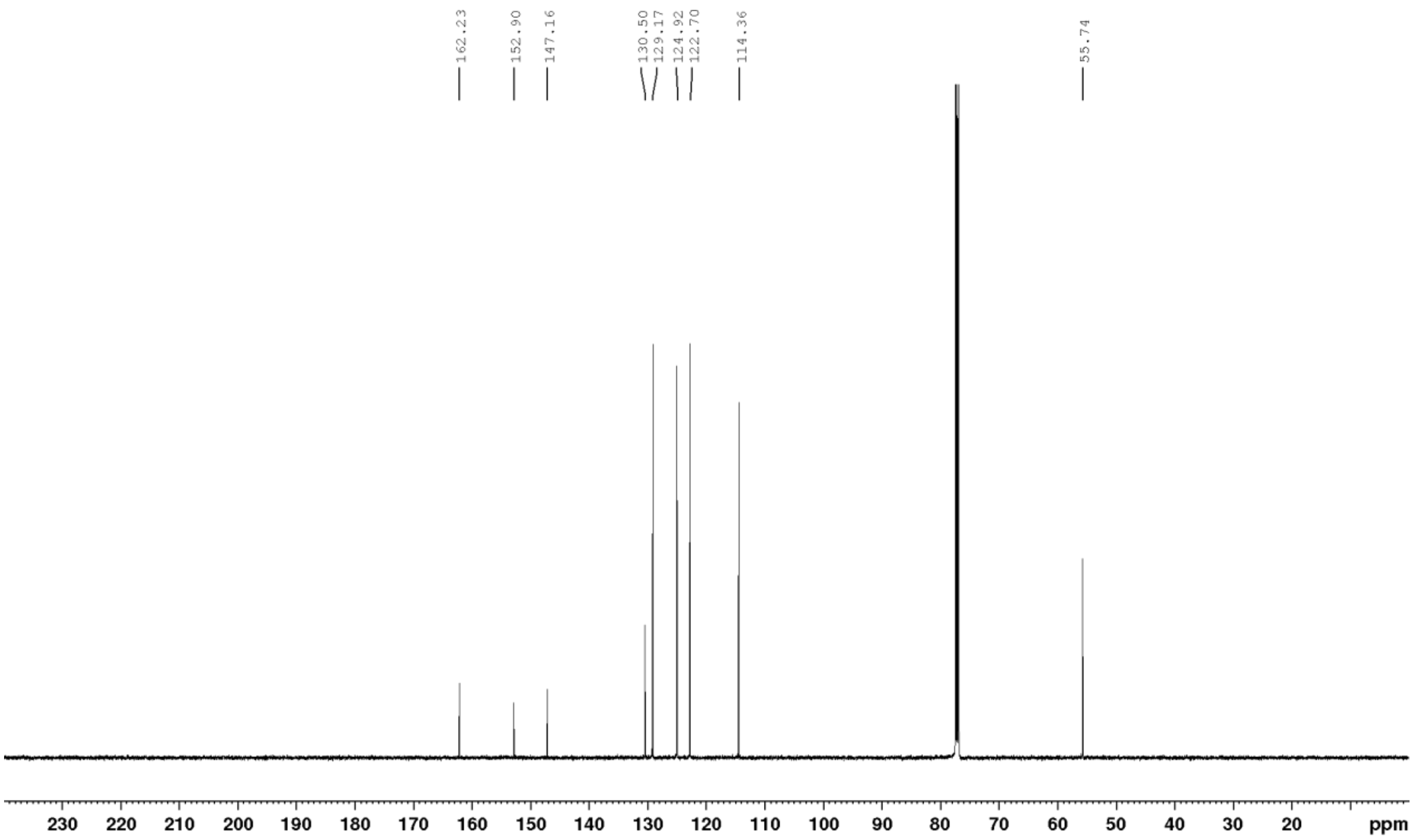

Figure S17. $^{13}C\{^{1}H\}$ NMR (126 MHz, $CDCl_3$) of (*E*)-1-(4-Methoxyphenyl)-2-phenyldiazene (**3bd**).
